# Nox4 Promotes RANKL-Induced Autophagy and Osteoclastogenesis via Activating ROS/PERK/eIF-2α/ATF4 Pathway

**DOI:** 10.3389/fphar.2021.751845

**Published:** 2021-09-28

**Authors:** Jing Sun, Wugui Chen, Songtao Li, Sizhen Yang, Ying Zhang, Xu Hu, Hao Qiu, Jigong Wu, Shangcheng Xu, Tongwei Chu

**Affiliations:** ^1^ Department of Orthopedics, Xinqiao Hospital of Army Medical University, Chongqing, China; ^2^ Department of Spinal Surgery, 306 Hospital of PLA, Beijing, China; ^3^ The Center of Laboratory Medicine, The Sixth People’s Hospital of Chongqing, Chongqing, China

**Keywords:** NOX4, RANKL, autophagy, ROS, osteoclastogenesis, UPR, PERK/eIF-2α/ATF4 pathway

## Abstract

Receptor activator of nuclear factor-κB ligand (RANKL) has been found to induce osteoclastogenesis and bone resorption. However, the underlying molecular mechanisms remain unclear. Via conducting a series of biochemical experiments with *in vitro* cell lines, this study investigated the role and mechanism of NADPH oxidase 4 (Nox4) in RANKL-induced autophagy and osteoclastogenesis. In the current study, we found that RANKL dramatically induced autophagy and osteoclastogenesis, inhibition of autophagy with chloroquine (CQ) markedly attenuates RANKL-induced osteoclastogenesis. Interestingly, we found that the protein level of Nox4 was remarkably upregulated by RANKL treatment. Inhibition of Nox4 by 5-O-methyl quercetin or knockdown of Nox4 with specific shRNA markedly attenuated RANKL-induced autophagy and osteoclastogenesis. Furthermore, we found that Nox4 stimulated the production of nonmitochondrial reactive oxygen species (ROS), activating the critical unfolded protein response (UPR)-related signaling pathway PERK/eIF-2α/ATF4, leading to RANKL-induced autophagy and osteoclastogenesis. Blocking the activation of PERK/eIF-2α/ATF4 signaling pathway either by Nox4 shRNA, ROS scavenger (NAC) or PERK inhibitor (GSK2606414) significantly inhibited autophagy during RANKL-induced osteoclastogenesis. Collectively, this study reveals that Nox4 promotes RANKL-induced autophagy and osteoclastogenesis via activating ROS/PERK/eIF-2α/ATF4 pathway, suggesting that the pathway may be a novel potential therapeutic target for osteoclastogenesis-related disease.

## Introduction

Bone homeostasis is maintained through elaborate bone remodeling via coordinated bone formation and bone resorption (Upadhyay et al., 2015). Osteoclasts are the principal cells responsible for bone resorption (Chung et al., 2014). Osteoclasts, characterized as tartrate-resistant acid phosphatase (TRAP)-positive, are derived from the hematopoietic monocyte/macrophage lineage, and fuse to form multinucleated cells by an orchestrated process (Bloemen et al., 2010; Boyce, 2013a). The excessive differentiation of osteoclasts is the pathological basis of a variety of osteolytic diseases, such as postmenopausal osteoporosis, inflammatory arthritis, and Paget disease of bone (Chung and Van Hul, 2012; Johnson et al., 2015). Therefore, there is no doubt that identifying pharmacological inhibitors targeting osteoclasts differentiation will help the development of new prophylactic and therapeutic strategies for osteolytic bone lesions (Boyce, 2013b). The differentiation and maturation of osteoclasts is a complicated process that is regulated by various cytokines (Oikawa et al., 2013). RANKL, one of the tumor necrosis factor superfamily (Pacifici, 2012), has been demonstrated to interact with the RANK receptor, expressed on osteoclast precursors, to activate multiple osteoclastogenesis-related signaling pathways (NF-κB, Src, MAPK, etc.), releasing nuclear transcription factors (NFATc1, AP-1, etc.) and regulating the expression of osteoclastogenesis-related genes, which induce the differentiation and maturation of osteoclasts in the bone microenvironment (Feng and Teitelbaum, 2013; Kimura et al., 2014). Moreover, denosumab, a monoclonal antibody with activity against RANKL, has been demonstrated to be effective in the prevention and treatment of osteolytic disease (Dahiya et al., 2015). However, the detailed mechanisms of osteoclastogenesis induced by RANKL remain unclear.

Autophagy delivers cellular components to the lysosome for degradation (Kim and Lee, 2014), which is strongly associated with the development and differentiation of multiple cell types, including lymphocytes, adipocytes, chondrocytes, neurons, and erythrocytes (Zeng and Zhou, 2008; Colosetti et al., 2009; Singh et al., 2009; Stappenbeck, 2010; Rogov et al., 2014; De Meyer et al., 2015). Previous studies have revealed that the induction of autophagy contributes to osteoclastogenesis in response to glucocorticoid treatment, hypoxic conditions and microgravity *in vitro* (Zhao et al., 2012; Sambandam et al., 2014; Shi et al., 2015). Moreover, several key autophagy-regulated proteins such as Atg5 and LC3 have been shown to participate in osteoclast bone resorption by directing lysosomal content secretion into the extracellular space (DeSelm et al., 2011). But it is still unclear that the molecular mechanism of RANKL-induced autophagy in osteoclastogenesis.

Nox4 is a member of NADPH oxidase family that is a cellular enzyme devoted to the production of reactive oxygen species (ROS). Nox4, first identified by Shiose et al. in kidney in 2001, is widely distributed in human tissues. The human Nox4 gene is located on chromosome 11q14.2-q21 (Shiose et al., 2001). The transcriptional regulation of *Nox4* by a series of stimulus such as hypoxia, shear stress, high glucose, angiotensinII (AngII). A large number of transcription factors (E2F, Nrf2, HIF-1α, NF-κB, oct-1, sp3, and sp1, c-jun, STAT3) have been demonstrated to binding the promoter region of *Nox4* gene and enhance the mRNA level of *Nox4* (Guo and Chen, 2015). Nox4 is composed of conserved transmembrane domains, FAD- and NADPH-binding domains in the C-terminal region, and two heme groups (Zhang et al., 2013; Panday et al., 2015). Previous studies have demonstrated that Nox4 participates in different kinds of physiological and pathological process such as cell proliferation (Ateghang et al., 2006), cell migration (Meng et al., 2008), cell death (Pedruzzi et al., 2004), fibrosis (Piera-Velazquez and Jimenez, 2015), hypertension (Paravicini et al., 2004), and cancer (Tang et al., 2018). Recently, several studies reported that Nox4 plays an important role in autophagy induction (Sciarretta et al., 2013; Chen et al., 2019). However, whether Nox4-induced autophagy is involved in RANKL promoted osteoclastogenesis is still unclear.

In the current study, we firstly found that the upregulation of Nox4 protein level contributes to the activation of autophagy during RANKL-induced osteoclastogenesis. Furthermore, we observed that Nox4-mediated nonmitochondrial ROS upregulation dramatically promoted RANKL-induced autophagy activation and osteoclastogenesis. Either knockdown of Nox4 or blocking ROS inhibited the activation of the PERK/eIF-2α/ATF4 signaling pathway and autophagy during RANKL-induced osteoclastogenesis. In summary, our study reveals that Nox4 promotes RANKL-induced autophagy and osteoclastogenesis via activating ROS/PERK/eIF-2α/ATF4 pathway, which may facilitate the development of novel therapeutic strategies for osteoclastogenesis-related diseases.

## Materials and Methods

### Reagents and Antibodies

Recombinant murine soluble RANKL (315-11) was purchased from Peprotech (Rocky Hill, NJ, United States). CQ phosphate (S4157) was obtained from Selleck (Shanghai, China). ML171 (492002) was acquired from Millipore Corporation (Temecula, CA, United States). Diphenyleneiodonium chloride (DPI) (sc-202584) and 5-O-methyl quercetin (sc-483298) were purchased from Santa Cruz Biotechnology (Dallas, TX, United States). N-acetyl-L-cysteine (NAC) (A9165) and Mito-TEMPO (SML0737) were purchased from Sigma-Aldrich (St. Louis, MO, United States). GSK2795039 (HY-18950) and GSK2606414 (HY-18072) were purchased from MCE (Shanghai, China).

Both the mouse Nox4 short hairpin RNA (shRNA)-containing retrovirus and the corresponding empty vector were designed and synthesized by HanBio (Shanghai, China). The mRFP-GFP-LC3-containing adenovirus (HB-AP210 0001) was provided by HanBio. Primary antibodies against Nox3 (20065-1-AP), Nox4 (14347-1-AP), ATF6 (24169-1-AP), ATF4 (10835-1-AP), XBP1S (24868-1-AP), GAPDH (60004-1-Ig), ERp57/ERp60 (15967-1-AP), and VDAC1/Porin (55259-1-AP) were obtained from Proteintech (Wuhan, Hubei, China). Primary antibodies against LC3A/B (4108S), PERK (C33E10) (3192S), phospho-PERK (Thr980; 16F8) (3179S), eIF2α (D7D3) XP^®^ (5324T), and phospho-eIF2α (Ser51; D9G8) XP^®^ (3398T) were purchased from Cell Signaling Technology (Danvers, Massachusetts, United States). Primary antibodies against Nox1 (ab131088) and Nox2/gp91phox (ab129068) were obtained from Abcam (Cambridge, United Kingdom).

### Cell Culture

The RAW264.7 mouse monocyte/macrophage cell line was purchased from the Cell Culture Center of the Chinese Academy of Sciences (Shanghai, China) and cultured in Dulbecco’s modified Eagle’s medium (11995065, Gibco, Grand Island, NY, United States) supplemented with 10% fetal bovine serum (10091148, Gibco) and 100 U/ml penicillin and 100 mg/ml streptomycin (15070063, Gibco). The cells were incubated in a humidified atmosphere with 95% air and 5% CO_2_ at 37°C. To induce osteoclast differentiation, RAW264.7 cells were stimulated with 100 ng/ml RANKL and further cultured for the indicated times.

### Retrovirus-Mediated Stable Knockdown of Nox4

RAW264.7 cells were plated and cultured in 35 mm dishes. When the confluence reached 50%, the cells were transfected with retrovirus encoding Nox4 shRNAs or scrambled shRNA at a multiplicity of infection (MOI) of 100 for 24 h according to the manufacturer’s instructions (HanBio). The nucleotide sequences were as follows: sh-Nox4-1, 5′-GCA​GGA​GAA​CCA​GGA​GAT​TGT-3′; sh-Nox4-2, 5′-GCA​TGG​TGG​TGG​TGC​TAT​TCC-3′; sh-Nox4-3, 5′-GGT​ATA​CTC​ATA​ACC​TCT​TCT-3′; and sh-NC, 5′-TTC​TCC​GAA​CGT​GTC​ACG​T-3′. RAW264.7 cells with stable knockdown of Nox4 expression were screened by the addition of 2 µg/ml puromycin (HB-PU-1000, HanBio) to the culture medium for 48 h. Then, the stable cells were digested with 0.25% trypsin (T1350, Solarbio, Beijing, China) and seeded on 35 mm dishes at a density of 8 × 10^4^ cells/dish and incubated overnight for attachment. The next day, adherent cells were treated with or without RANKL (100 ng/ml) for 3 days. The knockdown efficiency of the three Nox4 shRNAs was measured using western blotting, and the most effective was selected for use in subsequent experiments.

### Osteoclast Differentiation Assay

Osteoclast formation was measured by quantifying cells positively stained with TRAP. Briefly, RAW264.7 cells were incubated at a density of 1 × 10^4^ cells/well in 24-well plates (Corning, New York, NY, United States) overnight. After stimulation with RANKL (100 ng/ml) and various concentrations of different pharmacological reagents for 6 days, the cells were fixed with 4% paraformaldehyde (AR1069, BOSTER, Wuhan, Hubei, China) for 30 min at room temperature and then stained by using a Tartrate Resistant Acid Phosphatase Assay Kit (P0332, Beyotime Biotechnology, Shanghai, China) according to the manufacturer’s instructions. TRAP-positive and multinucleated cells containing three or more nuclei were considered osteoclasts. For each well, the osteoclasts were observed and counted under a light microscope (Leica, Wetzlar, Germany).

### Osteoclast Bone Resorption Pit Formation Assay

To confirm the bone resorption ability of differentiated osteoclasts, RAW264.7 cells were seeded at a density of 2 × 10^4^ cells/well overnight in 24-well Osteo Assay Surface plates (Corning) coated with hydroxyapatite matrix. Then, the cells were incubated with RANKL (100 ng/ml) or in the presence of various concentrations of different pharmacological reagents. The medium was replaced every 3 days. After 7 days of culture, the cells were removed using a 10% sodium hypochlorite solution, and the wells were stained with 1% toluidine blue. The plate was washed twice with distilled water and air dried at room temperature. The bone resorption pits in each well were observed and photographed by a light microscope. The pit formation area was analyzed using the ImageJ software (National Institutes of Health, Bethesda, MD, United States).

### Quantitative Real-Time PCR

Total cellular RNA was extracted using RNAiso plus reagent (9,108, Takara, Kyoto, Japan) according to the manufacturer’s instructions. Subsequently, the total RNA concentration was determined with a NanoDrop 2.0 spectrophotometer (Thermo Fisher Scientific, Pittsburgh, PA, United States) and the RNA was reverse transcribed to cDNA using a PrimeScript™ RT reagent kit with gDNA Eraser (RR047A, Takara) according to the manufacturer’s instructions. Subsequently, qRT-PCR assays were performed by using a SYBR Premix Ex Taq™ II (2×) kit (RR820A, Takara) according to the manufacturer’s instructions and run on an ABI 7500 Real-Time PCR Detection System (Foster City, CA, United States). The reactions were performed using the following parameters: 95°C for 30 s followed by 40 cycles of 95°C for 5 s and 60°C for 30 s. The primer nucleotide sequences used for qRT-PCR are listed in Supplementary Table S1. All primer sets for mRNA amplification were purchased from Sangon Biotech (Shanghai) Co., Ltd. (Shanghai, China). The relative expression levels of the target gene were normalized with respect to the levels of β-actin expression and calculated using the 2^−△△CT^ method.

### Transmission Electron Microscopy

RAW264.7 cells were cultured with the indicated treatments for 3 days. Then, the cells were digested with 0.25% trypsin, centrifuged (2000 rpm) for 10 min and fixed with 2.5% glutaraldehyde overnight at 4°C. Subsequently, the cells were postfixed with 1% osmium tetroxide for 1.5 h, washed and stained in 3% aqueous uranyl acetate for 1 h. Thereafter, the samples were washed again, dehydrated with a graded series of increasing ethanol concentrations to 100% and embedded in Epon-Araldite resin. Subsequently, the ultrathin sections were cut using a Reichert ultramicrotome (Reichert, New York, NY, United States) and counterstained with 0.3% lead citrate. Then, the ultrastructure of autophagic vacuoles (autophagosomes and autolysosomes) was observed under a transmission electron microscope (Leica), and images were captured.

### Autophagic Flux Assessment

After growth to 50% confluence in 35 mm dishes, the cells were transfected with adenovirus expressing mRFP-GFP-LC3 for 24 h using a MOI of 1,000, according to the manufacturer’s instructions (HanBio). Then, the cell growth medium was replaced with fresh complete medium for another 24 h. Afterward, the transfected cells were digested with 0.25% trypsin and seeded on confocal Petri dishes (NEST, Wuxi, Jiangsu, China) at a density of 5 × 10^4^ cells/dish and incubated overnight for attachment. Thereafter, adherent cells were treated with the various indicated treatments for 3 days. The treated cells were washed with phosphate buffer saline (PBS) (AR0032, BOSTER) and viewed with a laser scanning confocal microscope (Leica). GFP loses its fluorescence in acidic lysosomal conditions, whereas mRFP does not. Therefore, yellow (merged GFP signal and RFP signal) puncta represent early autophagosomes, whereas puncta detectable only as red (RFP signal alone) indicate late autolysosomes that are formed by autophagosome fusion with lysosomes. Autophagic flux was ultimately assessed by quantifying the mRFP and GFP puncta per cell. The number of GFP and mRFP puncta was determined by manually counting 30 cells randomly in 5 fields per dish, and the average number of puncta per cell was calculated.

### Endoplasmic Reticulum-Tracker Staining in Living Cells

RAW264.7 cells (5 × 10^4^) were plated on confocal Petri dishes and allowed to attach overnight. Then, the cells were cultured with the indicated treatments for the indicated times. Next, ER-Tracker (E34250, Thermo Fisher Scientific) was added directly to the culture medium at 500 nM and incubated with cells for 30 min in a 37°C humidified incubator containing 5% CO_2_. Then, the cells were washed with PBS and immediately observed under a laser scanning confocal microscope.

### Immunofluorescence Staining for Nox4 Localization

The colocalization of Nox4 with the ER was detected by double-labeling immunofluorescence. Briefly, RAW264.7 cells were seeded on confocal Petri dishes at a density of 5 × 10^4^ cells/dish overnight. Then, the cells were incubated with or without RANKL (100 ng/ml) for 3 days. Thereafter, the cells were stained with ER-Tracker (the detailed experimental procedure is described above) and fixed in 4% paraformaldehyde for 20 min at 37°C. Then, the cells were permeabilized with 0.1% Triton X-100 (P1080, Solarbio) for 10 min and blocked with 10% normal goat serum (AR0009, BOSTER) for 1 h at 37°C. Subsequently, the cells were incubated with a rabbit polyclonal anti-Nox4 antibody (1:25) in a humidified chamber at 4°C overnight. Then, the cells were washed and incubated with Alexa Fluor 488-labeled goat anti-rabbit IgG (1:500) (A0423, Beyotime Biotechnology) for 1.5 h at 37°C in the dark. DAPI Staining Solution (C1005, Beyotime Biotechnology) was used to counterstain the cell nuclei. Finally, the cells were observed using a laser scanning confocal microscope.

### Subcellular Fractionation Assay

RAW264.7 cells were seeded in 6 cm dishes overnight. Then, the cells were cultured with the indicated treatments for the indicated times. Subsequently, the cells were digested with 0.25% trypsin and centrifuged (1,000 rpm) for 5 min. Mitochondria and the ER were extracted from cells using a Cell Mitochondria Isolation kit (C3601, Beyotime Biotechnology) and Endoplasmic Reticulum Isolation kit (BB-31454-1, BestBio Science, Shanghai, China) according to the manufacturer’s instructions. The fractions of mitochondria and ER were lysed in RIPA buffer (P0013, Beyotime Biotechnology) that contained a protease and phosphatase inhibitor cocktail (P1050, Beyotime Biotechnology) for subsequent western blot analysis.

### Western Blot Analysis

RAW264.7 cells were lysed in RIPA buffer that contained a protease and phosphatase inhibitor cocktail. After centrifugation at 14,000 g for 5 min at 4°C, the concentrations of protein were measured using a BCA protein assay kit (P0010, Beyotime Biotechnology). Subsequently, equal amounts of protein (40 μg) were separated by 6, 8 or 12%/5% (w/v) sodium dodecyl sulfate-polyacrylamide gel electrophoresis (Bio-Rad, Hercules, CA, United States) and transferred to polyvinylidene difluoride membranes (Millipore). The membranes were incubated with anti-GAPDH, anti-Nox4, anti-LC3A/B, anti-ERp57/ERp60, anti-VDAC1/Porin, anti-Nox3, anti-Nox2/gp91phox, anti-Nox1, anti-PERK (C33E10), anti-phospho-PERK (Thr980) (16F8), anti-ATF6, anti-ATF4, anti-XBP1S, anti-eIF2α (D7D3) XP^®^, and anti-phospho-eIF2α (Ser51; D9G8) XP^®^ antibodies separately overnight at 4°C. Then, the membranes were incubated with horseradish peroxidase-conjugated goat anti-mouse/rabbit IgG (H + L) secondary antibodies (1:5,000) (ZB-2305/ZB-2301, ZSGB-BIO, Beijing, China) for 1 h at room temperature. Finally, the protein bands of interest on the membranes were visualized with a chemiluminescence substrate kit (WBKLS0100, Millipore Corporation) using an Image Quant LAS4000 instrument (GE, Boston, MA, United States). The band intensity was quantified by densitometric analysis using Image J software.

### Determination of Intracellular and ER ROS

The intracellular production of ROS was detected by staining cells with a ROS Assay kit (S0033S, Beyotime Biotechnology). Briefly, RAW264.7 cells (5 × 10^4^) were seeded in confocal Petri dishes overnight. Then, the adherent cells were cultured under conditions with various treatments for the indicated times. Subsequently, the cells were stained with ER-Tracker (the detailed experimental procedure is described above) and washed with PBS. Then, 2′, 7′-dichlorodihydrofluorescein diacetate (DCFH-DA), which was added directly to serum-free medium, was diluted to a final concentration of 10 μM and incubated with cells for 30 min at 37°C in a humidified incubator containing 5% CO_2_. DCFH-DA diffuses into cells and is deacetylated by cellular esterases to nonfluorescent 2′,7′-dichlorodihydrofluorescein, which can be oxidized by ROS to produce highly fluorescent 2′,7′-dichlorofluorescein (DCF). The green fluorescence intensity is proportional to the levels of ROS within a cell. The cells were then washed three times with PBS, and the fluorescence intensity was observed using a laser scanning confocal microscope.

### Measurement of Mitochondrial ROS

The levels of mitochondrial ROS were measured by staining cells with MitoSOX™ Red Mitochondrial Superoxide Indicator (M36008, Thermo Fisher Scientific). Briefly, RAW264.7 cells were seeded at a density of 3 × 10^3^ cells/well on 96-well plates and allowed to attach overnight. Then, the cells were cultured with various treatments for the indicated times. Subsequently, the cells were incubated with MitoSOX at a final concentration of 5 μM for 15 min at 37°C in a humidified incubator containing 5% CO_2_. Then, the cells were washed three times with PBS, and the fluorescence intensity was immediately measured with a Varioskan Flash Spectral Scanning Multimode Reader (Thermo Fisher Scientific). The red fluorescence intensity is proportional to the levels of mitochondrial ROS within the cell.

### Statistical Analysis

The SPSS software, version 13.0 (SPSS, Inc., Chicago, IL, United States) and GraphPad Prism 7.0 (GraphPad Software) were used for statistical analysis. Data are presented as the mean ± standard deviation (SD). The unpaired Student’s t-test was utilized to calculate the *p* value of the statistic difference between 2 independent datasets. One-way analysis of variance (ANOVA) was used to analyze the significance among three or more independent datasets, and the Tukey’s *post-hoc* multiple comparison testing was performed for multiple comparisons when the probability for ANOVA was statistically significant. Methods of non-parametric statistics such as the Mann–Whitney and Kruskal–Wallis tests were used when variances did not pass the Levene test for normality or homogeneity. All the statistical tests were two-sided and a *p* value of <0.05 was considered statistically significant in all cases.

## Results

### RANKL Induces Osteoclastogenesis and Bone Resorption via Autophagy

Consistent with previous studies, we found that RANKL enhanced the proportion of fused multinuclear cells (Supplementary Figure S1A) and the expression levels of osteoclastogenesis-related genes (TRAP, Cath K and MMP-9; Supplementary Figure S1B), inducing the formation of TRAP-positive multinuclear (≥3) osteoclasts and bone resorption pits in RAW264.7 cells (Supplementary Figure S1C). These results indicate that RANKL induced the differentiation and subsequent bone resorption activity of osteoclasts *in vitro*, which is consistent with previous findings (Asai et al., 2014).

**FIGURE 1:**
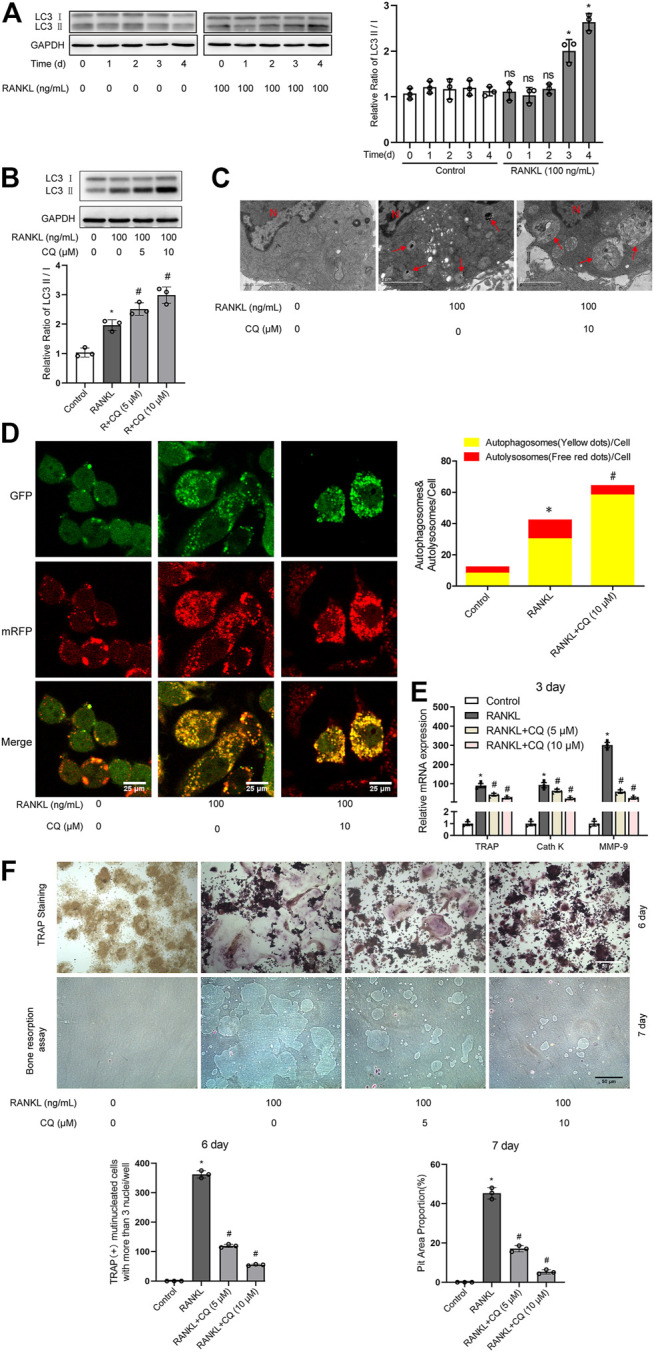
RANKL induces osteoclastogenesis and bone resorption via autophagy. **(A)** RAW264.7 cells were seeded overnight and incubated with or without RANKL (100 ng/ml) for the indicated times. The protein levels of LC3-I and LC3-II were tested by western blot, and the ratio of LC3-II/LC3-I was quantified by Image J. **(B)** RAW264.7 cells were seeded overnight and treated with the autophagy inhibitor CQ (5 and 10 μM) in the presence of RANKL (100 ng/ml) for 3 days. The ratio of LC3-II/LC3-I was quantified as described in **(A)**. **(C)** RAW264.7 cells were seeded overnight and treated with CQ (10 μM) in the presence of RANKL (100 ng/ml) for 3 days. The autophagic vacuoles (autophagosomes and autolysosomes) were monitored by TEM. Representative TEM images are shown, and the typical autophagic vacuoles are marked with red arrows. **(D)** After transfection with Ad-mRFP-GFP-LC3 for 48 h, RAW264.7 cells were treated as described in **(C)**. Then, autophagic flux was assessed by quantifying the number of mRFP and GFP puncta per cell under a laser scanning confocal microscope. Representative images of mRFP and GFP puncta are shown, together with the quantification of autophagosomes and autolysosomes. **(E)** RAW264.7 cells were treated as described in **(B)**. Then, the mRNA expression levels of TRAP, Cath K and MMP-9 were detected by qRT-PCR. **(F)** RAW264.7 cells were treated as described in **(B)** for the indicated times. Then, TRAP staining and bone resorption assays were performed to evaluate the formation of TRAP-positive multinucleated (≥3) osteoclasts and bone resorption pits respectively. All the data derived from at least three independent replicates and were presented as mean ± SD. *p < 0.05 versus corresponding control group; ^#^ *p* < 0.05 versus corresponding RANKL group; ns, no significance, versus corresponding control group.

Autophagy has been demonstrated to play critical roles in enhanced osteoclastogenesis under many conditions, such as hypoxia, oxidative stress, and microgravity (Wang et al., 2011; Sambandam et al., 2014; Sun et al., 2015). First, we assayed the level of autophagy after RANKL treatment. Western blot analysis showed that the LC3-II/LC3-I ratio was significantly upregulated from day 3 in a time-dependent manner during RANKL-induced osteoclastogenesis (Figure 1A). The TEM showed that the number of autophagic vacuoles was dramatically increased after 3 days of treatment with RANKL (Figure 1C). Then, the autophagic flux activity was further determined by using the adenovirus-mRFP-GFP-tagged LC3 system. The data showed that the number of yellow and red puncta in merged images was significantly increased after 3 days of RANKL-induced differentiation, indicating the activation of both autophagosome formation and lysosomal degradation in the RANKL-treated group (Figure 1D). Collectively, these observations indicate that autophagy is activated during RANKL-induced osteoclastogenesis. Second, using a pharmacological inhibitor of autophagy (CQ), we explored whether autophagy is essential in RANKL-induced osteoclastogenesis. The results showed that CQ treatment markedly increased the LC3-II/LC3-I ratio (Figure 1B) and inhibited autolysosomal degradation (Figures 1C,D). More importantly, we found that CQ treatment suppressed the RANKL-induced upregulation of osteoclastogenesis-related genes (TRAP, Cath K and MMP-9; Figure 1E), reduced the number of TRAP-positive multinuclear (≥3) osteoclasts and reduced the area of the bone resorption pits (Figure 1F). Taken together, the above data reveal that RANKL induces osteoclastogenesis and bone resorption through autophagy.

### Pharmacological Inhibition of Nox4 Suppresses RANKL-Induced Autophagy and Osteoclastogenesis

The above experiments showed that RANKL induced osteoclastogenesis and bone resorption through autophagy. It is well known that NADPH oxidase (Nox) family proteins promote the activation of autophagy in many cell types by generating ROS (Sciarretta et al., 2013). Recent evidence indicated that RANKL increases the generation of intracellular ROS by promoting the expression and activity of intracellular Nox family proteins during osteoclastogenesis (Lee et al., 2005; Goettsch et al., 2013; Kang and Kim, 2016). Therefore, we explored whether Nox family proteins are involved in RANKL-induced autophagy.

**FIGURE 2:**
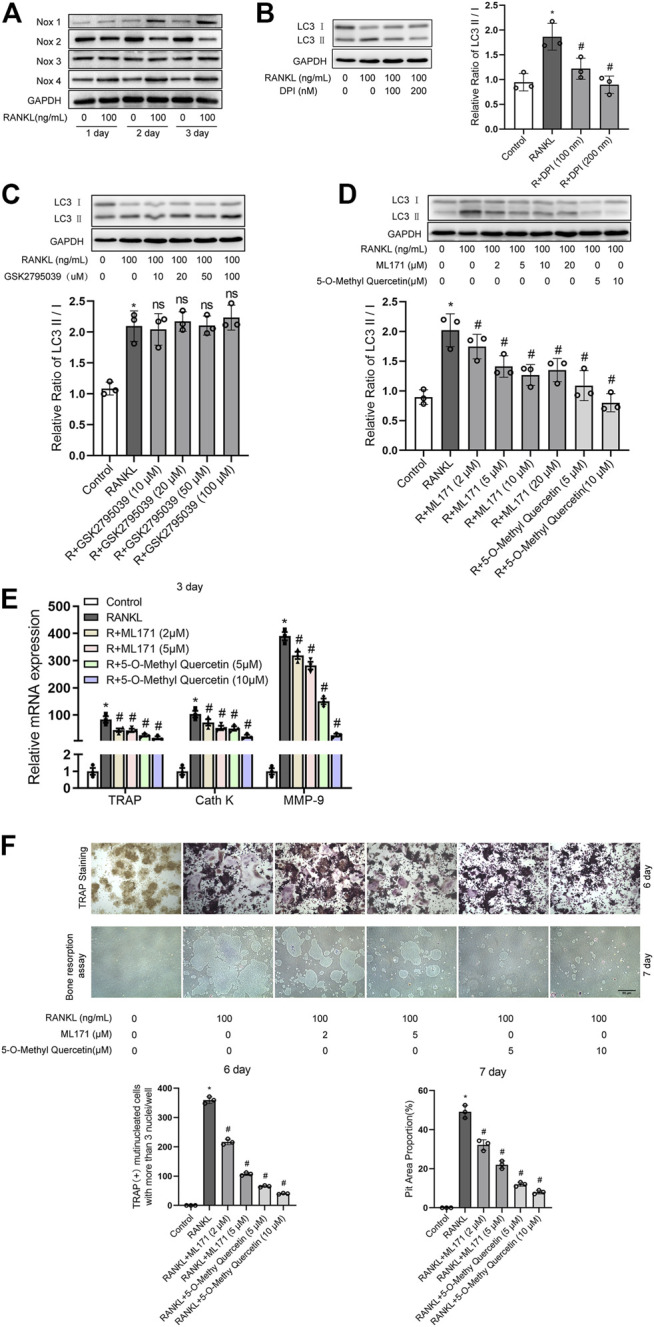
Drug inhibition of Nox4 suppresses RANKL-induced autophagy and osteoclastogenesis. **(A)** RAW264.7 cells were seeded overnight and incubated with or without RANKL (100 ng/ml) for the indicated times. The levels of Nox family proteins (Nox1, Nox2, Nox3, and Nox4) were assessed by western blot. **(B–D)** RAW264.7 cells were seeded overnight and treated with the Nox inhibitor DPI **(B)**, Nox2 inhibitor GSK2795039 **(C)**, Nox1 inhibitor ML171 and Nox4 inhibitor 5-O-methyl quercetin **(D)** in the presence of RANKL (100 ng/ml) for 3 days. The protein levels of LC3-I and LC3-II were tested by western blot, and the ratio of LC3-II/LC3-I was quantified by Image J. **(E)** RAW264.7 cells were seeded overnight and incubated with ML171 (2 and 5 μM) and 5-O-methyl quercetin (5 and 10 μM) in the presence of RANKL (100 ng/ml) for 3 days. The mRNA expression levels of TRAP, Cath K and MMP-9 were detected by qRT-PCR. **(F)** RAW264.7 cells were treated as described in **(E)** for the indicated times. Then, TRAP staining and bone resorption assays were performed to evaluate the formation of TRAP-positive multinucleated (≥3) osteoclasts and bone resorption pits respectively. All the data derived from at least three independent replicates and were presented as mean ± SD. *p < 0.05 versus corresponding control group; ^#^ *p* < 0.05 vs. corresponding RANKL group; ns, no significance, versus corresponding RANKL group.

Western blot analysis showed that RANKL time-dependently upregulated the levels of Nox1 and Nox4 proteins and decreased the levels of Nox2 protein but had no significant influence on the levels of Nox3 protein (Figure 2A). Nox pharmacological inhibitor DPI treatment obviously downregulated the RANKL-induced increase in the LC3-II/LC3-I ratio (Figure 2B). To further investigate which Nox isoforms are involved in RANKL-induced autophagy and osteoclastogenesis, pharmacological inhibitors targeting specific Nox isoforms were used. The data showed that the inhibition of Nox1 or Nox4 (not Nox2) separately by ML171 and 5-O-methyl quercetin significantly inhibited the RANKL-induced increase in the LC3-II/LC3-I ratio and osteoclastogenesis-related gene (TRAP, Cath K and MMP-9) expression and reduced the number of TRAP-positive multinuclear (≥3) osteoclasts and the area of the bone resorption pits (Figures 2C–F). Importantly, the inhibitory effect of 5-O-methyl quercetin on RANKL-induced autophagy, osteoclastogenesis and bone resorption was significantly greater than that of ML171. Therefore, compared with Nox1, Nox4 may play a leading role in autophagy activation induced by RANKL. We selected Nox4 for the subsequent experiments.

### Knockdown of Nox4 Suppresses RANKL-Induced Autophagy and Osteoclastogenesis

**FIGURE 3:**
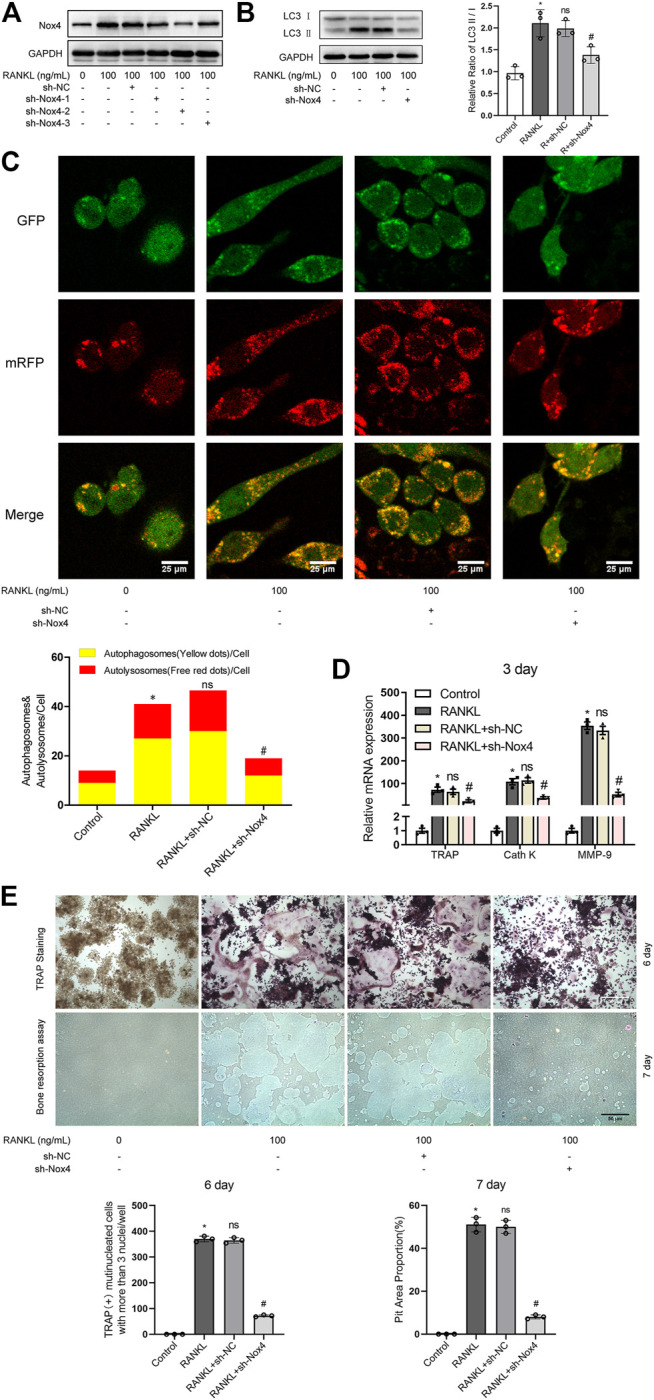
Nox4 knockdown suppresses RANKL-induced autophagy and osteoclastogenesis. **(A)** RAW264.7 cells were transfected with retrovirus encoding Nox4 shRNA (sh-Nox4) or scrambled shRNA (sh-NC). Then, the cells were seeded overnight and incubated with or without RANKL (100 ng/ml) for 3 days. The protein levels of Nox4 were assessed by western blot. **(B)** RAW264.7 cells were treated as described in **(A)**. Then, the protein levels of LC3-I and LC3-II were tested by western blot, and the ratio of LC3-II/LC3-I was quantified by Image J. **(C)** After sh-NC or sh-Nox4 transfection, RAW264.7 cells were transfected with Ad-mRFP-GFP-LC3 for 48 h. Then, the cells were seeded overnight and incubated with or without RANKL (100 ng/ml) for 3 days. Autophagic flux was assessed by quantifying the number of mRFP and GFP puncta per cell under a laser scanning confocal microscope. Representative images of mRFP and GFP puncta are shown, together with the quantification of autophagosomes and autolysosomes. **(D)** RAW264.7 cells were treated as described in **(A)**. Then, the mRNA expression levels of TRAP, Cath K, and MMP-9 were detected by qRT-PCR. **(E)** RAW264.7 cells were treated as described in **(A)** for the indicated times. Then, TRAP staining and bone resorption assays were performed to evaluate the formation of TRAP-positive multinucleated (≥3) osteoclasts and bone resorption pits respectively. All the data derived from at least three independent replicates and were presented as mean ± SD. *p < 0.05 versus corresponding control group; ^#^ *p* < 0.05 versus corresponding RANKL group; ns, no significance, versus corresponding RANKL group.

To further determine the functional significance of Nox4 in RANKL-induced autophagy and osteoclastogenesis, retroviruses encoding three different Nox4 shRNAs or scrambled shRNA were utilized. The results showed that the expression levels of Nox4 were dramatically decreased after the transfection of sh-Nox4-1, sh-Nox4-2, and sh-Nox4-3 (Figure 3A). Importantly, sh-Nox4-2 had the greatest Nox4 silencing effect. Therefore, sh-Nox4-2 was selected for the subsequent experiments. The knockdown of Nox4 markedly inhibited the RANKL-induced increase in the LC3-II/LC3-I ratio, autophagic flux activity, and expression of osteoclastogenesis-related genes (TRAP, Cath K and MMP-9) and decreased the number of TRAP-positive multinuclear (≥3) osteoclasts and bone resorption pit area (Figures 3B–E). Collectively, the above results indicate that knockdown of Nox4 suppresses RANKL-induced autophagy and osteoclastogenesis.

### RANKL Upregulates the Protein Level of Nox4 in the ER

**FIGURE 4:**
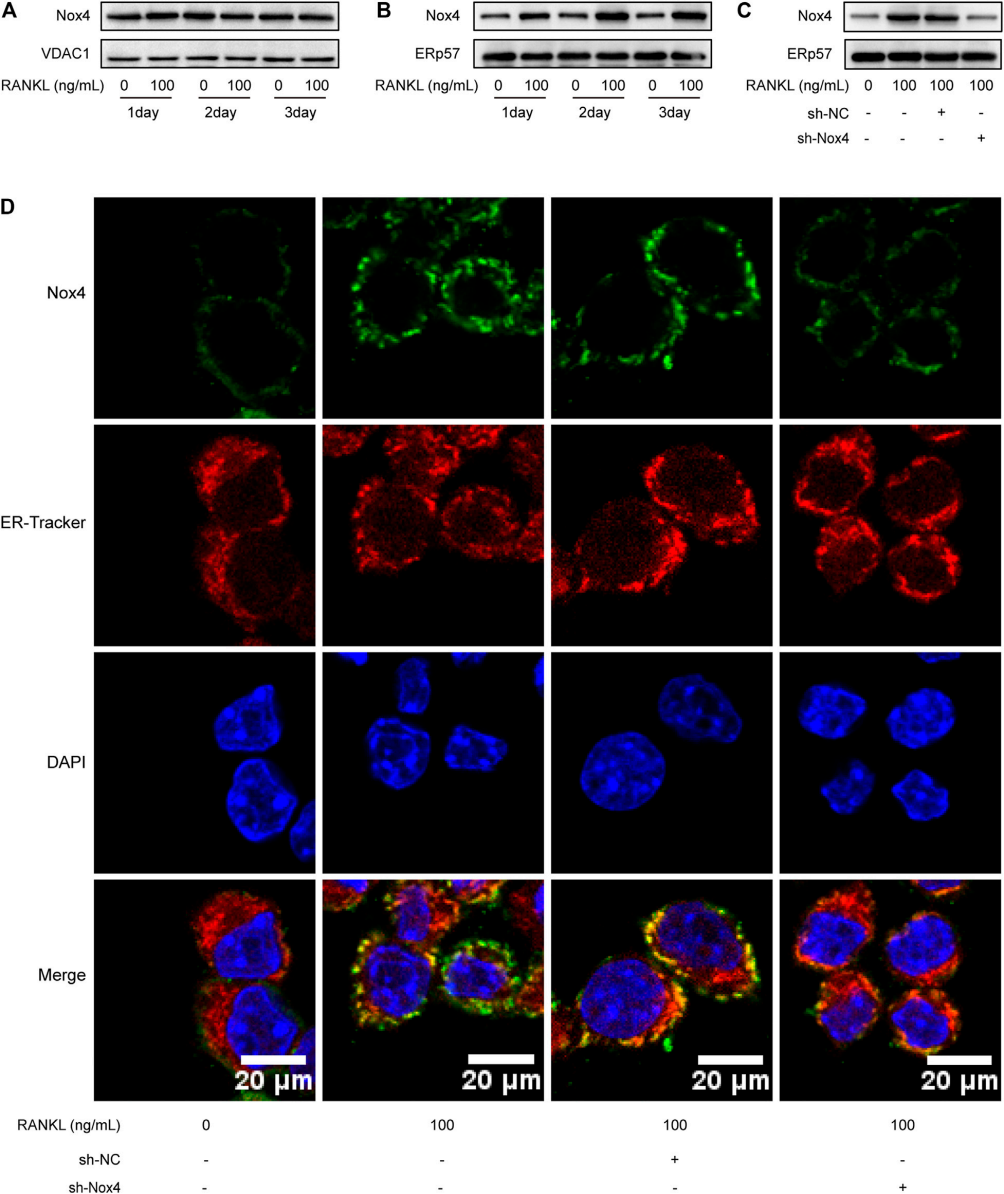
RANKL treatment specifically upregulates the level of Nox4 protein in the ER. **(A-B)** RAW264.7 cells were seeded overnight and incubated with or without RANKL (100 ng/ml) for the indicated times. The mitochondrial **(A)** and ER **(B)** fractions were isolated from cells. The protein levels of Nox4 in each fraction were assessed by western blot. VDAC1 and ERp57 were separately used as loading controls for mitochondria and the ER. **(C)** After sh-NC or sh-Nox4 transfection, RAW264.7 cells were seeded overnight and incubated with or without RANKL (100 ng/ml) for 3 days. The ER fraction was isolated from cells. The protein level of Nox4 in the ER was assessed by western blot. **(D)** RAW264.7 cells were treated as described in **(C)**. Then, the cells were stained with ER-Tracker. Immunofluorescence staining of Nox4 was performed to detect the colocalization of Nox4 with the ER.

Recent studies have indicated that Nox4 is localized on intracellular membranes, mainly in mitochondria and the ER (Lassegue et al., 2012). Western blot analysis showed that RANKL treatment markedly increased the protein level of Nox4 in the ER (not in the mitochondria) of RAW264.7 cells (Figures 4A,B). As shown in Figure 4C, Nox4 shRNA treatment significantly downregulated the RANKL-induced elevation of Nox4 protein in the ER of RAW264.7 cells. To further ascertain the ER localization of Nox4 protein induced by RANKL, an immunofluorescence staining assay was utilized. The results showed that RANKL treatment significantly enhanced the localization of Nox4 in the ER, which was markedly suppressed by Nox4 silencing (Figure 4D). Collectively, the above data reveal that RANKL markedly upregulates the protein level of Nox4 in the ER. These results are consistent with the fact that Nox4, mainly synthesized in the ER, contains multiple ER-specific signal sequences in the N-terminal portion of Nox4. (Chen et al., 2008; Stephens and Nicchitta, 2008).

### Nox4 Promotes RANKL-Induced Autophagy Activation and Osteoclastogenesis by Generating Nonmitochondrial ROS

**FIGURE 5:**
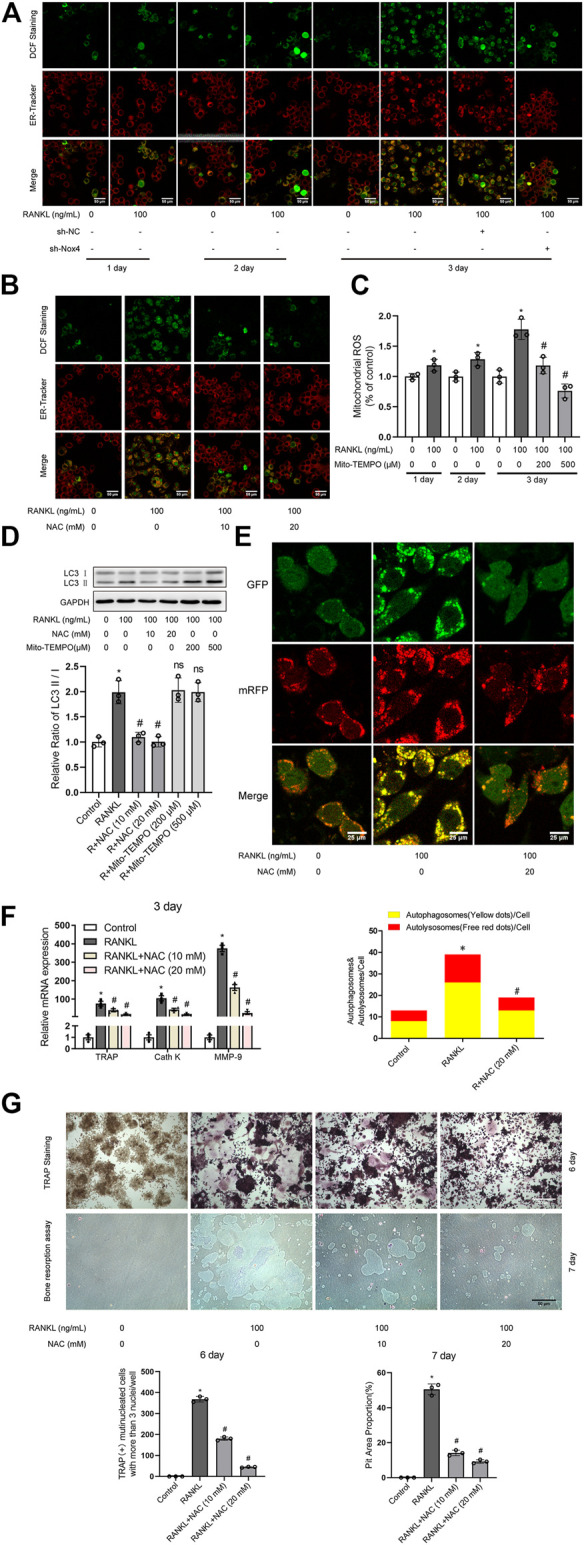
Nox4 promotes RANKL-induced autophagy and osteoclastogenesis via generating nonmitochondrial ROS. **(A)** After sh-NC or sh-Nox4 transfection, RAW264.7 cells were cultured with or without RANKL (100 ng/ml) for the indicated times. Then, the cells were stained with ER-Tracker. DCF fluorescence staining was performed to evaluate the level of intracellular ROS. **(B)** RAW264.7 cells were treated with the intracellular ROS scavenger NAC (10 and 20 mM) in the presence of RANKL (100 ng/ml) for 3 days. The intracellular ROS and ER ROS were measured as described in **(A)**. **(C)** RAW264.7 cells were treated with the mitochondrial-targeted antioxidant Mito-TEMPO (200 and 500 μM) in the presence of RANKL (100 ng/ml) for the indicated times. The level of mitochondrial ROS was measured by MitoSOX. **(D)** RAW264.7 cells were treated with NAC (10 and 20 mM) or Mito-TEMPO (200 and 500 μM) in the presence of RANKL (100 ng/ml) for 3 days. Protein levels of LC3-I and LC3-II were tested by western blot, and the ratio of LC3-II/LC3-I was quantified by Image J. **(E)** After transfection with Ad-mRFP-GFP-LC3 for 48 h, RAW264.7 cells were treated with NAC (20 mM) in the presence of RANKL (100 ng/ml) for 3 days. Then, autophagic flux was assessed by quantifying the number of mRFP and GFP puncta per cell under a laser scanning confocal microscope. Representative images of mRFP and GFP puncta are shown, together with the quantification of autophagosomes and autolysosomes. **(F)** RAW264.7 cells were treated as described in **(B)**. Then, the mRNA expression levels of TRAP, Cath K and MMP-9 were detected by qRT-PCR. **(G)** RAW264.7 cells were treated as described in **(B)** for the indicated times. TRAP-positive multinucleated (≥3) osteoclasts and bone resorption pits were evaluated by TRAP staining and bone resorption assays, respectively. All the data derived from at least three independent replicates and were presented as mean ± SD. *p < 0.05 versus corresponding control group; ^#^ *p* < 0.05 versus corresponding RANKL group; ns, no significance, versus corresponding RANKL group.

It has been reported that Nox family proteins can promote the production of intracellular ROS (Finkel, 2011). As shown in Figure 5A, RANKL treatment markedly enhanced the level of intracellular ROS and ER ROS in RAW264.7 cells, which was reduced by Nox4 silencing. As shown in Figure 5C, the level of mitochondrial ROS was increased in RAW264.7 cells during RANKL-induced osteoclastogenesis. As ROS have been reported to play an important role in autophagy regulation (Kongara and Karantza, 2012), we explored whether ROS are involved in RANKL-induced autophagy and osteoclastogenesis. Intracellular ROS scavenger (NAC) treatment significantly inhibited the RANKL-induced accumulation of intracellular ROS and ER ROS (Figure 5B). Mitochondrial-targeted antioxidant (Mito-TEMPO) treatment significantly inhibited RANKL-induced mitochondrial ROS accumulation (Figure 5C). Importantly, Mito-TEMPO treatment did not affect the RANKL-induced increase in the LC3-II/LC3-I ratio (Figure 5D), whereas NAC treatment obviously reduced the RANKL-induced increase in the LC3-II/LC3-I ratio (Figure 5D) and the number of yellow and red puncta in merged images, which implies an impairment in autophagic flux activity (Figure 5E). Additionally, NAC treatment also significantly downregulated the RANKL-induced upregulation of the expression of osteoclastogenesis-related genes (TRAP, Cath K and MMP-9; Figure 5F) and reduced the number of TRAP- positive multinuclear (≥3) osteoclasts and bone resorption pit area (Figure 5G). In summary, these data indicate that Nox4 promotes RANKL-induced autophagy activation and osteoclastogenesis by generating nonmitochondrial ROS. Furthermore, we also found that the majority of Nox4-derived ROS colocalize with ER-Tracker (Figure 5A). These results suggest that Nox4 may promote the activation of autophagy via the generation of ER-derived ROS during RANKL-induced osteoclastogenesis.

### Nox4-Derived ROS Promotes Autophagy via the PERK/eIF-2α/ATF4 Pathway

**FIGURE 6:**
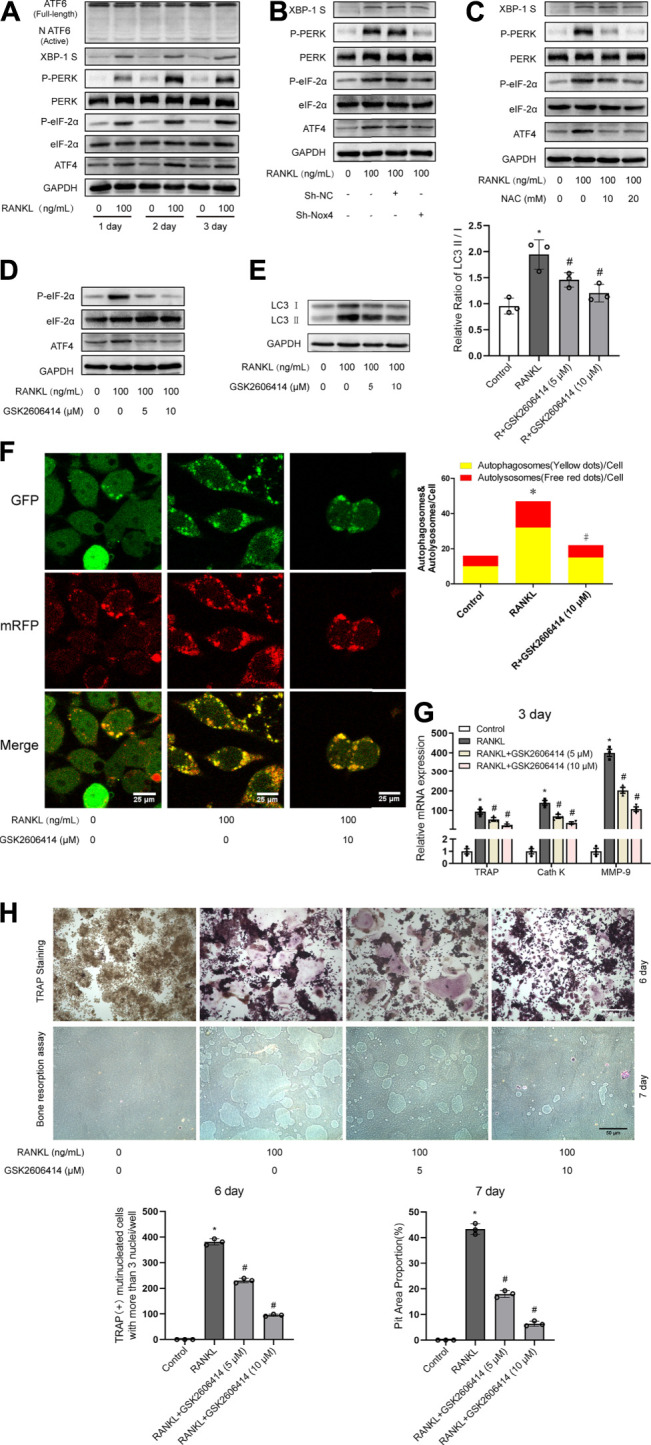
Nox4-derived ROS promotes autophagy via the PERK/eIF-2α/ATF4 pathway. **(A)** RAW264.7 cells cultured with or without RANKL (100 ng/ml) for the indicated times. The protein levels of ATF6, XBP-1S, phospho-PERK (Thr980), PERK, phospho-eIF-2α (Ser51), eIF-2α, and ATF4 were evaluated by western blot. **(B)** After sh-NC or sh-Nox4 transfection, RAW264.7 cells were incubated with or without RANKL (100 ng/ml) for 3 days. The proteins mentioned in **(A)** were assessed by western blot. **(C)** RAW264.7 cells were treated with NAC (10 and 20 mM) and RANKL (100 ng/ml) for 3 days. The proteins mentioned in **(A)** were assessed by western blot. **(D)** RAW264.7 cells were incubated with PERK inhibitor GSK2606414 (5 and 10 μM) in the presence of RANKL (100 ng/ml) for 3 days. The protein levels of phospho-eIF-2α (Ser51), eIF-2α, and ATF4 were evaluated by western blot. **(E)** RAW264.7 cells were treated as described in **(D)**. Then, the protein levels of LC3-I and LC3-II were tested by western blot, and the ratio of LC3-II/LC3-I was quantified by Image J. **(F)** After transfection with Ad-mRFP-GFP-LC3 for 48 h, RAW264.7 cells were treated with GSK2606414 (10 μM) in the presence of RANKL (100 ng/ml) for 3 days. Autophagic flux was assessed by quantifying the number of mRFP and GFP puncta per cell under a laser scanning confocal microscope. Representative images of mRFP and GFP puncta are shown, together with the quantification of autophagosomes and autolysosomes. **(G)** RAW264.7 cells were treated as described in **(D)**. Then, the mRNA expression levels of TRAP, Cath K and MMP-9 were detected by qRT-PCR. **(H)** RAW264.7 cells were treated as described in **(D)** for the indicated times. TRAP-positive multinucleated (≥3) osteoclasts and bone resorption pits were evaluated by TRAP staining and bone resorption assays, respectively. All the data derived from at least three independent replicates and were presented as mean ± SD. *p < 0.05 versus corresponding control group; ^#^ *p* < 0.05 vs. corresponding RANKL group.

Previous studies have demonstrated that UPR-related signaling pathways (ATF6, PERK/eIF-2α/ATF4, and IRE-1α/XBP-1) are involved in ROS-induced autophagy (Avivar-Valderas et al., 2011; Walter and Ron, 2011). Therefore, we further explored whether UPR-related signaling pathways mediate ROS-induced autophagy during RANKL-induced osteoclastogenesis. Western blot analysis showed that RANKL treatment time-dependently increased the phosphorylation of PERK (Thr980) and eIF-2α (Ser51) and upregulated the expression levels of ATF4 and XBP-1S in RAW264.7 cells but had little influence on the level of ATF6 (Figure 6A). Moreover, knockdown of Nox4 significantly repressed the RANKL-induced phosphorylation of PERK (Thr980) and eIF-2α (Ser51) and the upregulation of ATF4 expression but had little influence on XBP-1S (Figure 6B). Furthermore, NAC treatment significantly inhibited the RANKL-induced activation of the PERK/eIF-2α/ATF4 pathway (Figure 6C). These results reveal that RANKL activates the PERK/eIF-2α/ATF4 pathway by elevating Nox4-mediated ROS production in RAW264.7 cells. To further confirm the involvement of the PERK/eIF-2α/ATF4 pathway in the activation of autophagy, osteoclastogenesis and bone resorption, a pharmacological inhibitor of PERK (GSK2606414) was utilized. The data revealed that GSK2606414 treatment dose-dependently reduced the RANKL-induced phosphorylation of eIF-2α (Ser51) and the upregulation of ATF4 expression in RAW264.7 cells (Figure 6D). More importantly, GSK2606414 treatment obviously inhibited the RANKL-induced increase in the LC3-II/LC3-I ratio (Figure 6E) and the number of yellow and red puncta in merged images, which implies that autophagic flux is impaired (Figure 6F). Additionally, GSK2606414 treatment also significantly abrogated the RANKL-induced upregulation of osteoclastogenesis-related genes (TRAP, Cath K and MMP-9; Figure 6G) and reduced the number of TRAP-positive multinuclear (≥3) osteoclasts and bone resorption pit area (Figure 6H). These results indicate that Nox4-derived ROS promote autophagy via the PERK/eIF-2α/ATF4 pathway during RANKL-induced osteoclastogenesis.

## Discussion

**FIGURE 7:**
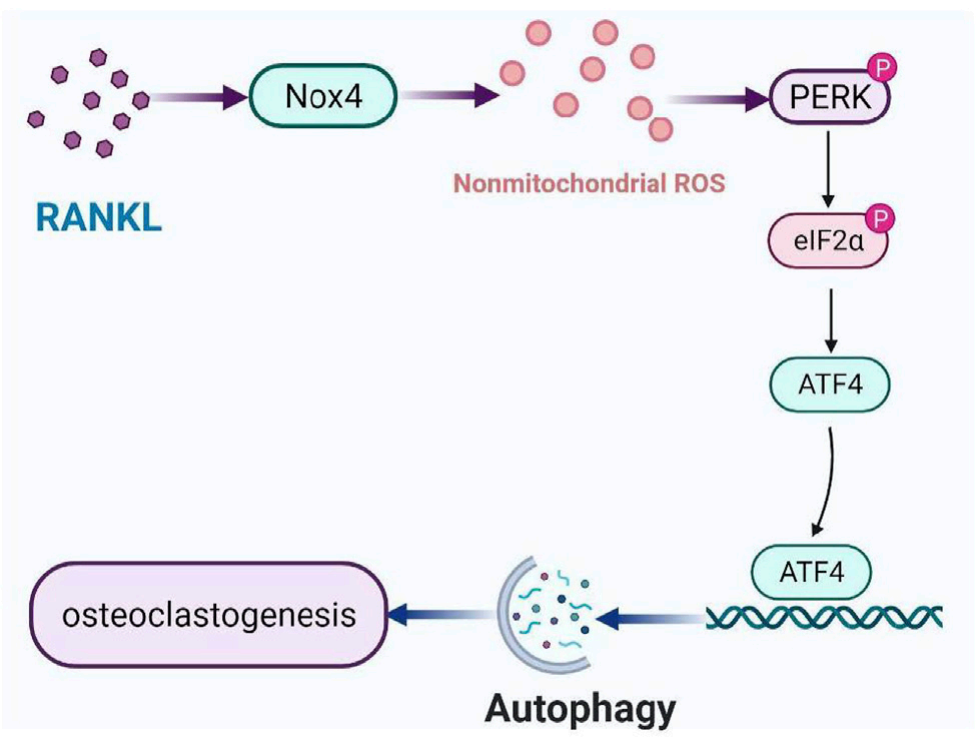
Schematic model of the mechanism of RANKL-induced autophagy and osteoclastogenesis. RANKL upregulates the protein level of Nox4, enhancing the level of nonmitochondrial ROS, activating PERK/eIF-2α/ATF4 pathway, leading to autophagy and osteoclastogenesis.

Autophagy, an evolutionarily conserved and dynamic catabolic process, plays a critical role in maintaining bone homeostasis (Shapiro et al., 2014). Recently, it has been demonstrated that autophagy plays an important role in osteoclastogenesis. However, it is still unclear that the molecular mechanism of RANKL-induced autophagy in osteoclastogenesis. In the present study, we identified a new mechanism of autophagy regulation during RANKL-induced osteoclastogenesis. Specifically, Nox4 promotes RANKL-induced autophagy activation and osteoclastogenesis by increasing the level of nonmitochondrial ROS and subsequent activation of the PERK/eIF-2α/ATF4 signaling pathway (Figure 7). Our results provide a new insight into the molecular mechanisms of RANKL-induced osteoclastogenesis and may help the development of new therapeutic strategies for osteoclastogenesis-related diseases.

Nox, which is widely distributed in various tissues and organs, is the key enzyme of redox signaling (Kleniewska et al., 2012). The Nox family includes seven members as Nox1-5 and Duox1-2 (Youn et al., 2013). The protein levels of Nox1 and Nox4 are increased, the protein level of Nox2 is decreased, and the protein level of Nox3 remains unchanged during RANKL-induced osteoclastogenesis (Sasaki et al., 2009; Goettsch et al., 2013). Compared with the other isoforms, Nox1 plays more prominent roles in stimulating RANKL-induced osteoclastogenesis (Lee et al., 2005). In our study, we found that RANKL treatment caused similar Nox protein expression patterns. However, we found that Nox4 plays a more critical role in regulating RANKL-induced autophagy activation than other Nox isoforms, including Nox1. This specific role of Nox4 in regulating autophagy may be dependent upon its intracellular localization. Nox4 is localized to intracellular membranes, particularly in the ER and mitochondria, while Nox1 is mainly located on the plasma membrane. (Lassegue et al., 2012). ER-localized Nox4 has been found to promote the proliferation, migration, differentiation, and survival of cells (Ago et al., 2010; Auer et al., 2017). The activity of ER-localized Nox4 in the regulation of cellular processes may be dependent upon its ability to produce H_2_O_2_ (Wu et al., 2010). Here, we observed for the first time that the level of ER-localized (not mitochondria-localized) Nox4 was dramatically increased during RANKL-induced osteoclastogenesis. This result may be explained by the fact that Nox4 is a membrane-bound protein and, therefore, is mainly translated in the ER through a cotranslational translocation mechanism (Stephens and Nicchitta, 2008). Recent study indicates that alternative splicing of *Nox4* mRNA may trigger Nox4 synthesis in different subcellular compartments (Anilkumar et al., 2013). It is reasonable to speculate that posttranslational modifications of nascent Nox4 protein or other unknown mechanisms may be associated with this selective elevation of Nox4 in the ER of RAW264.7 cells cultured under RANKL induction conditions (Colombo et al., 2005). However, whether the specific sub-cellullar location of Nox4 lead to RANKL-induced autophagy activation needs to be further studied.

ROS is a class of oxygen-containing compounds with oxidative activity that are produced during aerobic metabolism, including hydrogen peroxide (H_2_O_2_), superoxide anions (O2^−^) and free radicals (HO•) (Kongara and Karantza, 2012). In recent years, studies have shown that ROS can be used as a signal molecule to participate in the transduction of a variety of intracellular signals, and it plays an key role in various pathological and physiological processes such as immune response, autophagy, cellular stress, and inflammation (Wolf, 2005; Xia et al., 2007; Scherz-Shouval and Elazar, 2011; Lee et al., 2012). Nox proteins are considered the most important source of ROS from different parts of the cell, including mitochondria (Canugovi et al., 2019), the ER (Sciarretta et al., 2013) and the cytomembrane (Wang et al., 2011). Mitochondria, as the sites of oxidative respiration in cells, are the main production sites of intracellular ROS (Kim et al., 2020). Previously, we and others have found that RANKL treatment increases mitochondrial ROS in osteoclast precursors (Srinivasan et al., 2010); however, mitochondrial-targeted antioxidants (Mito-TEMPO) do not block RANKL-induced autophagy activation. These results indicate that Nox4 promotes the activation of autophagy by generating nonmitochondrial ROS during RANKL-induced osteoclastogenesis. Furthermore, we also found that the majority of Nox4-derived ROS was colocalized with ER-Tracker, which suggests that Nox4 may promote the activation of autophagy via the generation of ER-derived ROS during RANKL-induced osteoclastogenesis. Previous studies have demonstrated that UPR-related signaling pathways (ATF6, PERK/eIF-2α/ATF4, and IRE-1α/XBP-1) are involved in ER-derived ROS-induced autophagy (Sciarretta et al., 2013). Therefore, we further explored whether UPR-related signaling pathways mediate ROS-induced autophagy during RANKL-induced osteoclastogenesis. Western blot analysis showed that RANKL treatment time-dependently increased the phosphorylation of PERK (Thr980) and eIF-2α (Ser51) and upregulated the expression levels of ATF4 and XBP-1S in RAW264.7 cells but had little influence on the level of ATF6 (Figure 6A). Moreover, knockdown of Nox4 or inhibition of ROS by NAC significantly repressed the RANKL-induced phosphorylation of PERK (Thr980) and eIF-2α (Ser51) and the upregulation of ATF4 expression but had little influence on XBP-1S (Figures 6B,C). The above results suggest that Nox4 elevates ER-derived ROS, activating PERK/eIF-2α/ATF4 pathway, leading to autophagy during RANKL-induced osteoclastogenesis. Our finding is supported by the findings of a recent study in which an increase in Nox4-dependent ROS accumulation in the ER of cardiomyocytes was found to promote the activation of autophagy and survival during energy deprivation (Sciarretta et al., 2013).

A variety of osteolytic diseases including postmenopausal osteoporosis, Paget disease of bone and inflammatory arthritis are strongly correlated with the excessive differentiation of osteoclasts/osteoclastogenesis (Chung and Van Hul, 2012; Johnson et al., 2015). Our study is focus on the molecular mechanism of RANKL-induced osteoclastogenesis and we found that RANKL upregulates the protein level of Nox4, enhancing the level of ROS, activating PERK/eIF-2α/ATF4 pathway, leading to autophagy and osteoclastogenesis. Targeting the novel pathway “Nox4/ROS/PERK/eIF-2α/ATF4/autophagy” may facilitate the development of novel therapeutic strategies for osteoclastogenesis-related diseases. Currently, there are several molecular drugs have been been successfully used clinically to prevent and treat osteolytic disease. For example, Bazedoxifene, agonist of estrogen receptor α, was used to treat postmenopausal osteoporosis via activating estrogen receptor α on osteoblast and osteocyte and increasing the ratio of OPG/RANKL to inhibit the activity of osteoclast (Brown, 2017). Moreover, denosumab, a monoclonal antibody against RANKL, has been widely used in the clinical treatment of osteolytic disease. Denosumab exerts its pharmacology function by direct binding to RANKL and inhibiting osteoclast differentiation (Dahiya et al., 2015). In addition, previous study revealed that chloroquine, inhibitor of autophagy, ameliorated glucocorticoid-induced and ovariectomy-induced bone loss by inhibiting osteoclastogenesis in preclinical models (Lin et al., 2016), suggesting that inhibitors of autophagy such as CQ are already in clinical use may be used to treat for activated osteoclastogenesis disease. Importantly, GSK137831, an inhibitor of Nox1/4, has been passed through Phase 1 clinical trials (NCT02010242) for treatment of Type 2 Diabetes and Albuminuria, suggesting that GSK137831 may be used in the clinical treatment of activated osteoclastogenesis disease.

In conclusion, we identified a novel role and mechanism of Nox4 in regulating autophagy during RANKL-induced osteoclastogenesis. Specifically, Nox4 promotes RANKL-induced autophagy by increasing the level of nonmitochondrial ROS and activating the UPR-related signaling pathway (PERK/eIF-2α/ATF4). These findings provide a new insight into the processes of RANKL-induced osteoclastogenesis, which may help the development of new potential therapeutic strategies for osteoclastogenesis-related diseases.

## Conclusion

In the present study, we found that RANKL induced osteoclastogenesis via autophagy. The investigation for the corresponding mechanism revealed that RANKL promoted autophagy via upregulating the protein level of NADPH oxidase 4 (Nox4). Additionally, we found that Nox4 stimulated the production of nonmitochondrial reactive oxygen species (ROS), activating the critical unfolded protein response (UPR)-related signaling pathway PERK/eIF-2α/ATF4, leading to RANKL-induced autophagy and osteoclastogenesis. Collectively, this study reveals that Nox4 promotes RANKL-induced autophagy and osteoclastogenesis via activating ROS/PERK/eIF-2α/ATF4 pathway, suggesting that the pathway may be a novel potential therapeutic target for osteoclastogenesis-related disease.

## Data Availability

The original contributions presented in the study are included in the article/Supplementary Materials, further inquiries can be directed to the corresponding authors.

## Supplementary Material

Supplementary Figure S1 RANKL induces osteoclastogenesis and bone resorption. **(A,B)** RAW264.7 cells were seeded overnight and incubated with or without RANKL (100 ng/mL) for 3 days. Subsequently, the fused multinuclear osteoclasts (indicated by arrows) were observed under a light microscope **(A)**, and the mRNA expression levels of TRAP, Cath K and MMP-9 were detected by qRT-PCR **(B)**. **(C)** RAW264.7 cells were seeded overnight and incubated with or without RANKL (100 ng/mL) for the indicated times. Then, TRAP staining and bone resorption assays were performed to evaluate the formation of TRAP-positive multinucleated ( ≥ 3) osteoclasts and bone resorption pits respectively. All the data derived from at least three independent replicates and were presented as mean ± SD.
