# Supplementary figures and images for: Nox4 Promotes RANKL-Induced Autophagy and Osteoclastogenesis via Activating ROS/PERK/eIF-2α/ATF4 Pathway (part 1 of 3)
Source: Front Pharmacol. 2021 Sep 28;12:751845. doi: 10.3389/fphar.2021.751845 (PMC8505706; doi:10.3389/fphar.2021.751845)

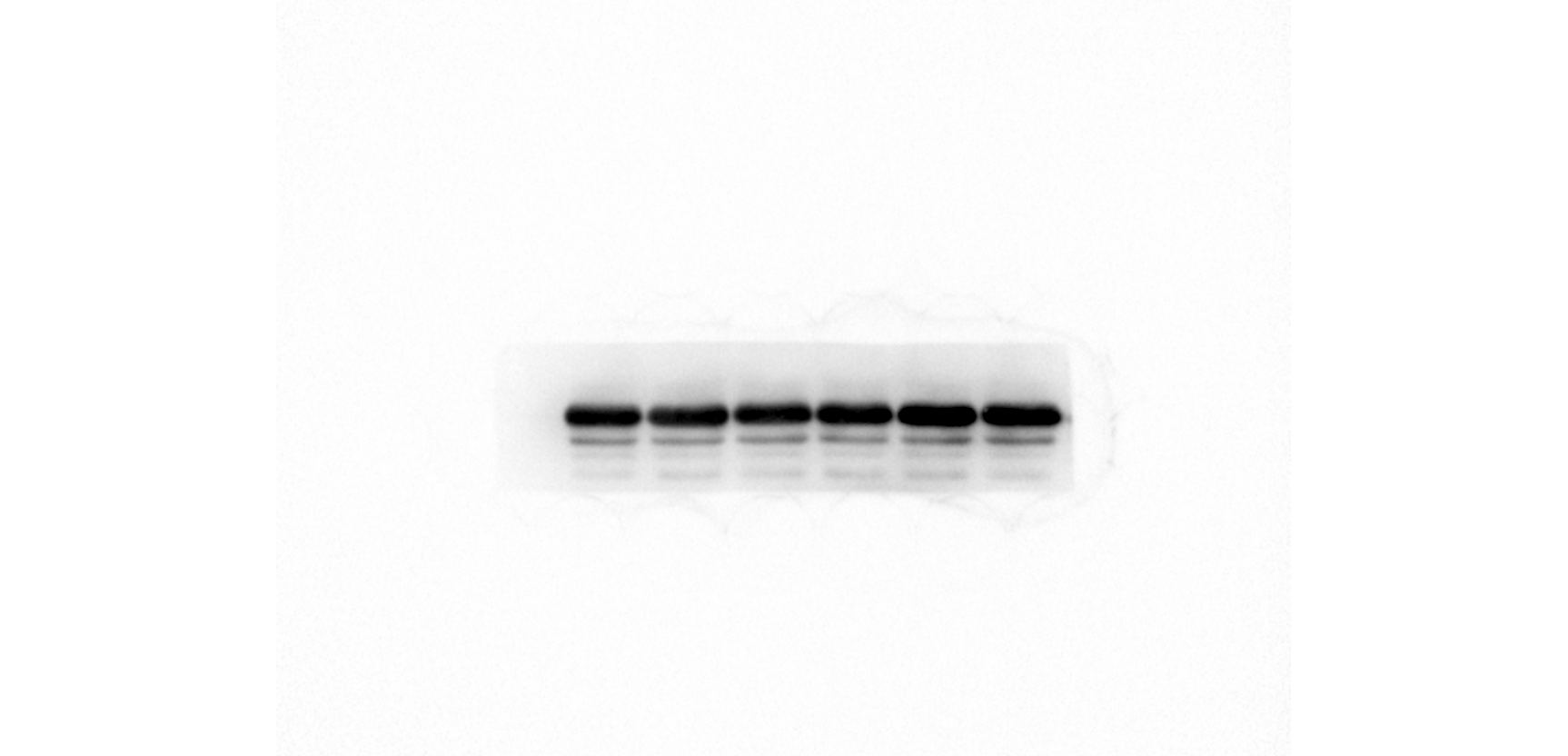

Supplement: Supplementary file 1 [file DataSheet3.ZIP › Fig.3-Source data/A/GAPDH.tif]

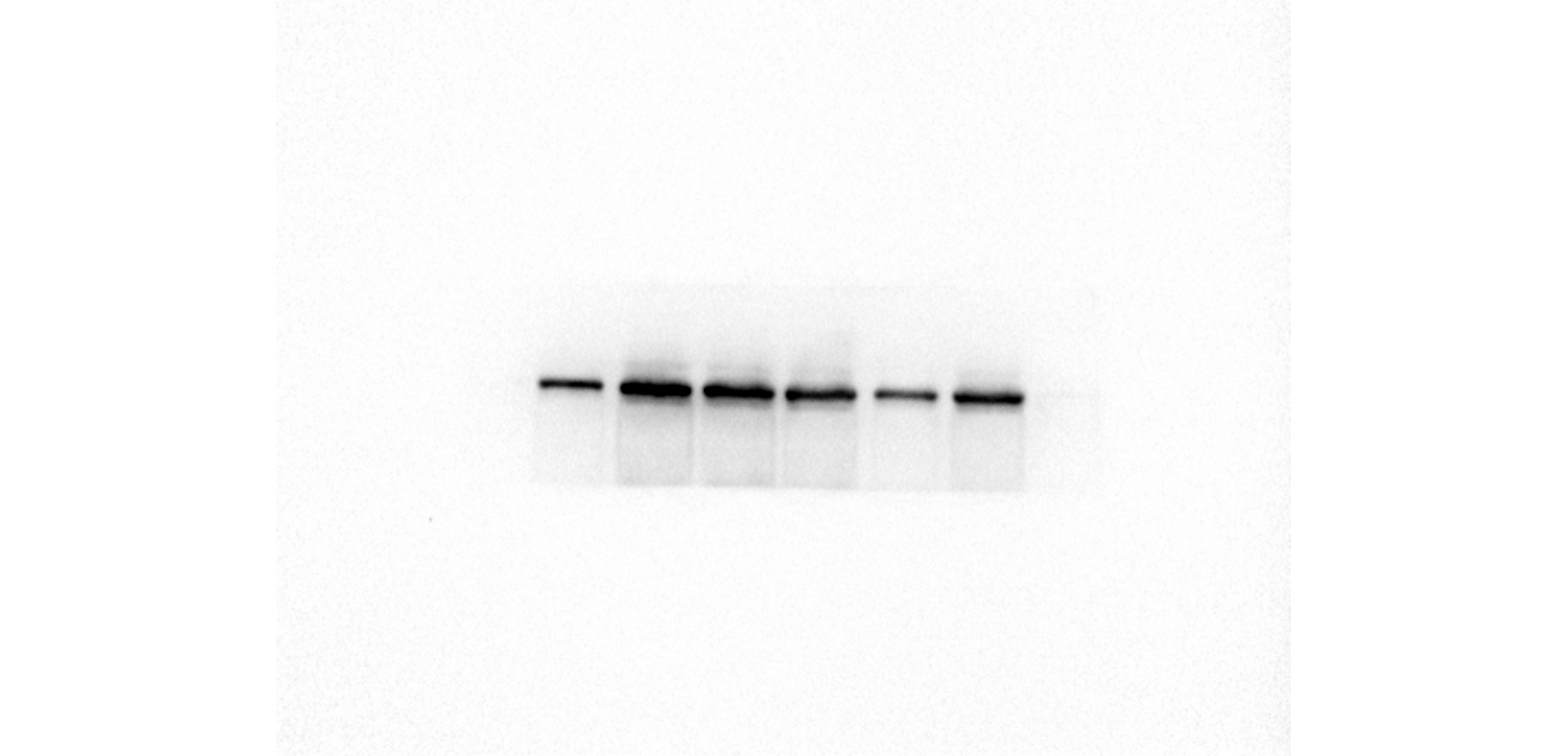

Supplement: Supplementary file 1 [file DataSheet3.ZIP › Fig.3-Source data/A/Nox4.tif]

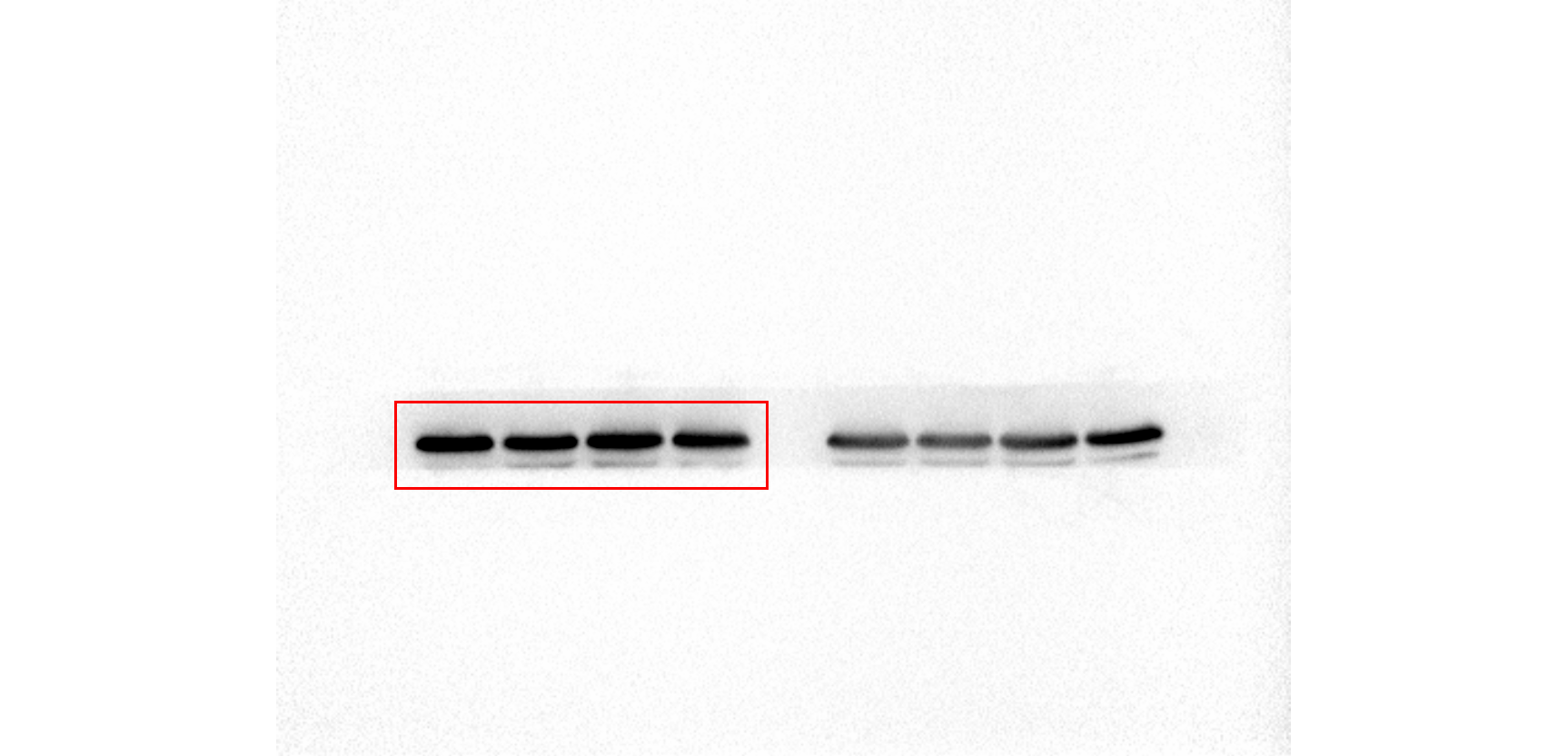

Supplement: Supplementary file 1 [file DataSheet3.ZIP › Fig.3-Source data/B/GAPDH.tif]

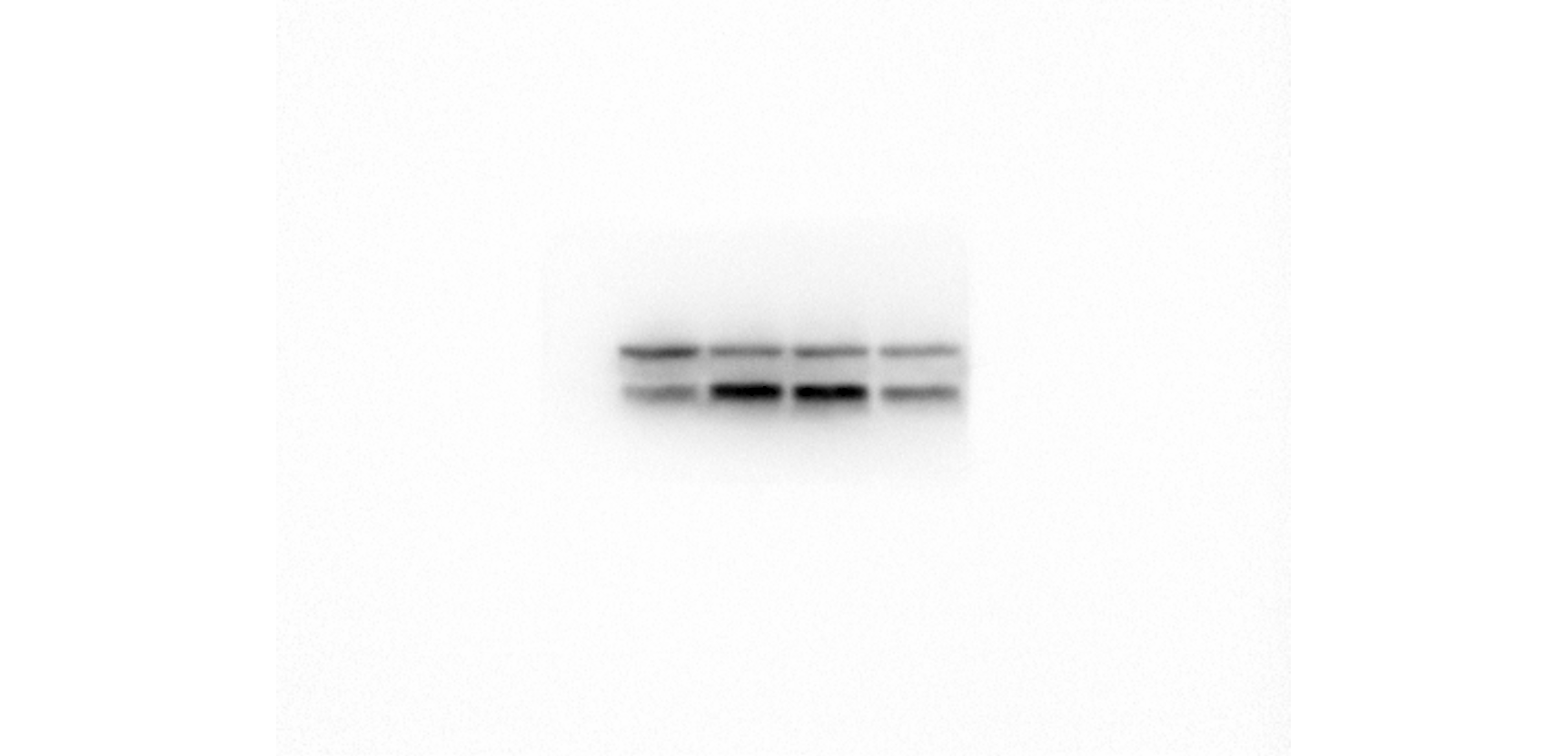

Supplement: Supplementary file 1 [file DataSheet3.ZIP › Fig.3-Source data/B/LC3.tif]

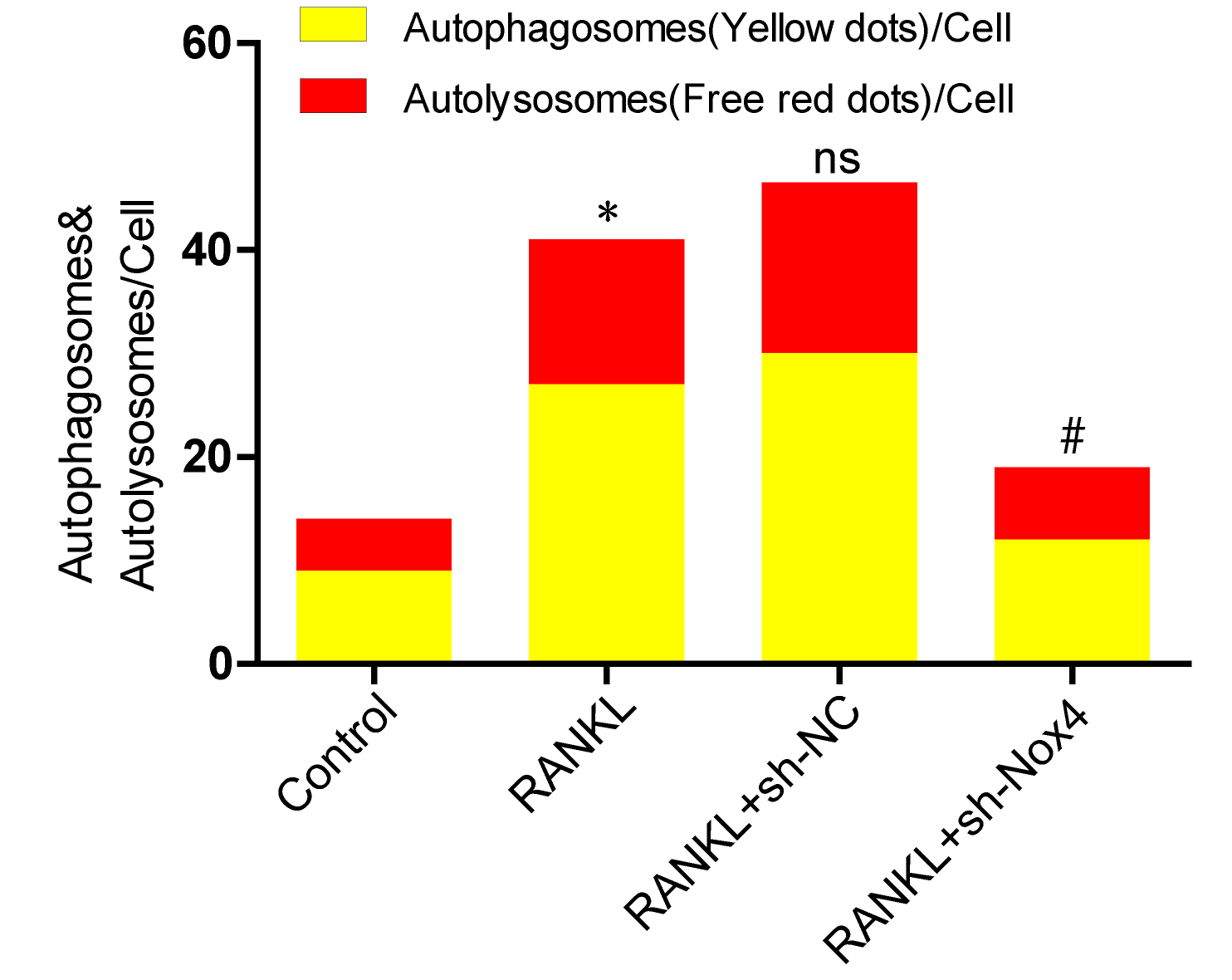

Supplement: Supplementary file 1 [file DataSheet3.ZIP › Fig.3-Source data/C/Fig.3C.tif]

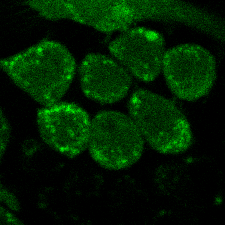

Supplement: Supplementary file 1 [file DataSheet3.ZIP › Fig.3-Source data/C/RANKL+sh-NC-GFP.tif]

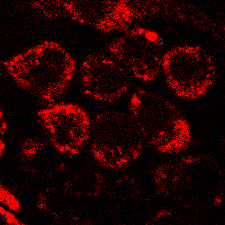

Supplement: Supplementary file 1 [file DataSheet3.ZIP › Fig.3-Source data/C/RANKL+sh-NC-mRFP.tif]

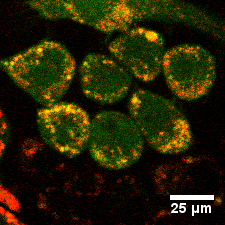

Supplement: Supplementary file 1 [file DataSheet3.ZIP › Fig.3-Source data/C/RANKL+sh-NC-merged.tif]

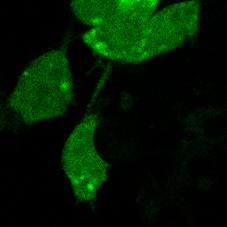

Supplement: Supplementary file 1 [file DataSheet3.ZIP › Fig.3-Source data/C/RANKL+sh-Nox4-GFP.tif]

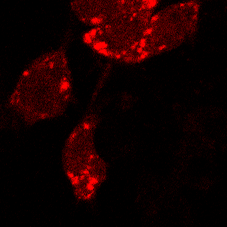

Supplement: Supplementary file 1 [file DataSheet3.ZIP › Fig.3-Source data/C/RANKL+sh-Nox4-mRFP.tif]

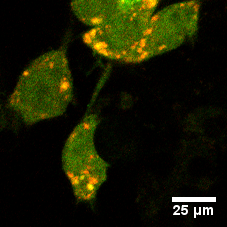

Supplement: Supplementary file 1 [file DataSheet3.ZIP › Fig.3-Source data/C/RANKL+sh-Nox4-merged.tif]

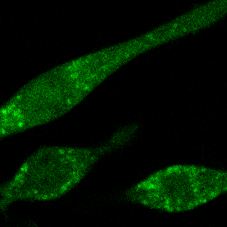

Supplement: Supplementary file 1 [file DataSheet3.ZIP › Fig.3-Source data/C/RANKL-GFP.tif]

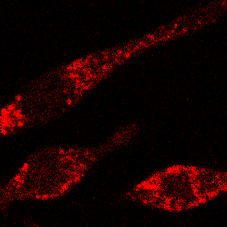

Supplement: Supplementary file 1 [file DataSheet3.ZIP › Fig.3-Source data/C/RANKL-mRFP.tif]

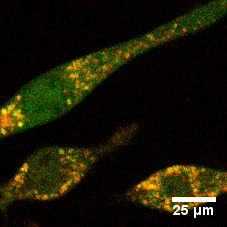

Supplement: Supplementary file 1 [file DataSheet3.ZIP › Fig.3-Source data/C/RANKL-merged.tif]

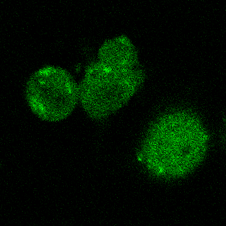

Supplement: Supplementary file 1 [file DataSheet3.ZIP › Fig.3-Source data/C/control-GFP.tif]

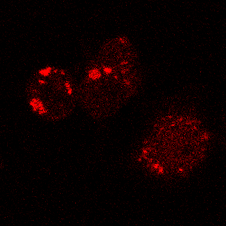

Supplement: Supplementary file 1 [file DataSheet3.ZIP › Fig.3-Source data/C/control-mRFP.tif]

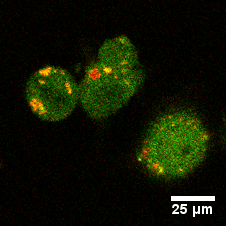

Supplement: Supplementary file 1 [file DataSheet3.ZIP › Fig.3-Source data/C/control-merged.tif]

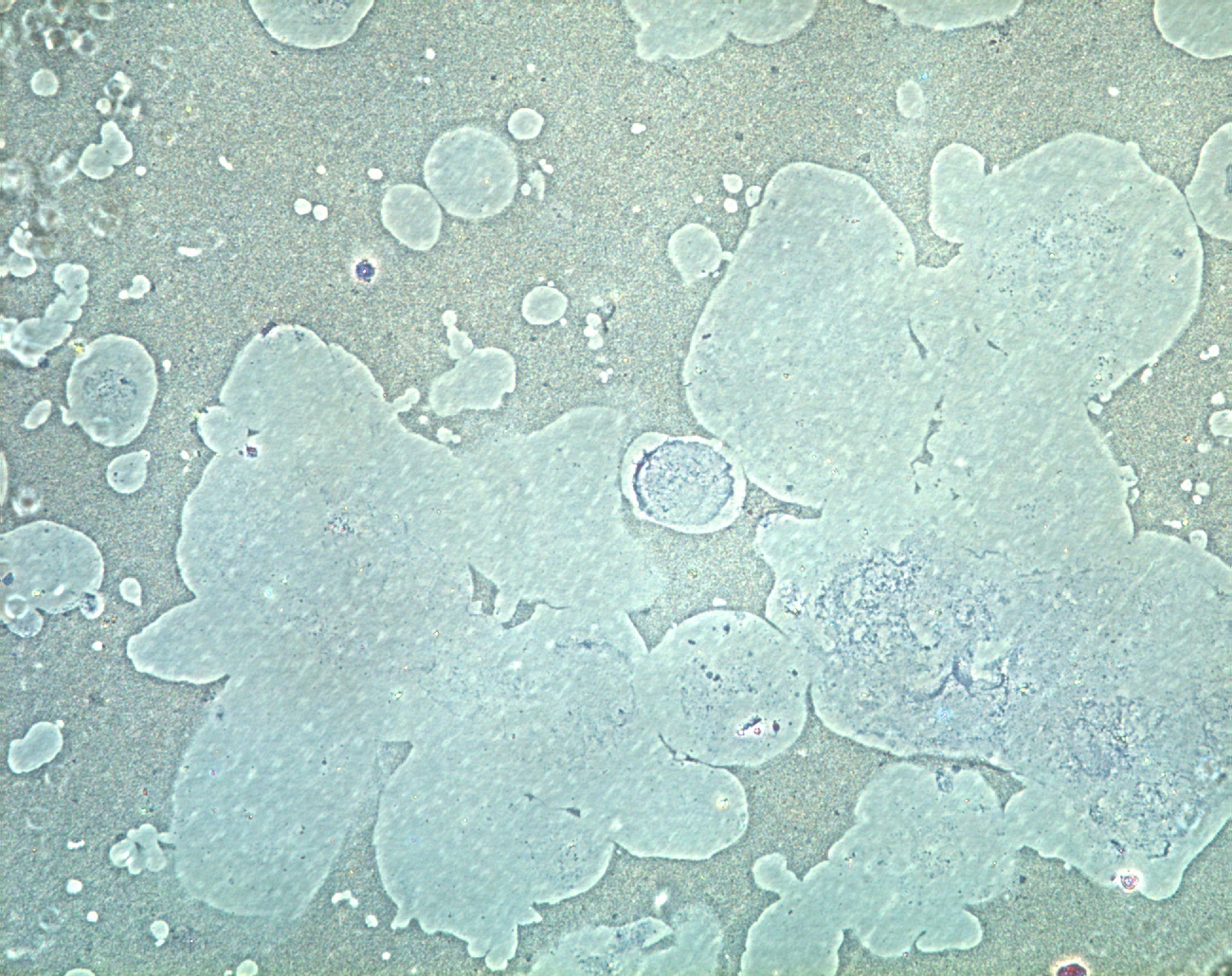

Supplement: Supplementary file 1 [file DataSheet3.ZIP › Fig.3-Source data/E/RANKL+sh-NC.jpg]

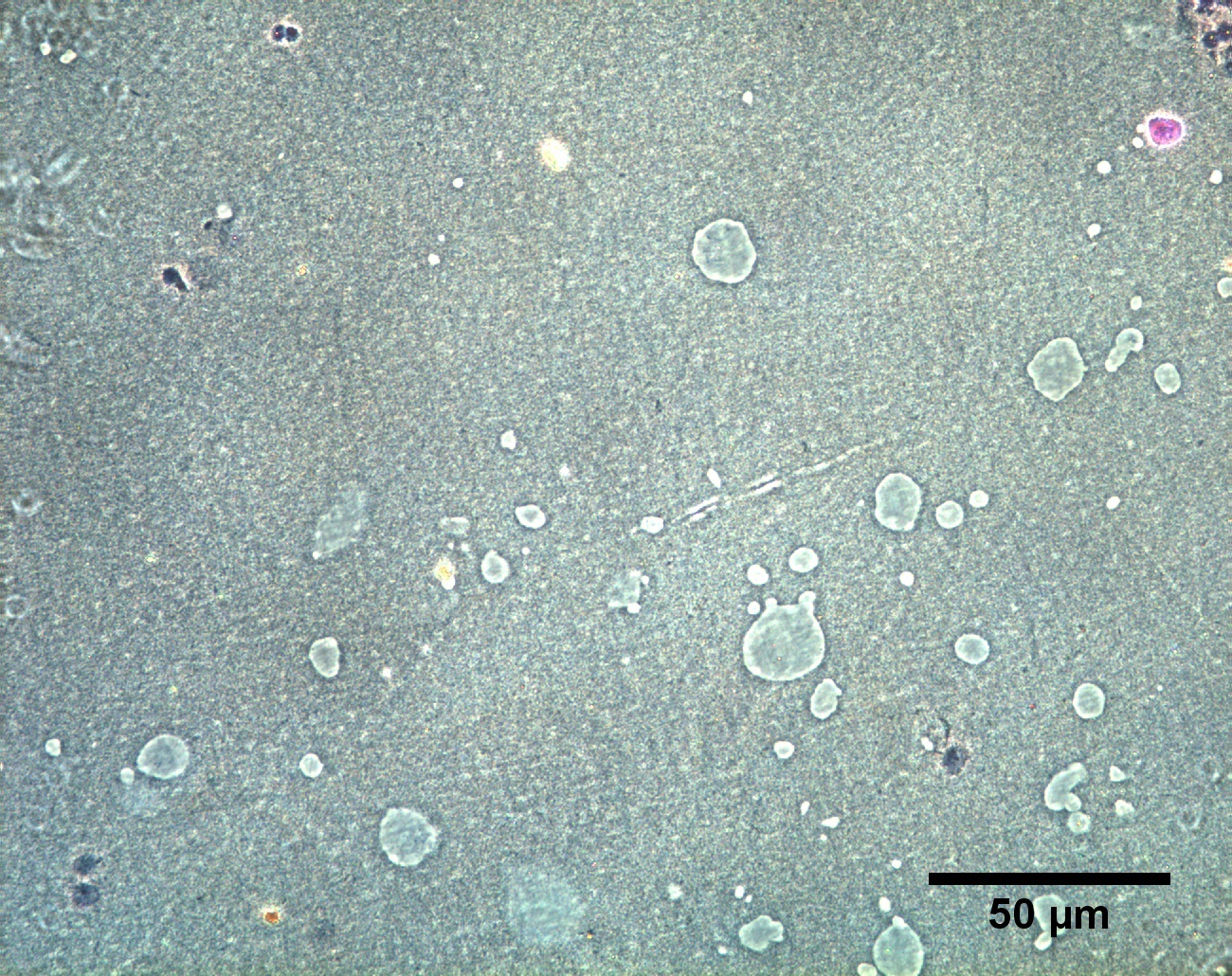

Supplement: Supplementary file 1 [file DataSheet3.ZIP › Fig.3-Source data/E/RANKL+sh-Nox4.jpg]

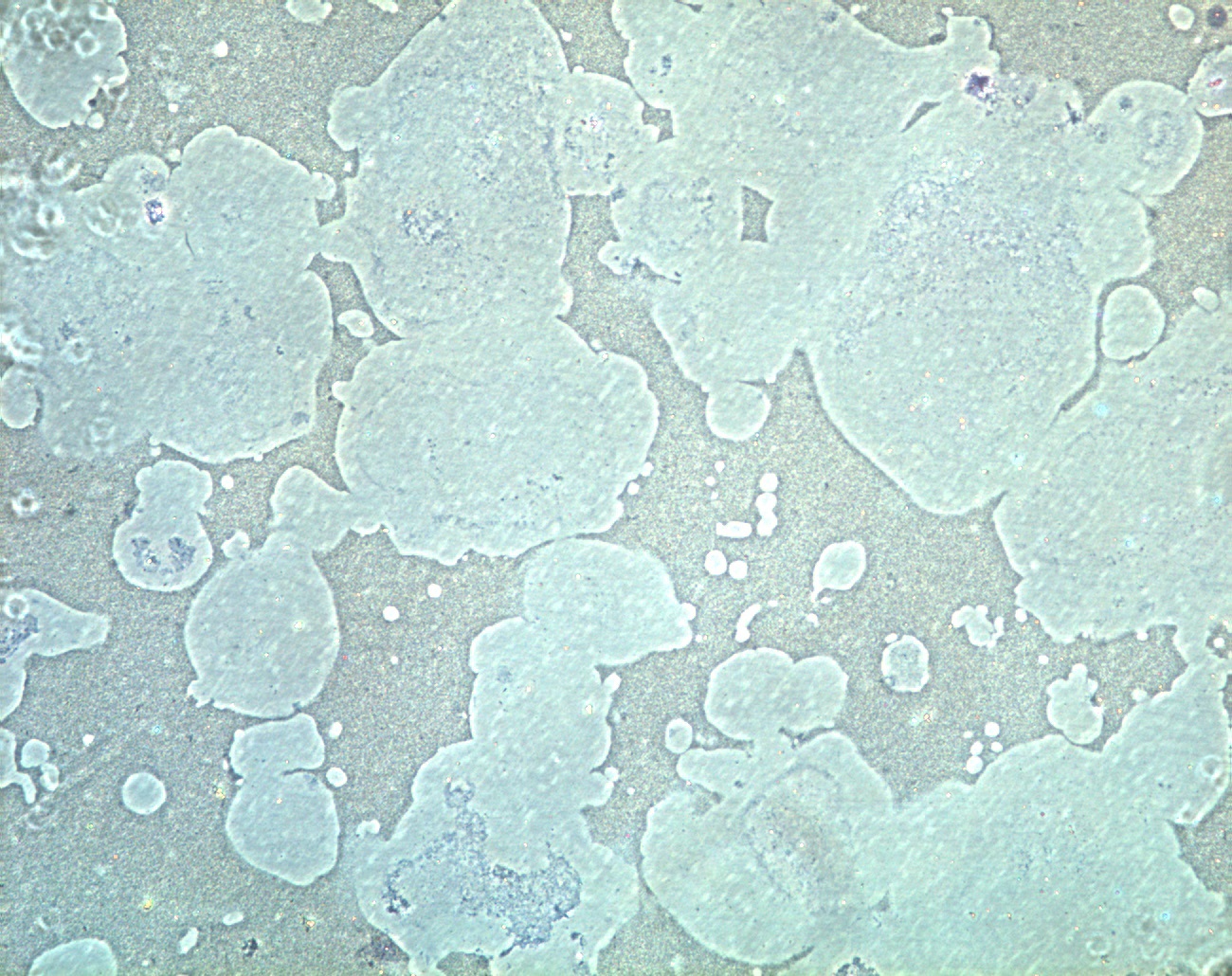

Supplement: Supplementary file 1 [file DataSheet3.ZIP › Fig.3-Source data/E/RANKL.jpg]

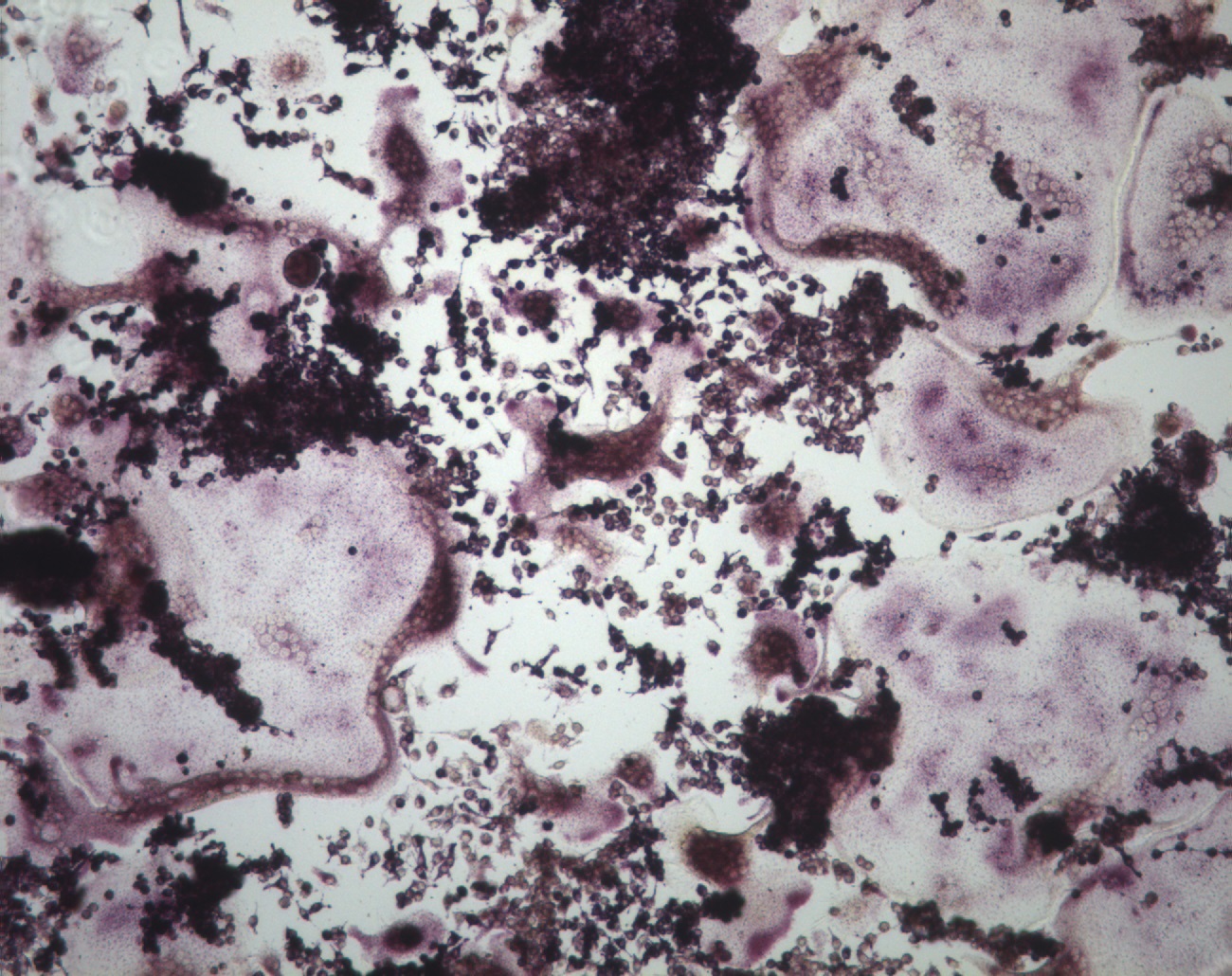

Supplement: Supplementary file 1 [file DataSheet3.ZIP › Fig.3-Source data/E/TRAP-RANKL+sh-NC.jpg]

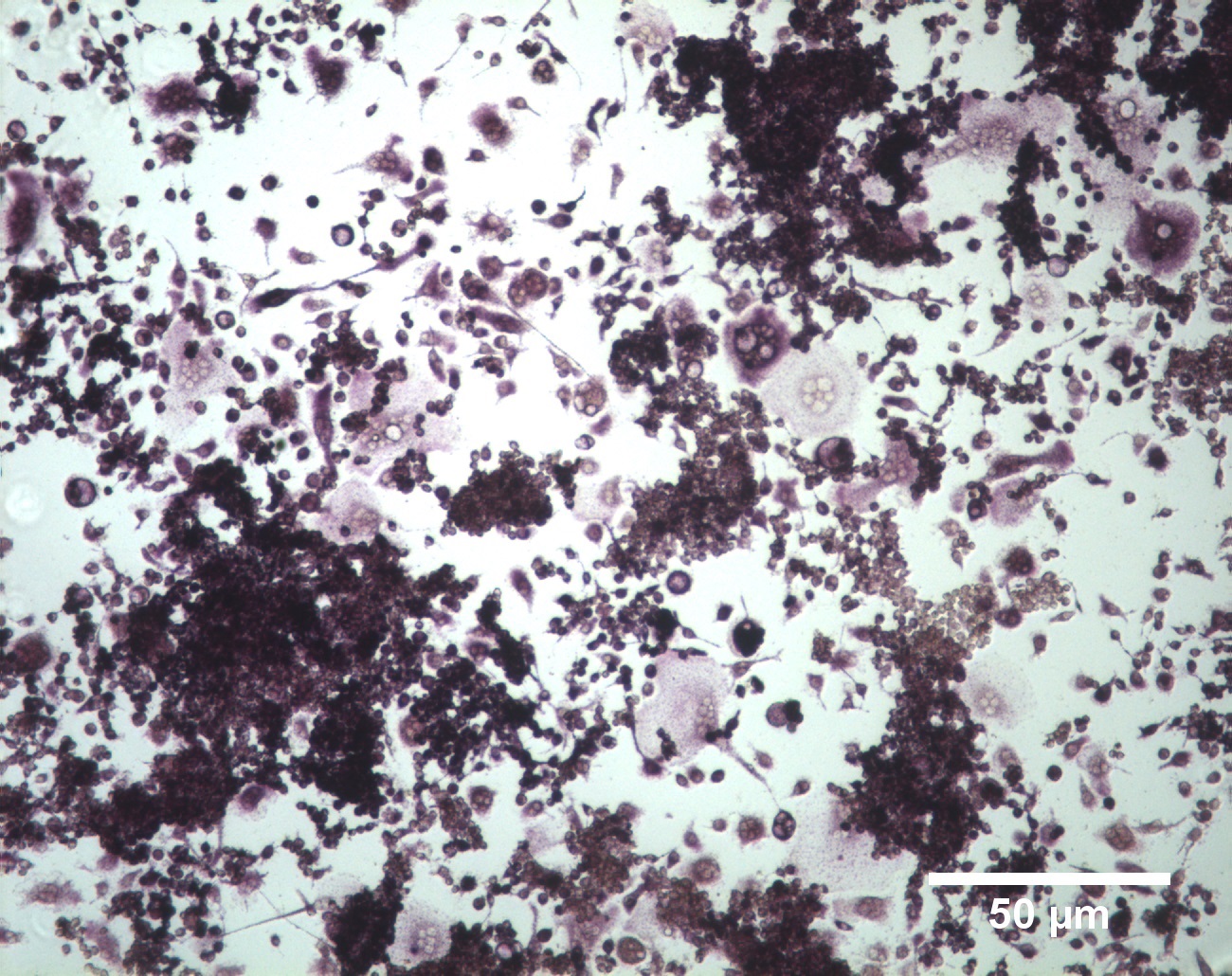

Supplement: Supplementary file 1 [file DataSheet3.ZIP › Fig.3-Source data/E/TRAP-RANKL+sh-Nox4.jpg]

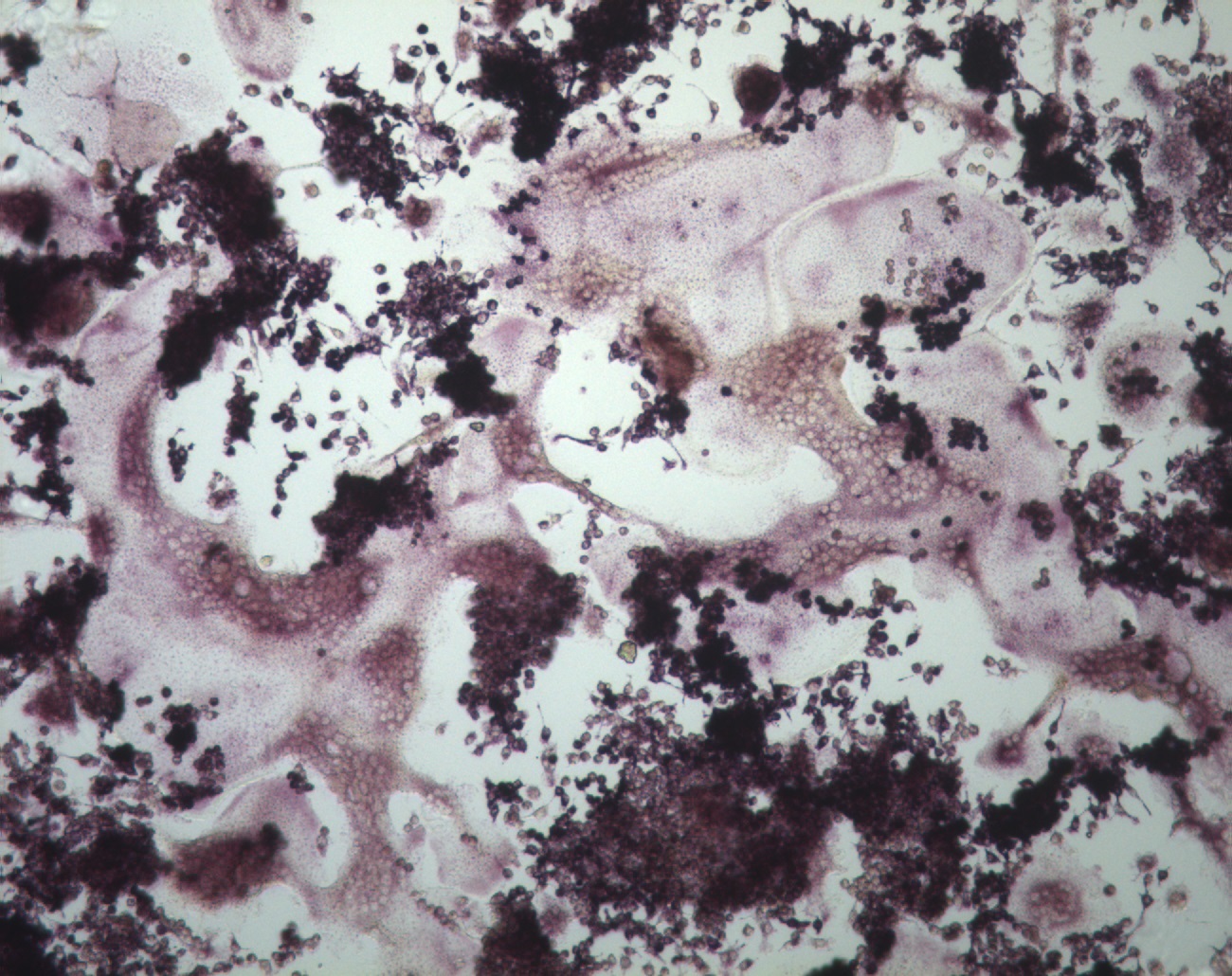

Supplement: Supplementary file 1 [file DataSheet3.ZIP › Fig.3-Source data/E/TRAP-RANKL.jpg]

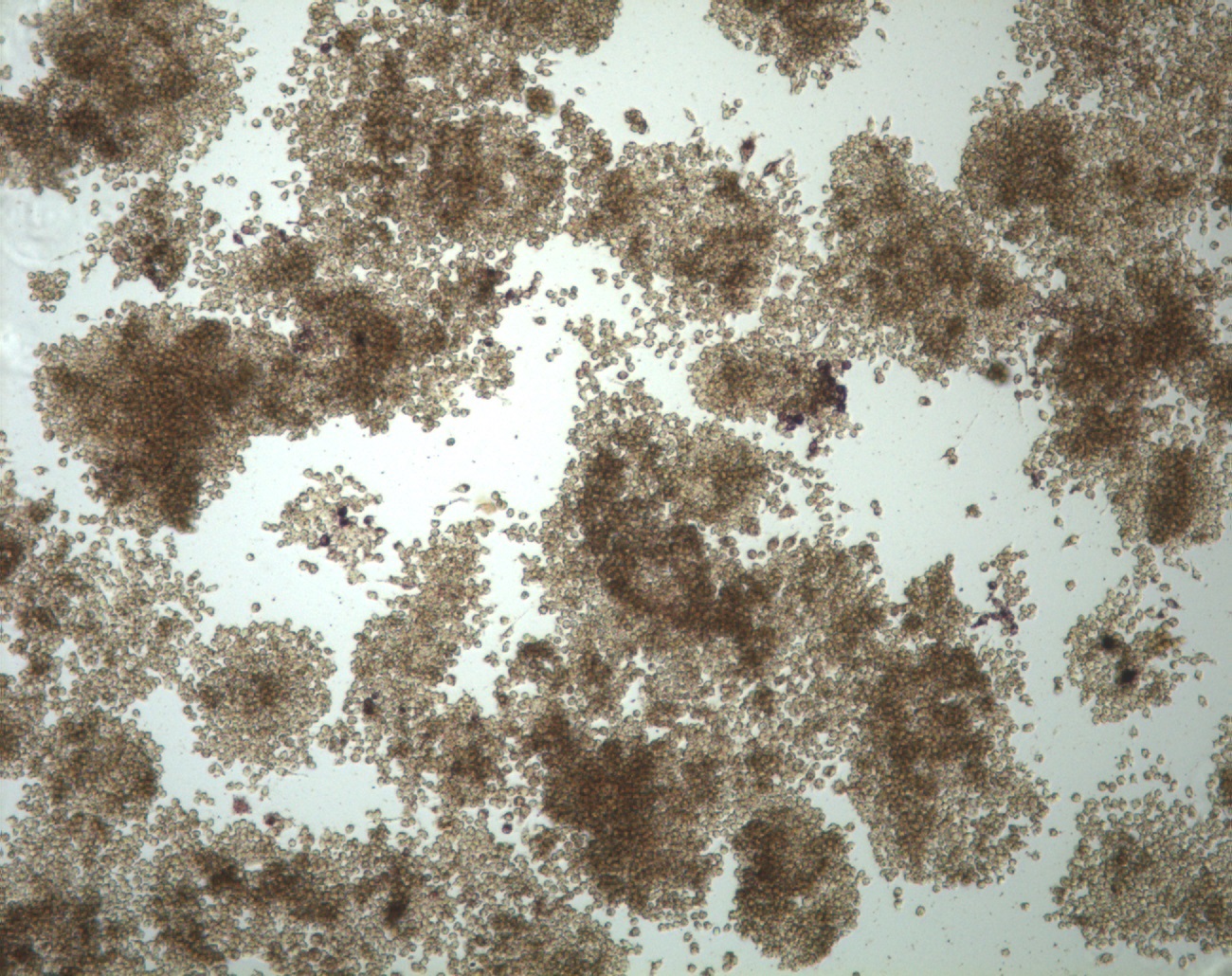

Supplement: Supplementary file 1 [file DataSheet3.ZIP › Fig.3-Source data/E/TRAP-control.jpg]

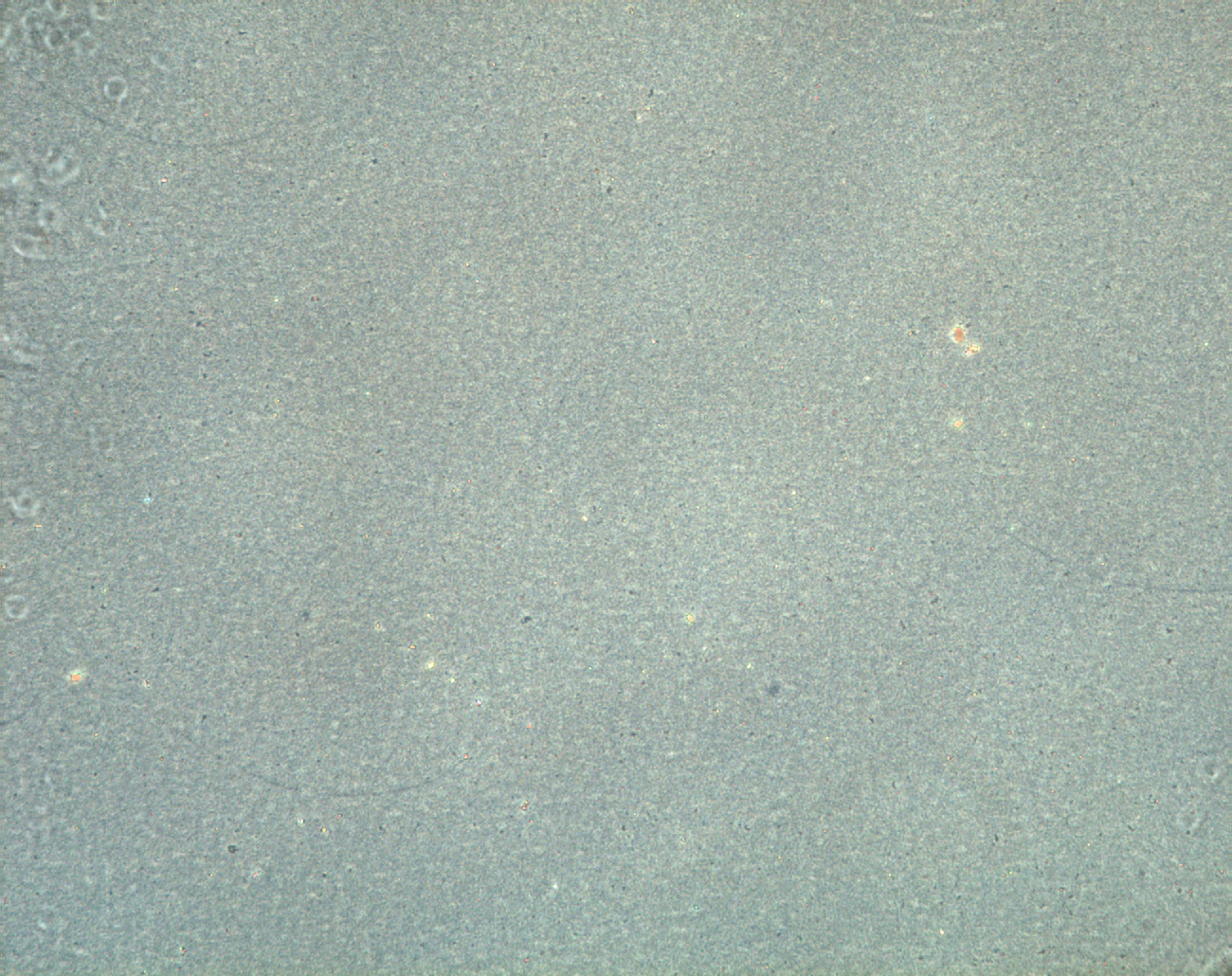

Supplement: Supplementary file 1 [file DataSheet3.ZIP › Fig.3-Source data/E/control.jpg]

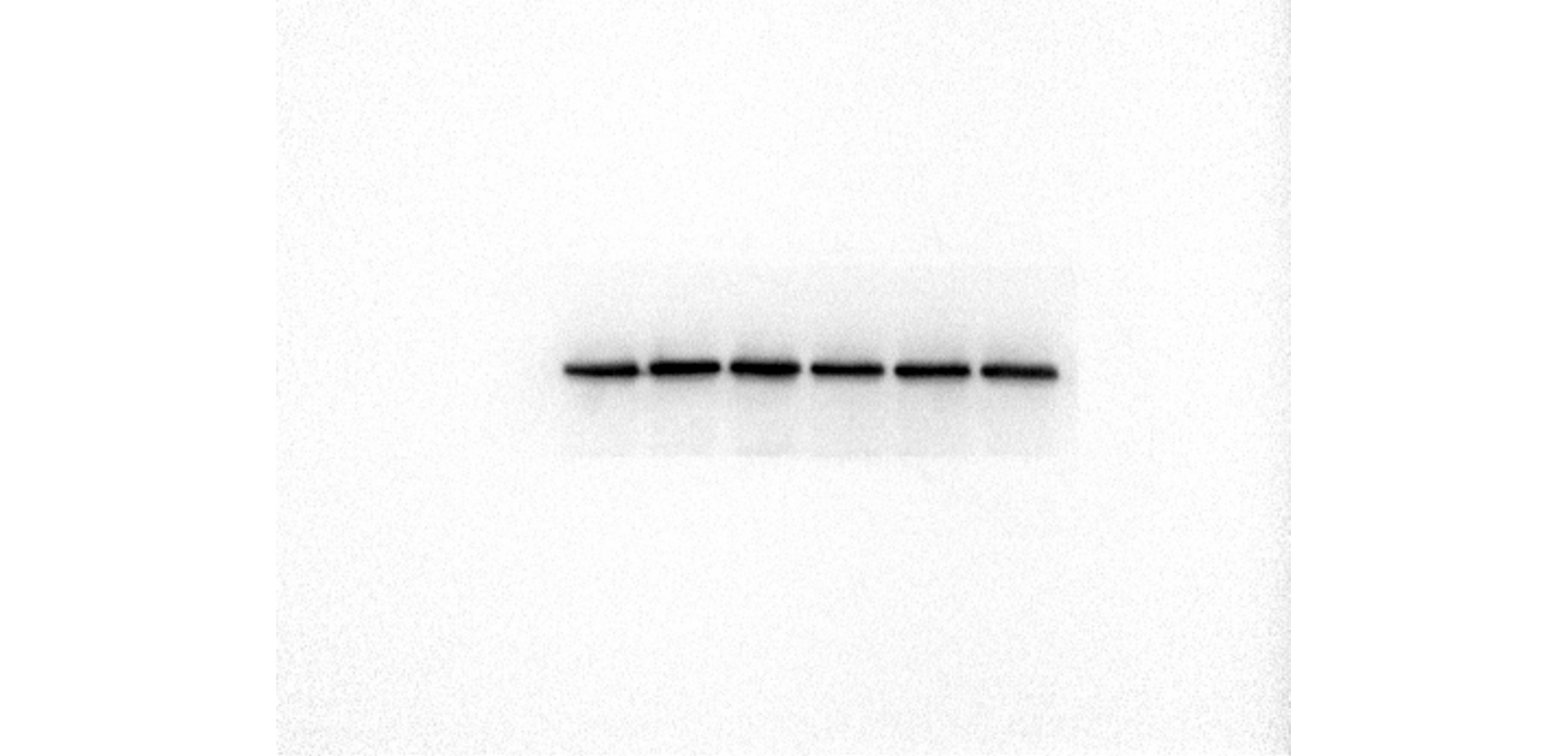

Supplement: Supplementary file 3 [file DataSheet4.ZIP › Fig.4-Source data/A/Nox4.tif]

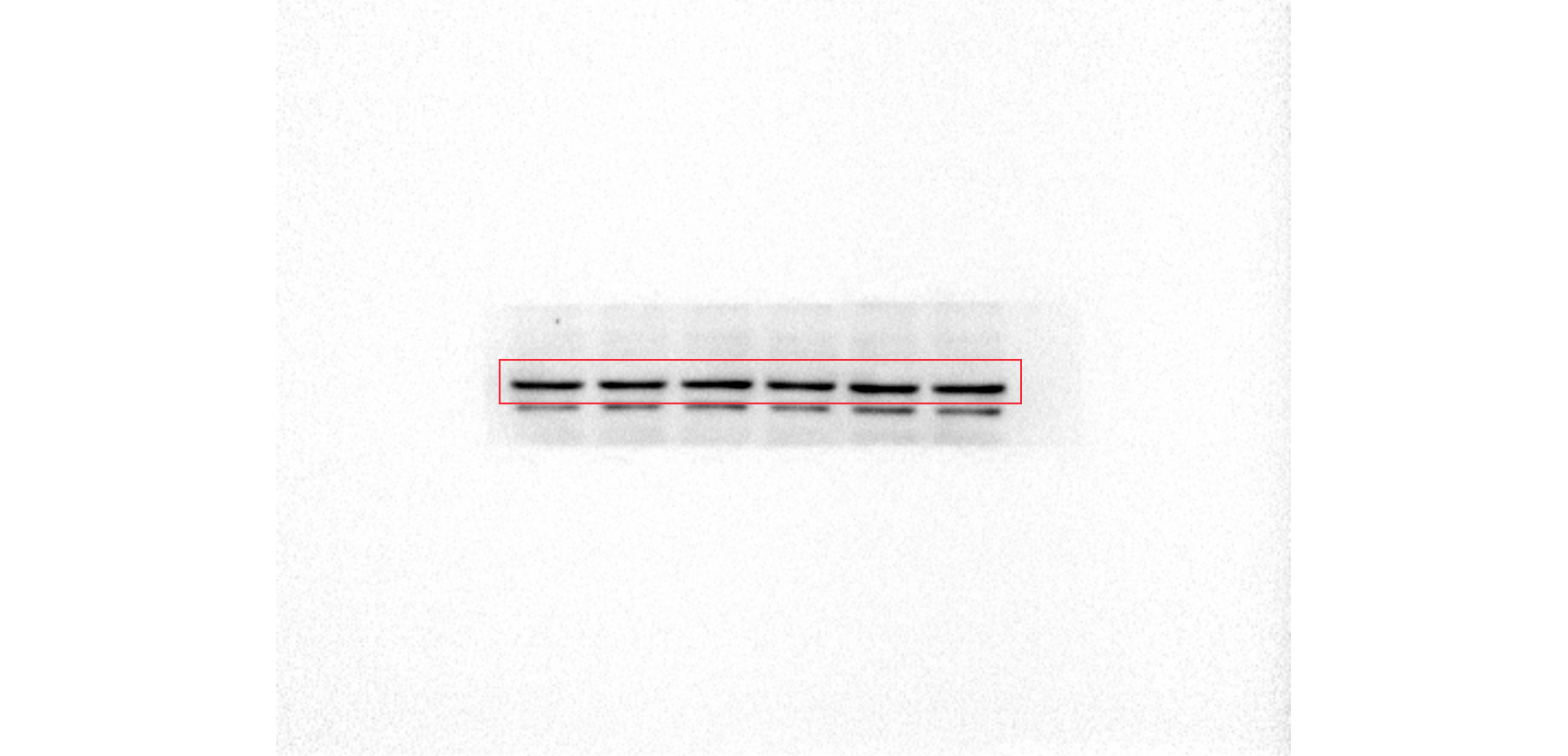

Supplement: Supplementary file 3 [file DataSheet4.ZIP › Fig.4-Source data/A/VDAC1.tif]

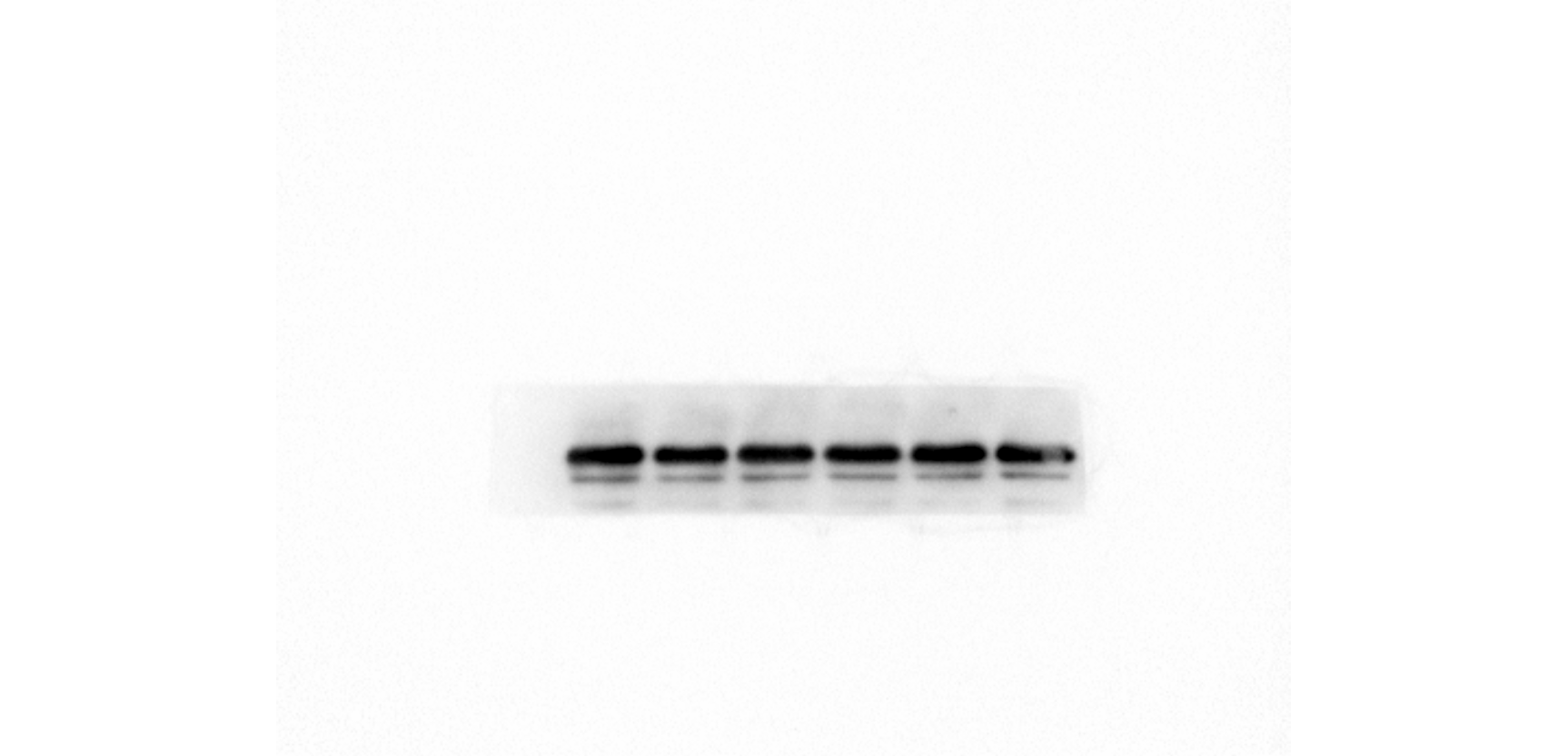

Supplement: Supplementary file 3 [file DataSheet4.ZIP › Fig.4-Source data/B/ERP57.tif]

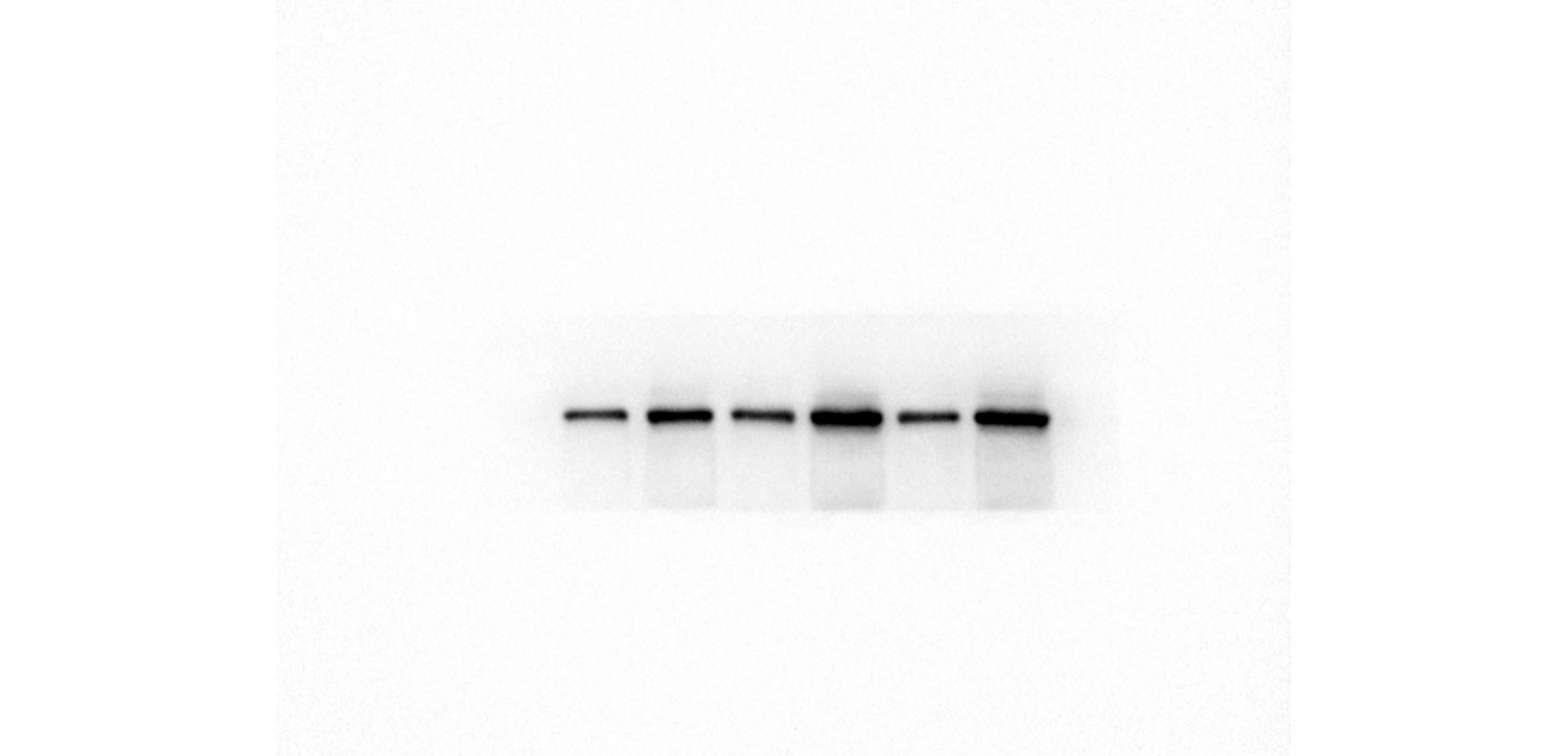

Supplement: Supplementary file 3 [file DataSheet4.ZIP › Fig.4-Source data/B/Nox4.tif]

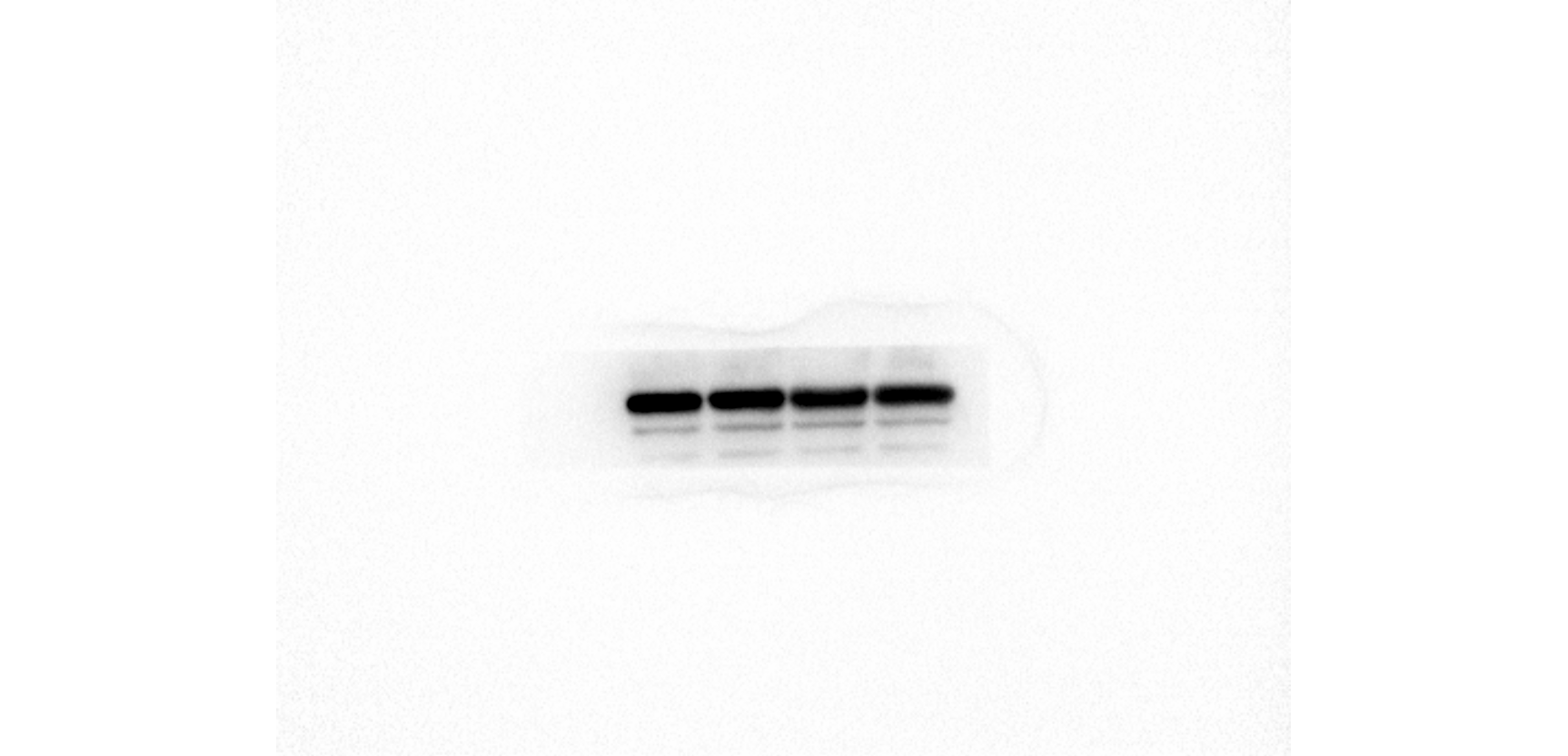

Supplement: Supplementary file 3 [file DataSheet4.ZIP › Fig.4-Source data/C/ERp57.tif]

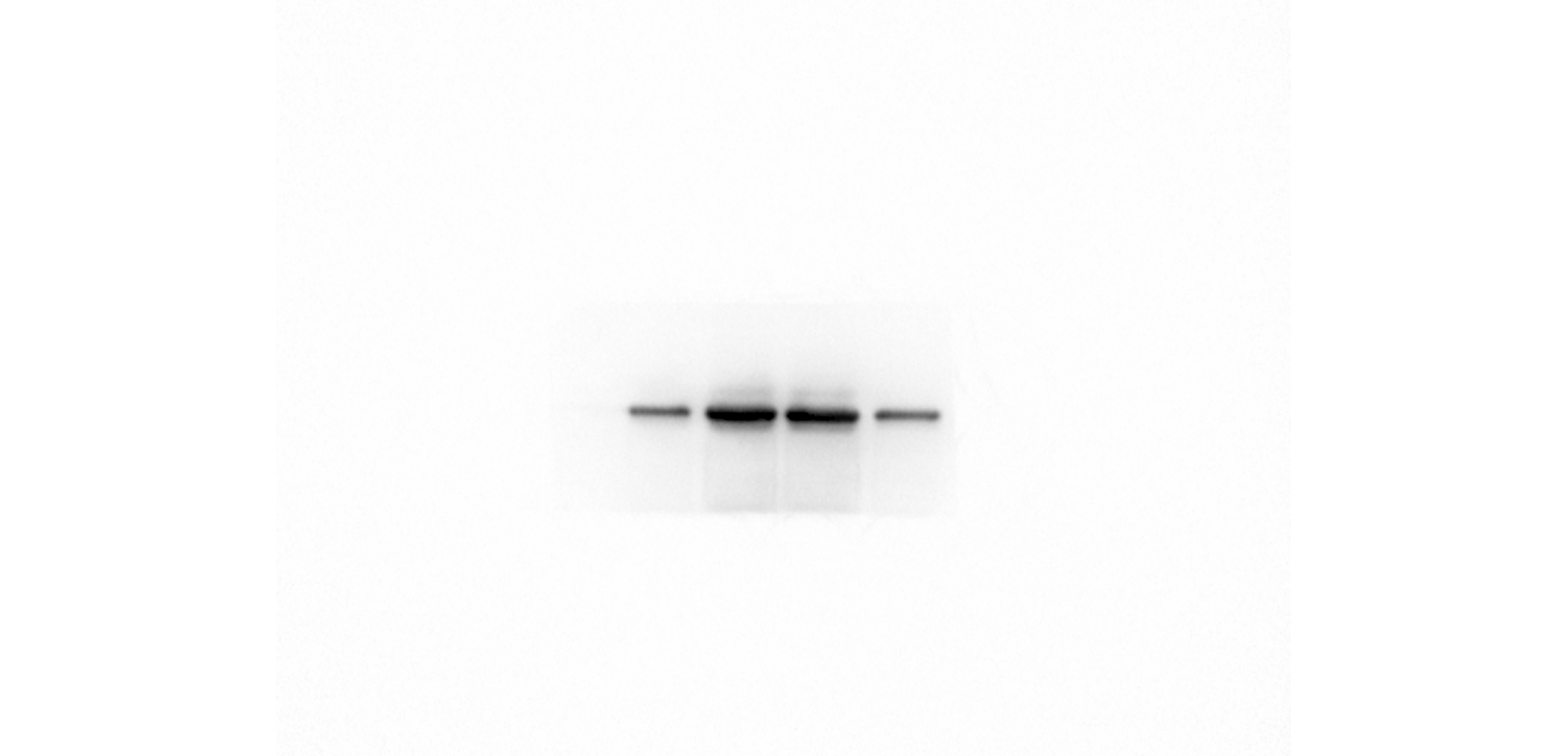

Supplement: Supplementary file 3 [file DataSheet4.ZIP › Fig.4-Source data/C/Nox4.tif]

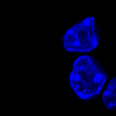

Supplement: Supplementary file 3 [file DataSheet4.ZIP › Fig.4-Source data/D/Control-DAPI.tif]

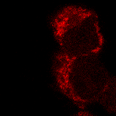

Supplement: Supplementary file 3 [file DataSheet4.ZIP › Fig.4-Source data/D/Control-ER-Tracker.tif]

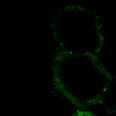

Supplement: Supplementary file 3 [file DataSheet4.ZIP › Fig.4-Source data/D/Control-Nox4.tif]

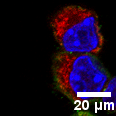

Supplement: Supplementary file 3 [file DataSheet4.ZIP › Fig.4-Source data/D/Control-merged.tif]

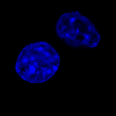

Supplement: Supplementary file 3 [file DataSheet4.ZIP › Fig.4-Source data/D/RANKL+sh-NC-DAPI.tif]

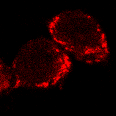

Supplement: Supplementary file 3 [file DataSheet4.ZIP › Fig.4-Source data/D/RANKL+sh-NC-ER-Tracker.tif]

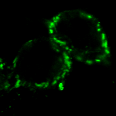

Supplement: Supplementary file 3 [file DataSheet4.ZIP › Fig.4-Source data/D/RANKL+sh-NC-Nox4.tif]

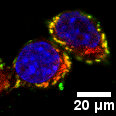

Supplement: Supplementary file 3 [file DataSheet4.ZIP › Fig.4-Source data/D/RANKL+sh-NC-merged.tif]

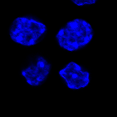

Supplement: Supplementary file 3 [file DataSheet4.ZIP › Fig.4-Source data/D/RANKL+sh-Nox4-DAPI.tif]

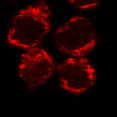

Supplement: Supplementary file 3 [file DataSheet4.ZIP › Fig.4-Source data/D/RANKL+sh-Nox4-ER-Tracker.tif]

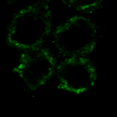

Supplement: Supplementary file 3 [file DataSheet4.ZIP › Fig.4-Source data/D/RANKL+sh-Nox4-Nox4.tif]

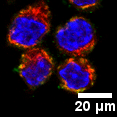

Supplement: Supplementary file 3 [file DataSheet4.ZIP › Fig.4-Source data/D/RANKL+sh-Nox4-merged.tif]

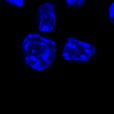

Supplement: Supplementary file 3 [file DataSheet4.ZIP › Fig.4-Source data/D/RANKL-DAPI.tif]

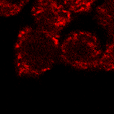

Supplement: Supplementary file 3 [file DataSheet4.ZIP › Fig.4-Source data/D/RANKL-ER-Tracker.tif]

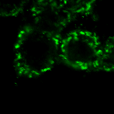

Supplement: Supplementary file 3 [file DataSheet4.ZIP › Fig.4-Source data/D/RANKL-Nox4.tif]

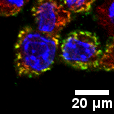

Supplement: Supplementary file 3 [file DataSheet4.ZIP › Fig.4-Source data/D/RANKL-merged.tif]

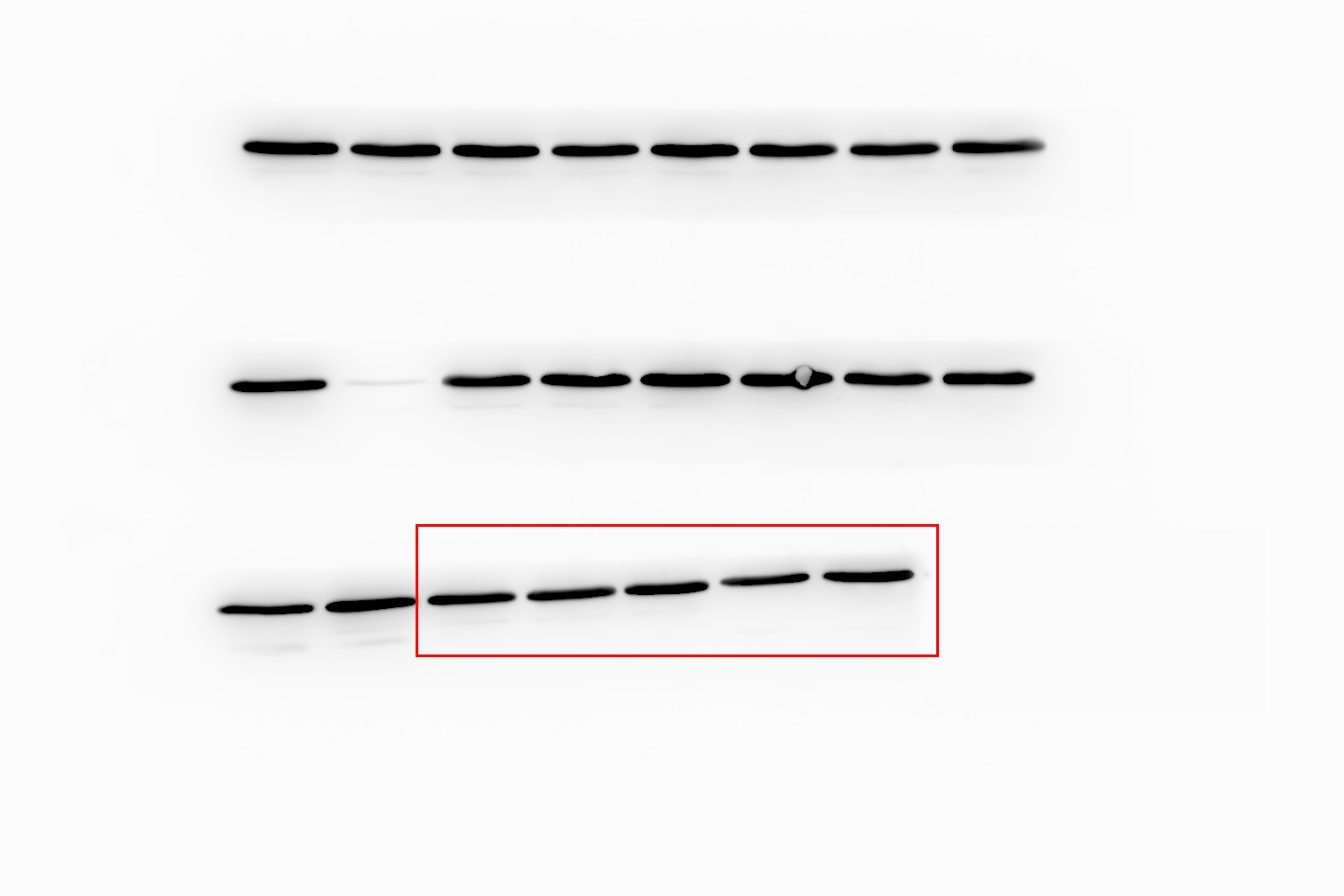

Supplement: Supplementary file 4 [file DataSheet1.ZIP › Fig.1-Source data/A/GAPDH-1.jpg]

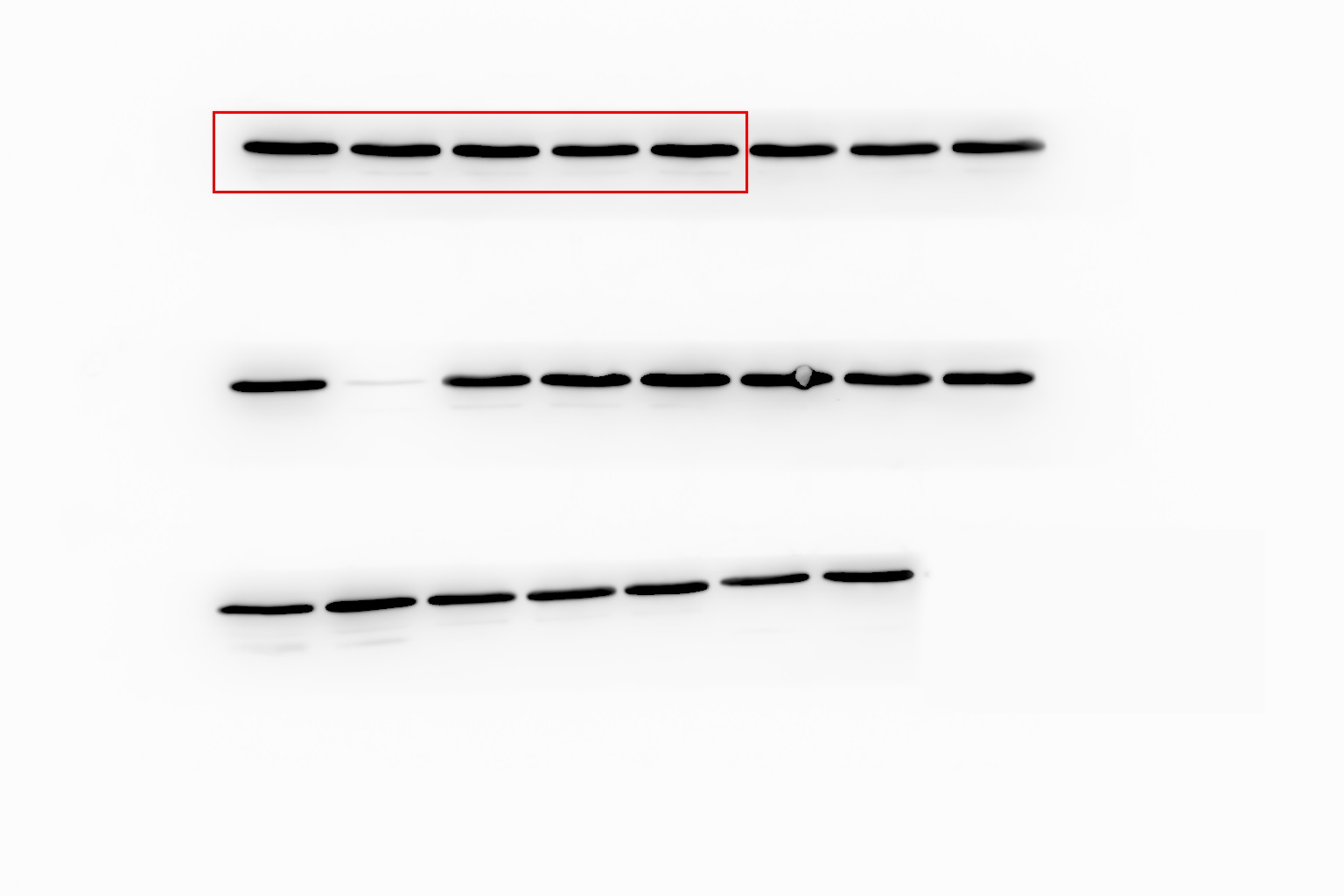

Supplement: Supplementary file 4 [file DataSheet1.ZIP › Fig.1-Source data/A/GAPDH-2.jpg]

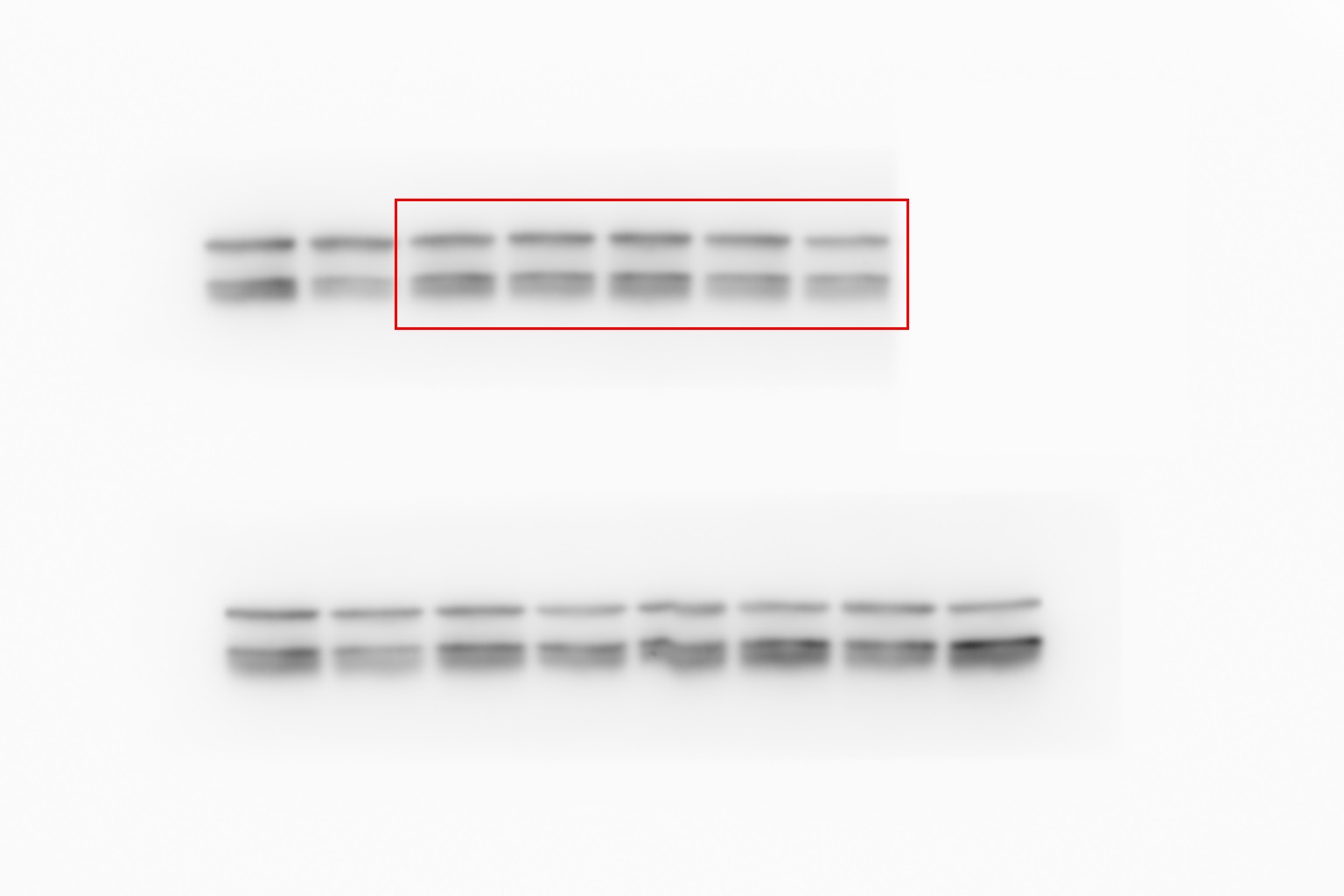

Supplement: Supplementary file 4 [file DataSheet1.ZIP › Fig.1-Source data/A/LC3-1.jpg]

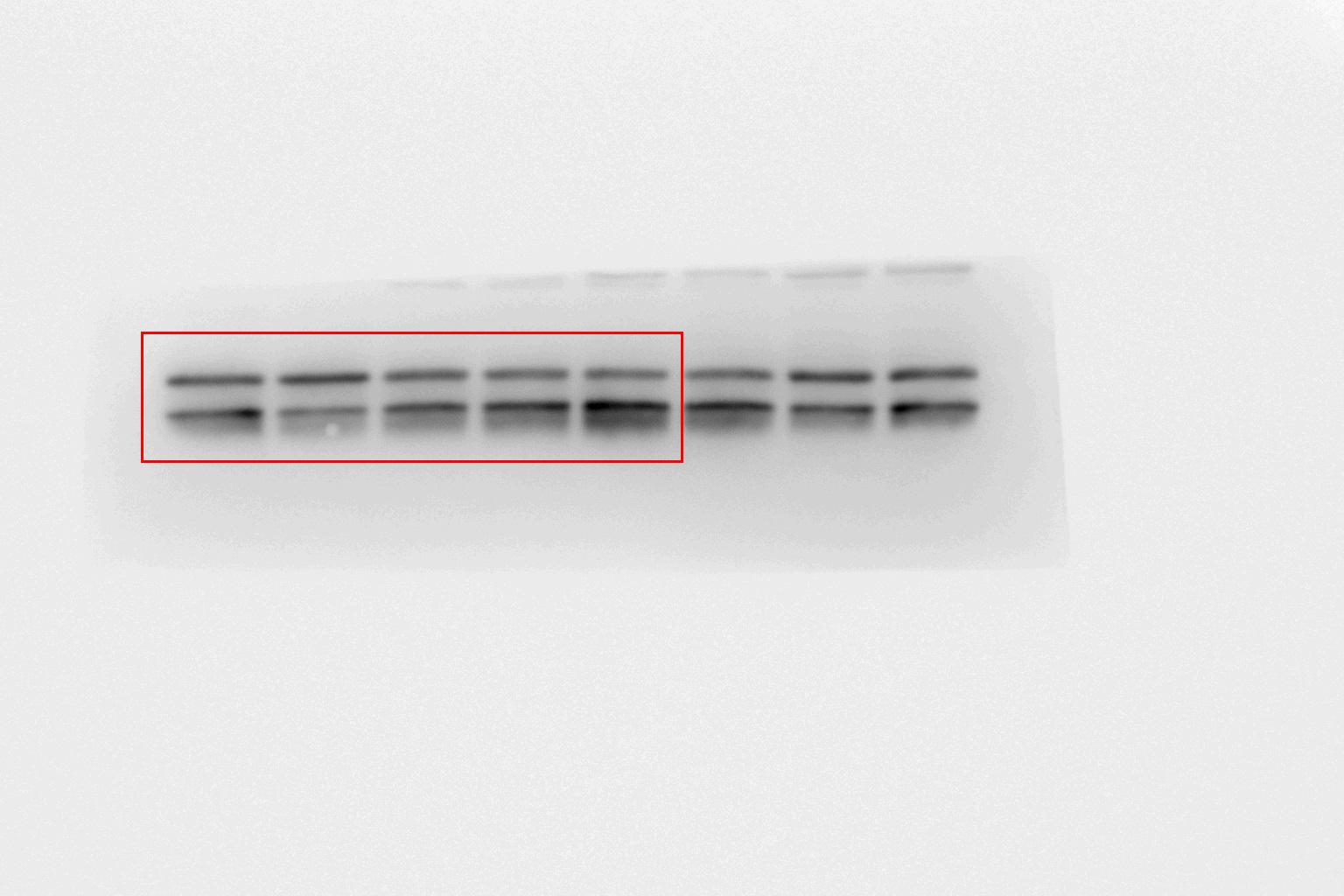

Supplement: Supplementary file 4 [file DataSheet1.ZIP › Fig.1-Source data/A/LC3-2.jpg]

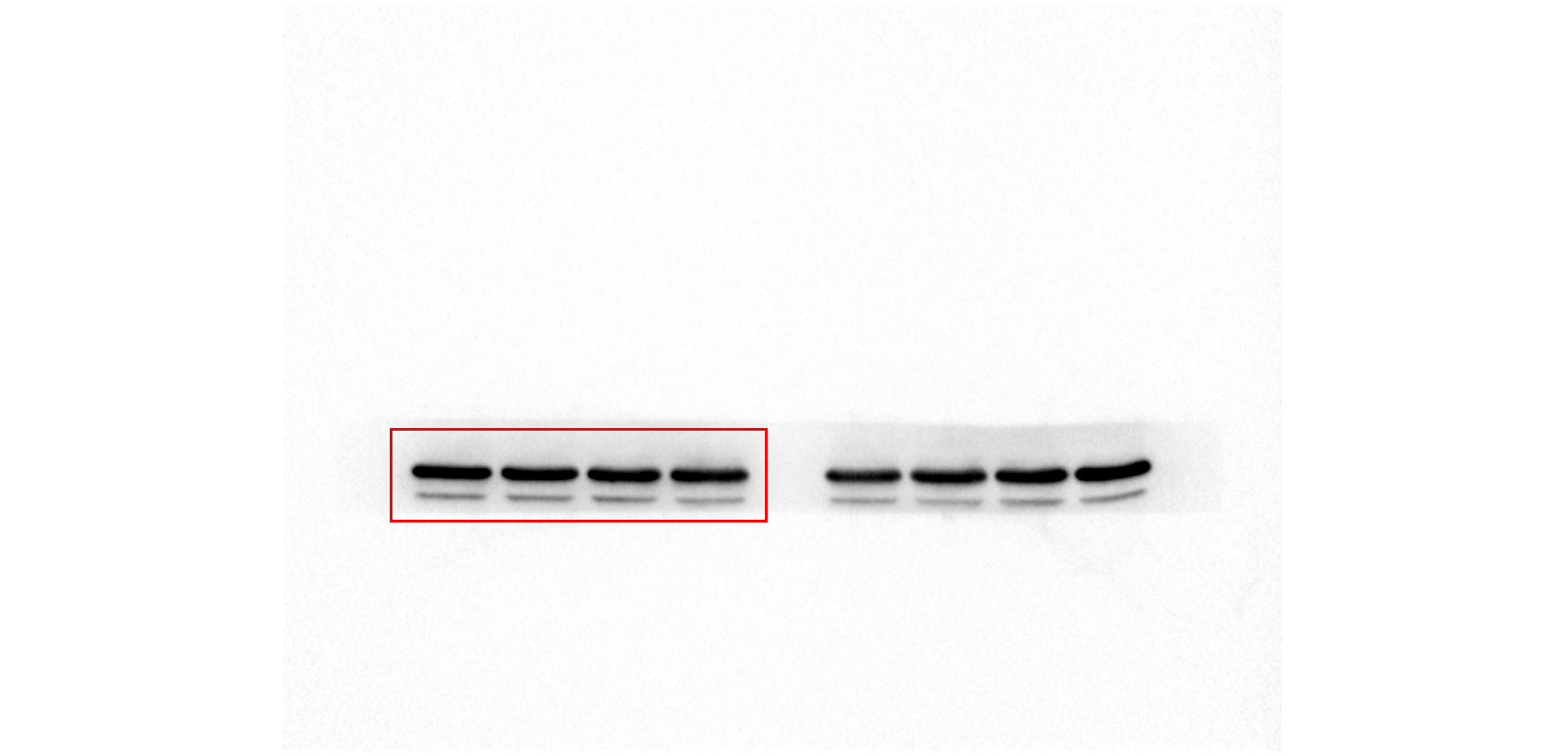

Supplement: Supplementary file 4 [file DataSheet1.ZIP › Fig.1-Source data/B/GAPDH.tif]

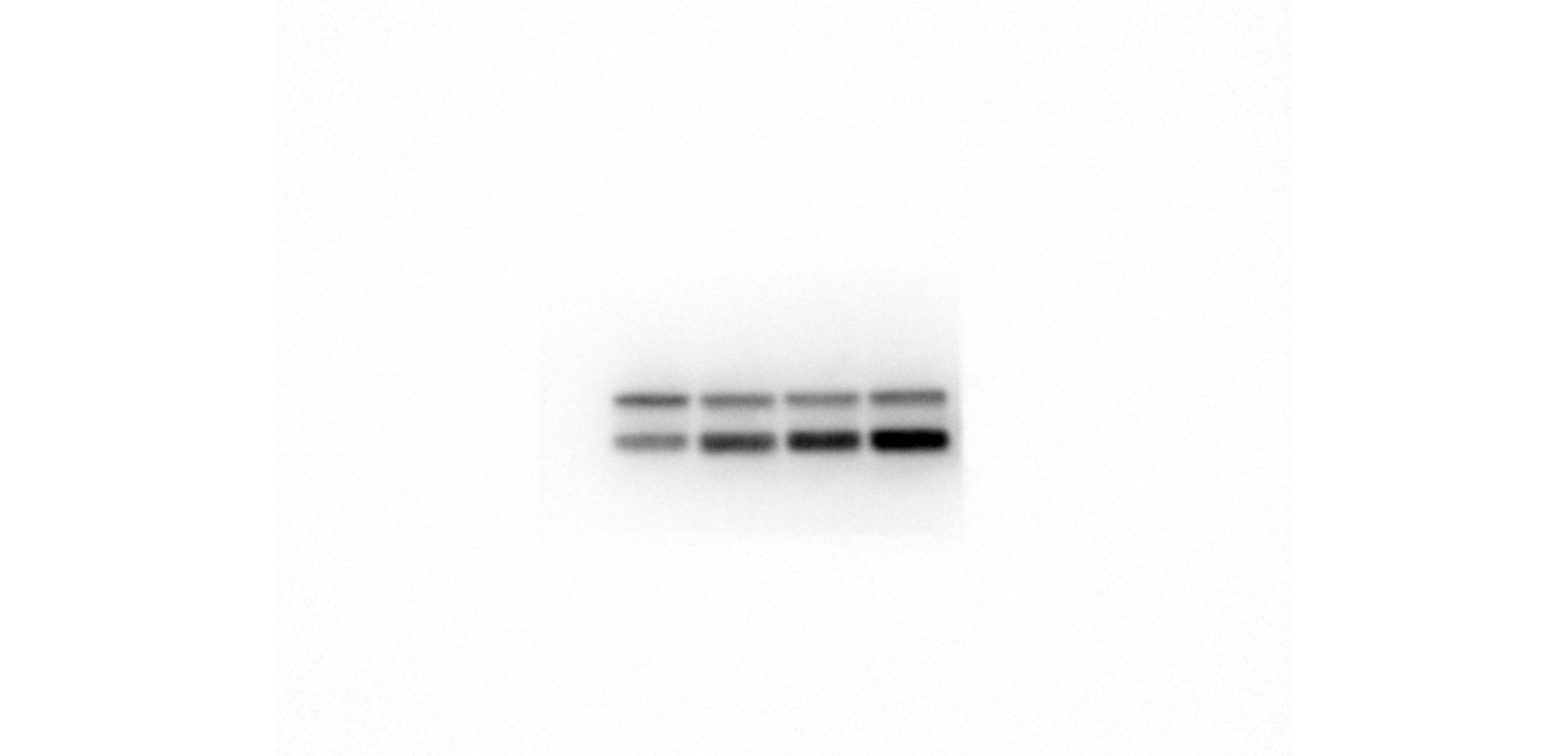

Supplement: Supplementary file 4 [file DataSheet1.ZIP › Fig.1-Source data/B/LC3.tif]

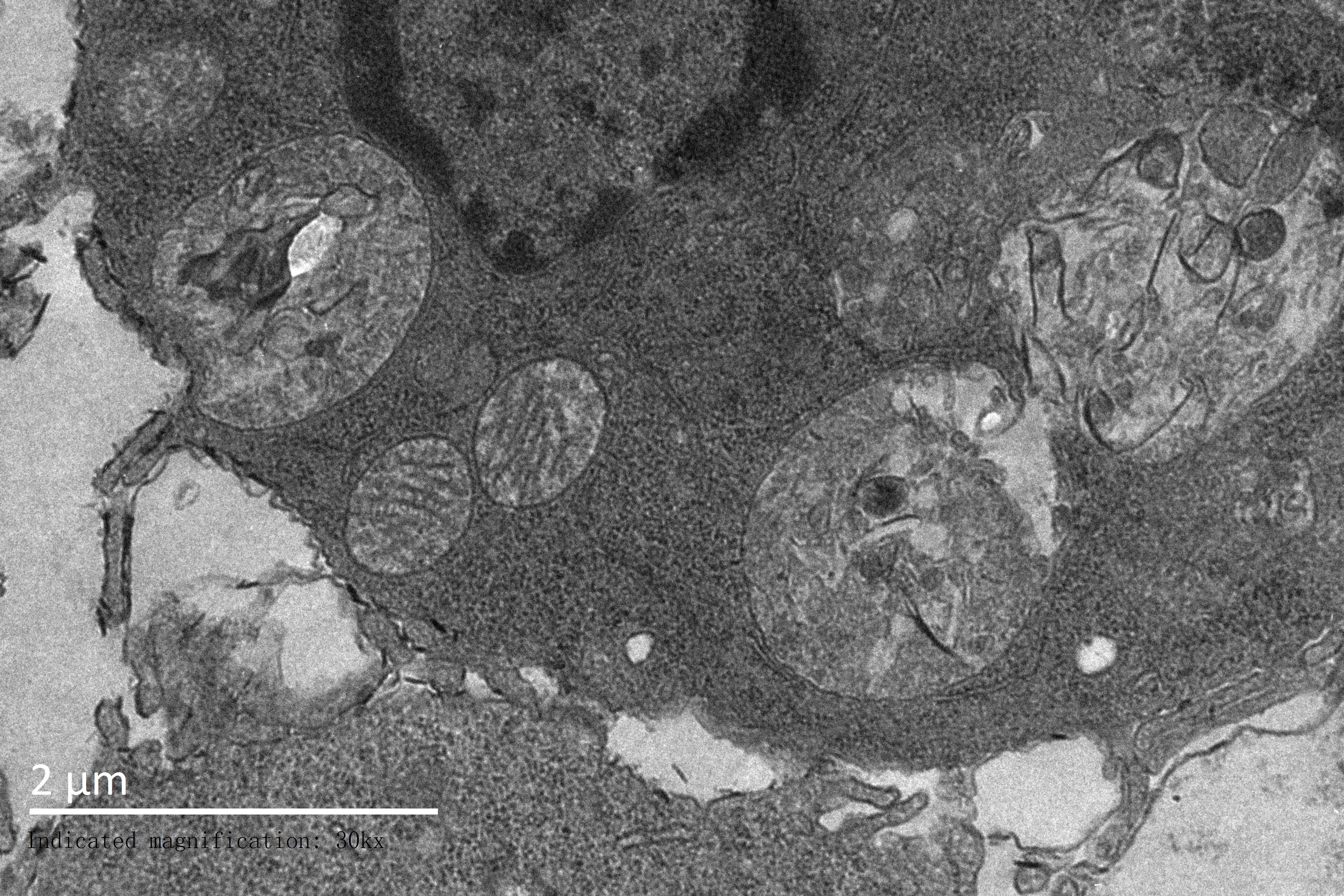

Supplement: Supplementary file 4 [file DataSheet1.ZIP › Fig.1-Source data/C/RANKL+CQ.jpg]

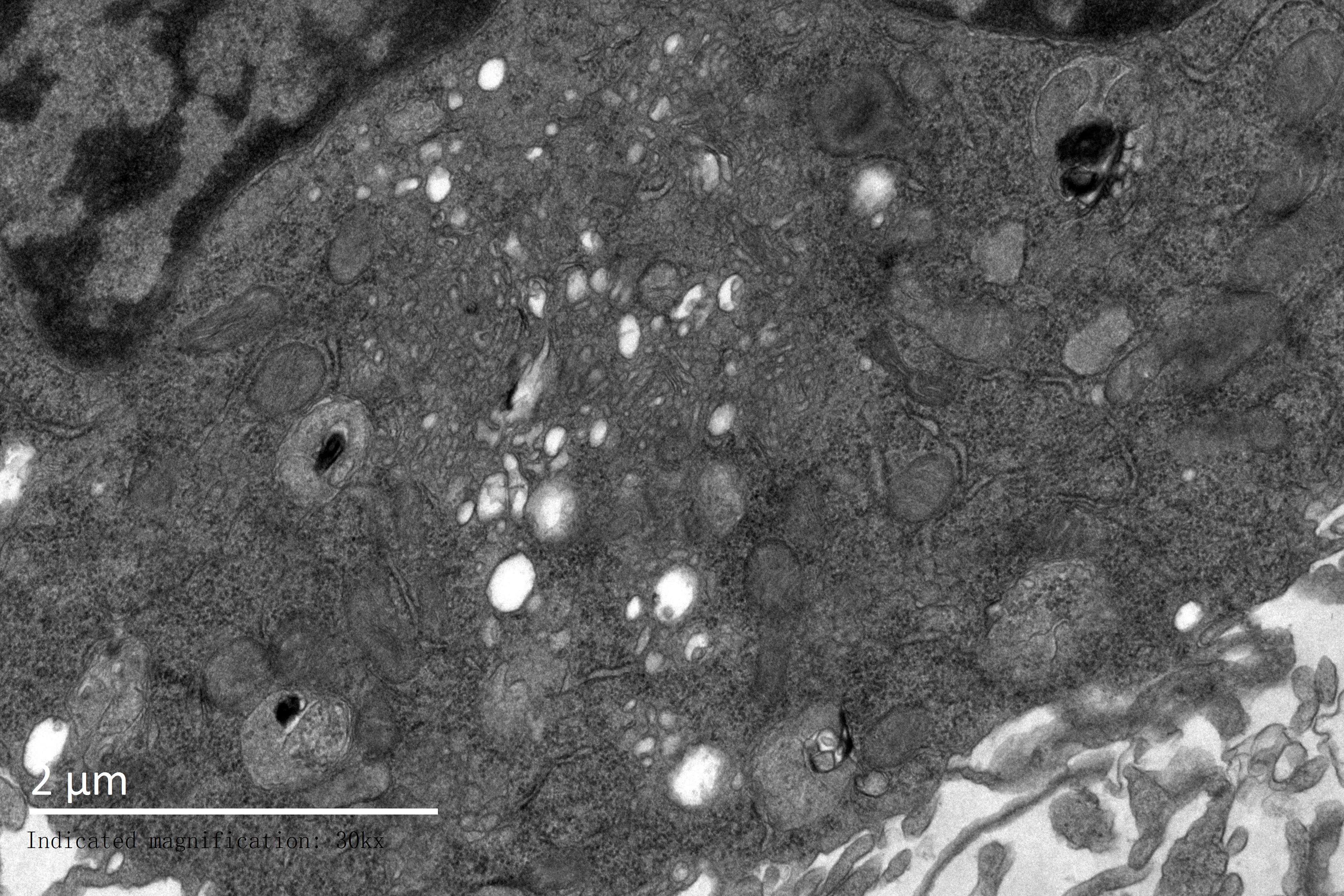

Supplement: Supplementary file 4 [file DataSheet1.ZIP › Fig.1-Source data/C/RANKL.jpg]

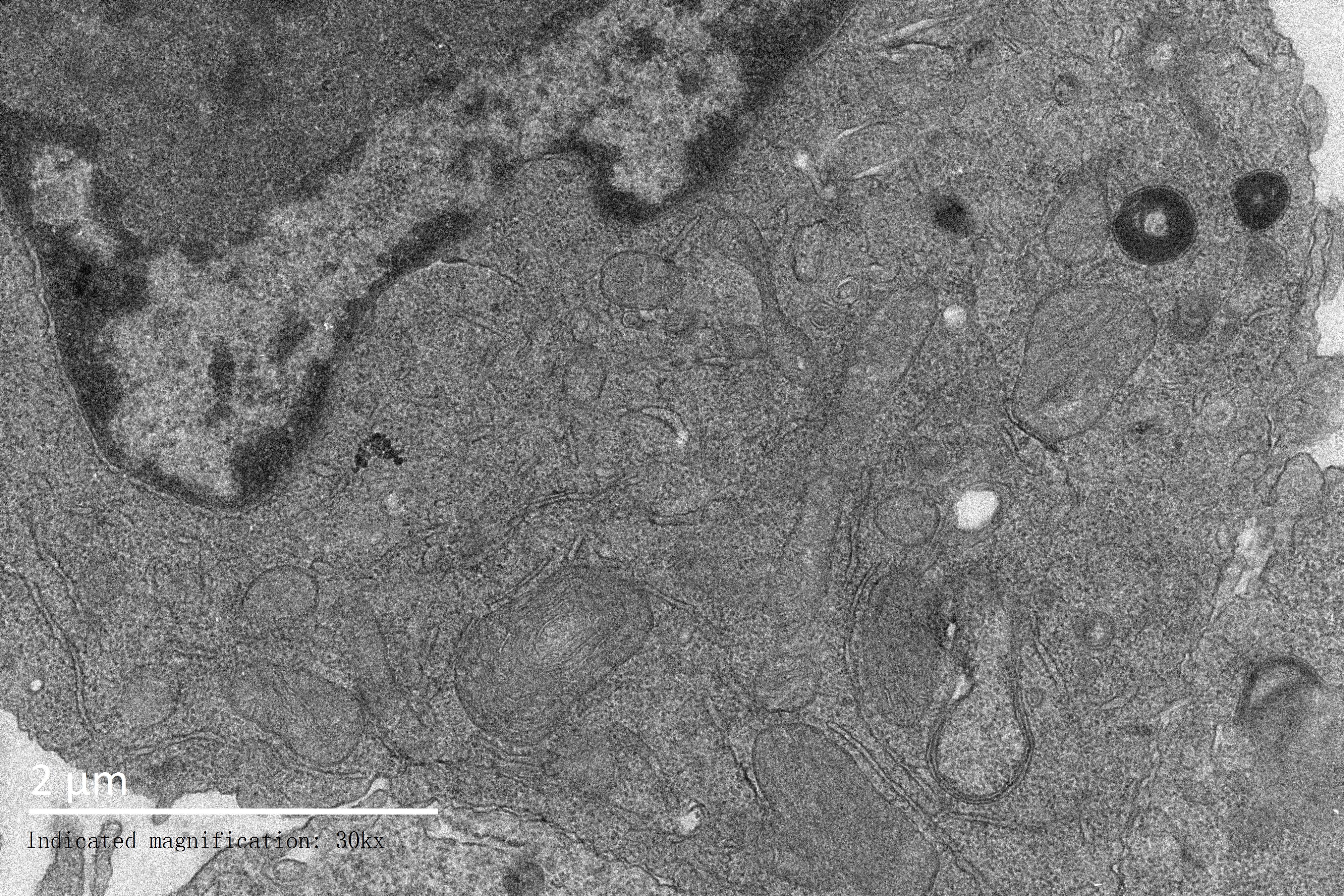

Supplement: Supplementary file 4 [file DataSheet1.ZIP › Fig.1-Source data/C/control.jpg]

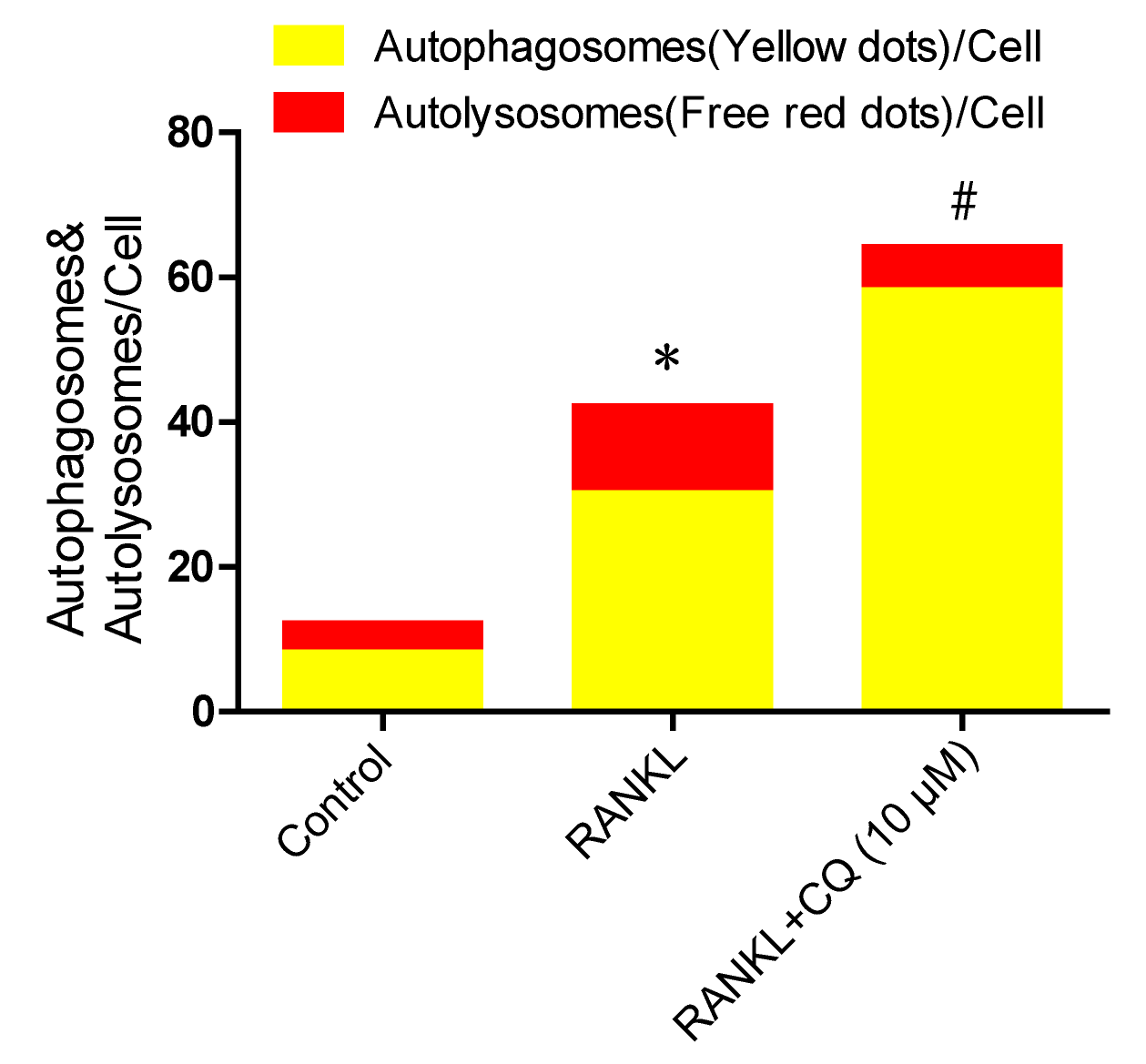

Supplement: Supplementary file 4 [file DataSheet1.ZIP › Fig.1-Source data/D/Fig.1D.tif]

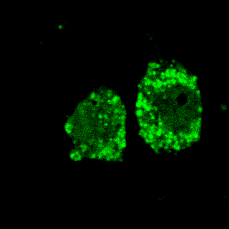

Supplement: Supplementary file 4 [file DataSheet1.ZIP › Fig.1-Source data/D/RANKL+CQ-GFP.tif]

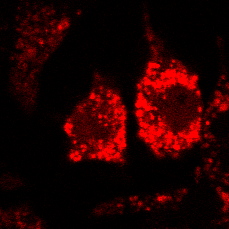

Supplement: Supplementary file 4 [file DataSheet1.ZIP › Fig.1-Source data/D/RANKL+CQ-mRFP.tif]

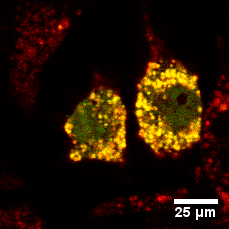

Supplement: Supplementary file 4 [file DataSheet1.ZIP › Fig.1-Source data/D/RANKL+CQ-merged.tif]

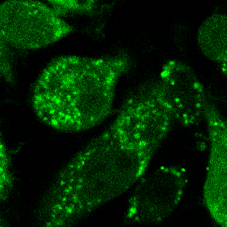

Supplement: Supplementary file 4 [file DataSheet1.ZIP › Fig.1-Source data/D/RANKL-GFP.tif]

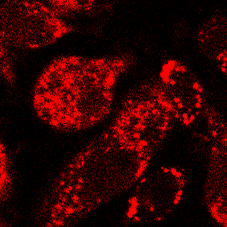

Supplement: Supplementary file 4 [file DataSheet1.ZIP › Fig.1-Source data/D/RANKL-mRFP.tif]

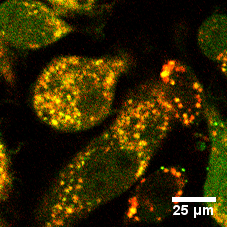

Supplement: Supplementary file 4 [file DataSheet1.ZIP › Fig.1-Source data/D/RANKL-merged.tif]

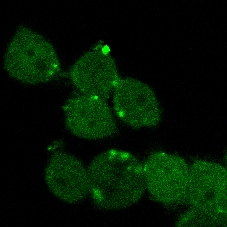

Supplement: Supplementary file 4 [file DataSheet1.ZIP › Fig.1-Source data/D/control-GFP.tif]

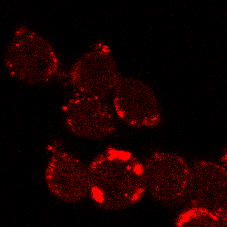

Supplement: Supplementary file 4 [file DataSheet1.ZIP › Fig.1-Source data/D/control-mRFP.tif]

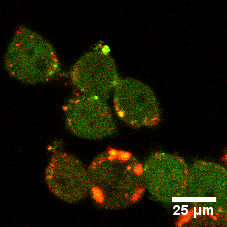

Supplement: Supplementary file 4 [file DataSheet1.ZIP › Fig.1-Source data/D/control-merged.tif]

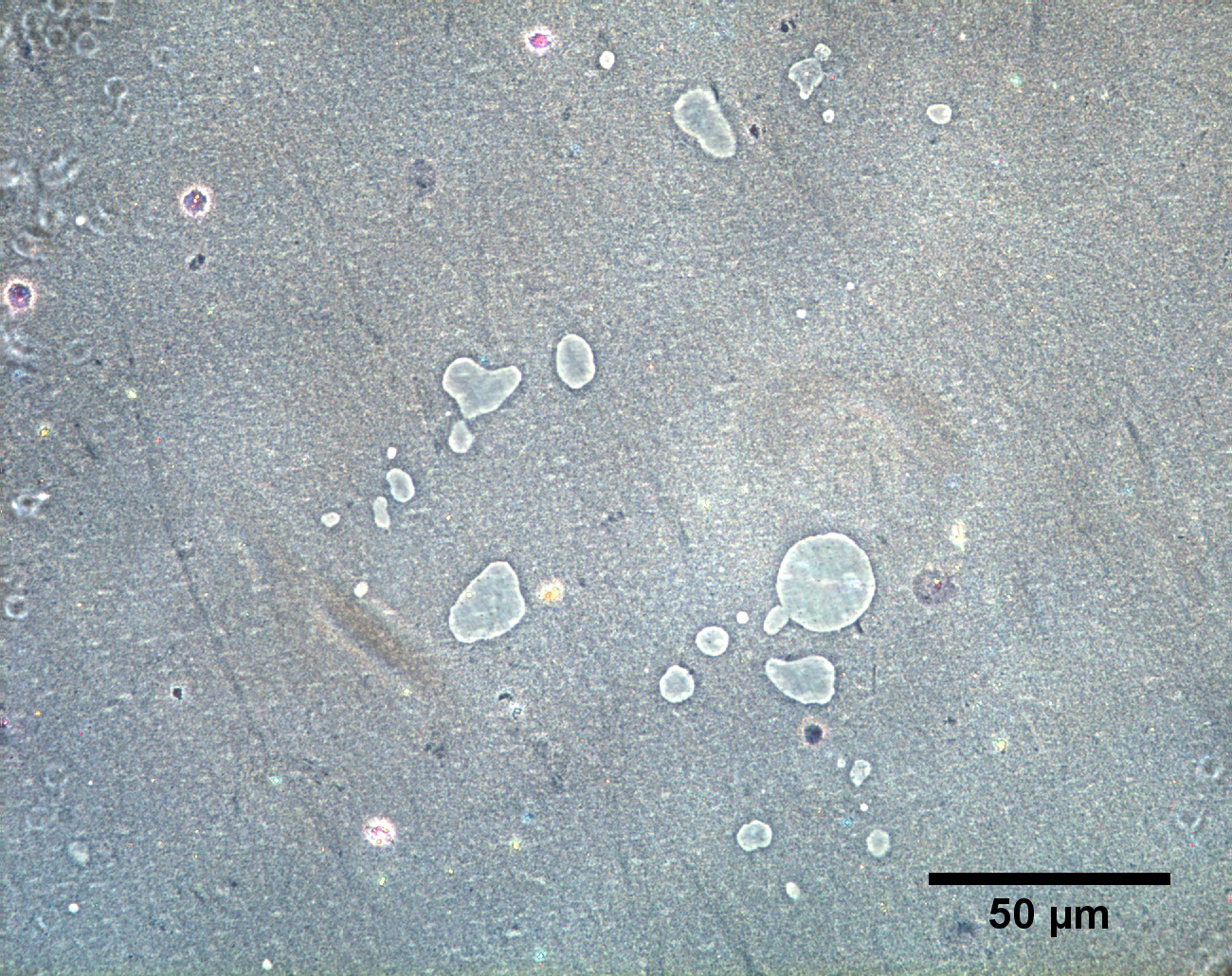

Supplement: Supplementary file 4 [file DataSheet1.ZIP › Fig.1-Source data/F/RANKL+CQ (10 uM).jpg]

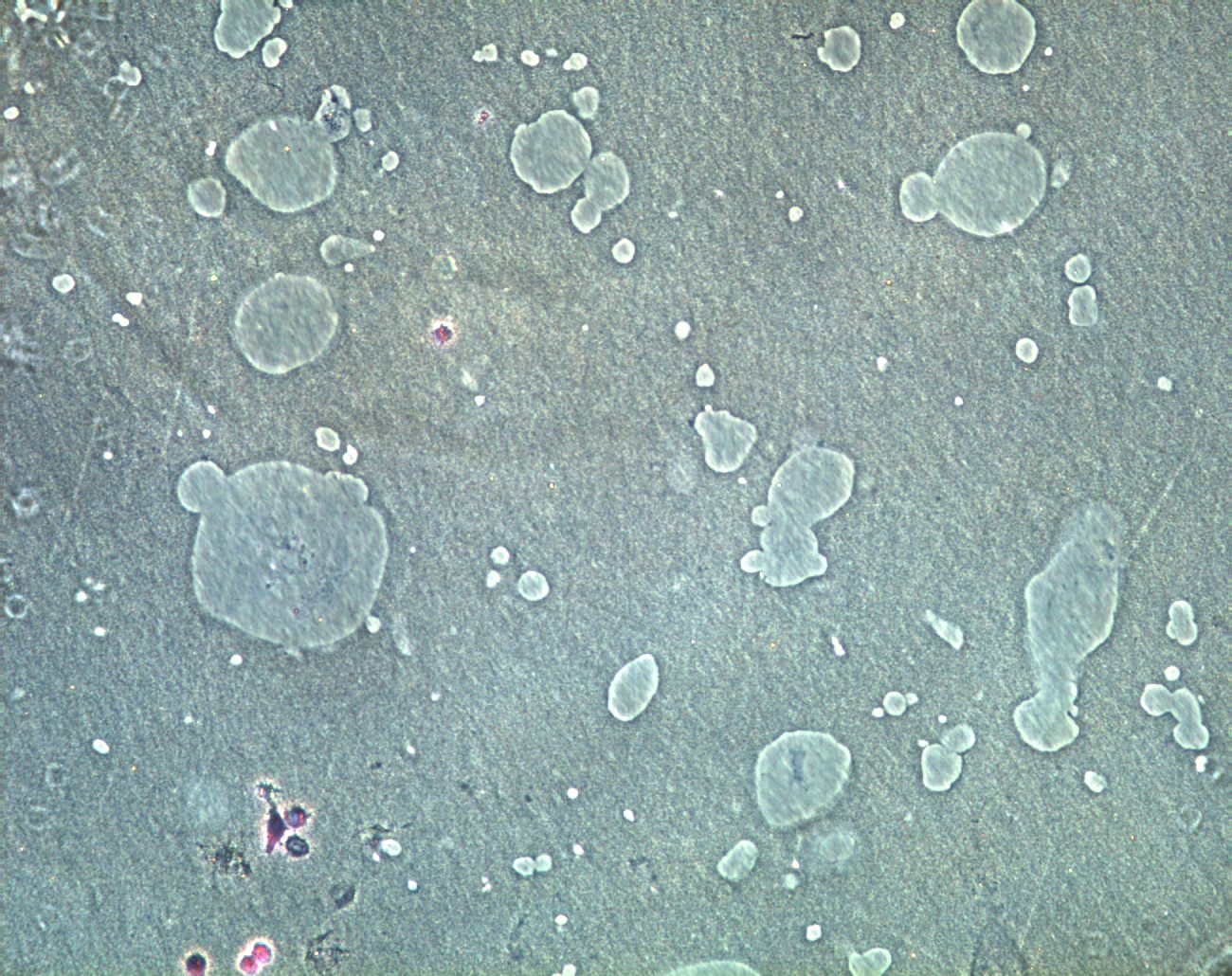

Supplement: Supplementary file 4 [file DataSheet1.ZIP › Fig.1-Source data/F/RANKL+CQ (5 uM).jpg]

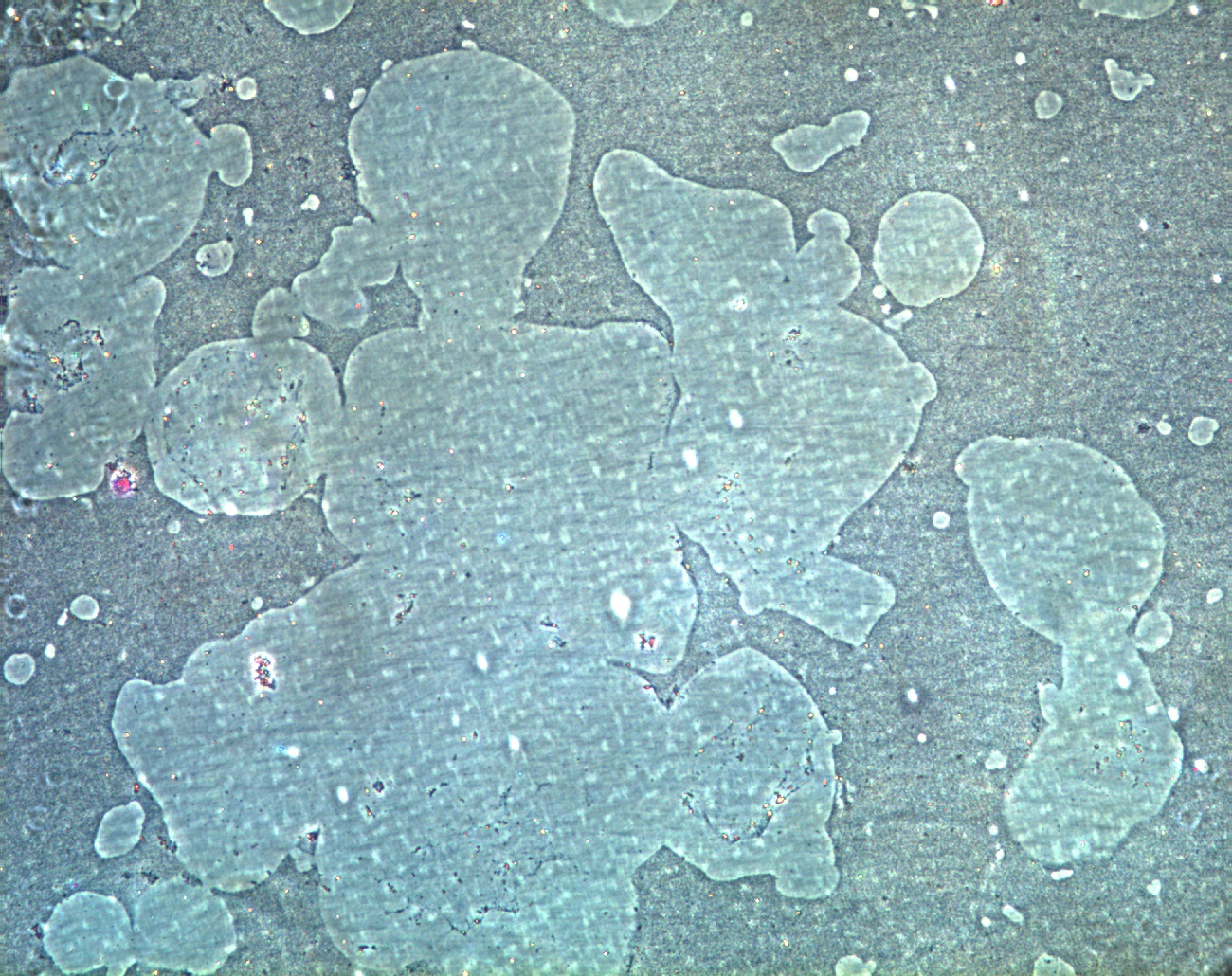

Supplement: Supplementary file 4 [file DataSheet1.ZIP › Fig.1-Source data/F/RANKL.jpg]

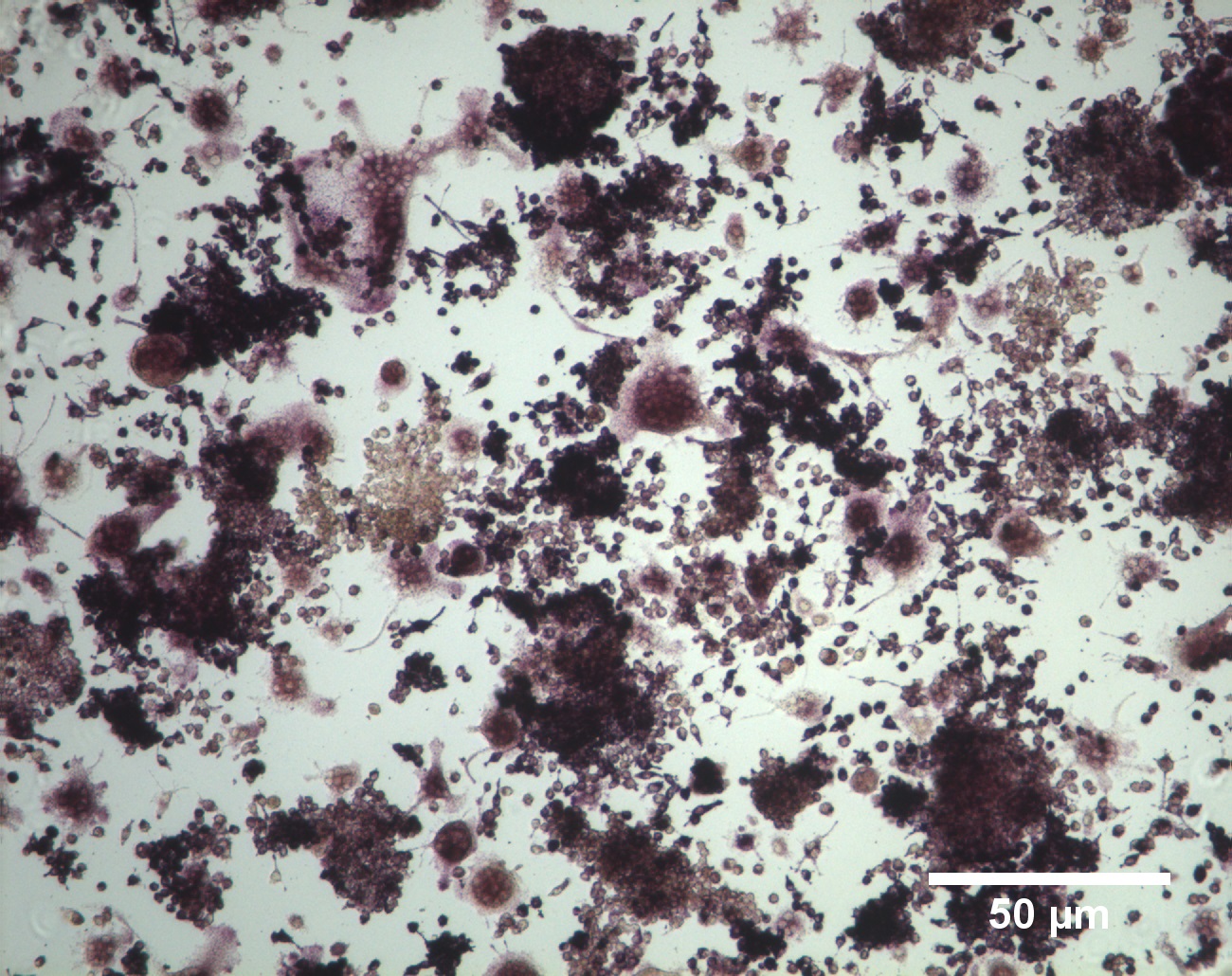

Supplement: Supplementary file 4 [file DataSheet1.ZIP › Fig.1-Source data/F/TRAP-RANKL+CQ (10 uM).jpg]

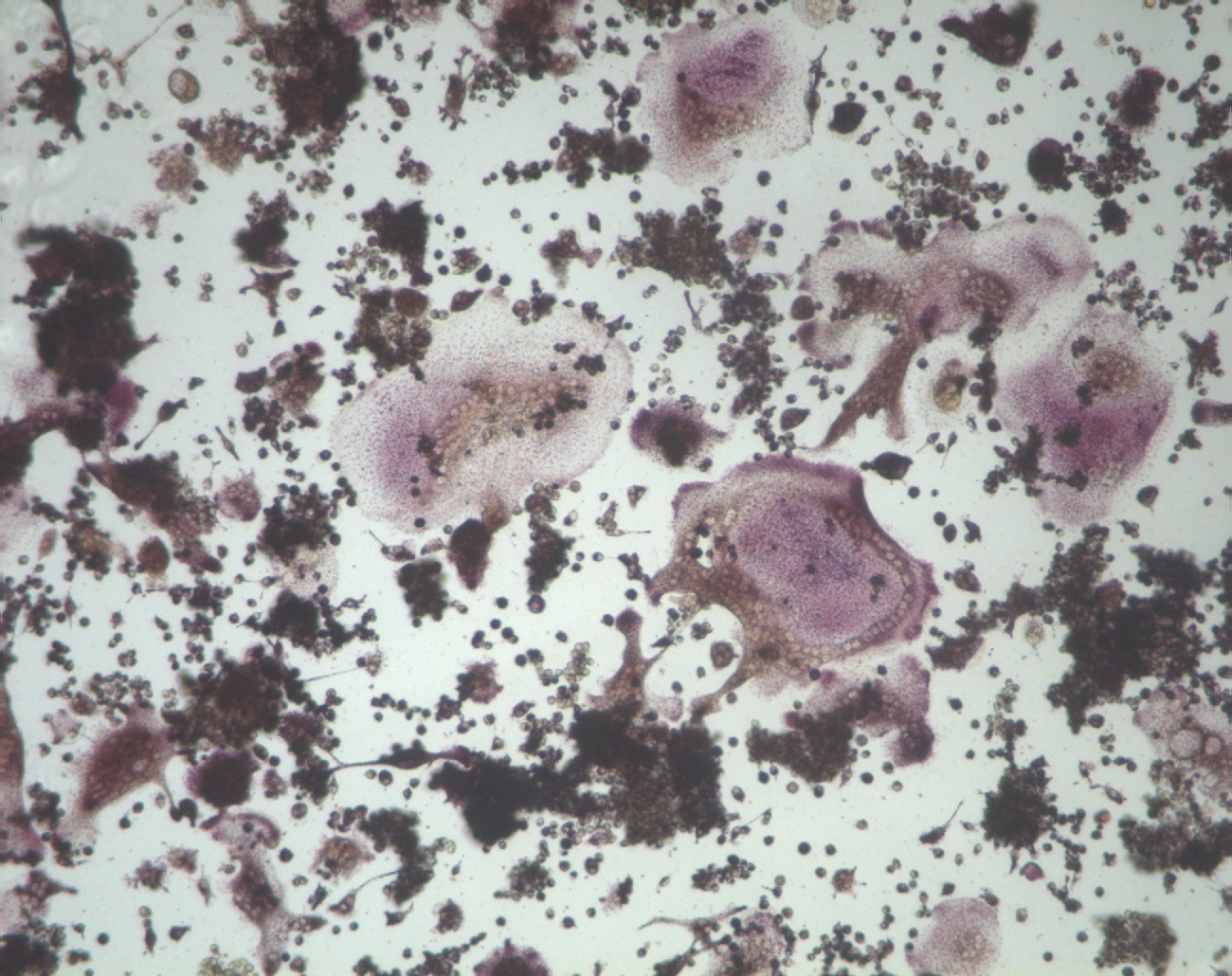

Supplement: Supplementary file 4 [file DataSheet1.ZIP › Fig.1-Source data/F/TRAP-RANKL+CQ (5 uM).jpg]

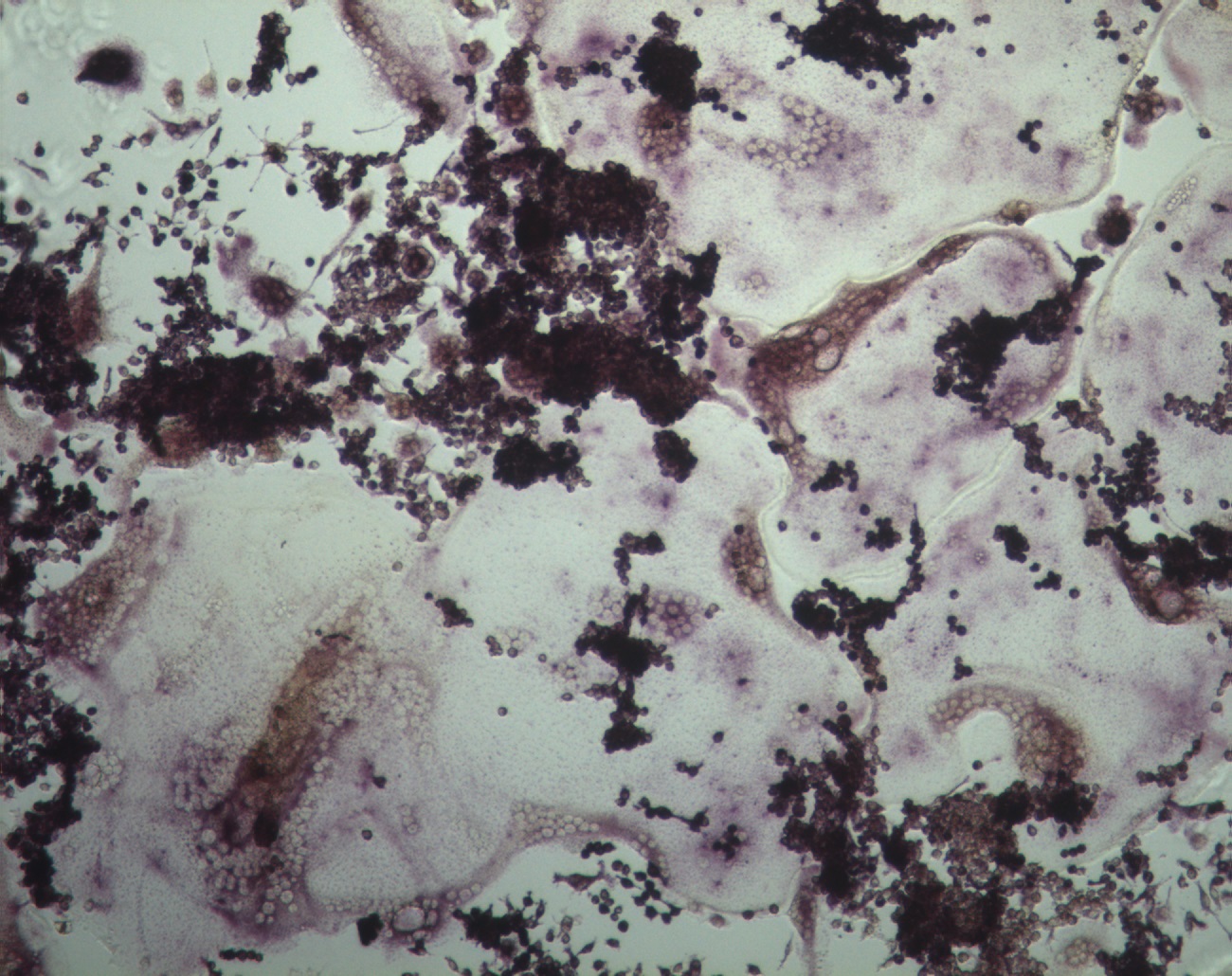

Supplement: Supplementary file 4 [file DataSheet1.ZIP › Fig.1-Source data/F/TRAP-RANKL.jpg]

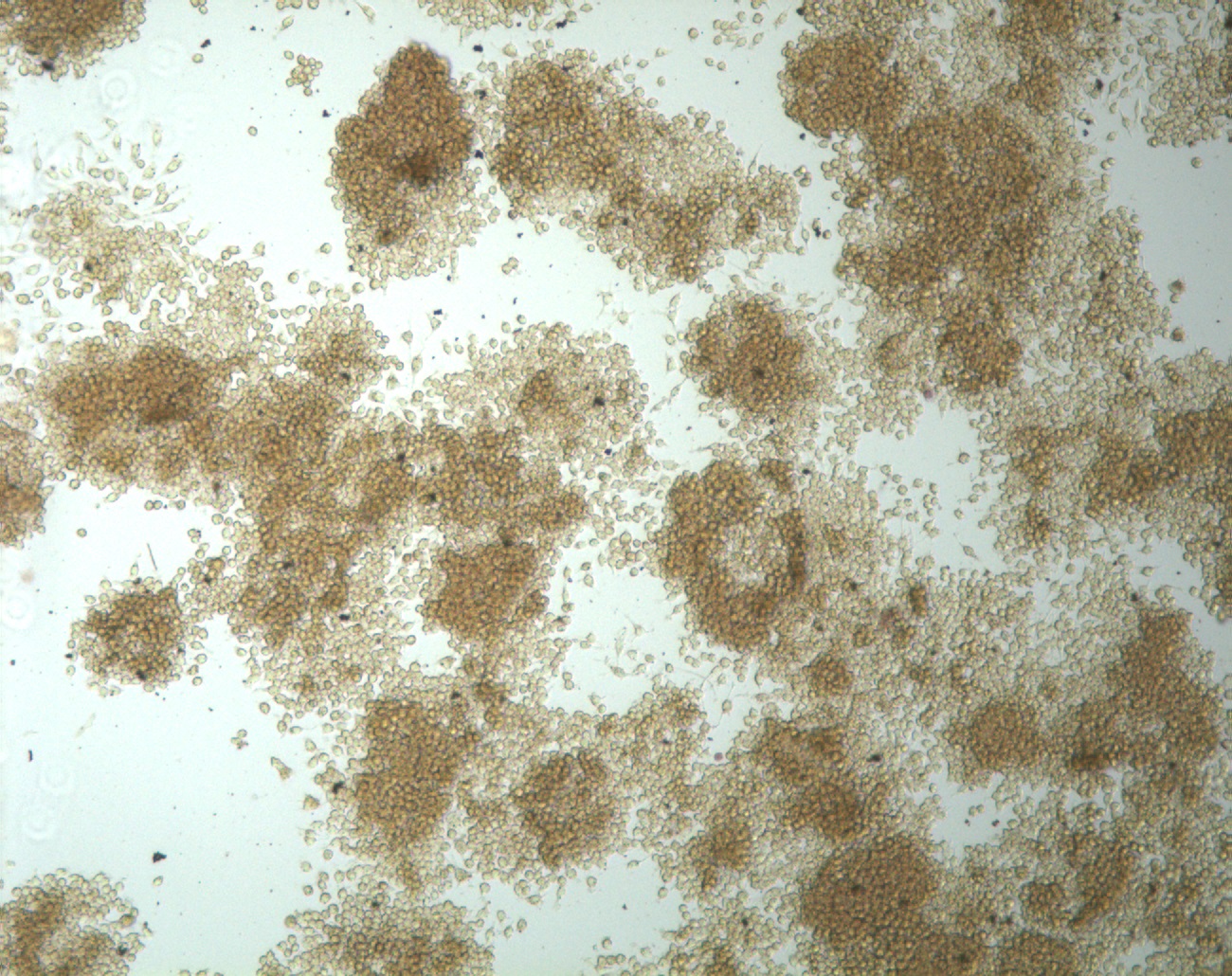

Supplement: Supplementary file 4 [file DataSheet1.ZIP › Fig.1-Source data/F/TRAP-comtrol.jpg]

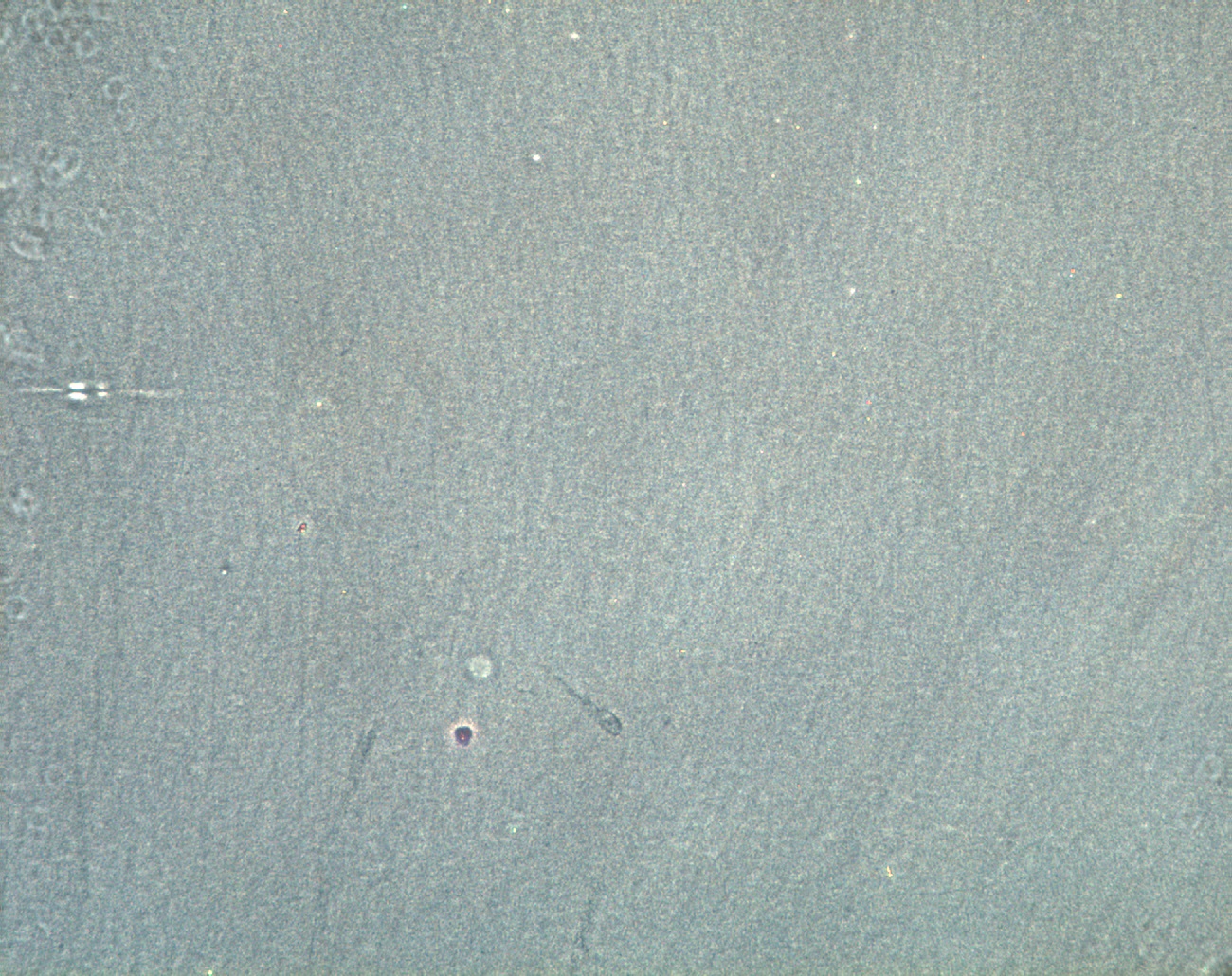

Supplement: Supplementary file 4 [file DataSheet1.ZIP › Fig.1-Source data/F/comtrol.jpg]

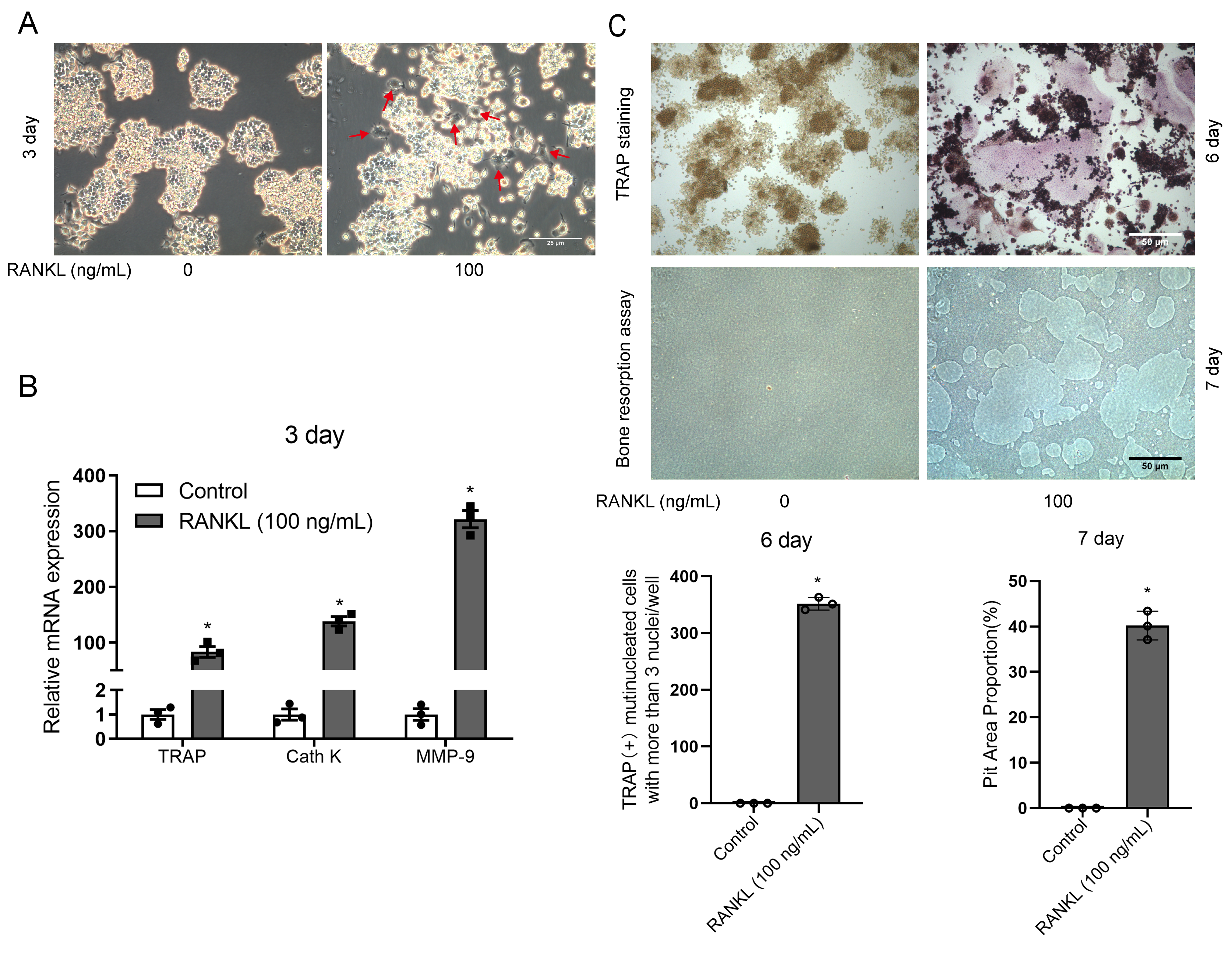

Supplement: Supplementary file 5 [file Image1.TIF]

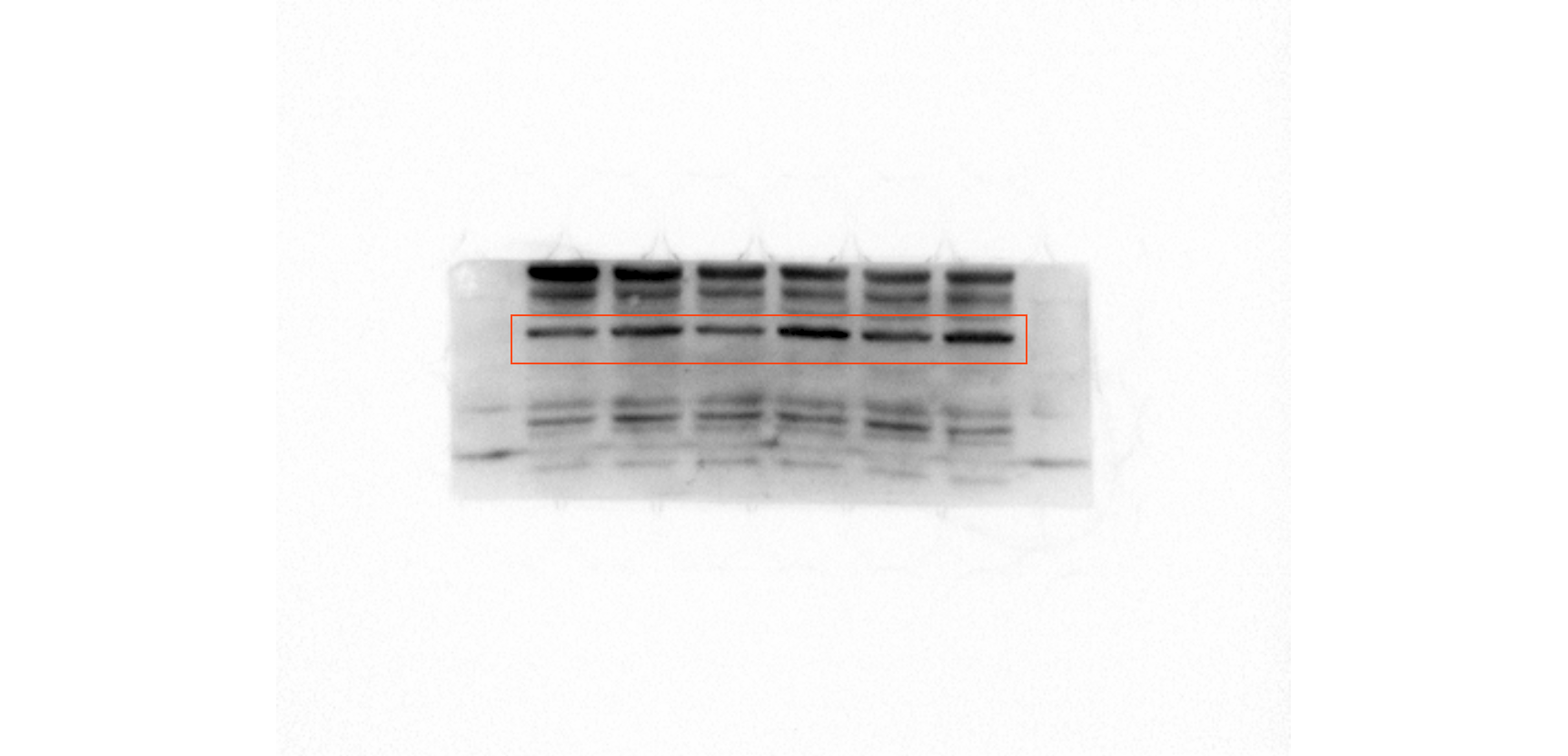

Supplement: Supplementary file 6 [file DataSheet6.ZIP › Fig.6-Source data/A/ATF4.tif]

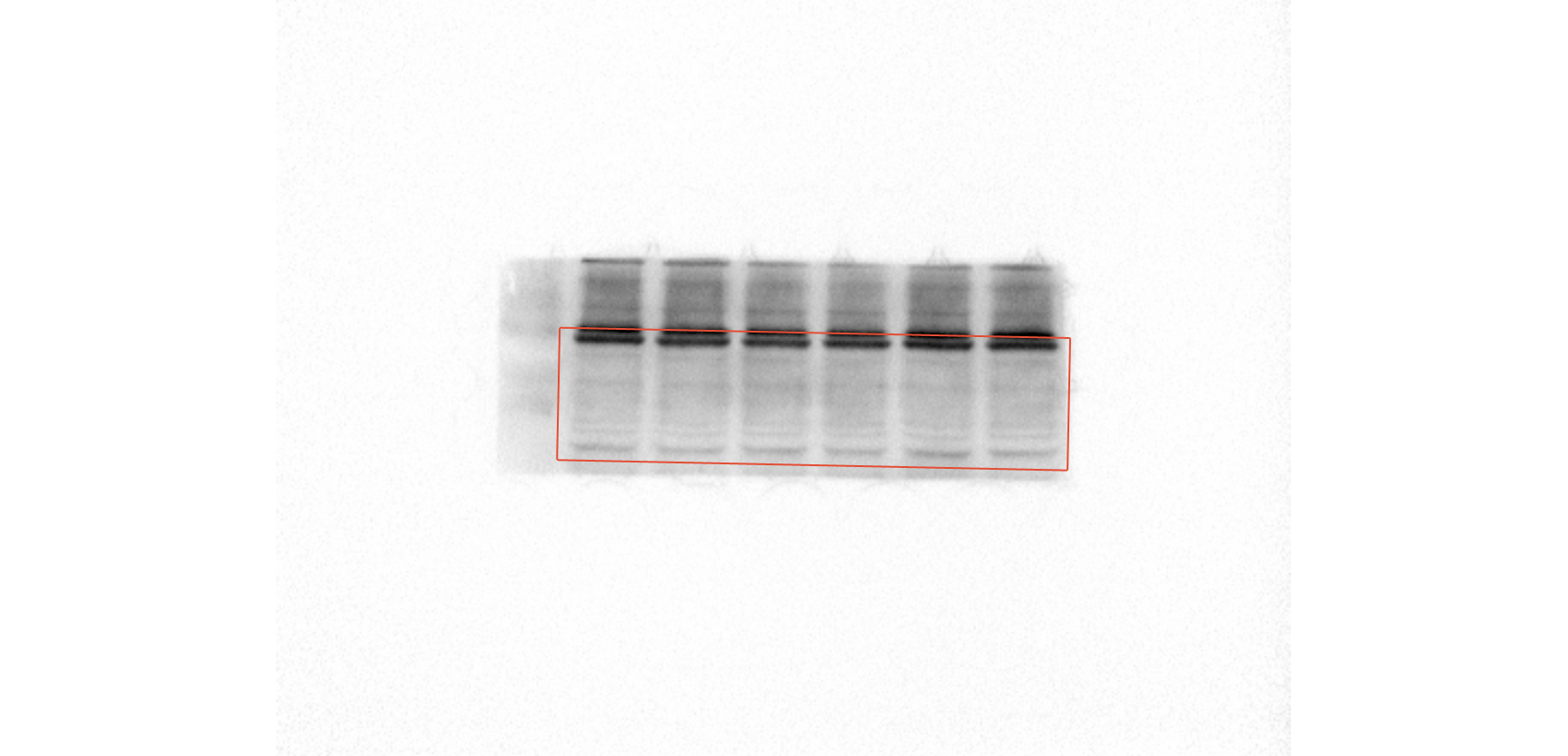

Supplement: Supplementary file 6 [file DataSheet6.ZIP › Fig.6-Source data/A/ATF6.tif]

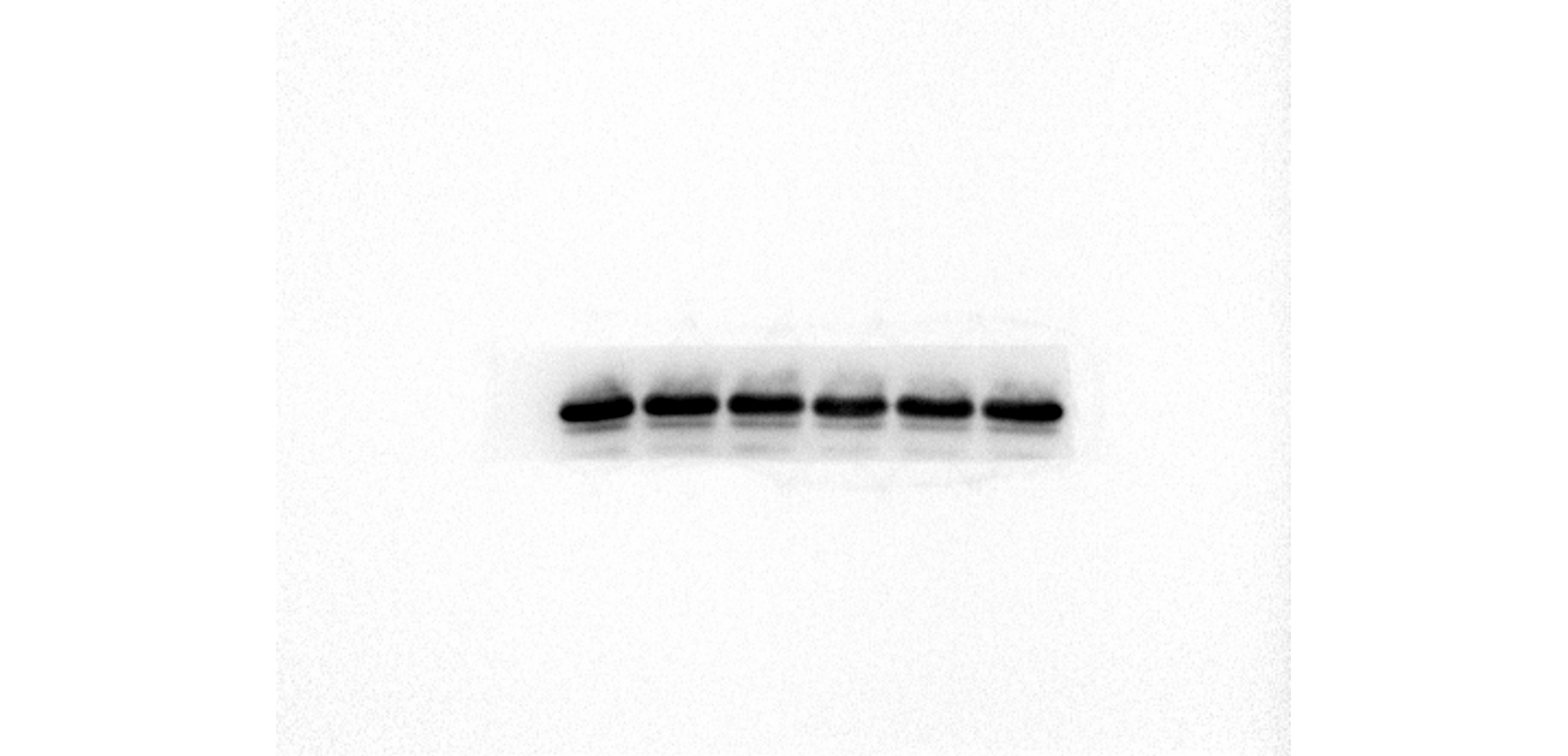

Supplement: Supplementary file 6 [file DataSheet6.ZIP › Fig.6-Source data/A/GAPDH.tif]

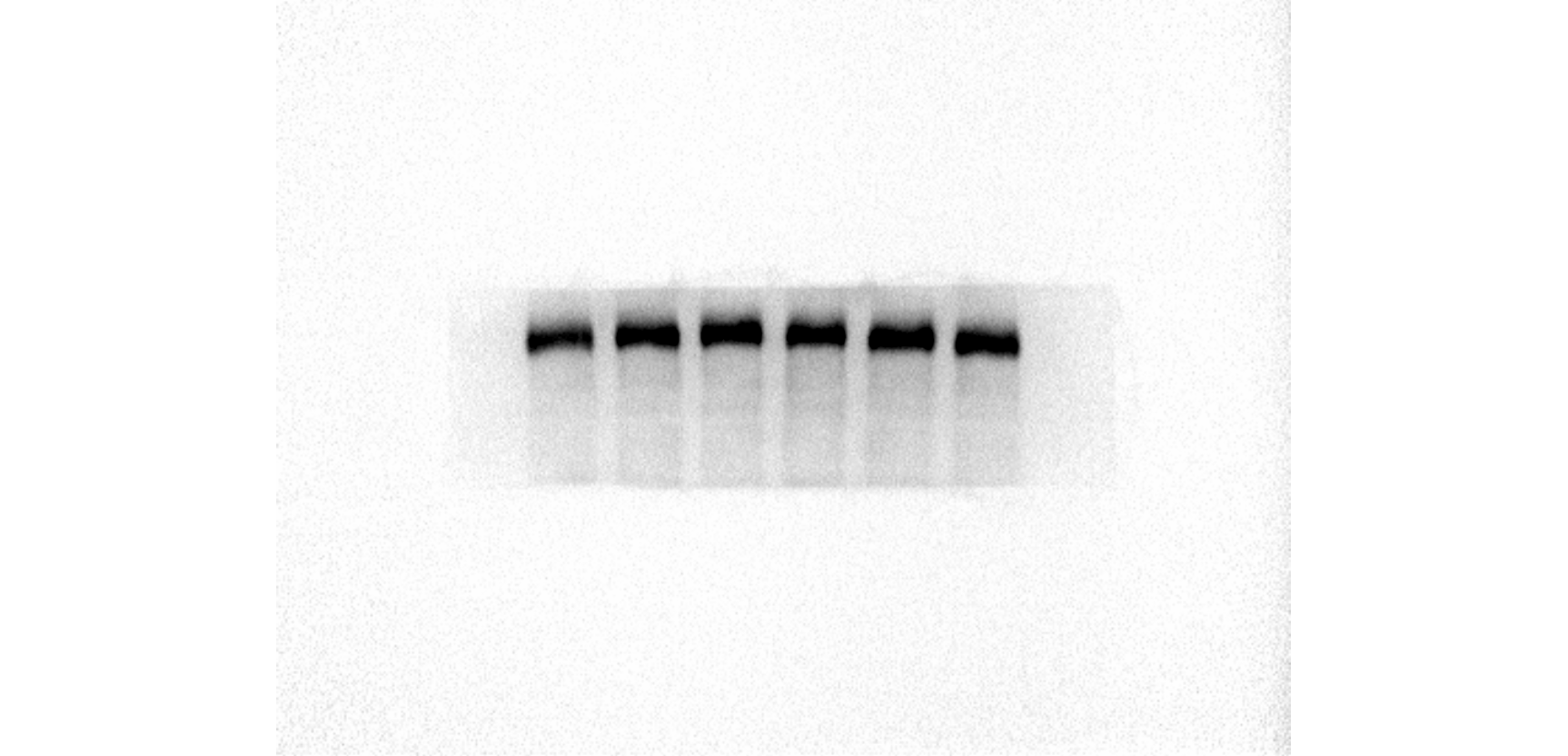

Supplement: Supplementary file 6 [file DataSheet6.ZIP › Fig.6-Source data/A/PERK.tif]

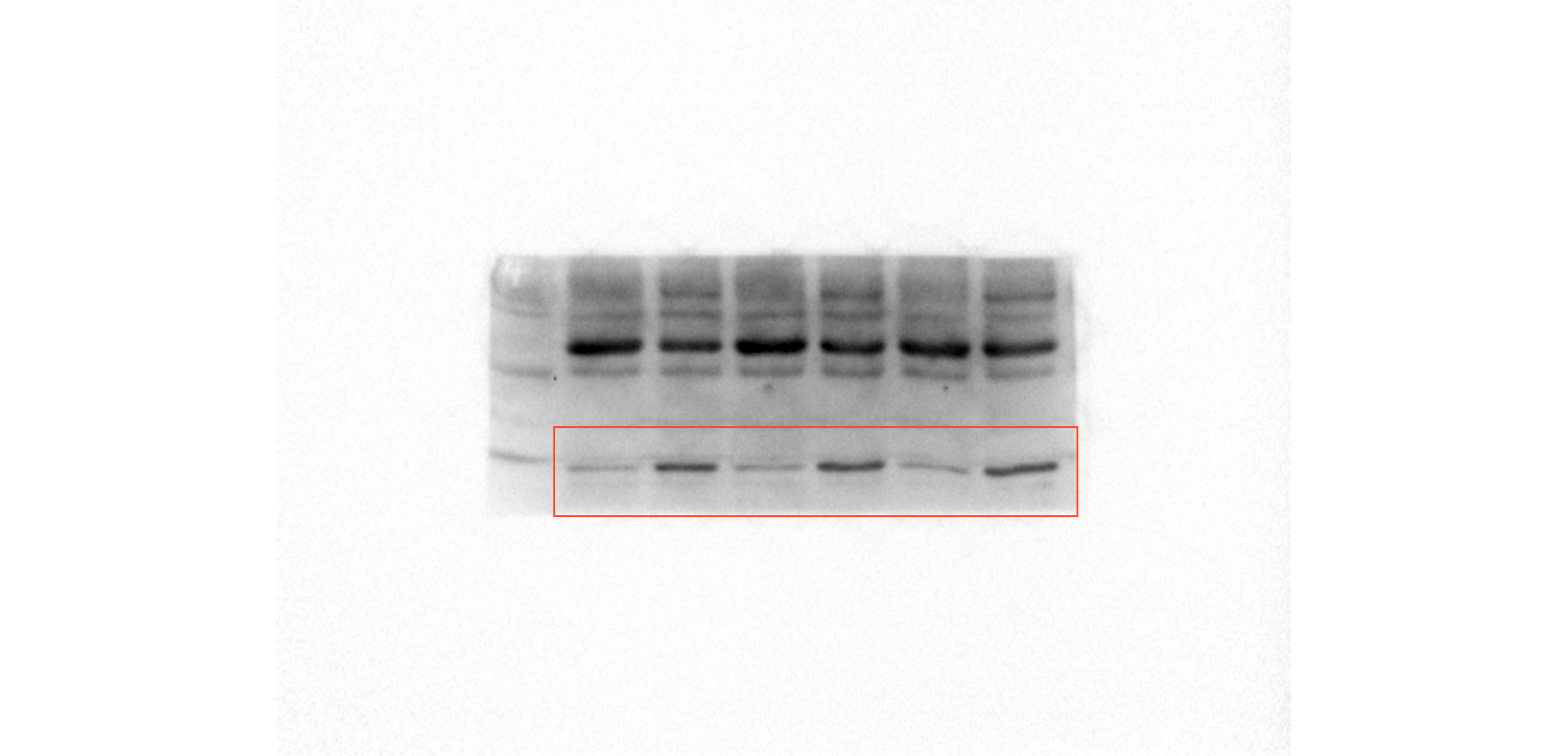

Supplement: Supplementary file 6 [file DataSheet6.ZIP › Fig.6-Source data/A/XBP-1S.tif]

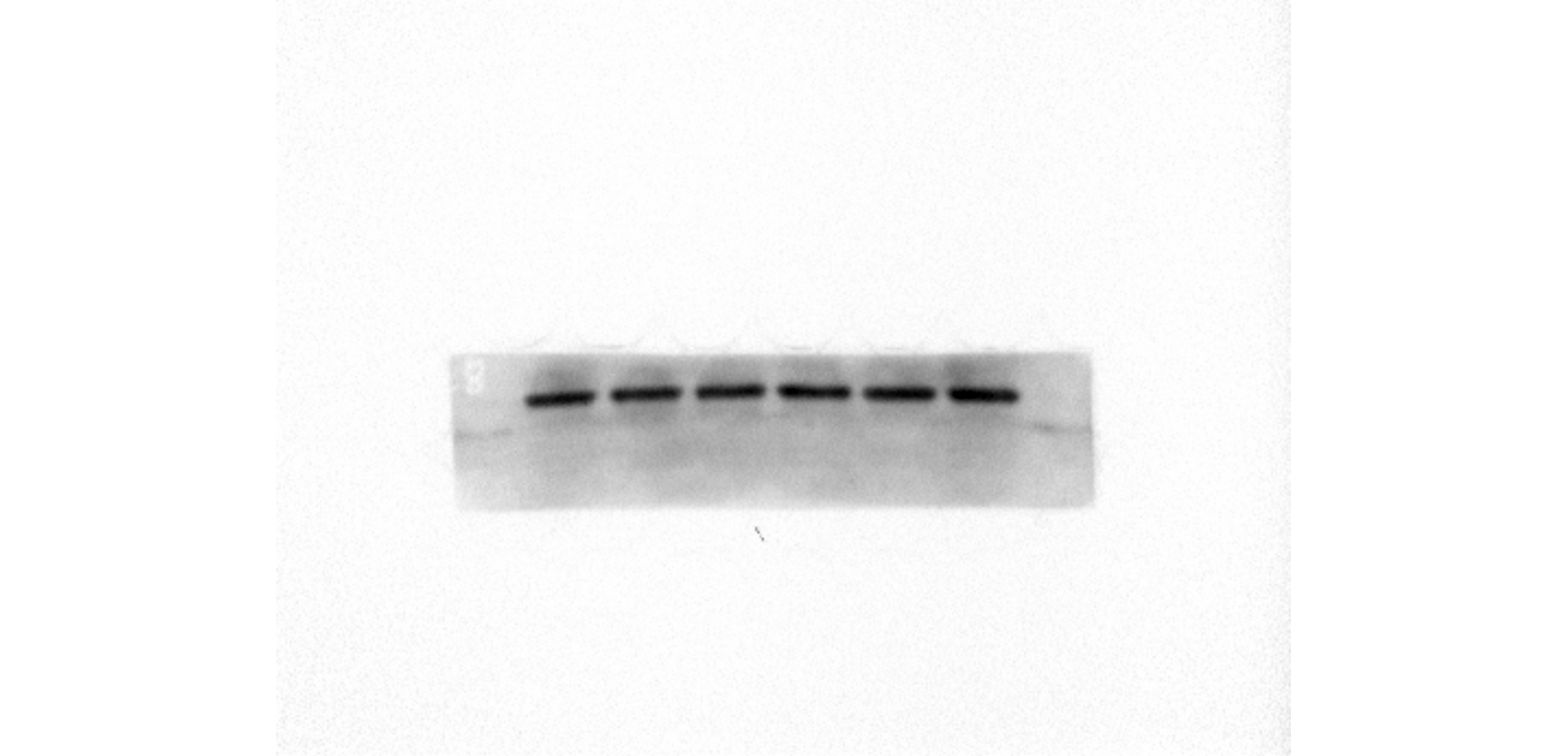

Supplement: Supplementary file 6 [file DataSheet6.ZIP › Fig.6-Source data/A/eIF-2a.tif]

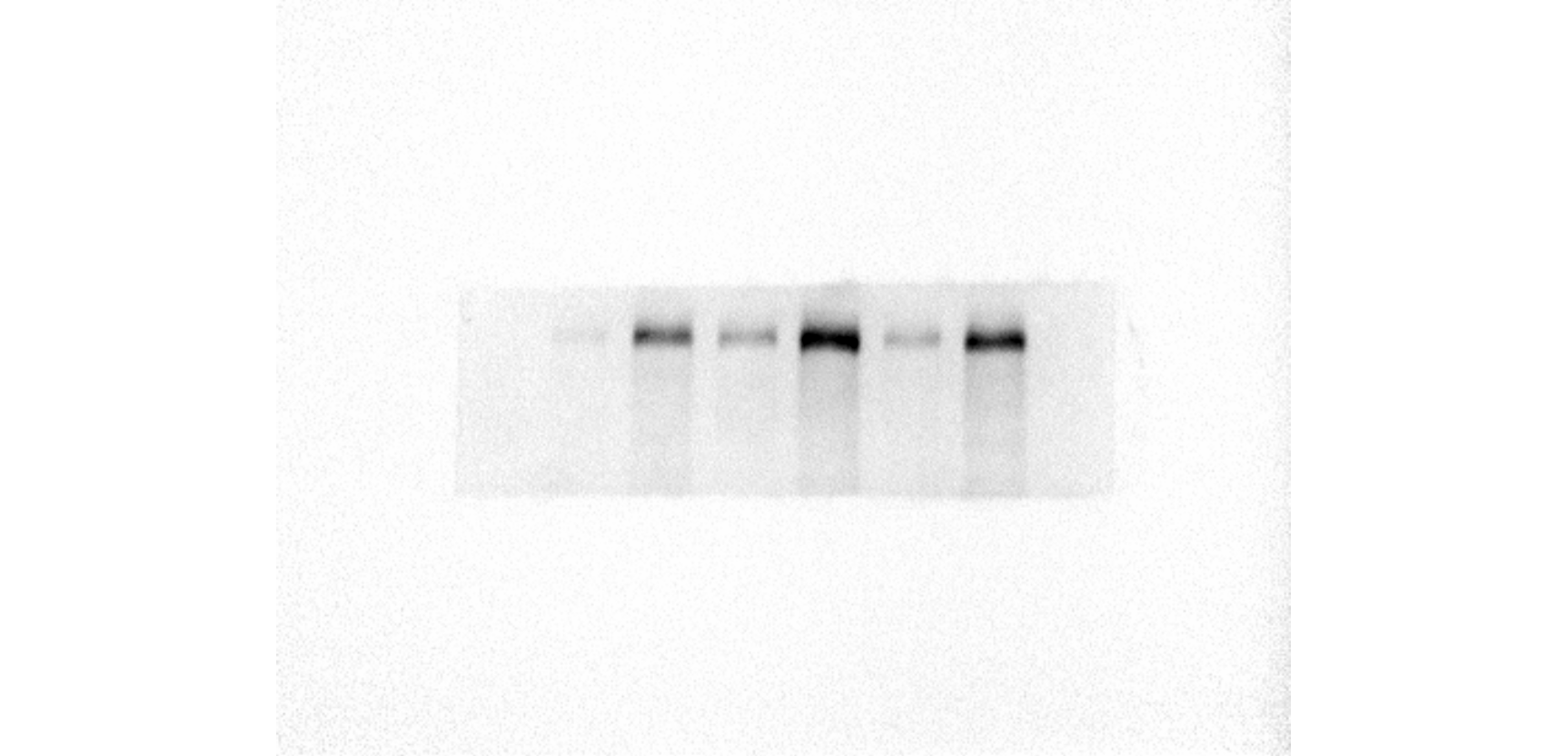

Supplement: Supplementary file 6 [file DataSheet6.ZIP › Fig.6-Source data/A/p-PERK.tif]

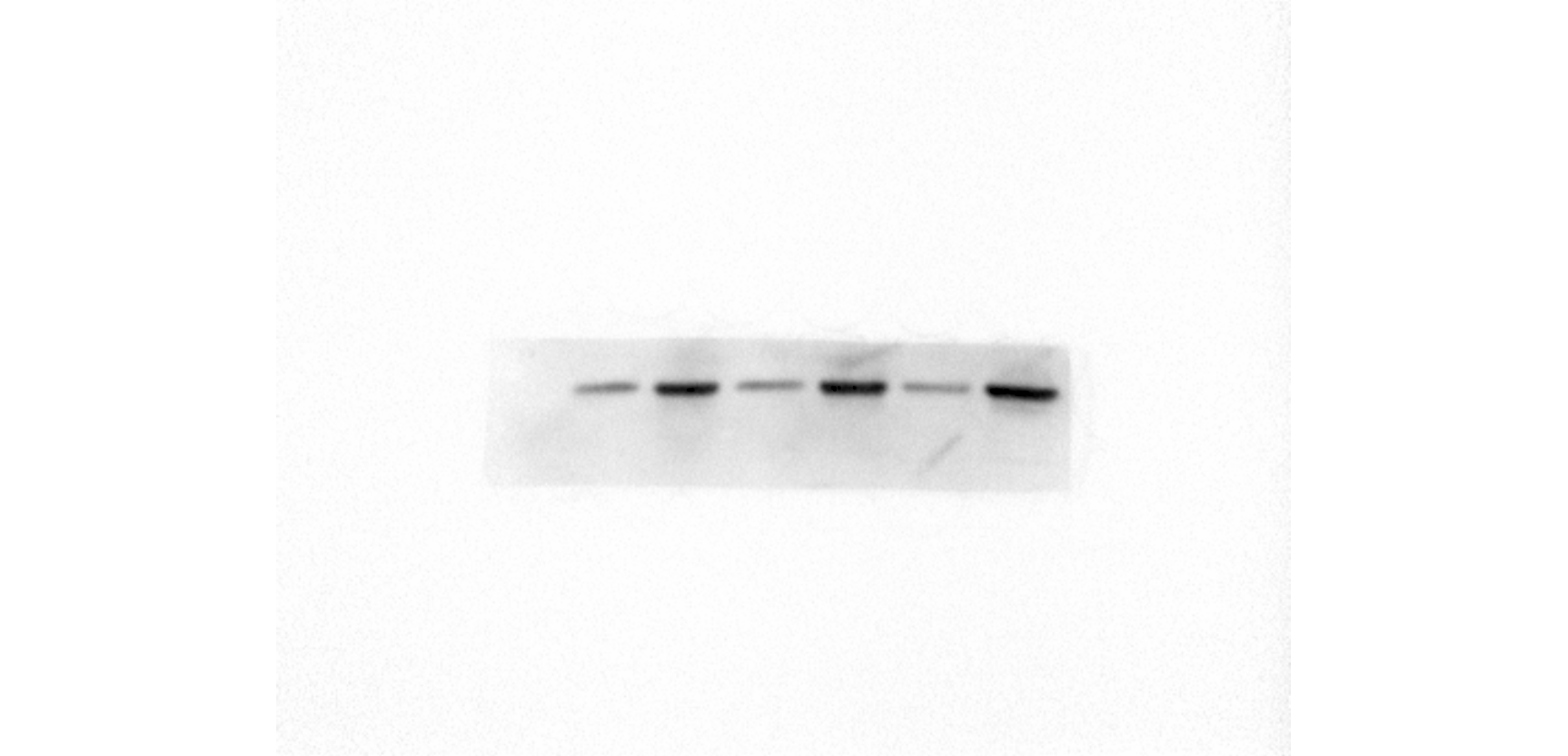

Supplement: Supplementary file 6 [file DataSheet6.ZIP › Fig.6-Source data/A/p-eIF-2a.tif]

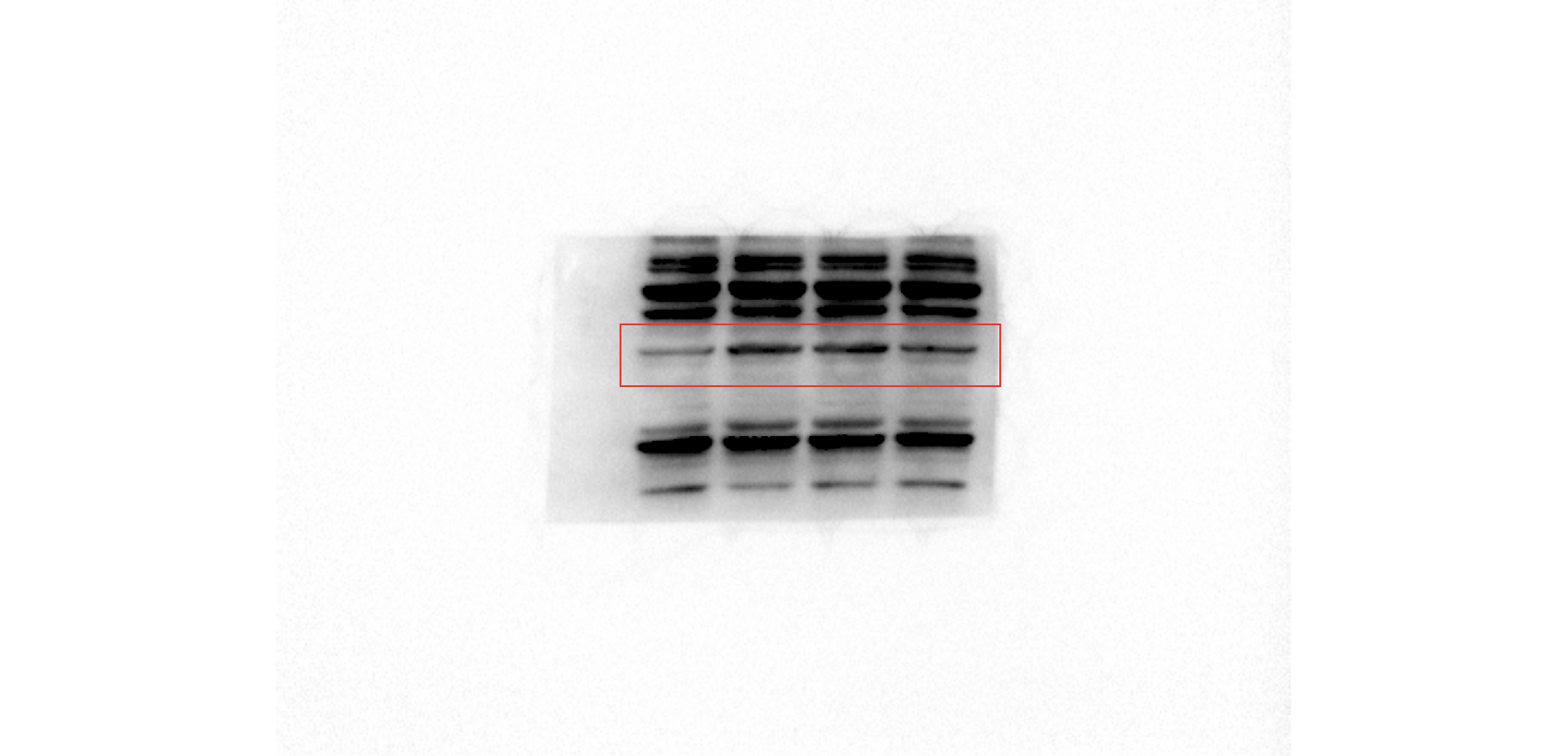

Supplement: Supplementary file 6 [file DataSheet6.ZIP › Fig.6-Source data/B/ATF4.tif]

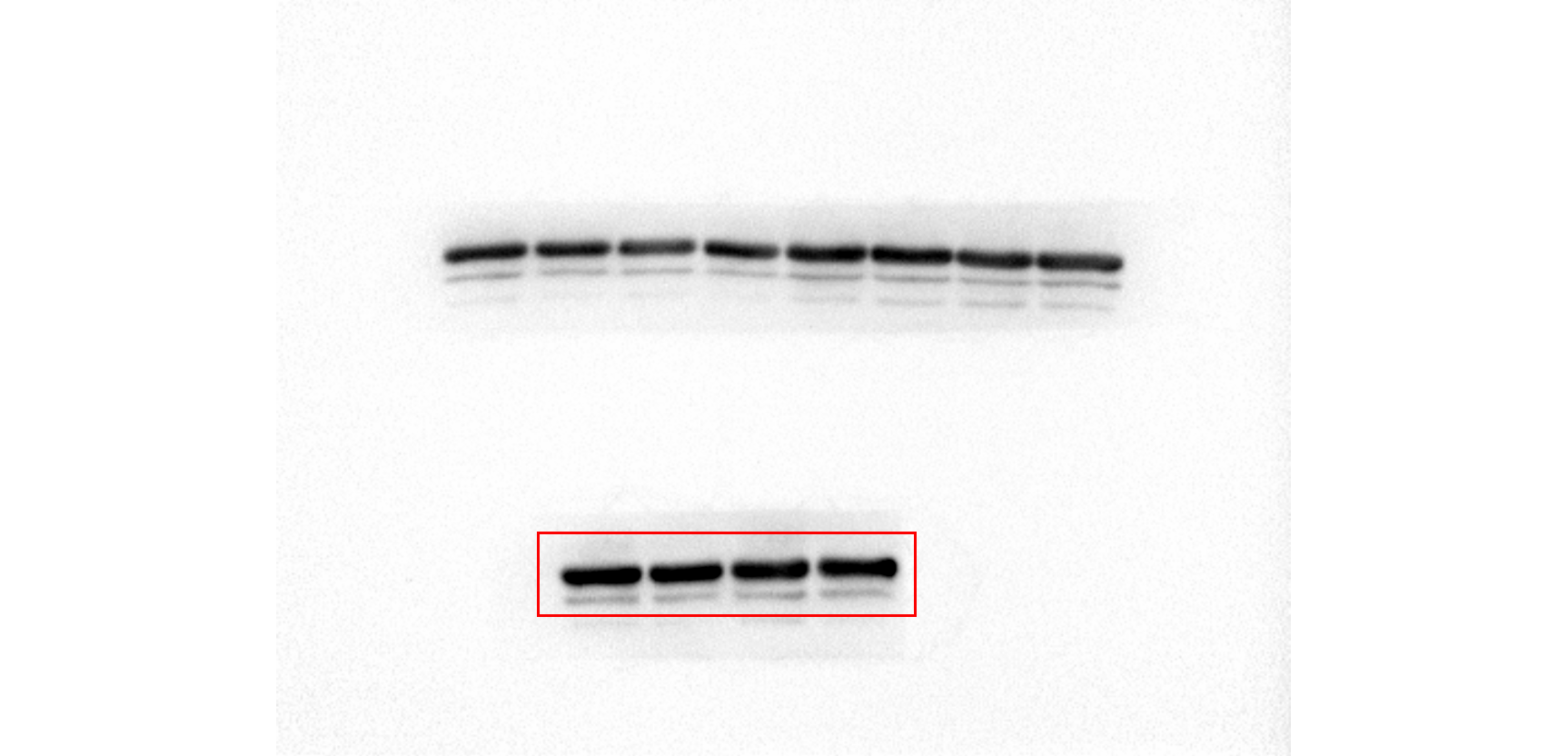

Supplement: Supplementary file 6 [file DataSheet6.ZIP › Fig.6-Source data/B/GAPDH.tif]

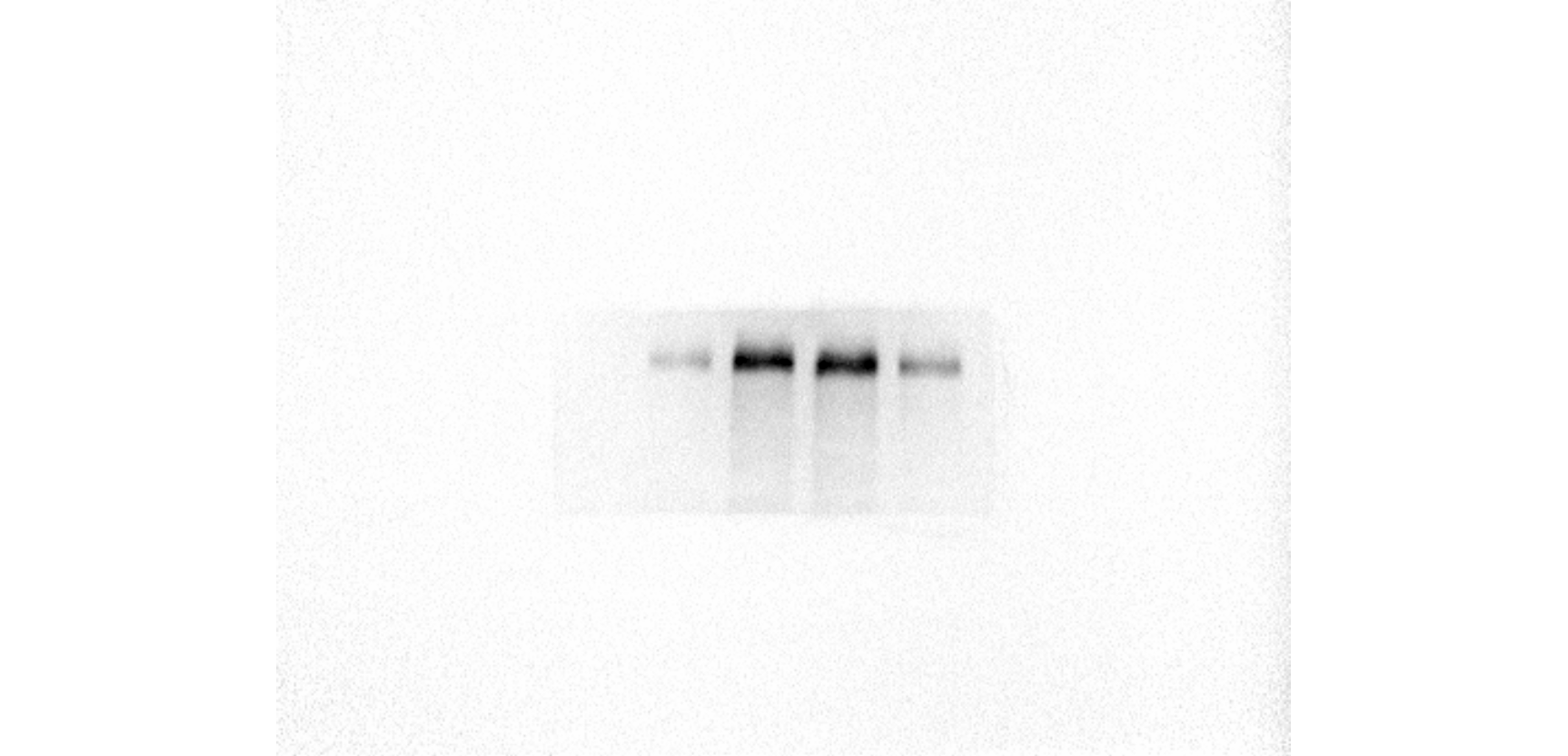

Supplement: Supplementary file 6 [file DataSheet6.ZIP › Fig.6-Source data/B/P-PERK.tif]

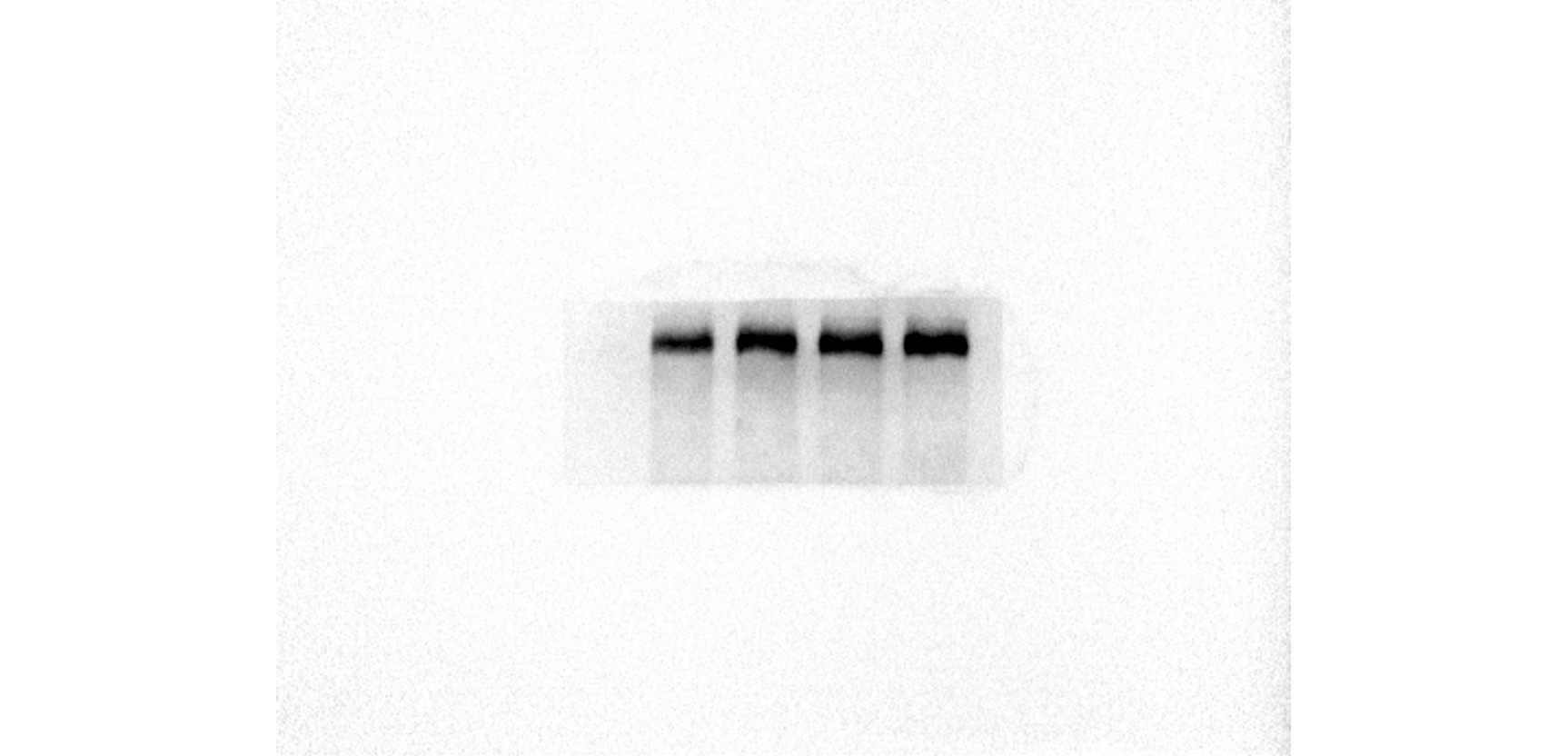

Supplement: Supplementary file 6 [file DataSheet6.ZIP › Fig.6-Source data/B/PERK.tif]

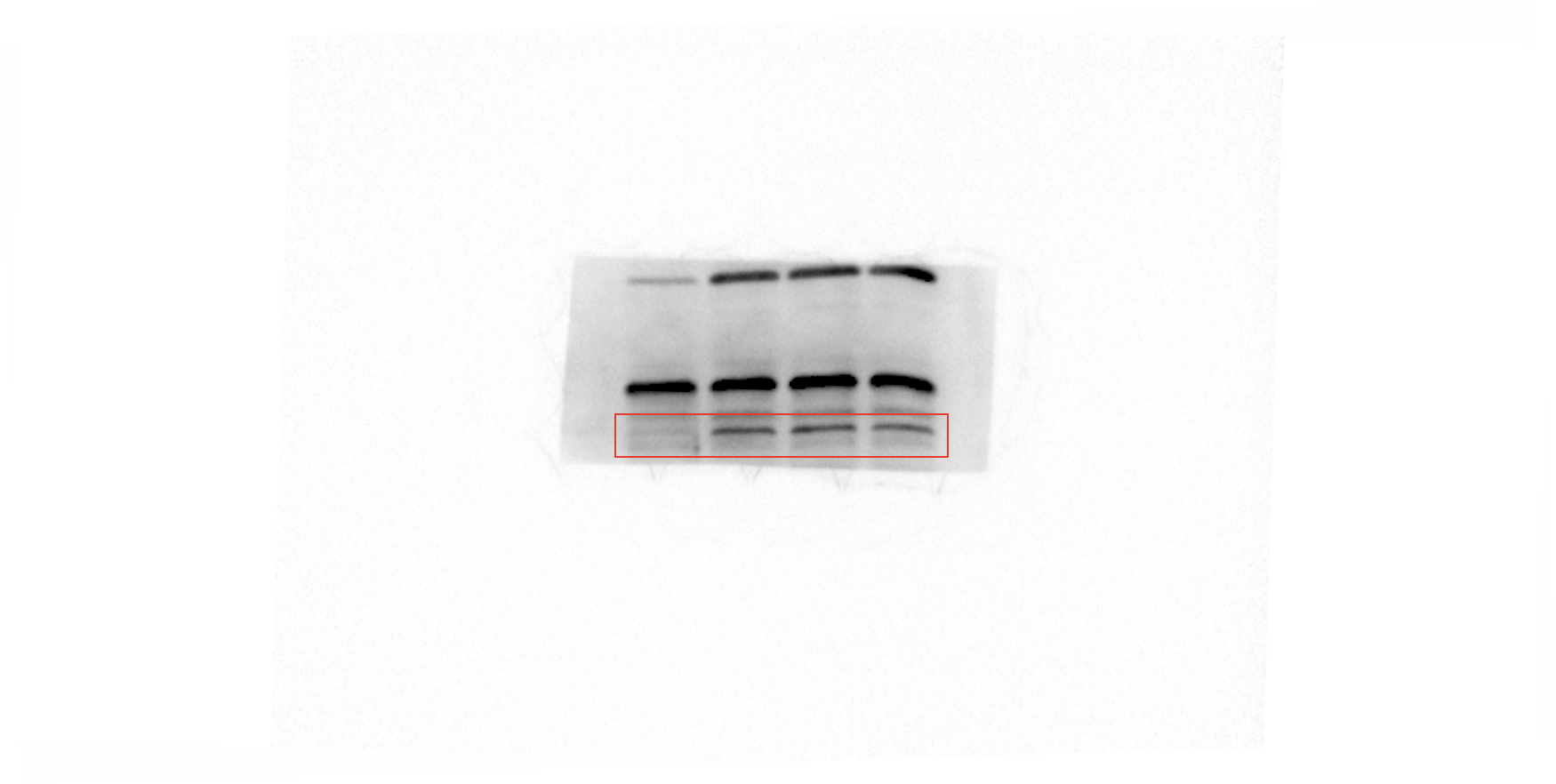

Supplement: Supplementary file 6 [file DataSheet6.ZIP › Fig.6-Source data/B/XBP-1s.tif]

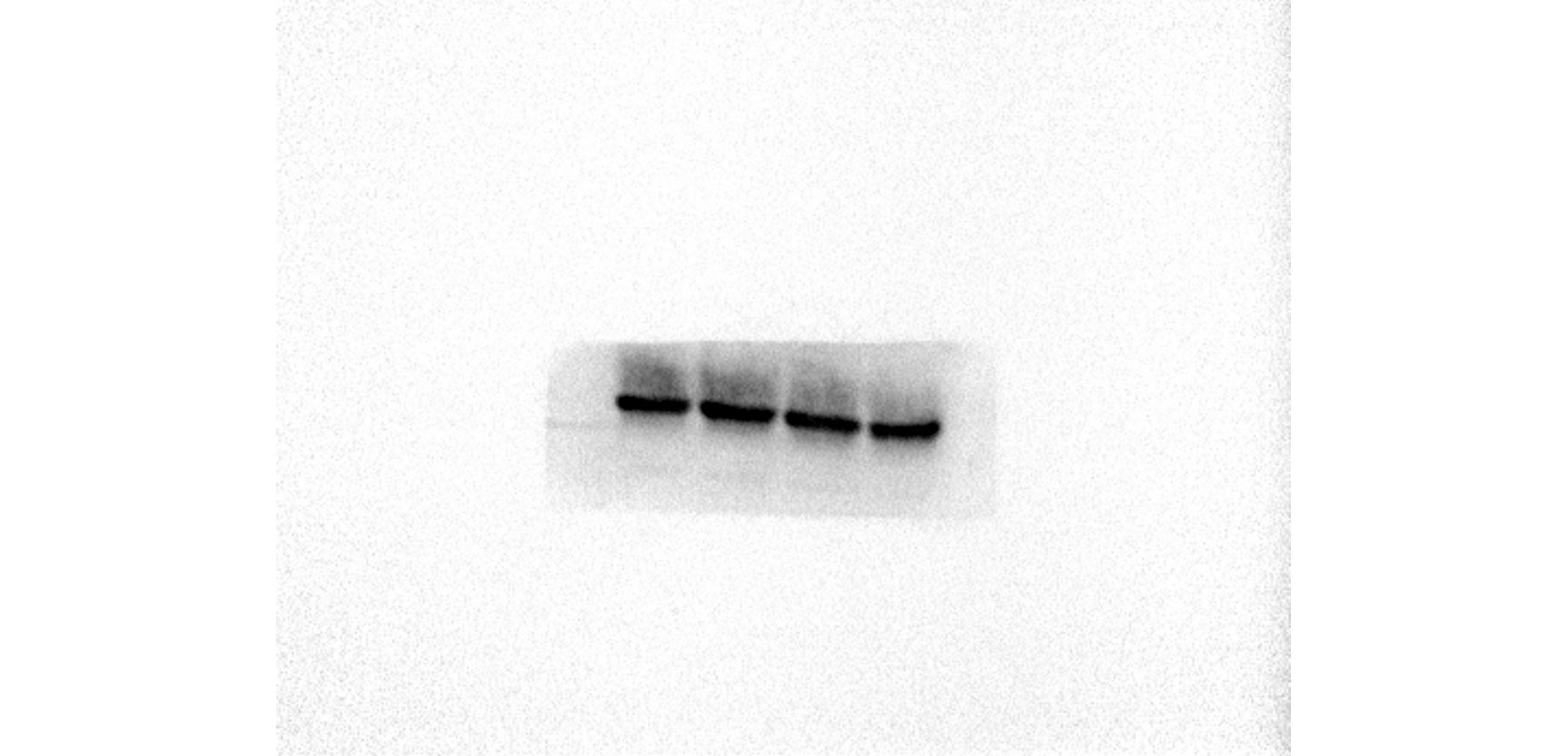

Supplement: Supplementary file 6 [file DataSheet6.ZIP › Fig.6-Source data/B/eIF2a.tif]

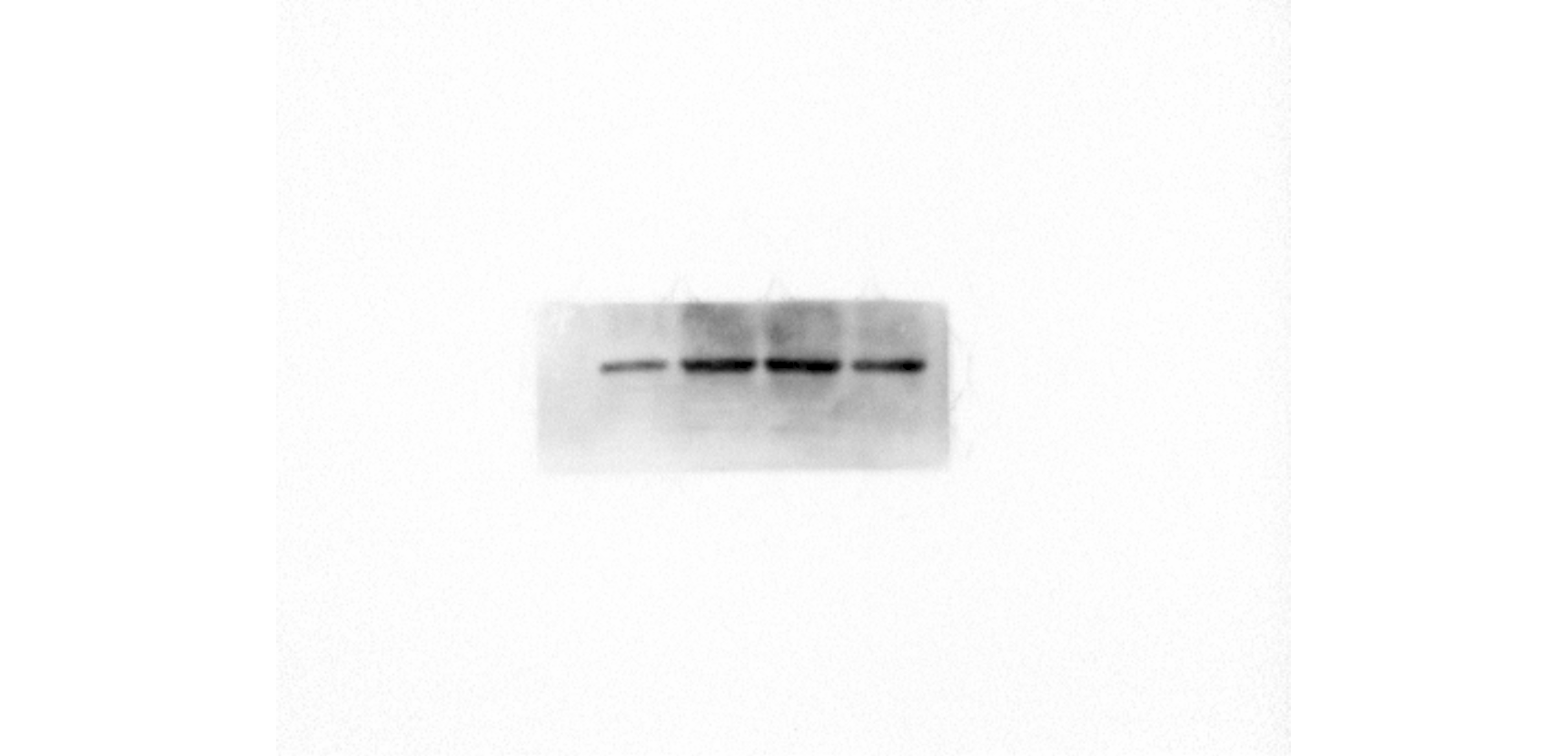

Supplement: Supplementary file 6 [file DataSheet6.ZIP › Fig.6-Source data/B/p-eIF-2a.tif]

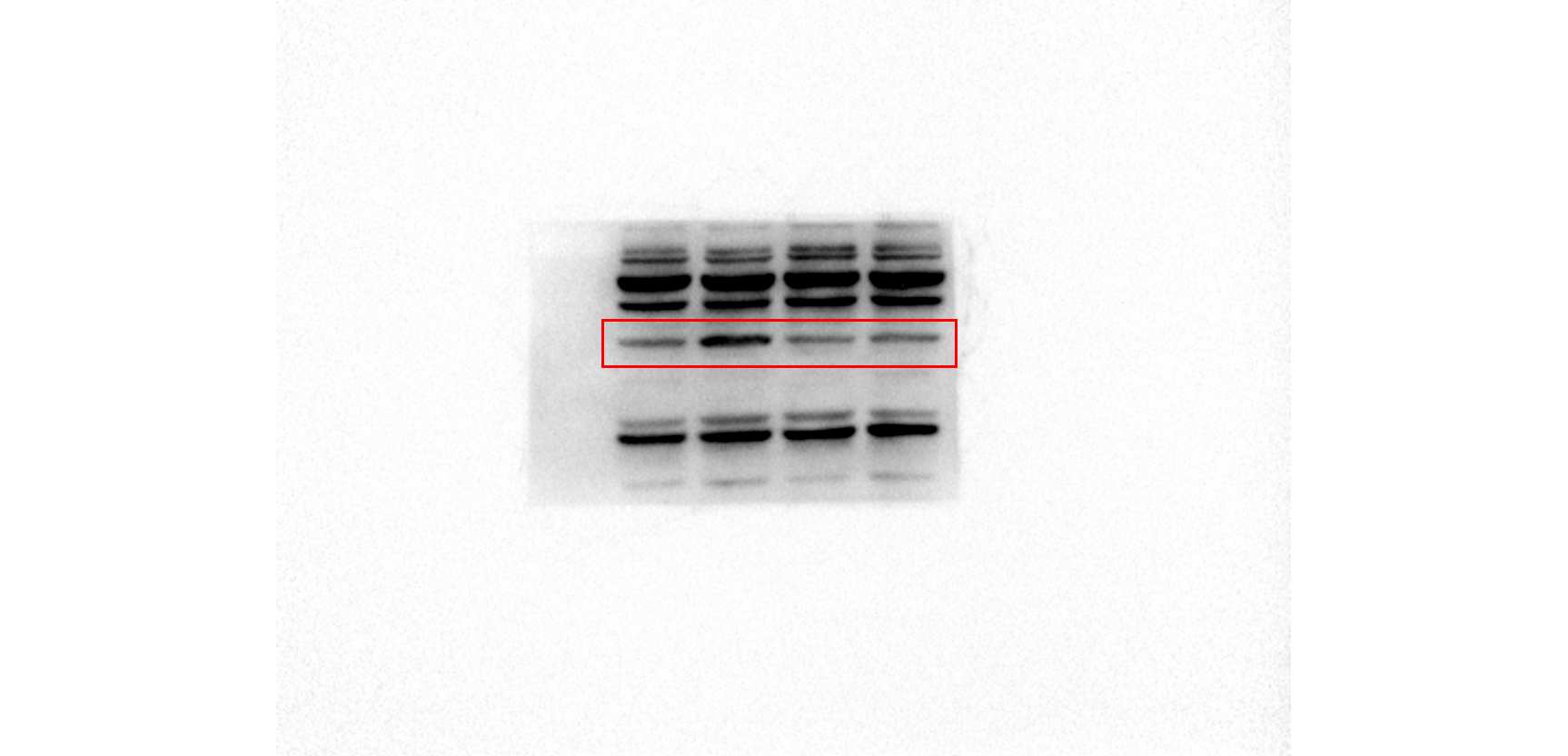

Supplement: Supplementary file 6 [file DataSheet6.ZIP › Fig.6-Source data/C/ATF4.tif]

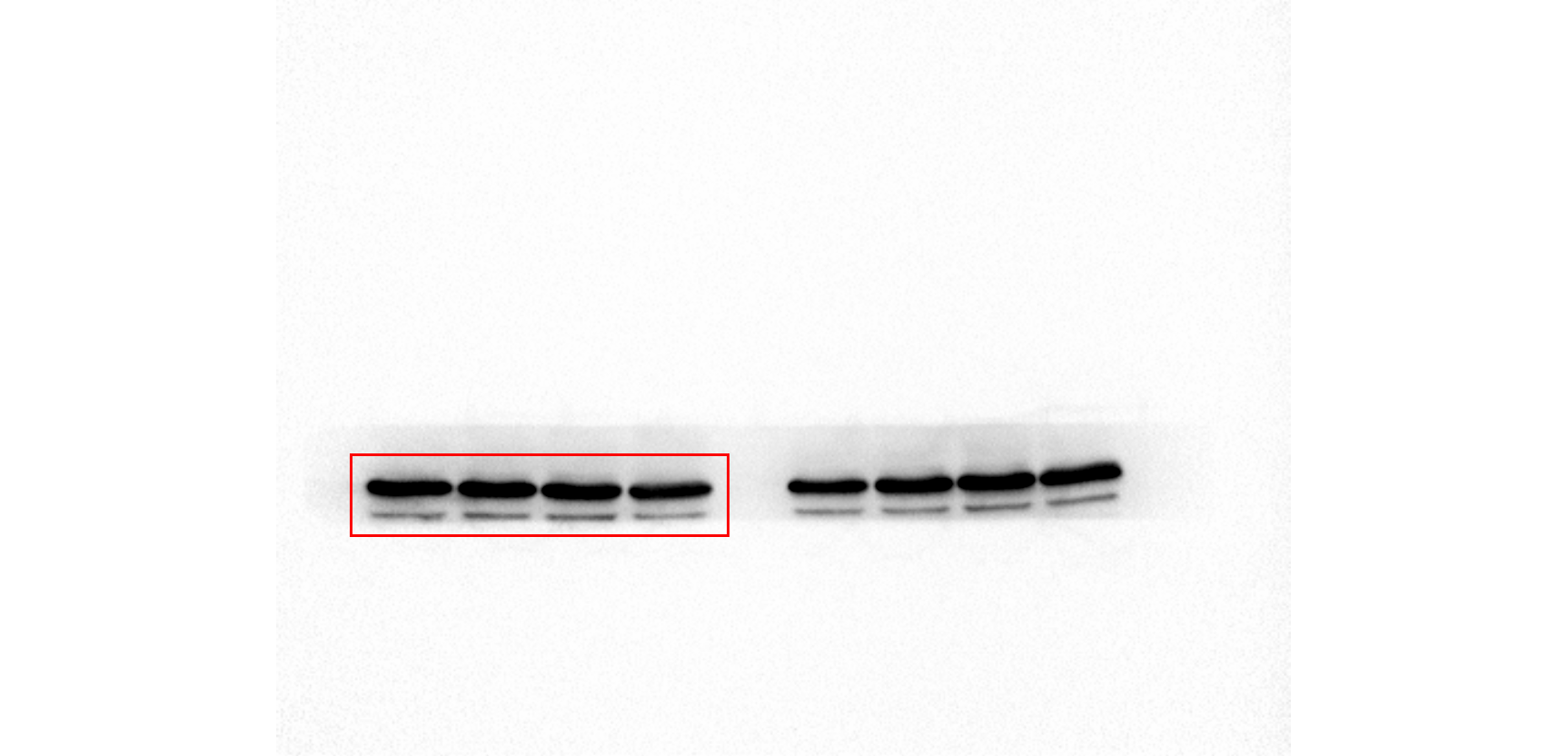

Supplement: Supplementary file 6 [file DataSheet6.ZIP › Fig.6-Source data/C/GAPDH.tif]

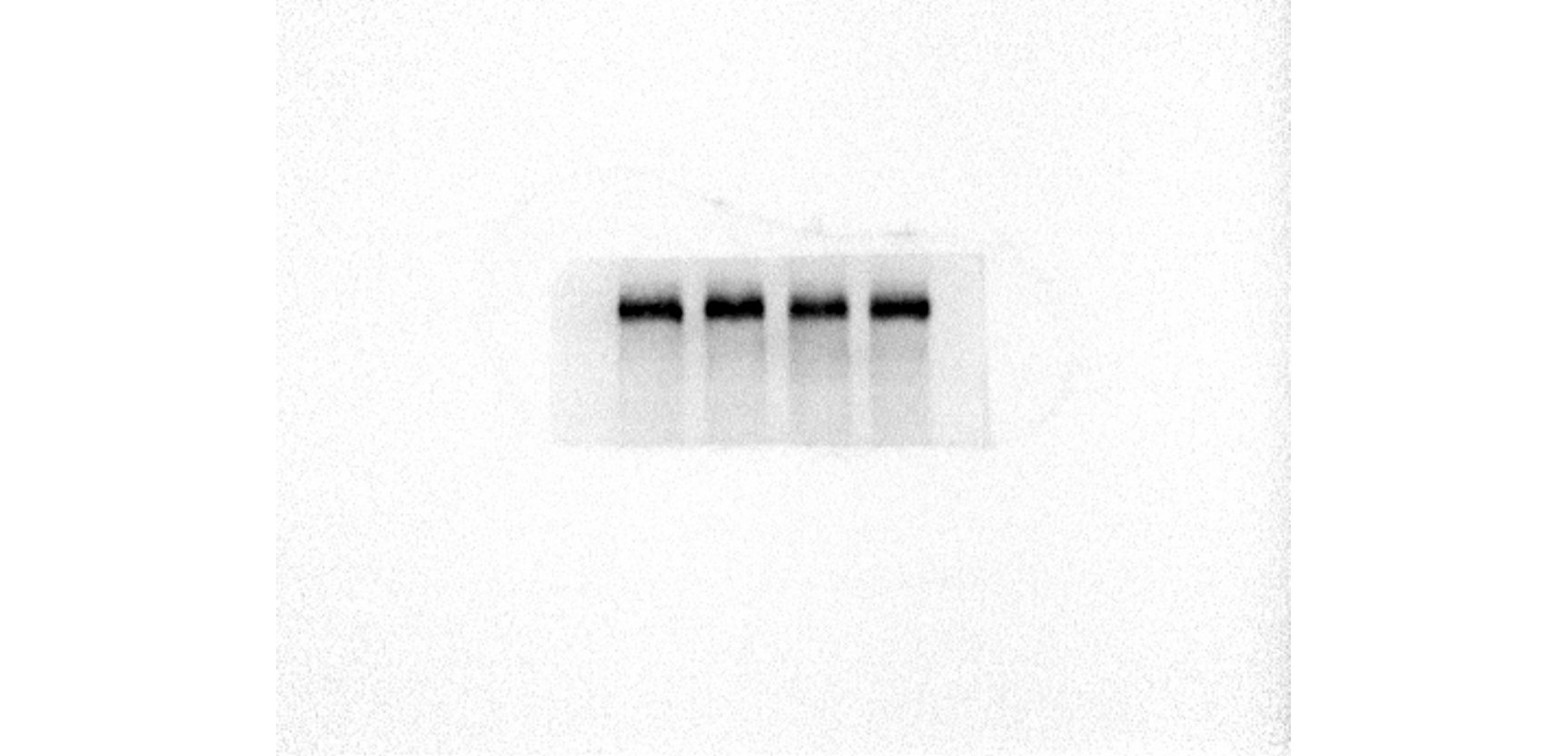

Supplement: Supplementary file 6 [file DataSheet6.ZIP › Fig.6-Source data/C/PERK.tif]

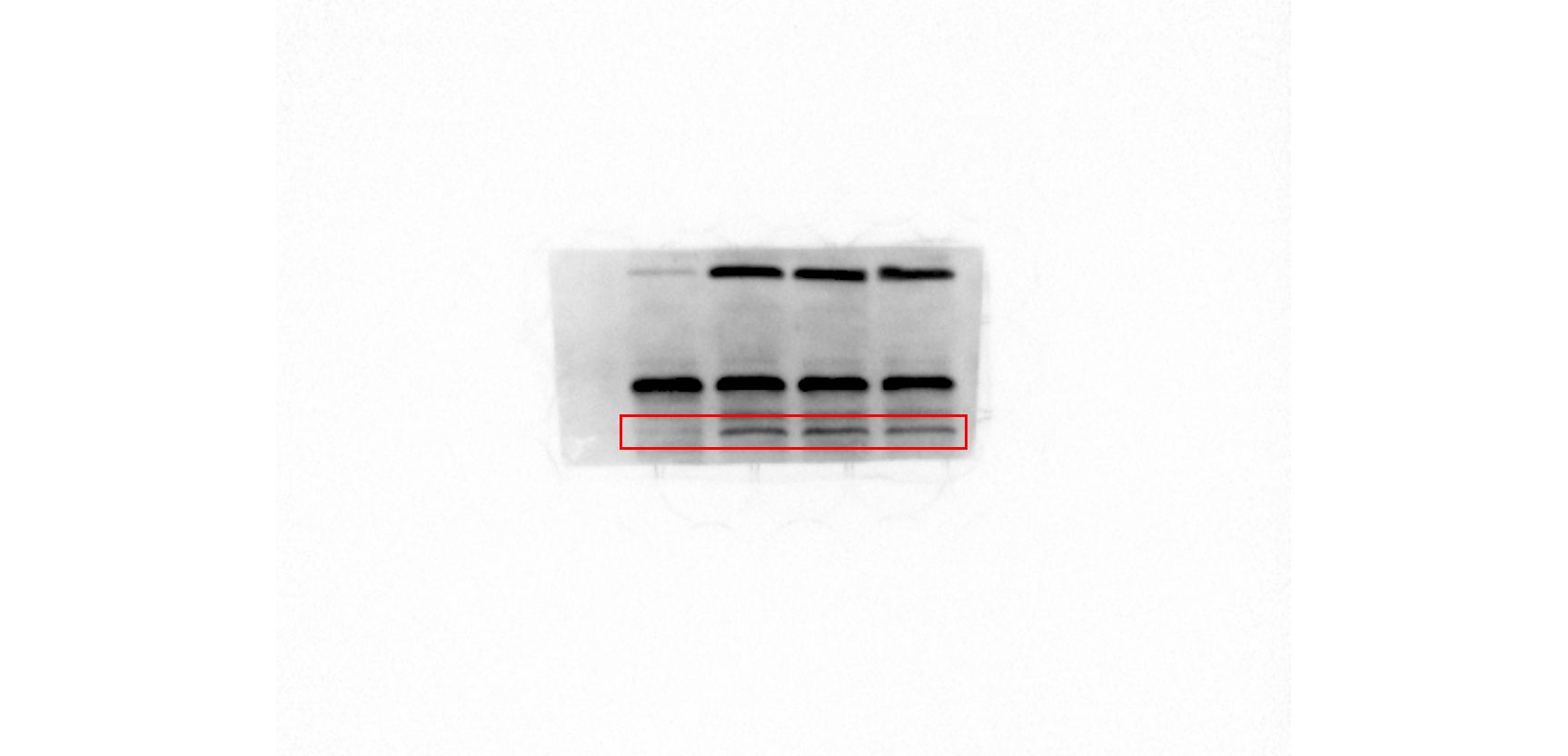

Supplement: Supplementary file 6 [file DataSheet6.ZIP › Fig.6-Source data/C/XBP1s.tif]

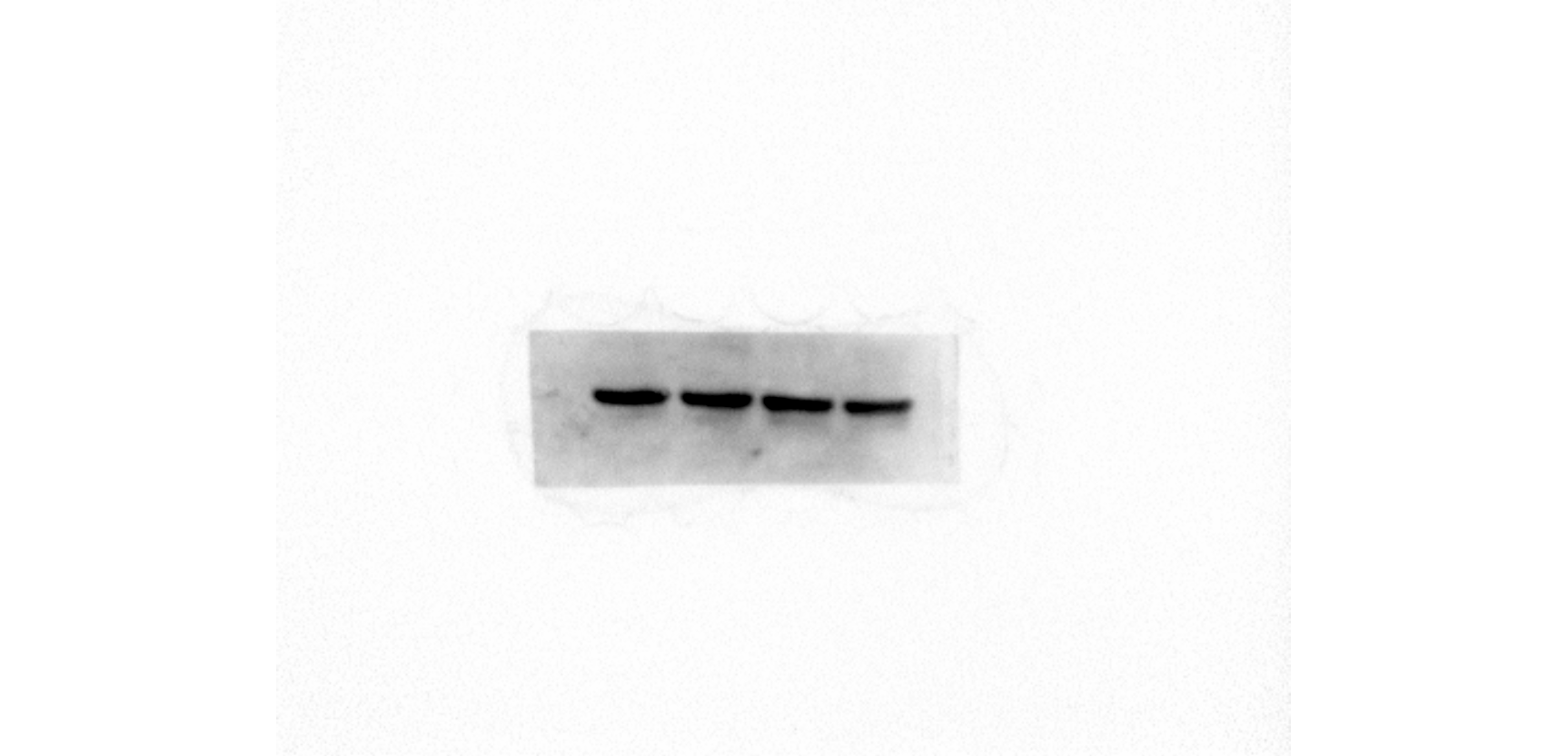

Supplement: Supplementary file 6 [file DataSheet6.ZIP › Fig.6-Source data/C/eIF-2a.tif]

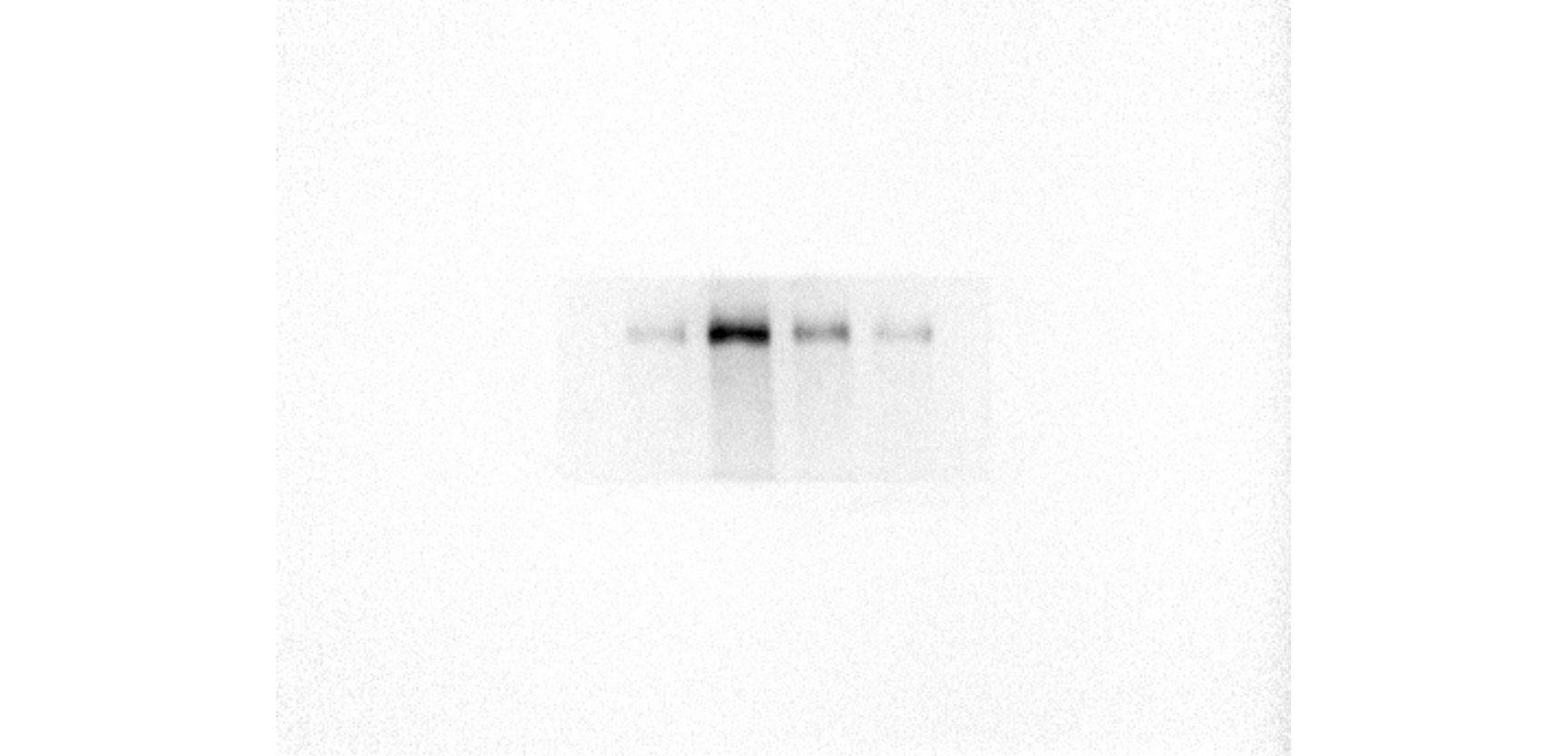

Supplement: Supplementary file 6 [file DataSheet6.ZIP › Fig.6-Source data/C/p-PERK.tif]

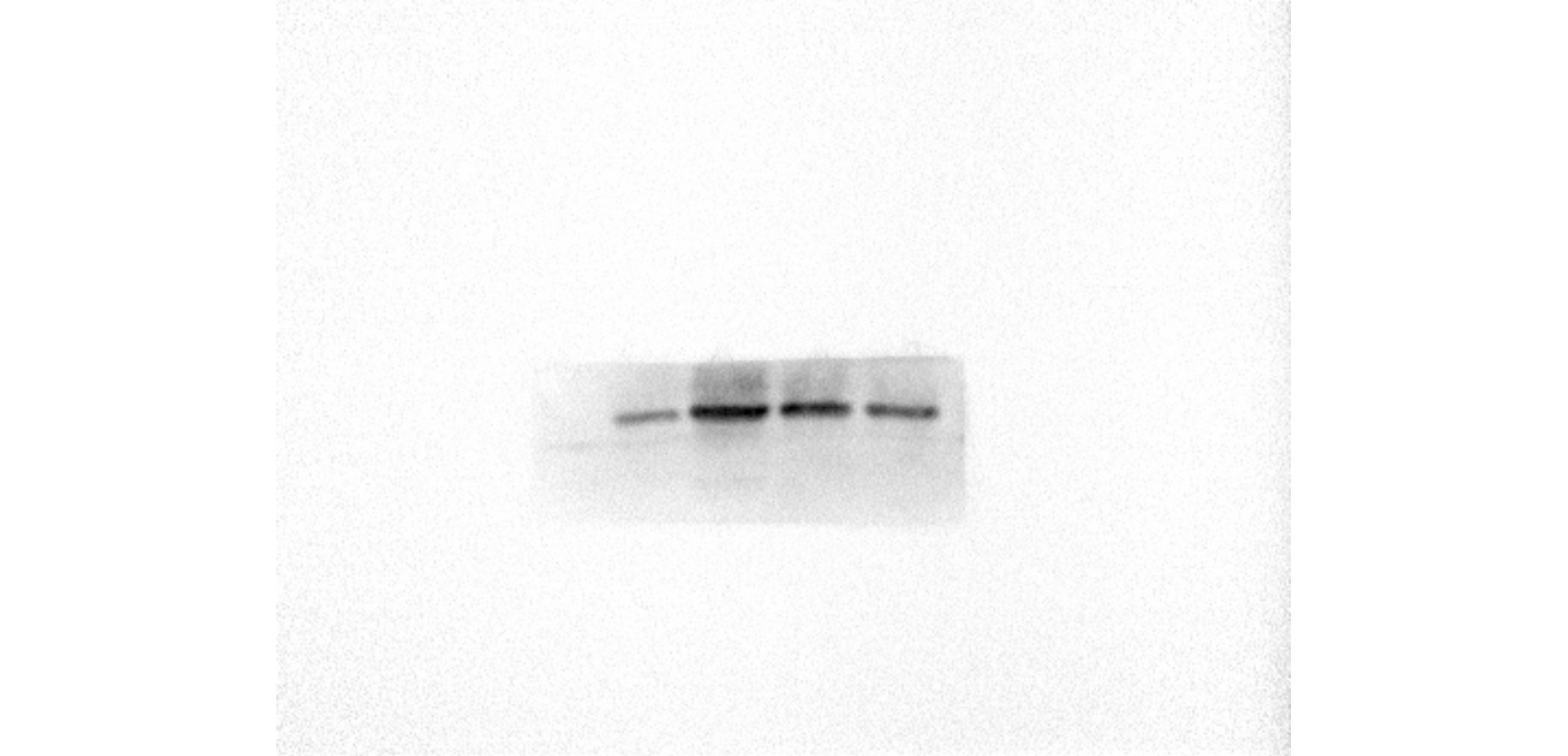

Supplement: Supplementary file 6 [file DataSheet6.ZIP › Fig.6-Source data/C/p-eIF-2a.tif]

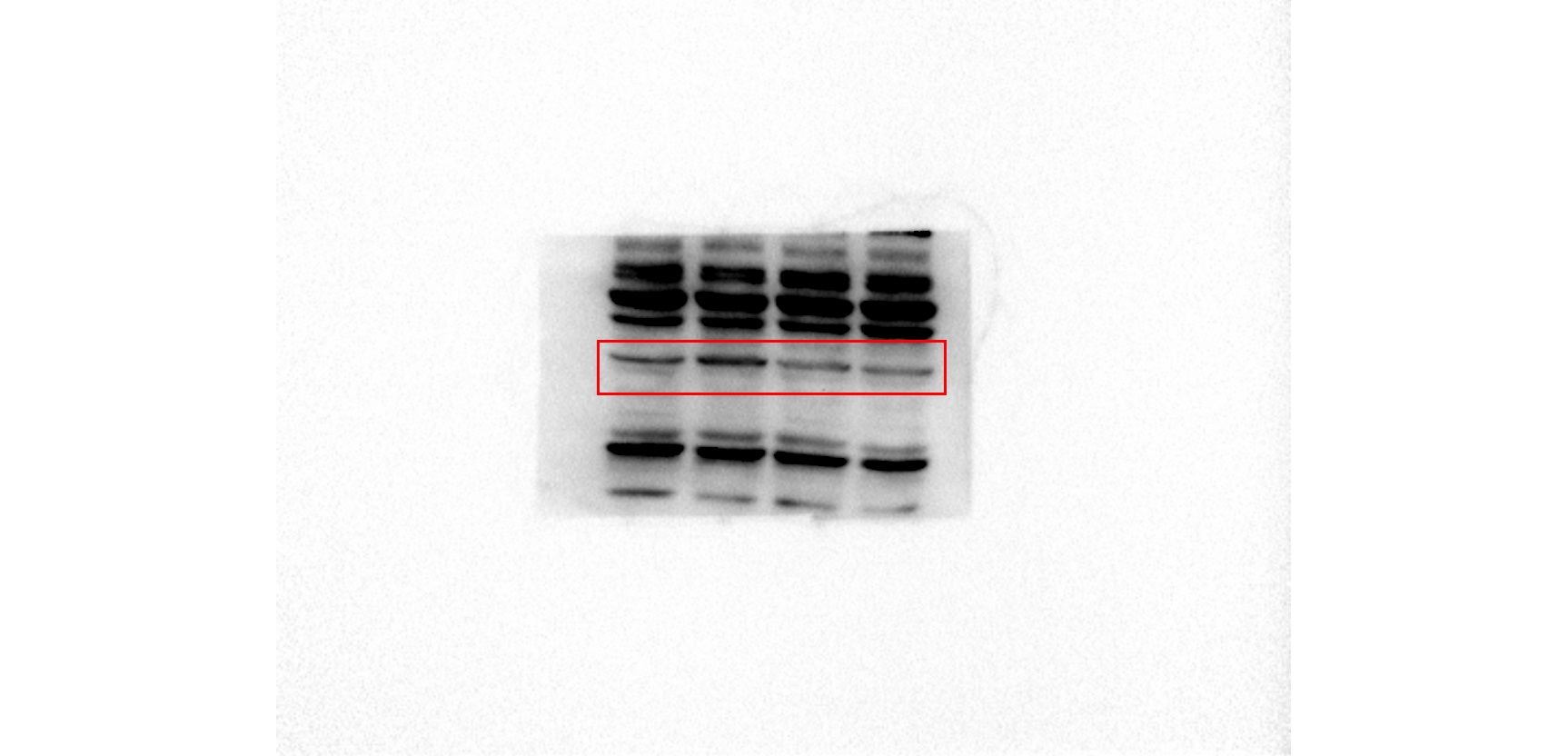

Supplement: Supplementary file 6 [file DataSheet6.ZIP › Fig.6-Source data/D/ATF4.jpg]

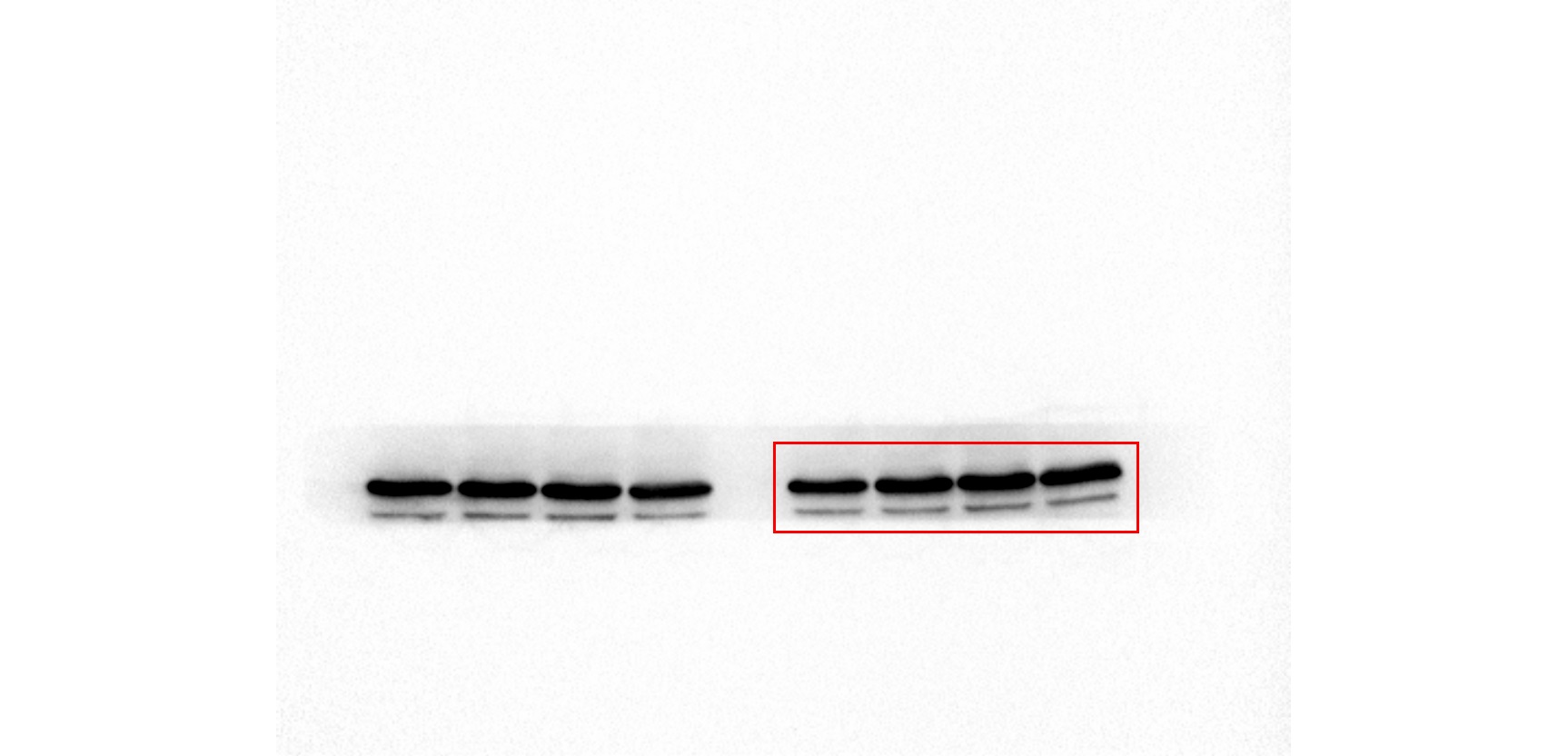

Supplement: Supplementary file 6 [file DataSheet6.ZIP › Fig.6-Source data/D/GAPDH.jpg]

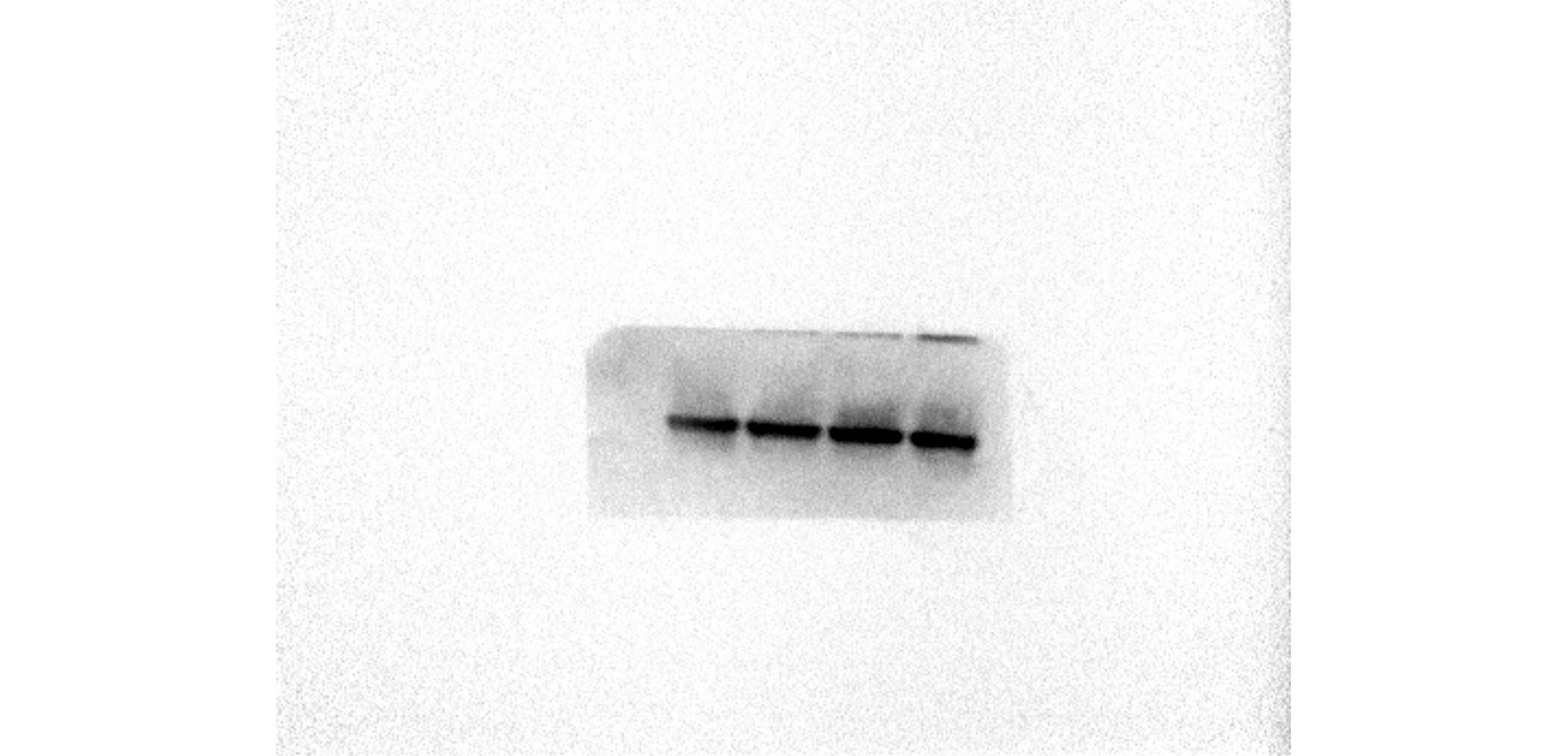

Supplement: Supplementary file 6 [file DataSheet6.ZIP › Fig.6-Source data/D/eIF-2a.jpg]
