# Supplementary material for: Nox4 Promotes RANKL-Induced Autophagy and Osteoclastogenesis via Activating ROS/PERK/eIF-2α/ATF4 Pathway
Source: Front Pharmacol. 2021 Sep 28;12:751845. doi: 10.3389/fphar.2021.751845 (PMC8505706; doi:10.3389/fphar.2021.751845)
Supplement: Supplementary file 2 [file Table1.docx]

**SUPPLEMENTARY TABLE S1 |** Primer sequences used for qRT-PCR.

| Gene | Sequence (5′ to 3′) | Amplicon size (bp) |
| --- | --- | --- |
| TRAP | Forward: ACTTGCGACCATTGTTAGCCACAT | 91 |
|  | Reverse: ACACCGTTCTCGTCCTGAAGATACT |  |
| MMP-9 | Forward: CCAGTATCTGTATGGTCGTGGCTCTA | 82 |
|  | Reverse: AGGTGCTGTCGGCTGTGGTT |  |
| Cath K | Forward: AGGATATGCTCTCTTGGCTCGGAAT | 91 |
|  | Reverse: GCTGGCTGGCTGGAATCACATC |  |
| β-actin | Forward: TCACTATTGGCAACGAGCGGTTC | 153 |
|  | Reverse: GCACTGTGTTGGCATAGAGGTCTT |  |
