# Supplementary figures and images for: Nox4 Promotes RANKL-Induced Autophagy and Osteoclastogenesis via Activating ROS/PERK/eIF-2α/ATF4 Pathway (part 2 of 3)
Source: Front Pharmacol. 2021 Sep 28;12:751845. doi: 10.3389/fphar.2021.751845 (PMC8505706; doi:10.3389/fphar.2021.751845)

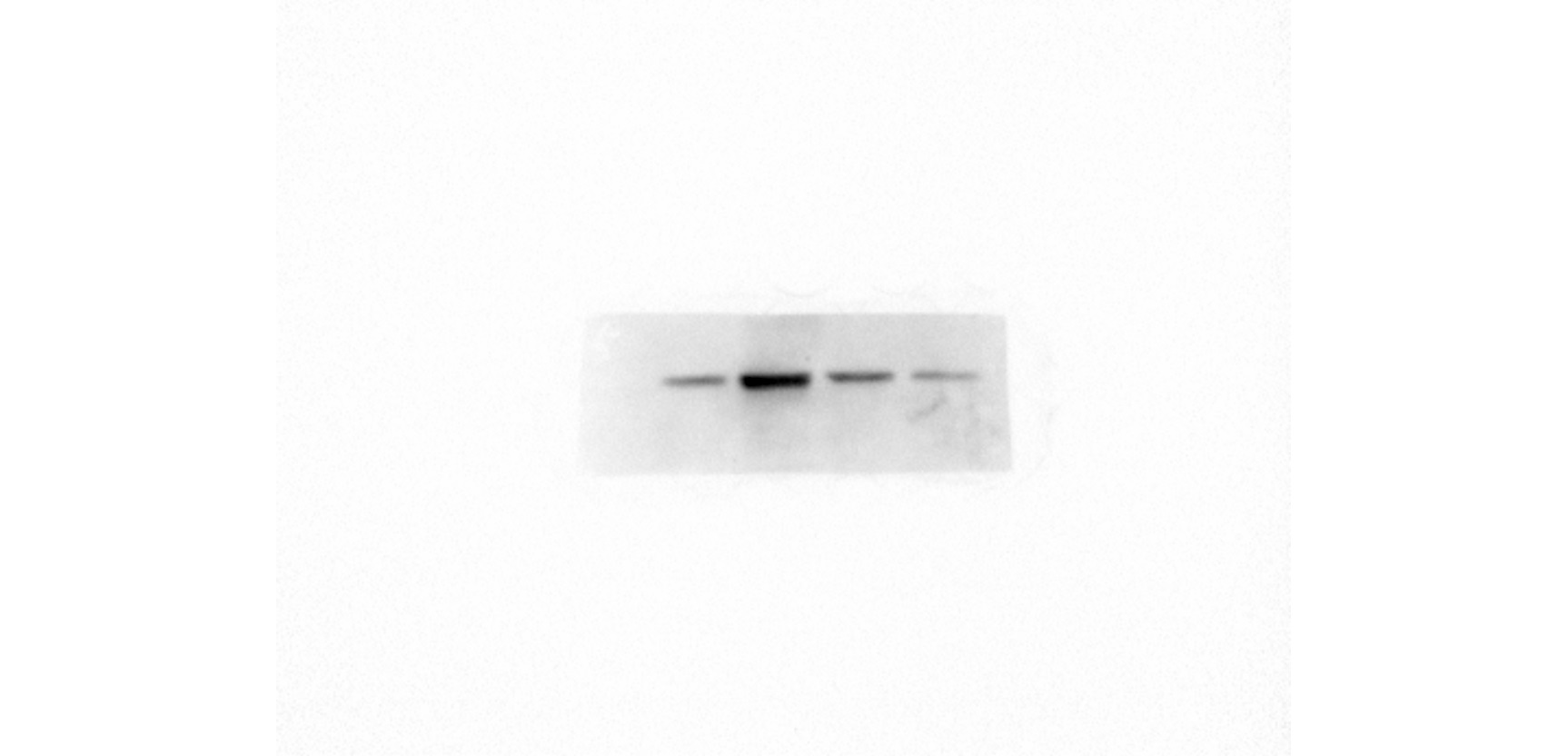

Supplement: Supplementary file 6 [file DataSheet6.ZIP › Fig.6-Source data/D/p-eIF-2a.jpg]

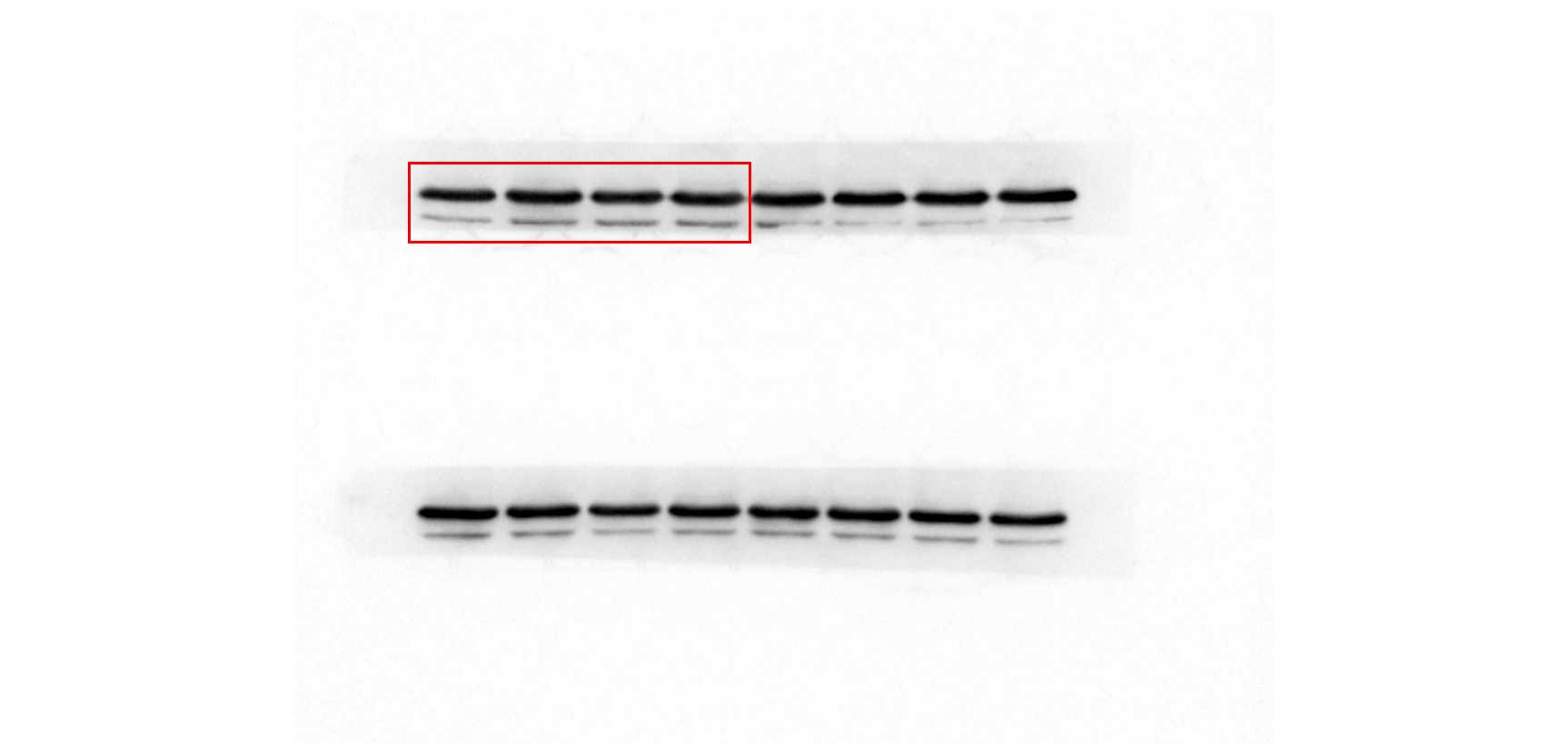

Supplement: Supplementary file 6 [file DataSheet6.ZIP › Fig.6-Source data/E/GAPDH.jpg]

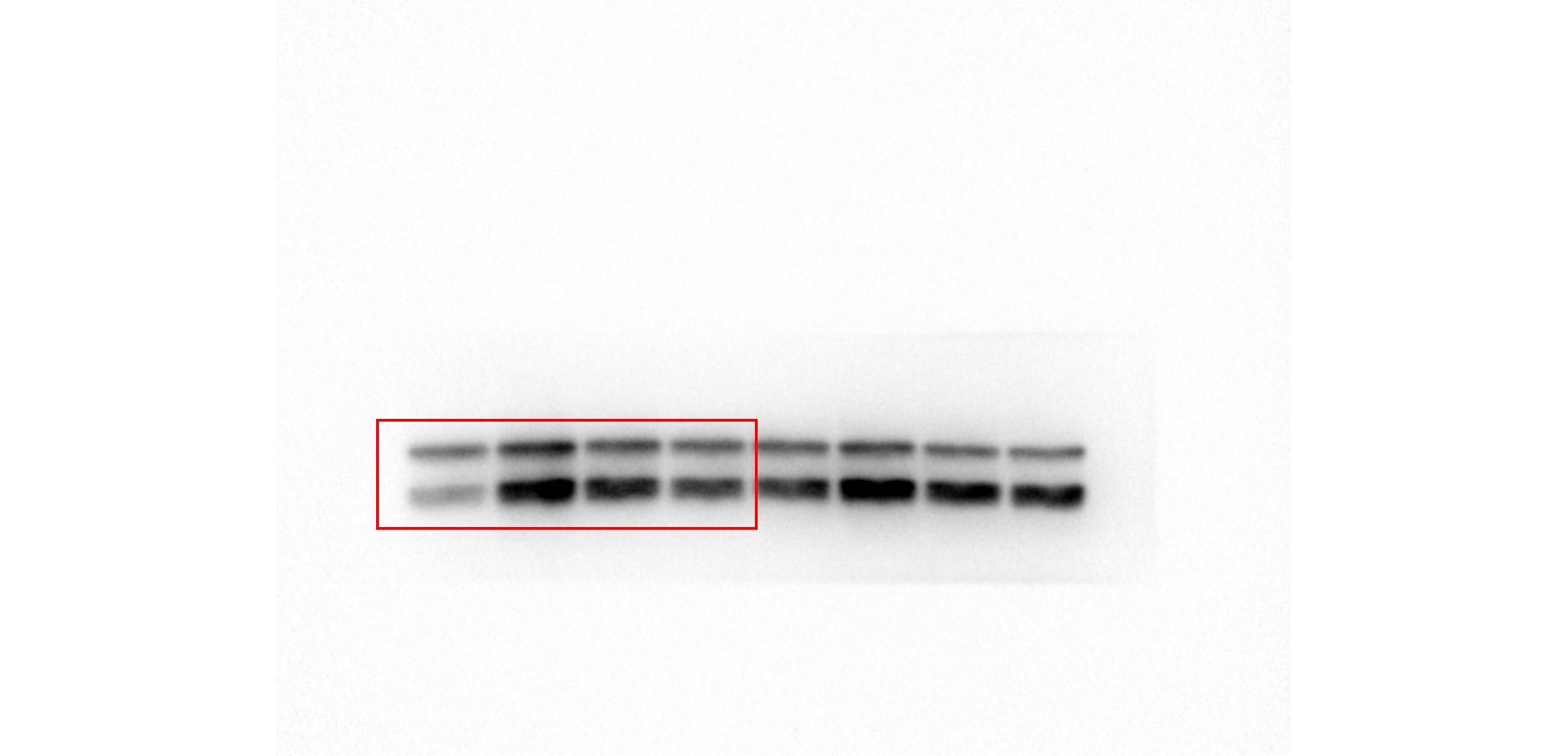

Supplement: Supplementary file 6 [file DataSheet6.ZIP › Fig.6-Source data/E/LC3.jpg]

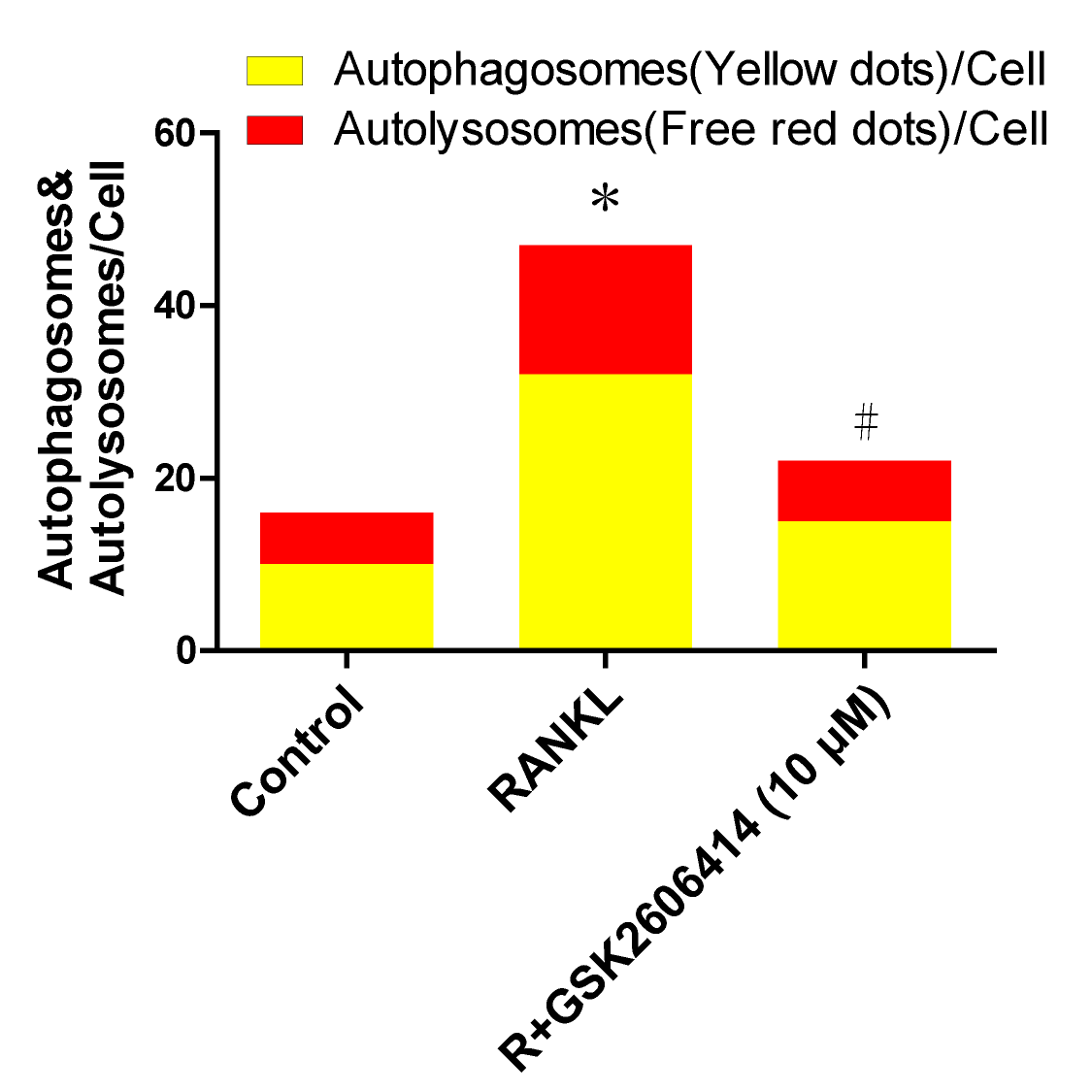

Supplement: Supplementary file 6 [file DataSheet6.ZIP › Fig.6-Source data/F/Fig.6F.tif]

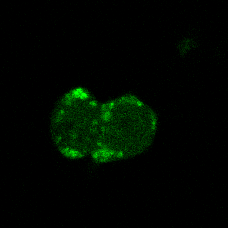

Supplement: Supplementary file 6 [file DataSheet6.ZIP › Fig.6-Source data/F/RANKL+GSK2606414-GFP.tif]

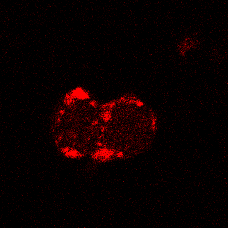

Supplement: Supplementary file 6 [file DataSheet6.ZIP › Fig.6-Source data/F/RANKL+GSK2606414-mRFP.tif]

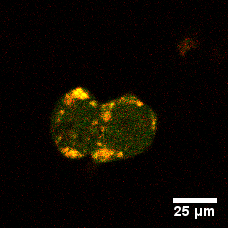

Supplement: Supplementary file 6 [file DataSheet6.ZIP › Fig.6-Source data/F/RANKL+GSK2606414-merged.tif]

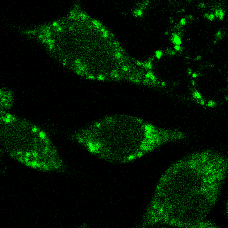

Supplement: Supplementary file 6 [file DataSheet6.ZIP › Fig.6-Source data/F/RANKL-GFP.tif]

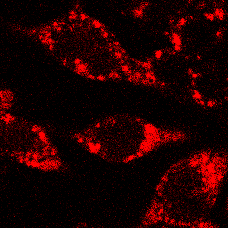

Supplement: Supplementary file 6 [file DataSheet6.ZIP › Fig.6-Source data/F/RANKL-mRFP.tif]

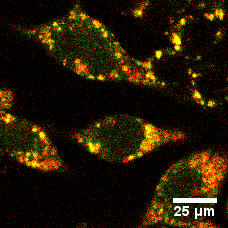

Supplement: Supplementary file 6 [file DataSheet6.ZIP › Fig.6-Source data/F/RANKL-merged.tif]

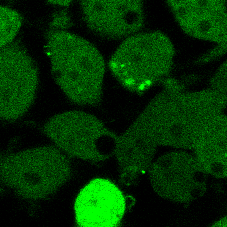

Supplement: Supplementary file 6 [file DataSheet6.ZIP › Fig.6-Source data/F/control-GFP.tif]

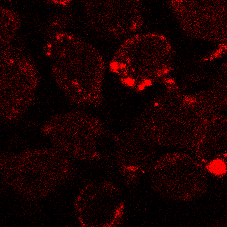

Supplement: Supplementary file 6 [file DataSheet6.ZIP › Fig.6-Source data/F/control-mRFP.tif]

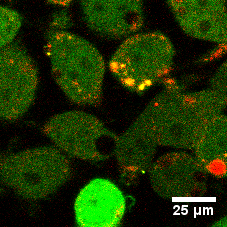

Supplement: Supplementary file 6 [file DataSheet6.ZIP › Fig.6-Source data/F/control-merged.tif]

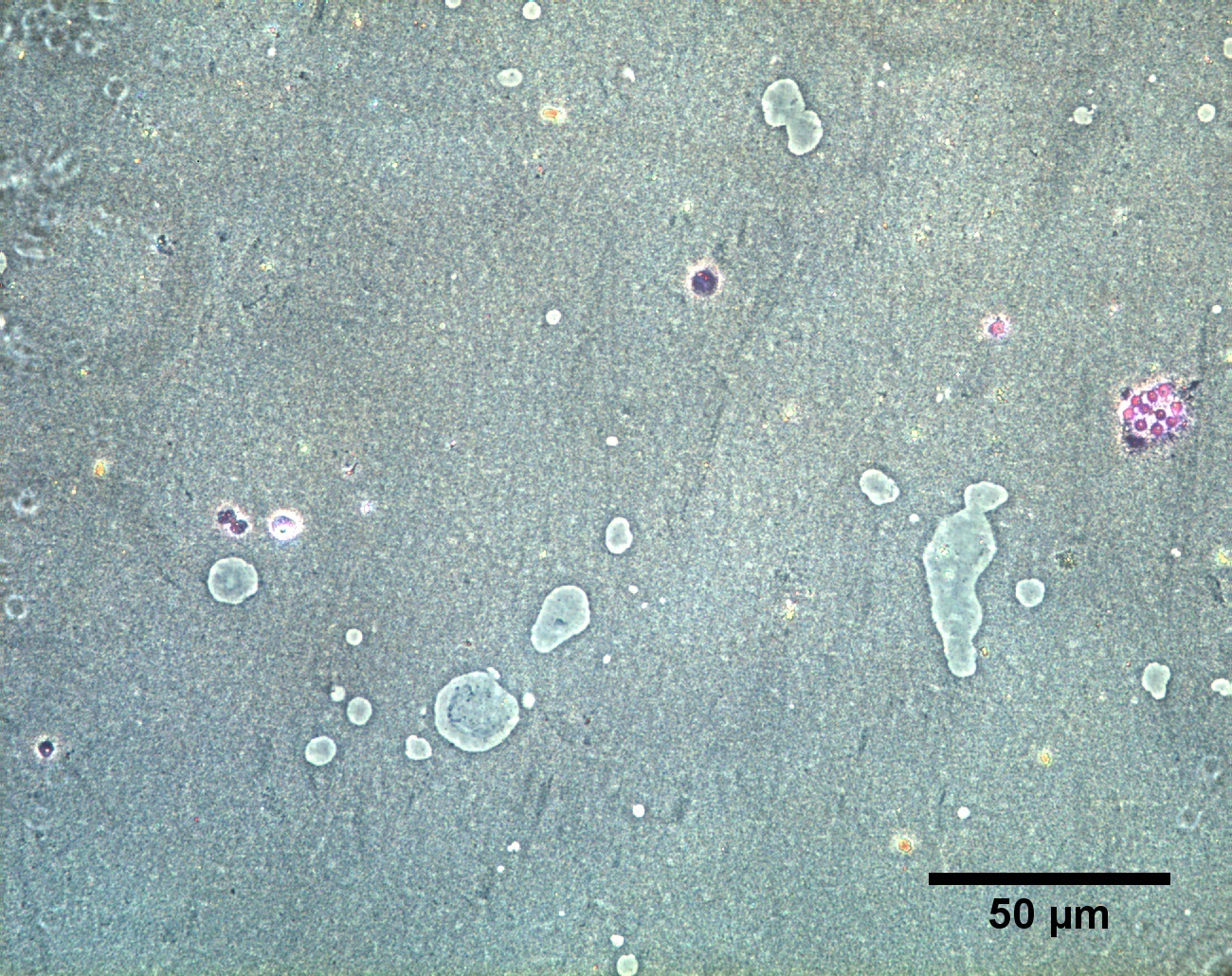

Supplement: Supplementary file 6 [file DataSheet6.ZIP › Fig.6-Source data/H/RANKL+GSK2606414 (10 a╠M).jpg]

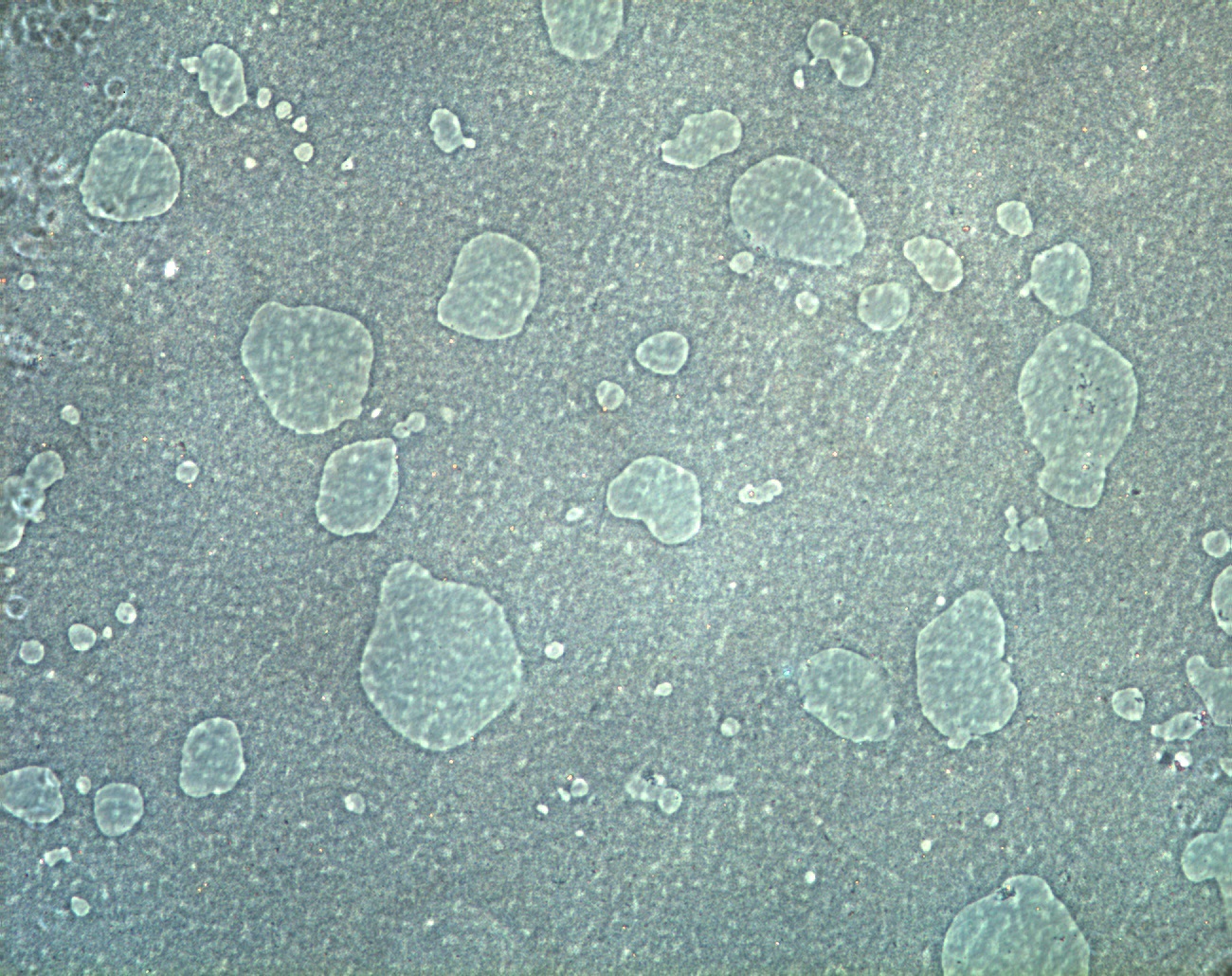

Supplement: Supplementary file 6 [file DataSheet6.ZIP › Fig.6-Source data/H/RANKL+GSK2606414 (5 a╠M).jpg]

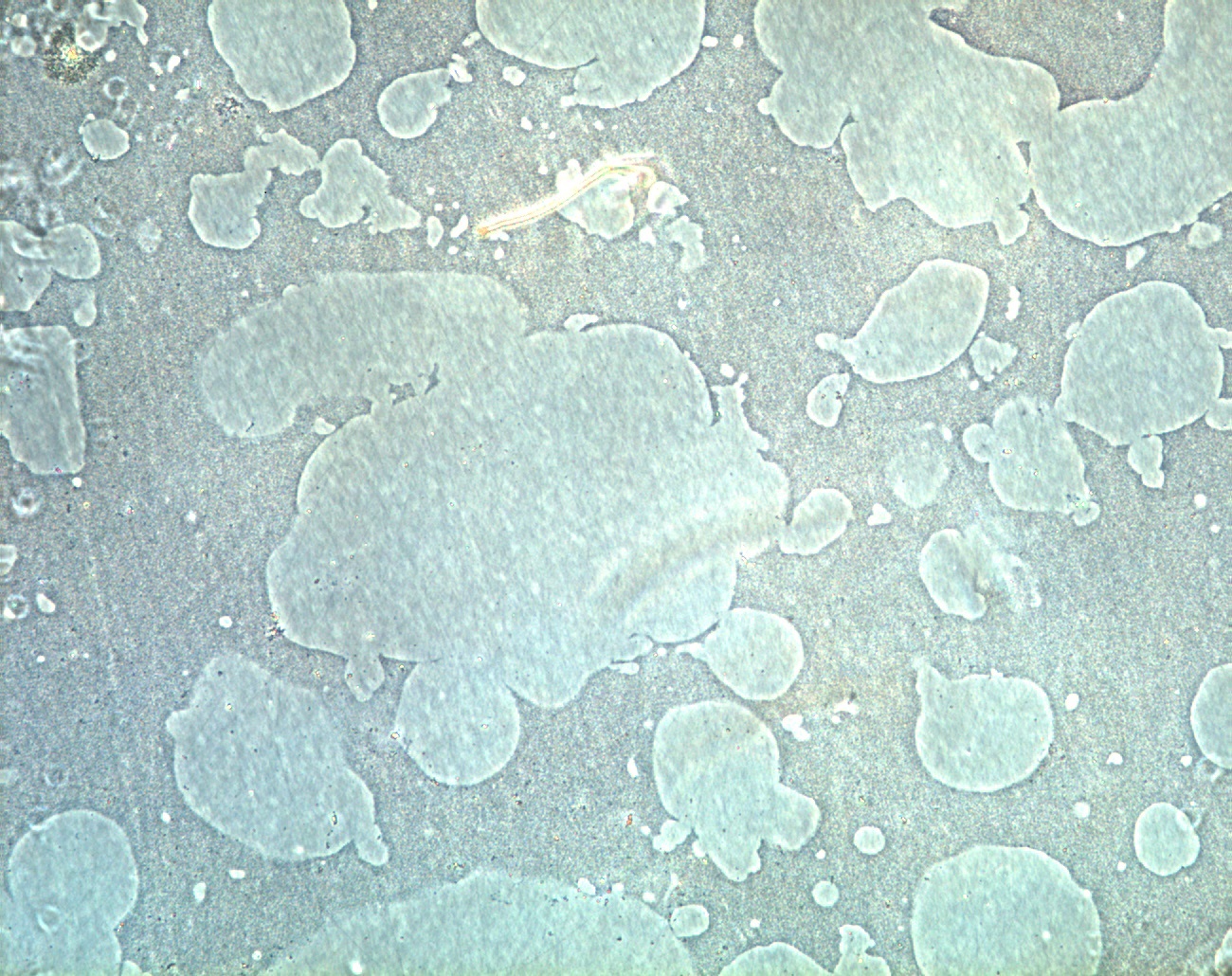

Supplement: Supplementary file 6 [file DataSheet6.ZIP › Fig.6-Source data/H/RANKL.jpg]

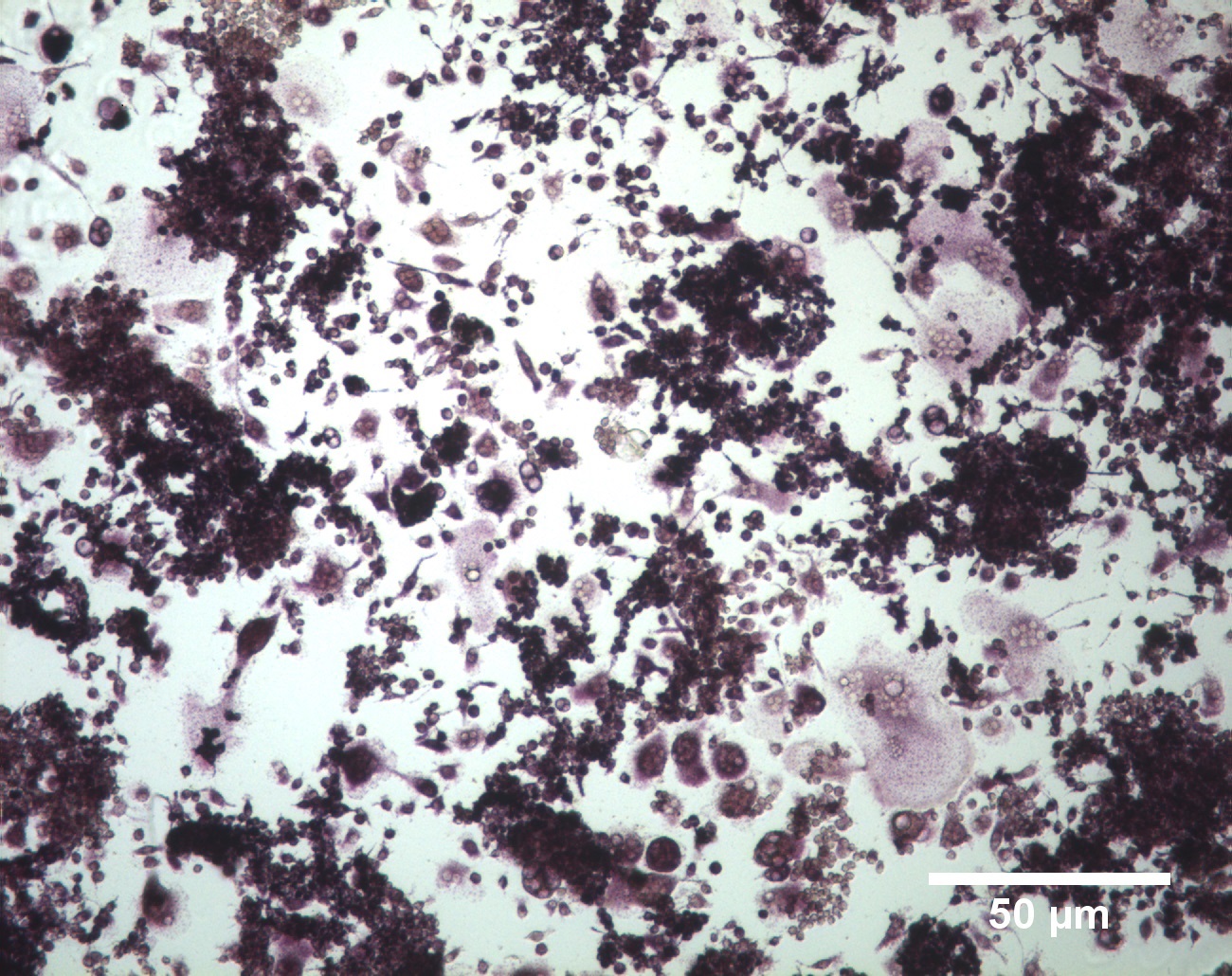

Supplement: Supplementary file 6 [file DataSheet6.ZIP › Fig.6-Source data/H/TRAP-RANKL+GSK2606414 (10 a╠M).jpg]

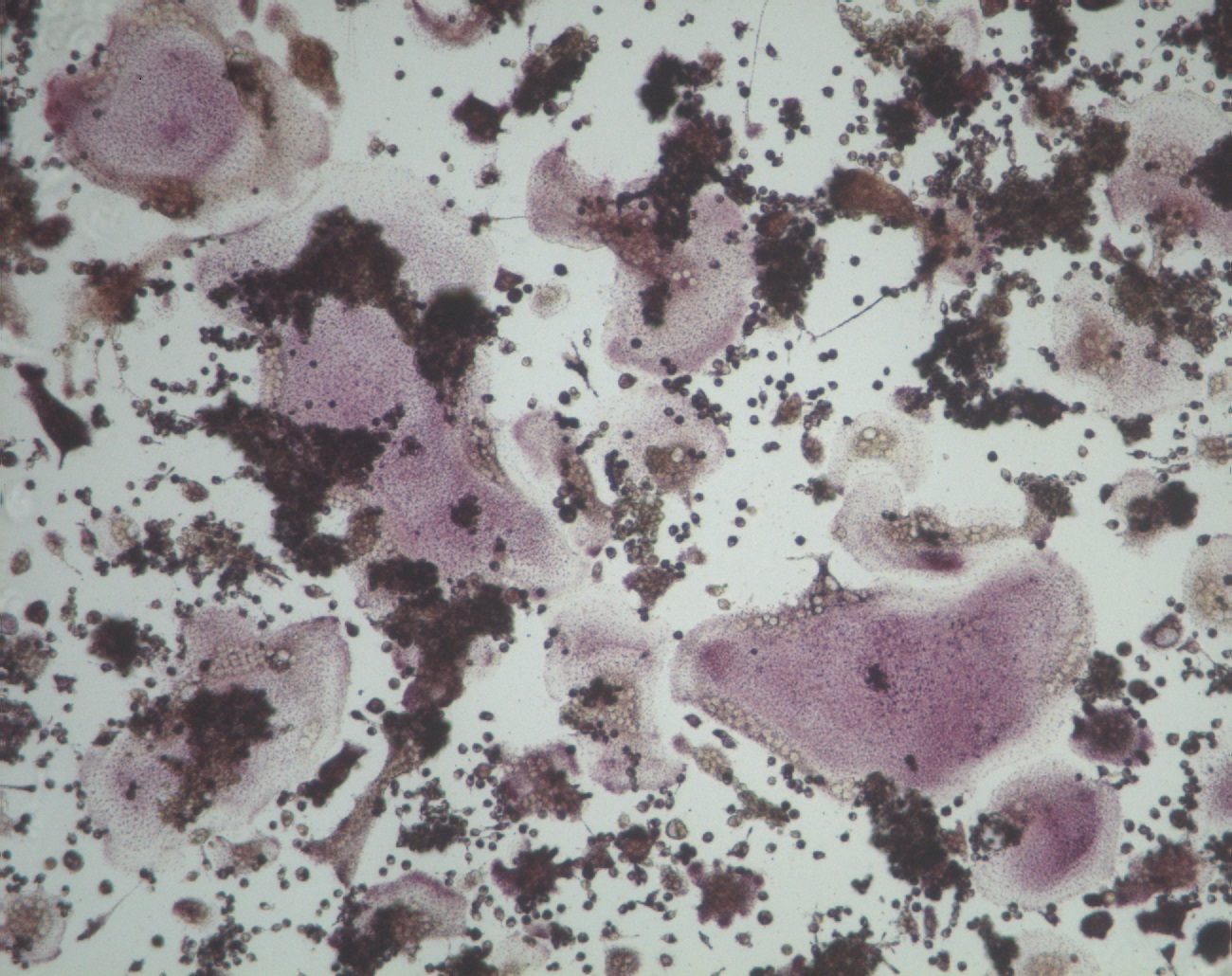

Supplement: Supplementary file 6 [file DataSheet6.ZIP › Fig.6-Source data/H/TRAP-RANKL+GSK2606414 (5 a╠M).jpg]

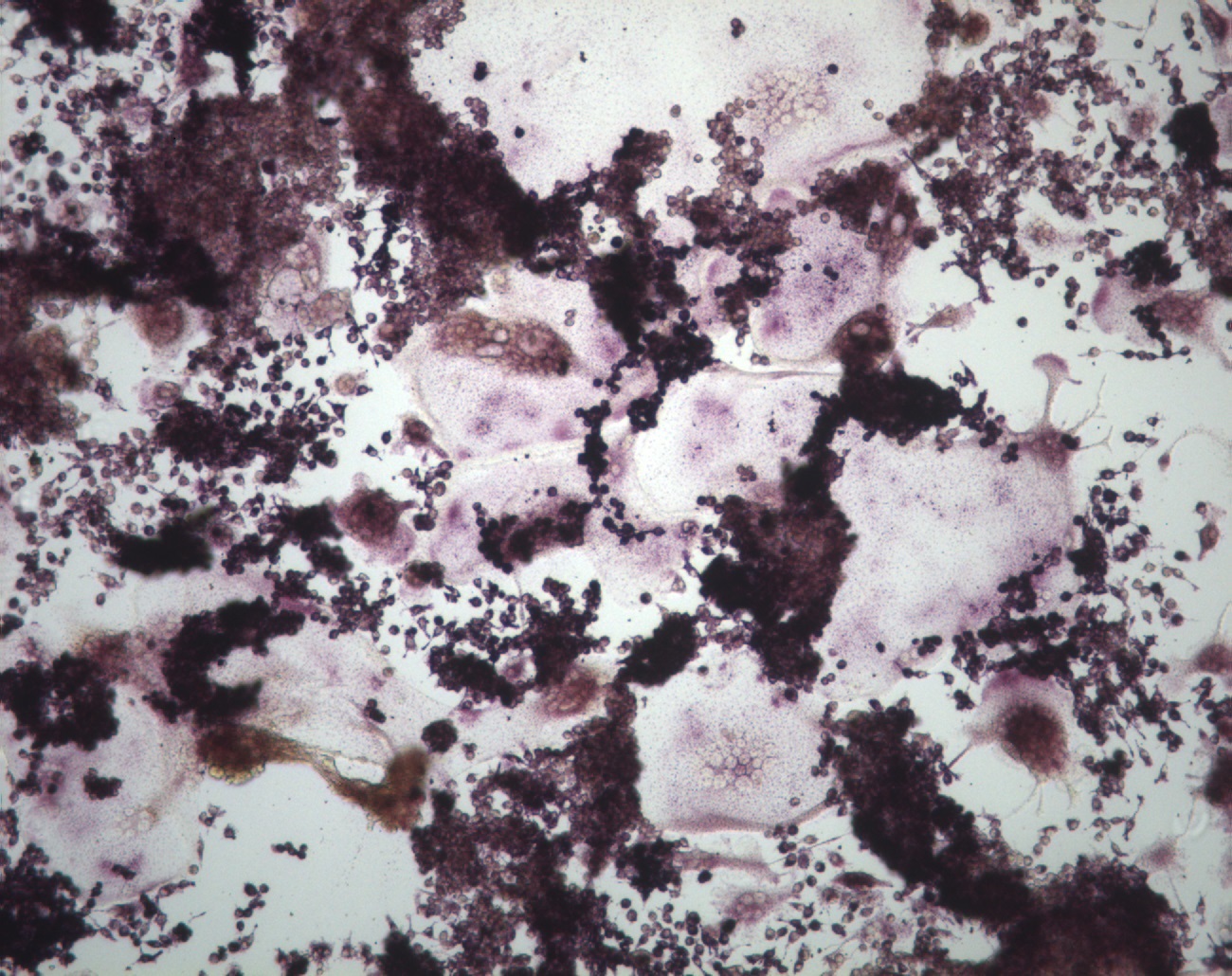

Supplement: Supplementary file 6 [file DataSheet6.ZIP › Fig.6-Source data/H/TRAP-RANKL.jpg]

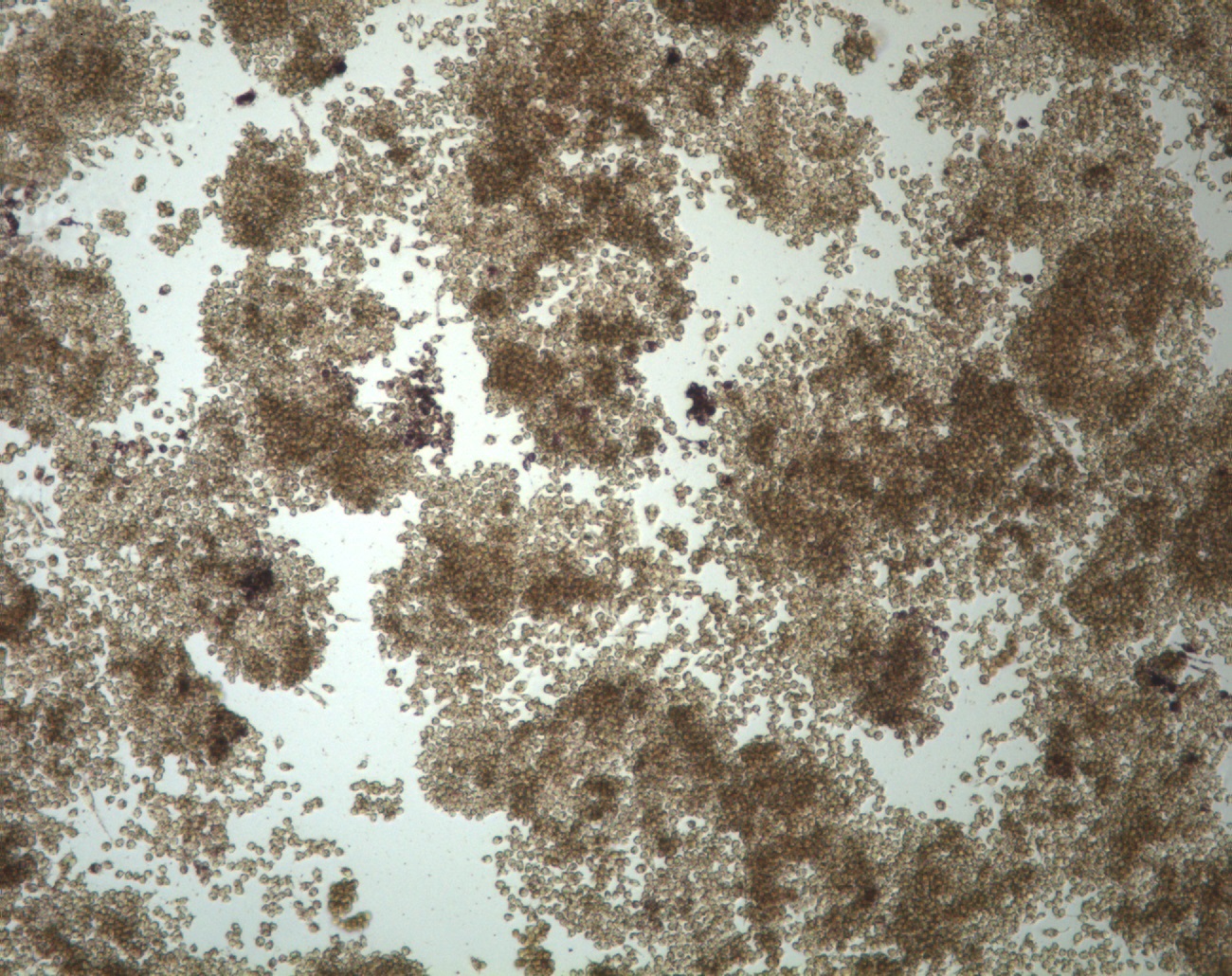

Supplement: Supplementary file 6 [file DataSheet6.ZIP › Fig.6-Source data/H/TRAP-control.jpg]

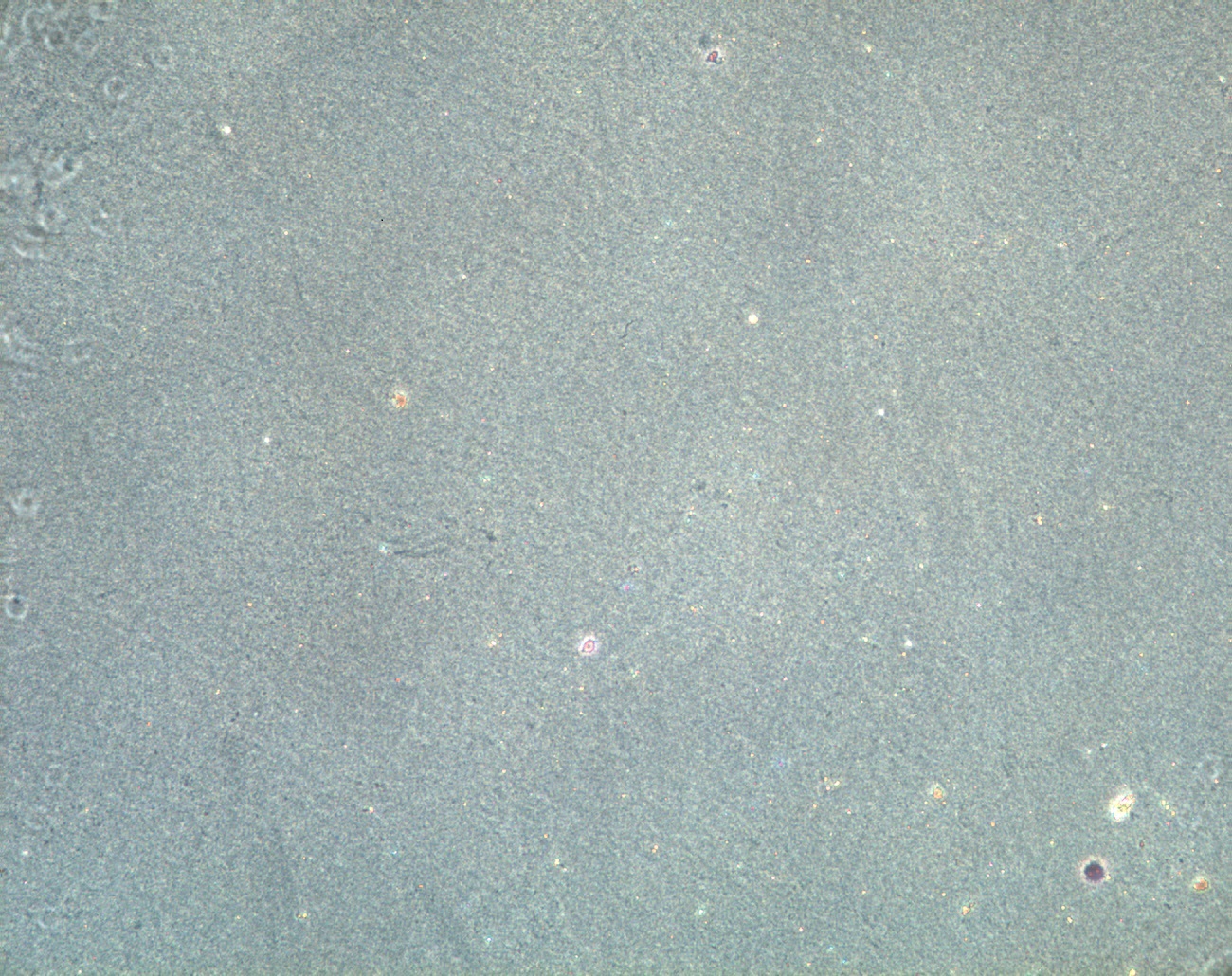

Supplement: Supplementary file 6 [file DataSheet6.ZIP › Fig.6-Source data/H/control.jpg]

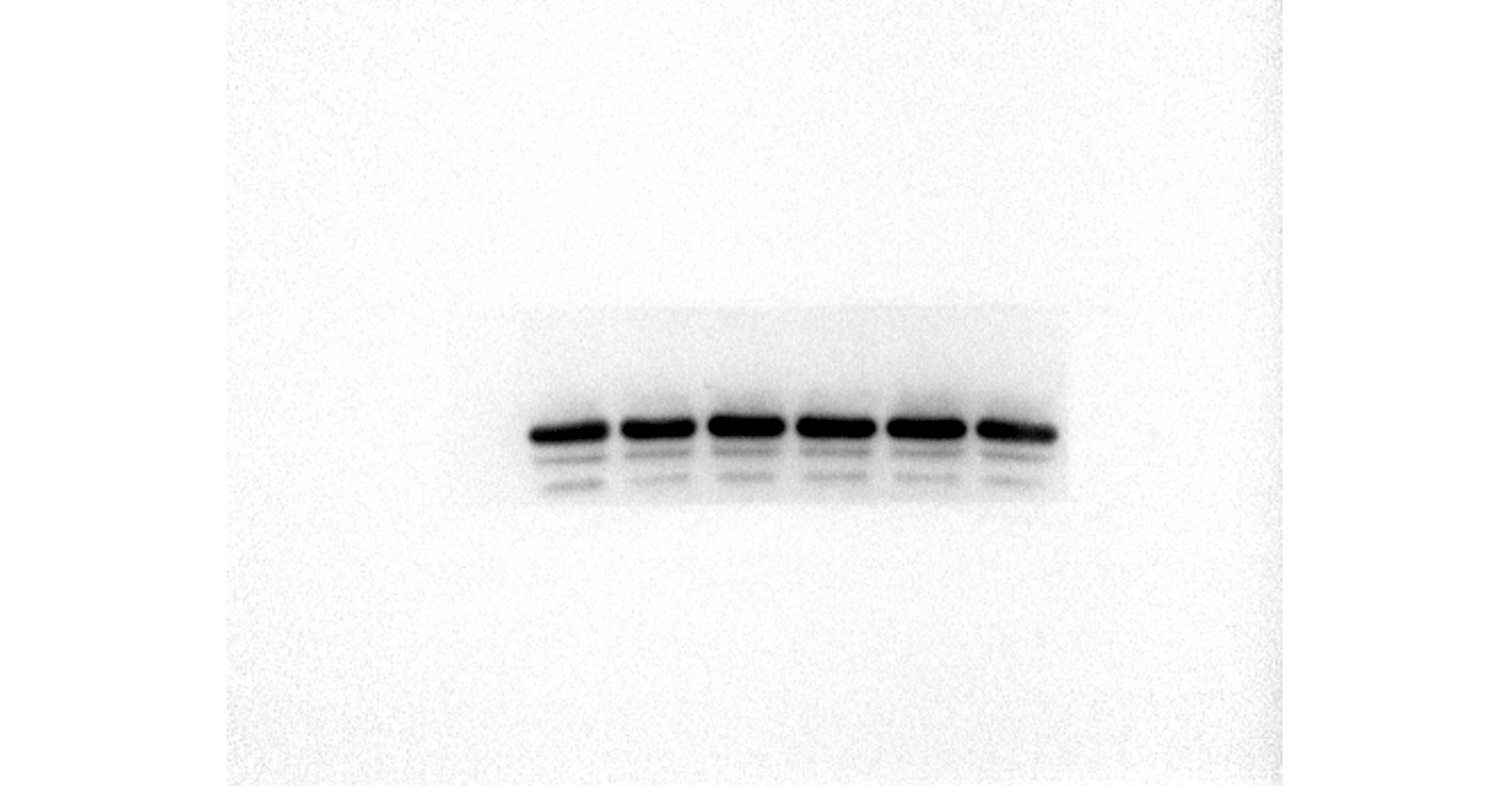

Supplement: Supplementary file 7 [file DataSheet2.ZIP › Fig.2-Source data/A/GAPDH.jpg]

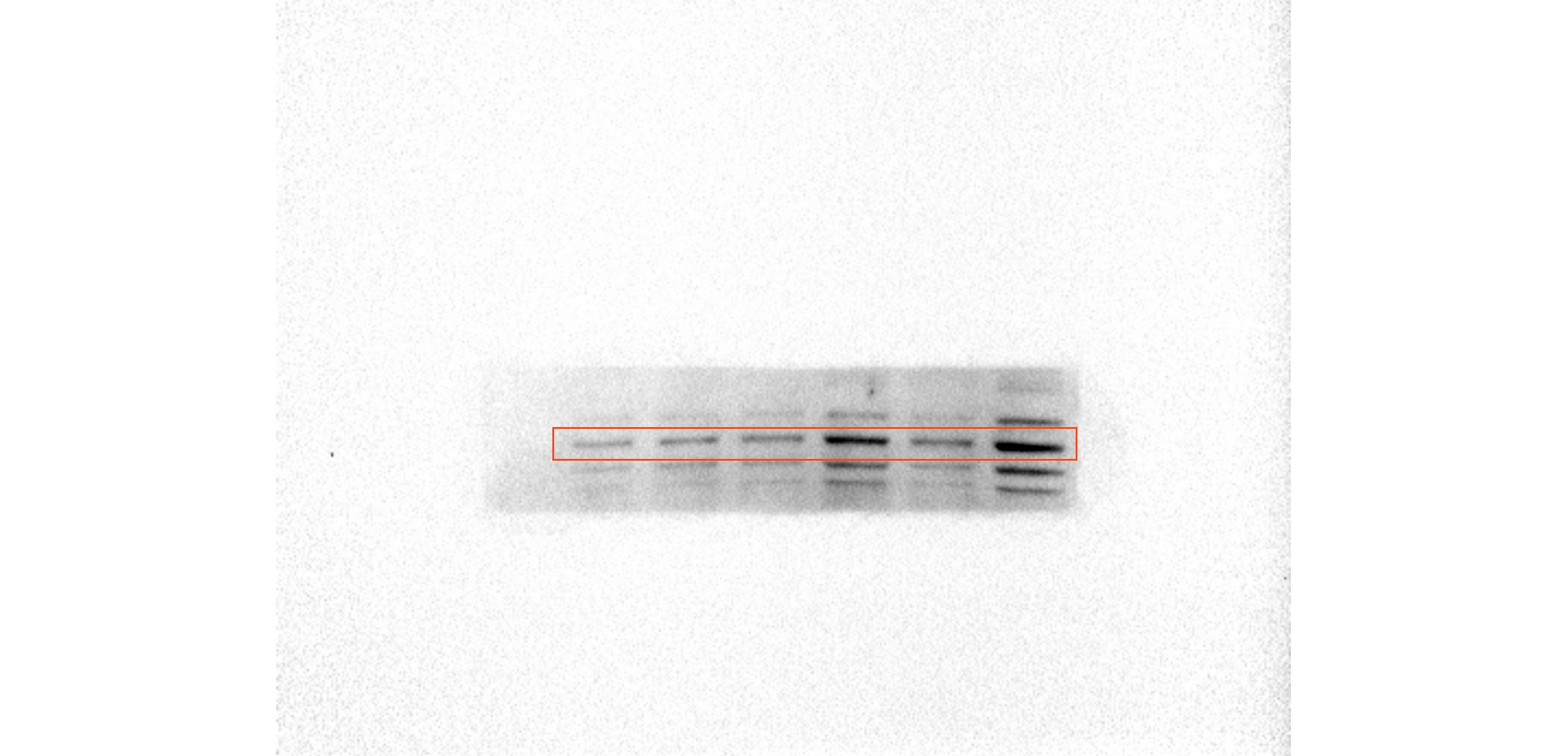

Supplement: Supplementary file 7 [file DataSheet2.ZIP › Fig.2-Source data/A/Nox1.jpg]

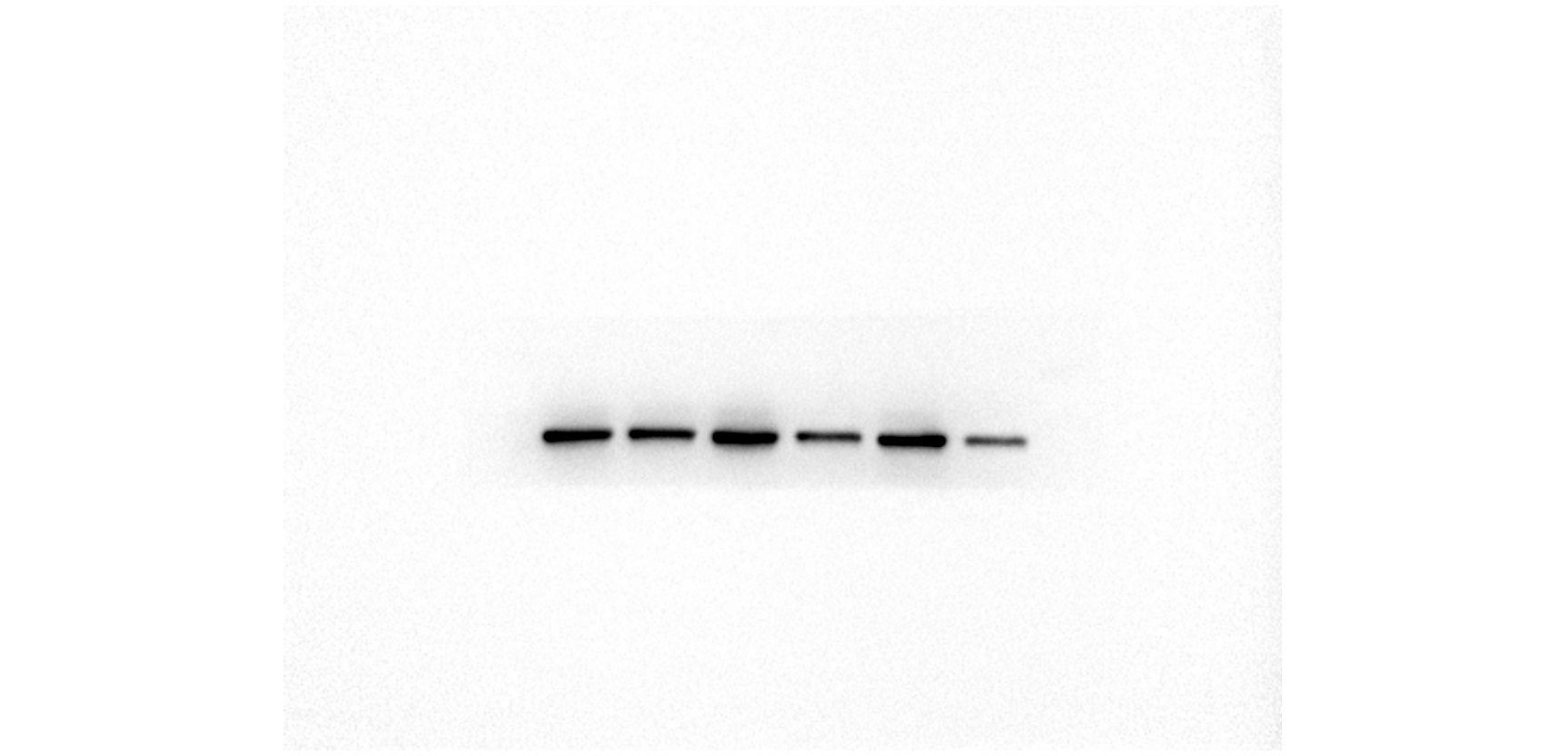

Supplement: Supplementary file 7 [file DataSheet2.ZIP › Fig.2-Source data/A/Nox2.jpg]

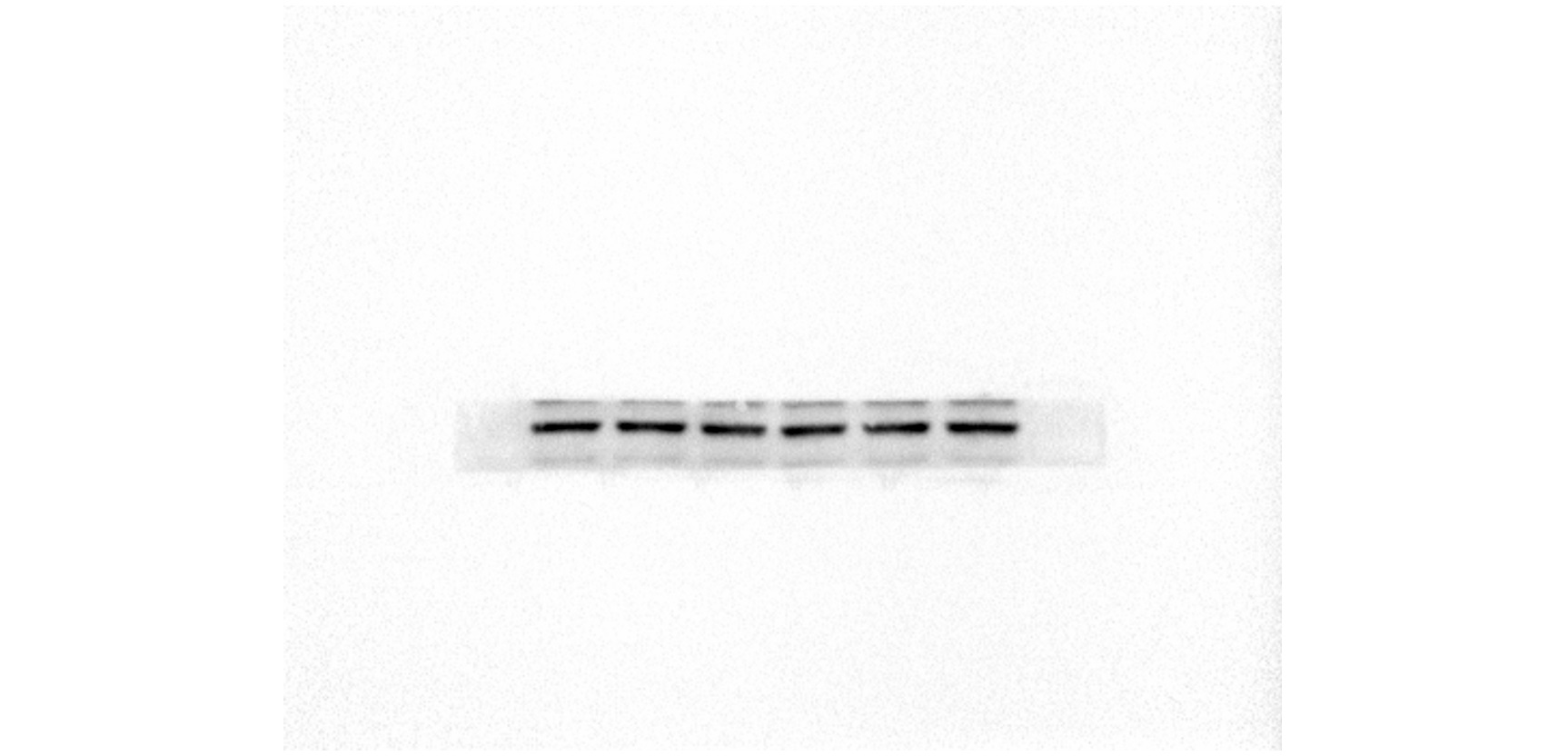

Supplement: Supplementary file 7 [file DataSheet2.ZIP › Fig.2-Source data/A/Nox3.jpg]

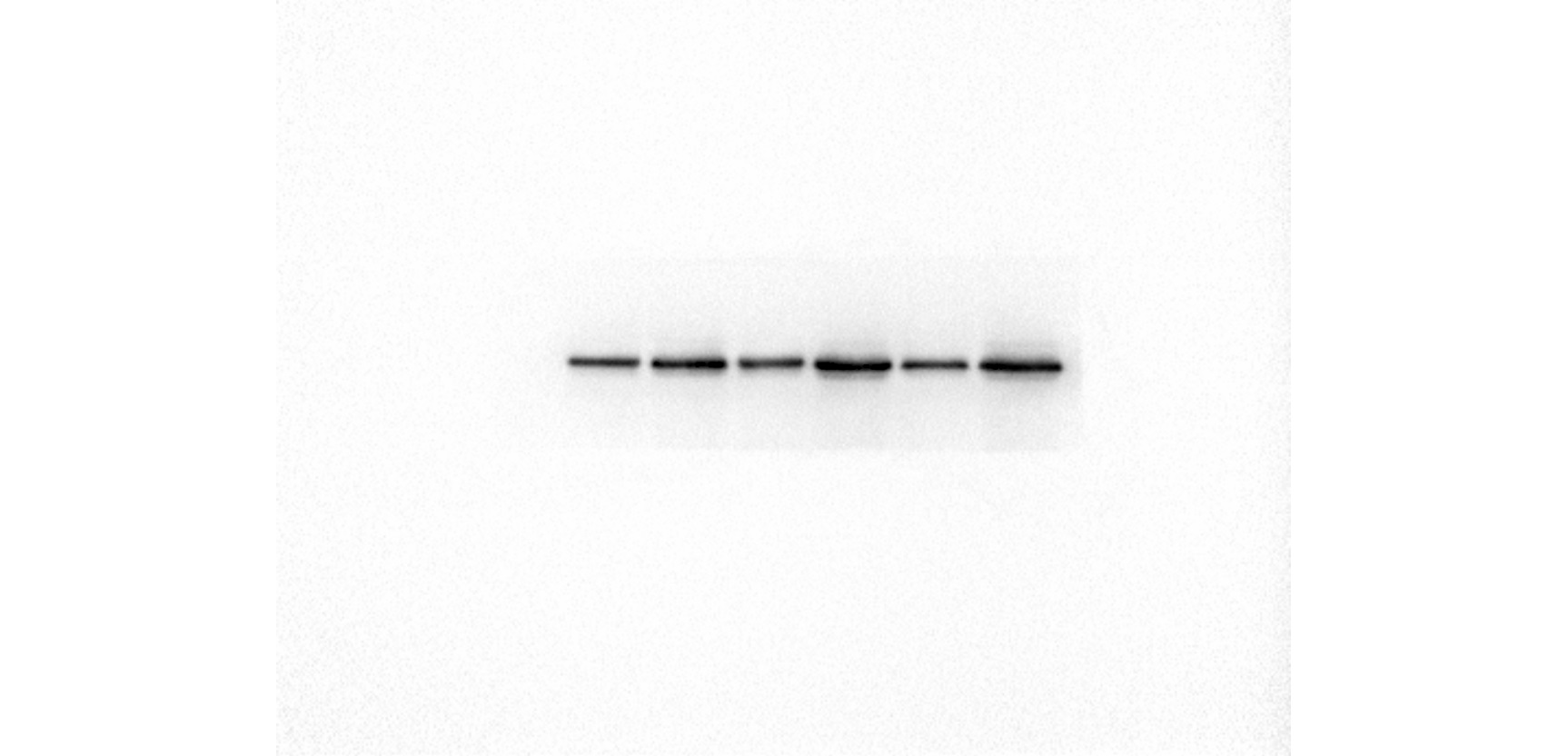

Supplement: Supplementary file 7 [file DataSheet2.ZIP › Fig.2-Source data/A/Nox4.jpg]

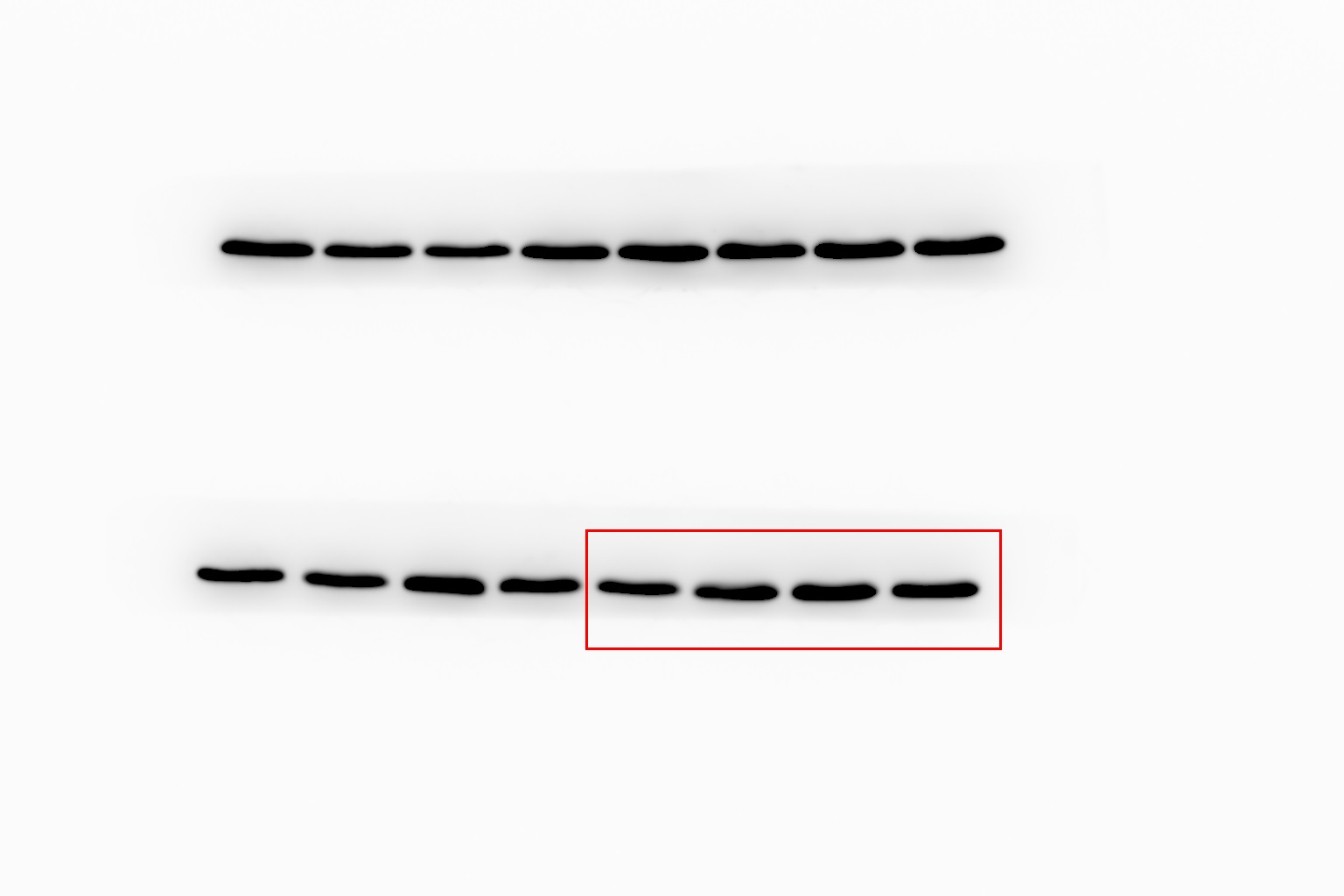

Supplement: Supplementary file 7 [file DataSheet2.ZIP › Fig.2-Source data/B/GAPDH.jpg]

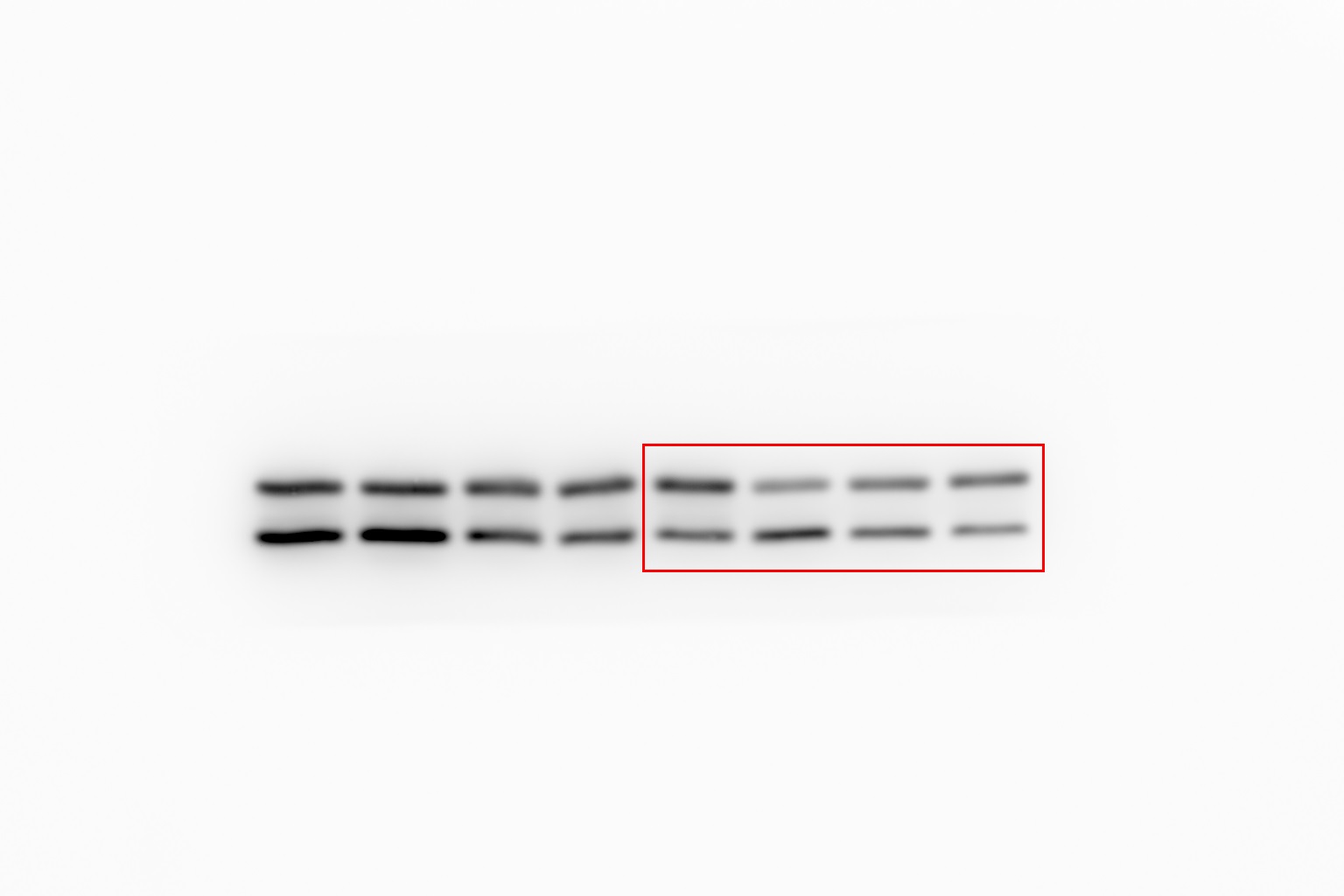

Supplement: Supplementary file 7 [file DataSheet2.ZIP › Fig.2-Source data/B/LC3.jpg]

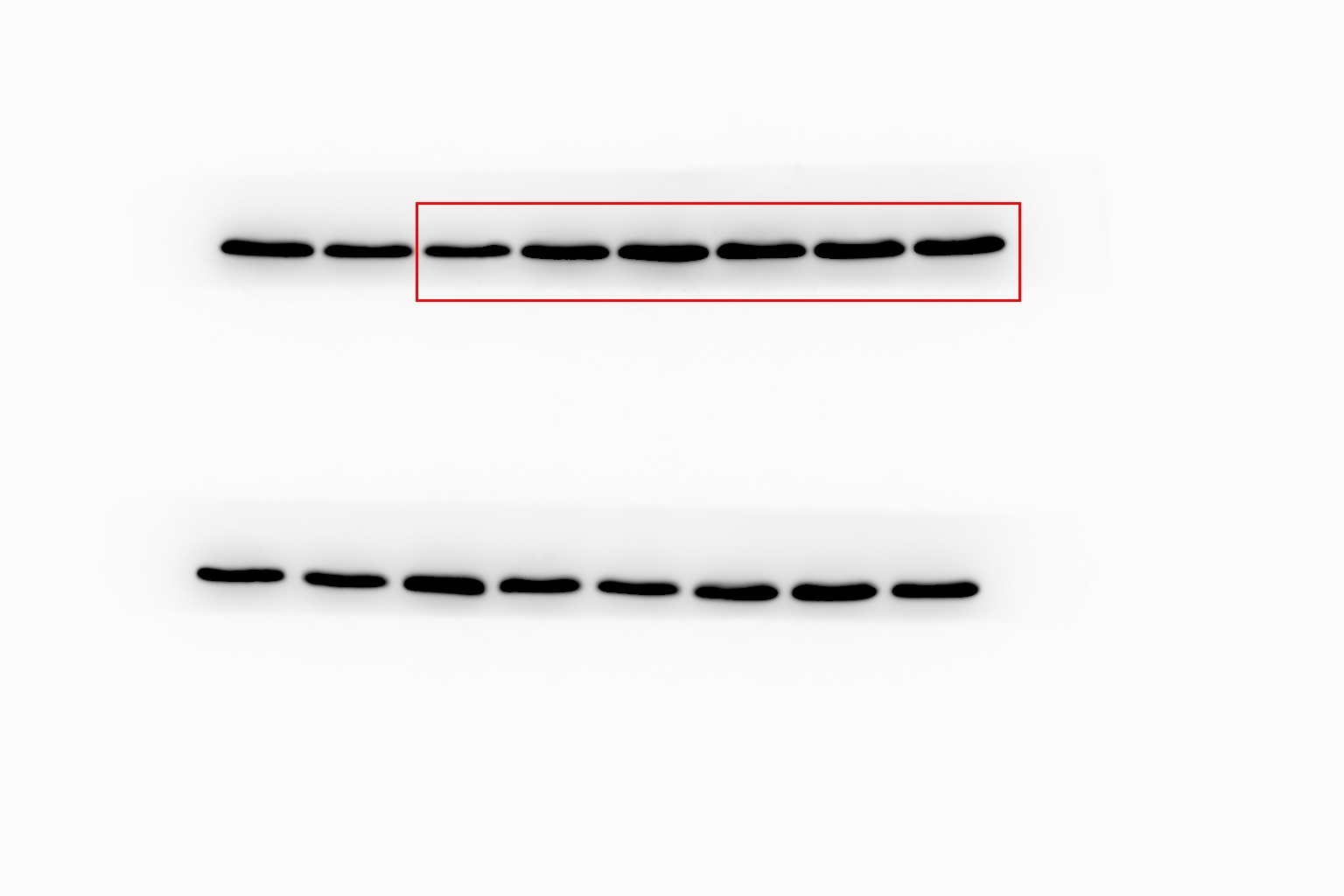

Supplement: Supplementary file 7 [file DataSheet2.ZIP › Fig.2-Source data/C/GAPDH.jpg]

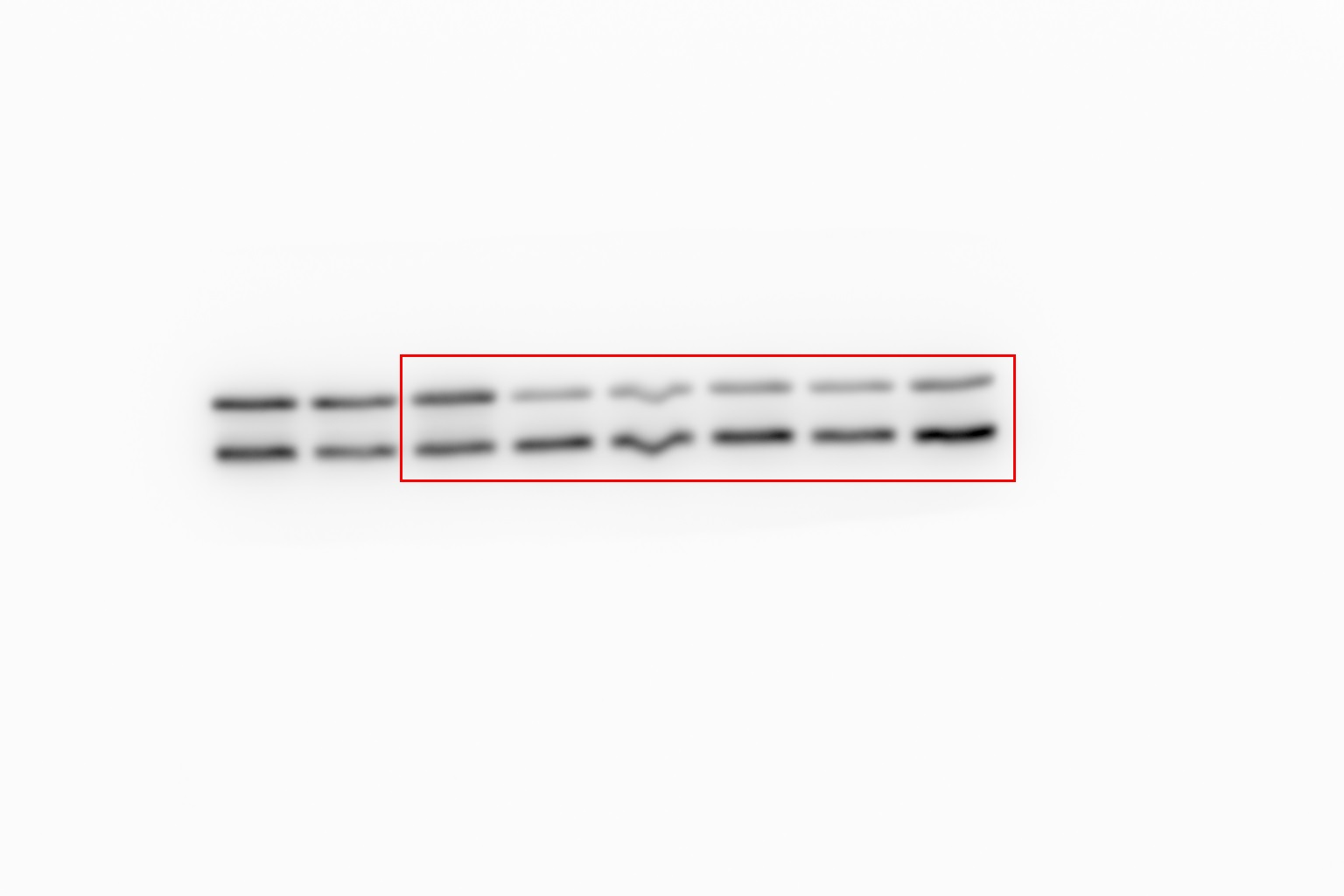

Supplement: Supplementary file 7 [file DataSheet2.ZIP › Fig.2-Source data/C/LC3.jpg]

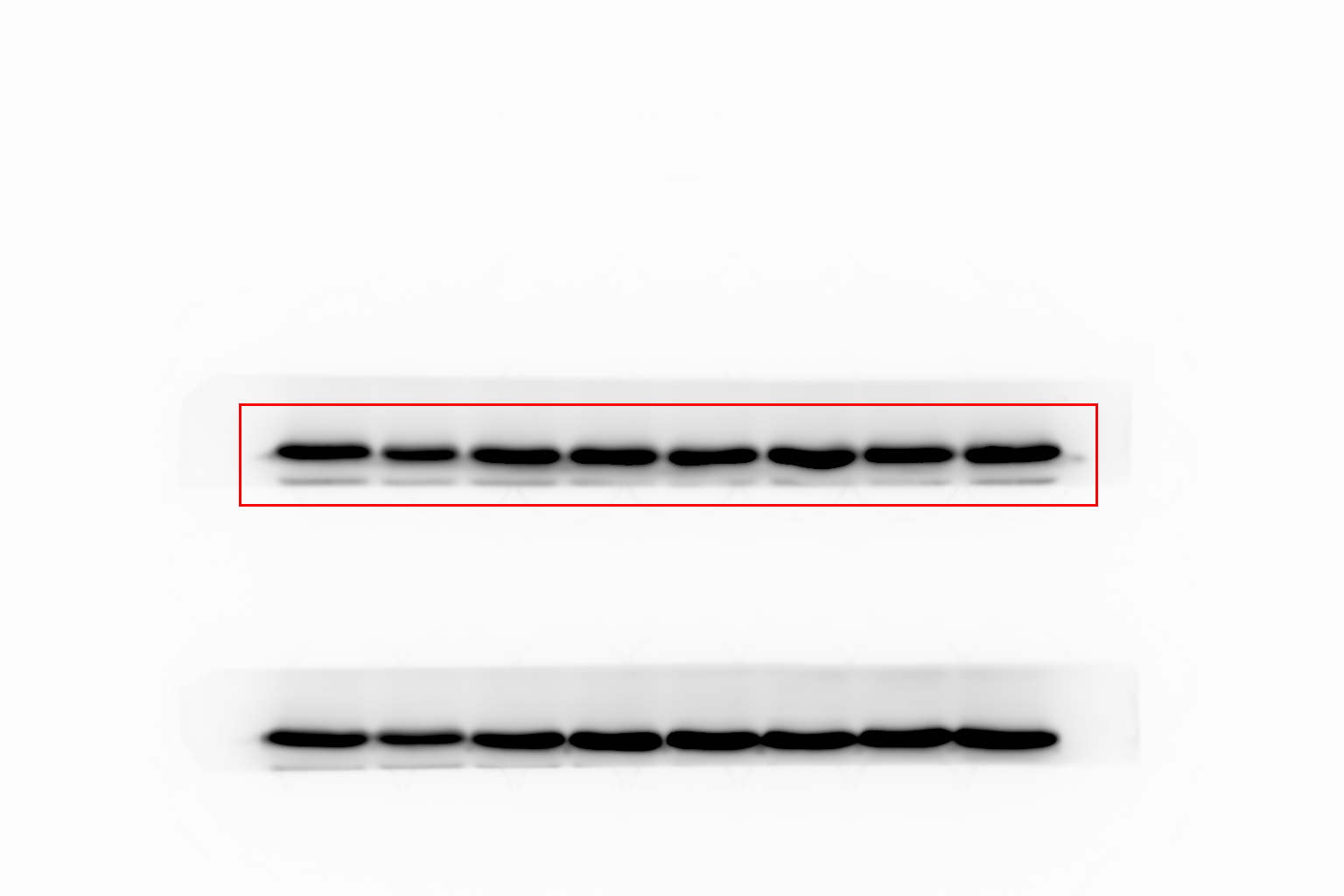

Supplement: Supplementary file 7 [file DataSheet2.ZIP › Fig.2-Source data/D/GAPDH.tif]

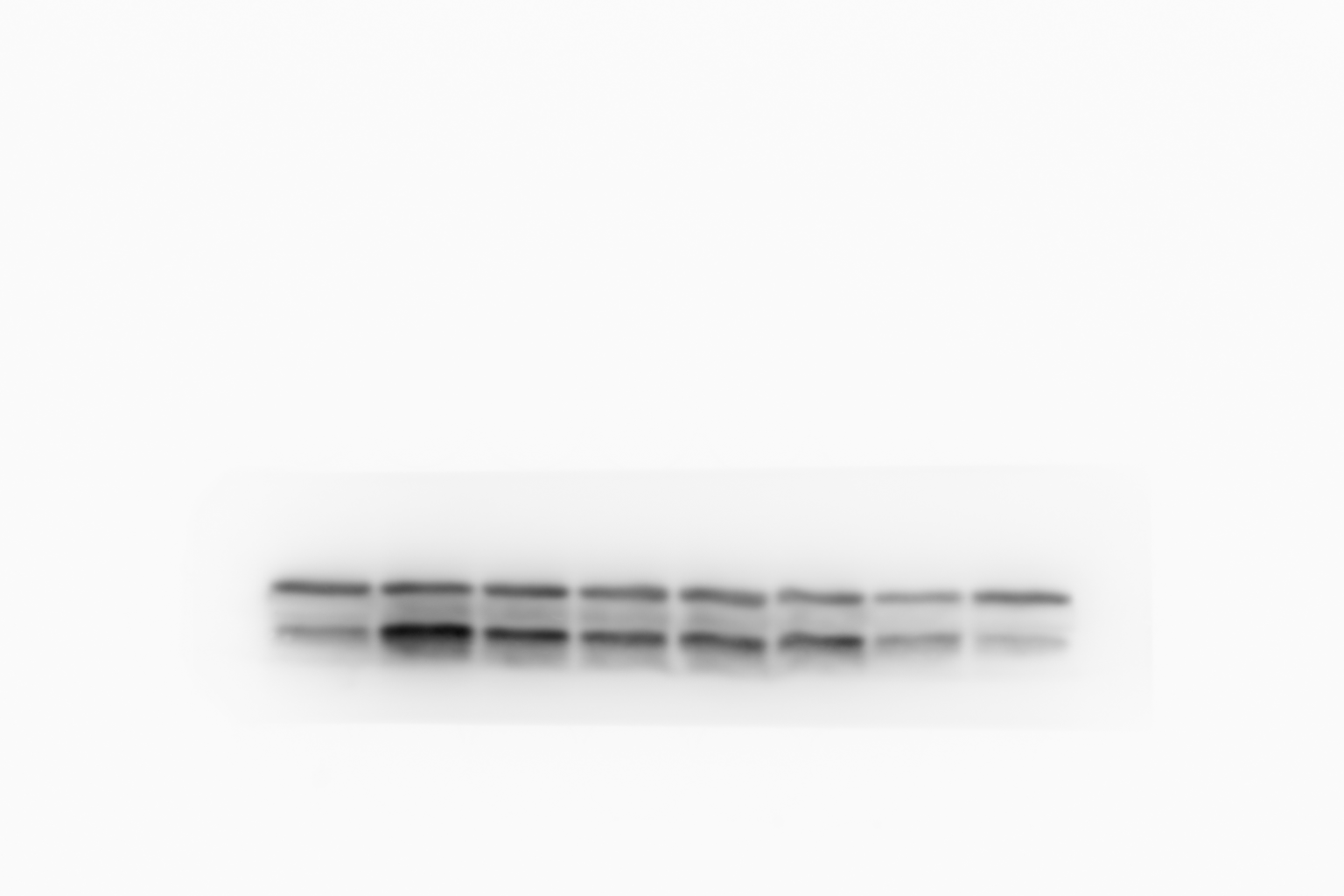

Supplement: Supplementary file 7 [file DataSheet2.ZIP › Fig.2-Source data/D/LC3.tif]

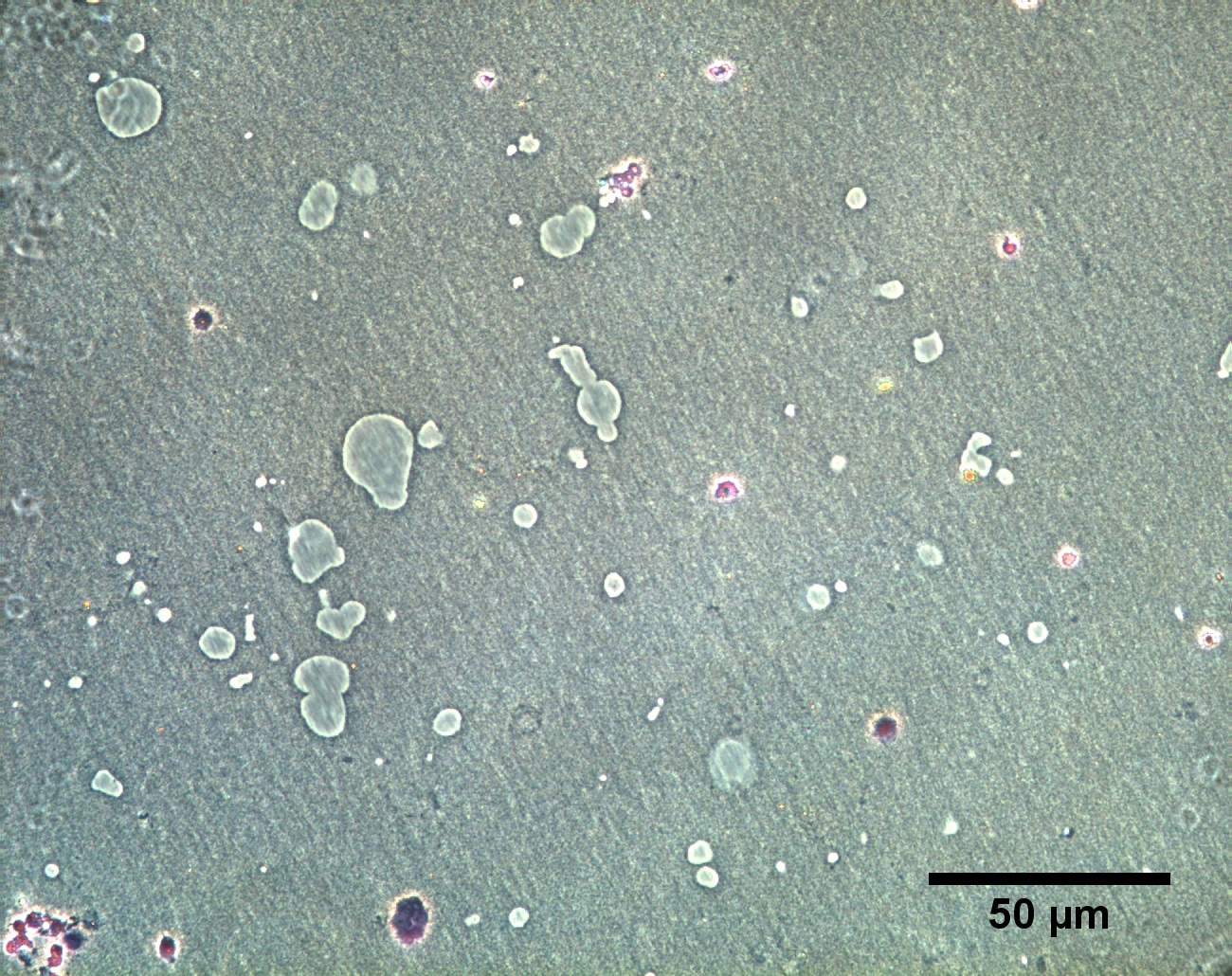

Supplement: Supplementary file 7 [file DataSheet2.ZIP › Fig.2-Source data/F/RANKL+5-O-Methyl Quercetin (10 a╠M).jpg]

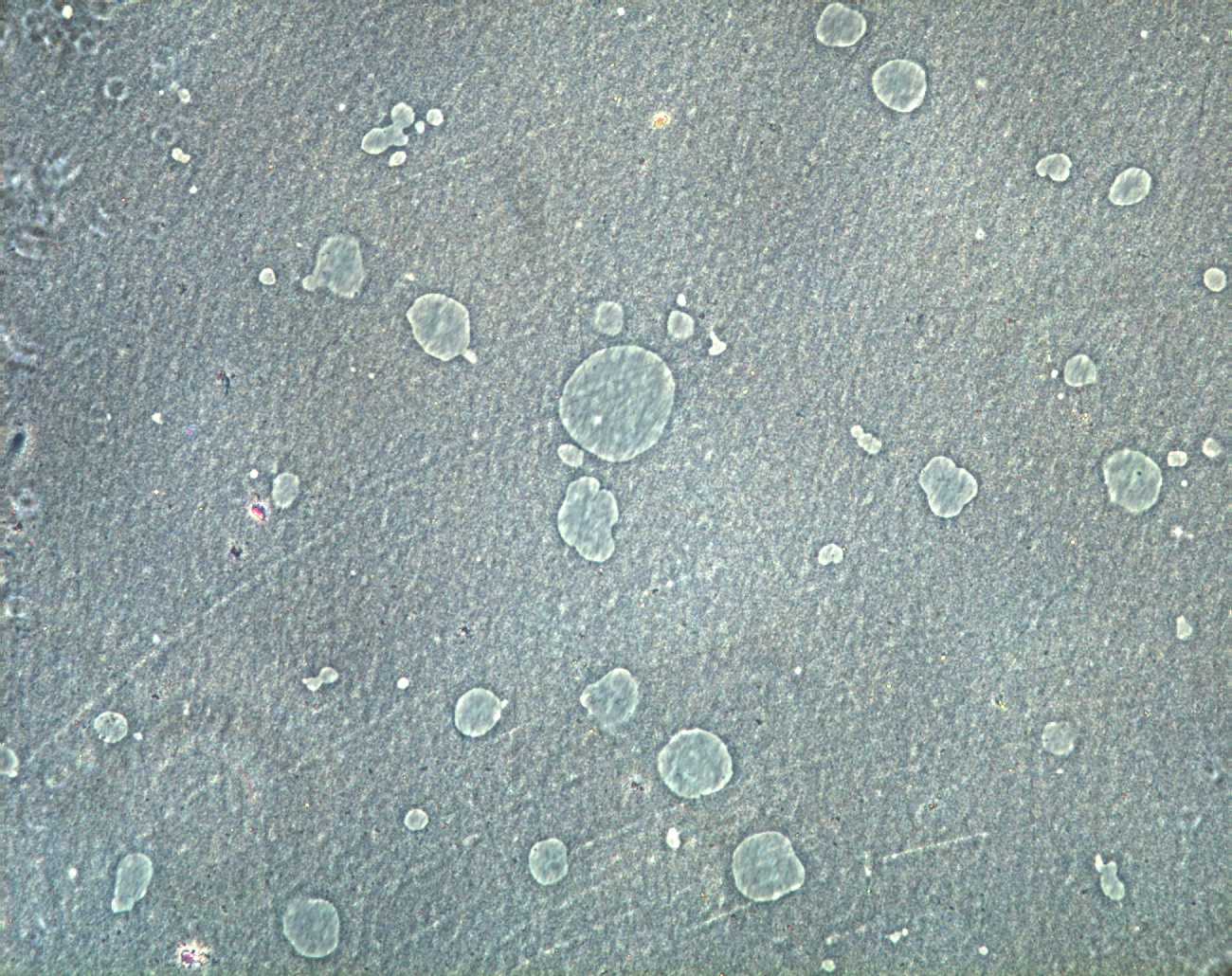

Supplement: Supplementary file 7 [file DataSheet2.ZIP › Fig.2-Source data/F/RANKL+5-O-Methyl Quercetin (5 a╠M).jpg]

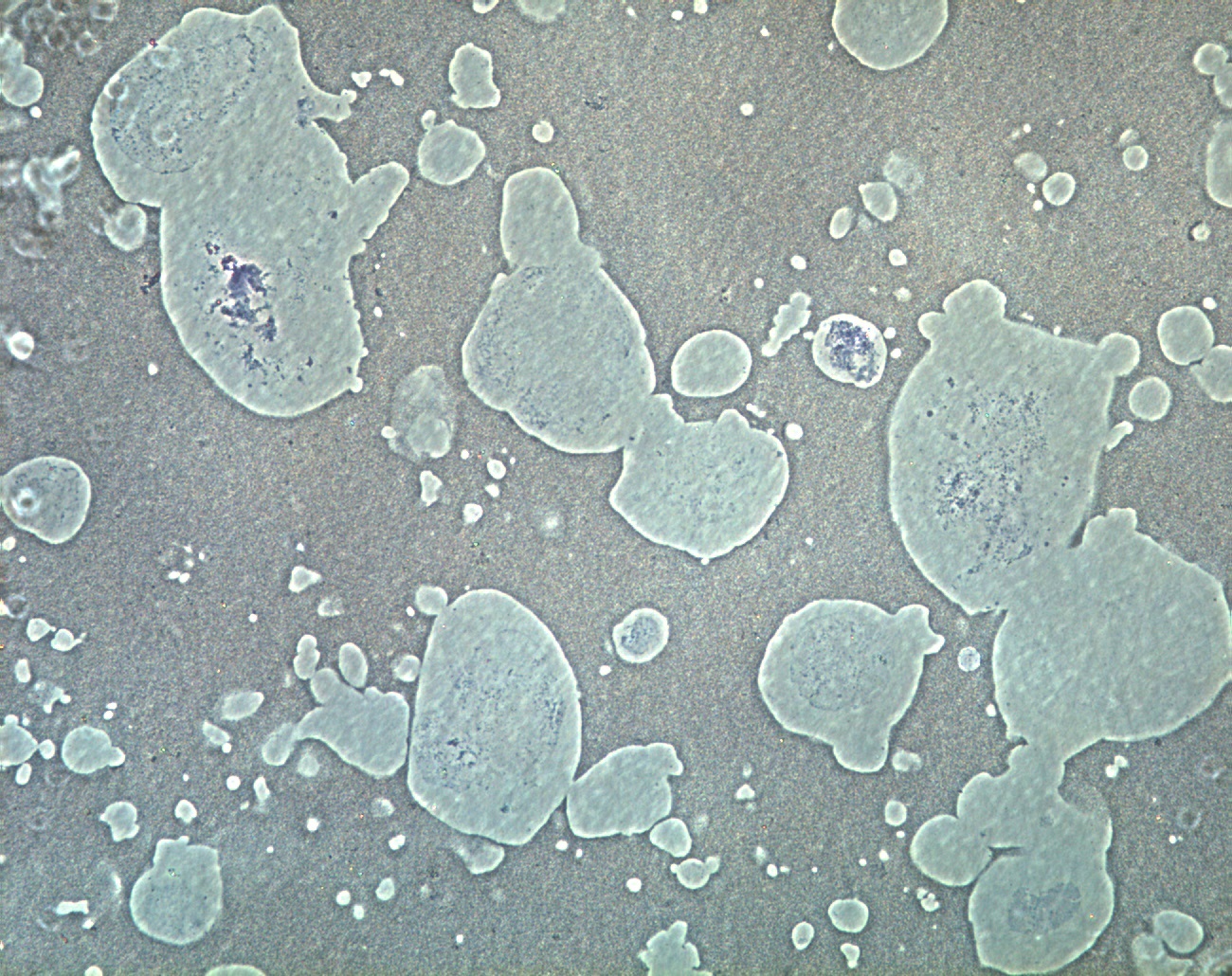

Supplement: Supplementary file 7 [file DataSheet2.ZIP › Fig.2-Source data/F/RANKL+ML171 (2 uM).jpg]

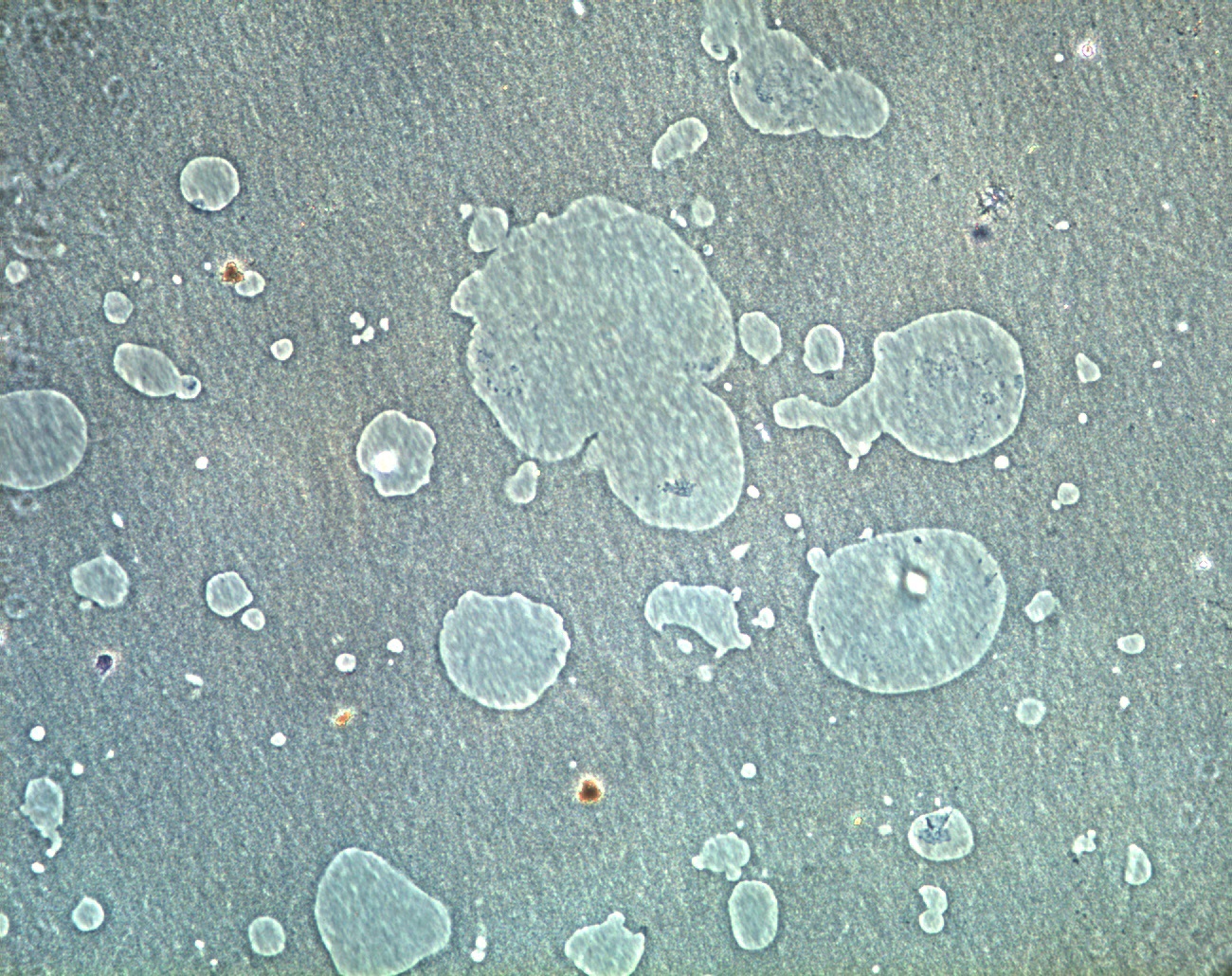

Supplement: Supplementary file 7 [file DataSheet2.ZIP › Fig.2-Source data/F/RANKL+ML171 (5 uM).jpg]

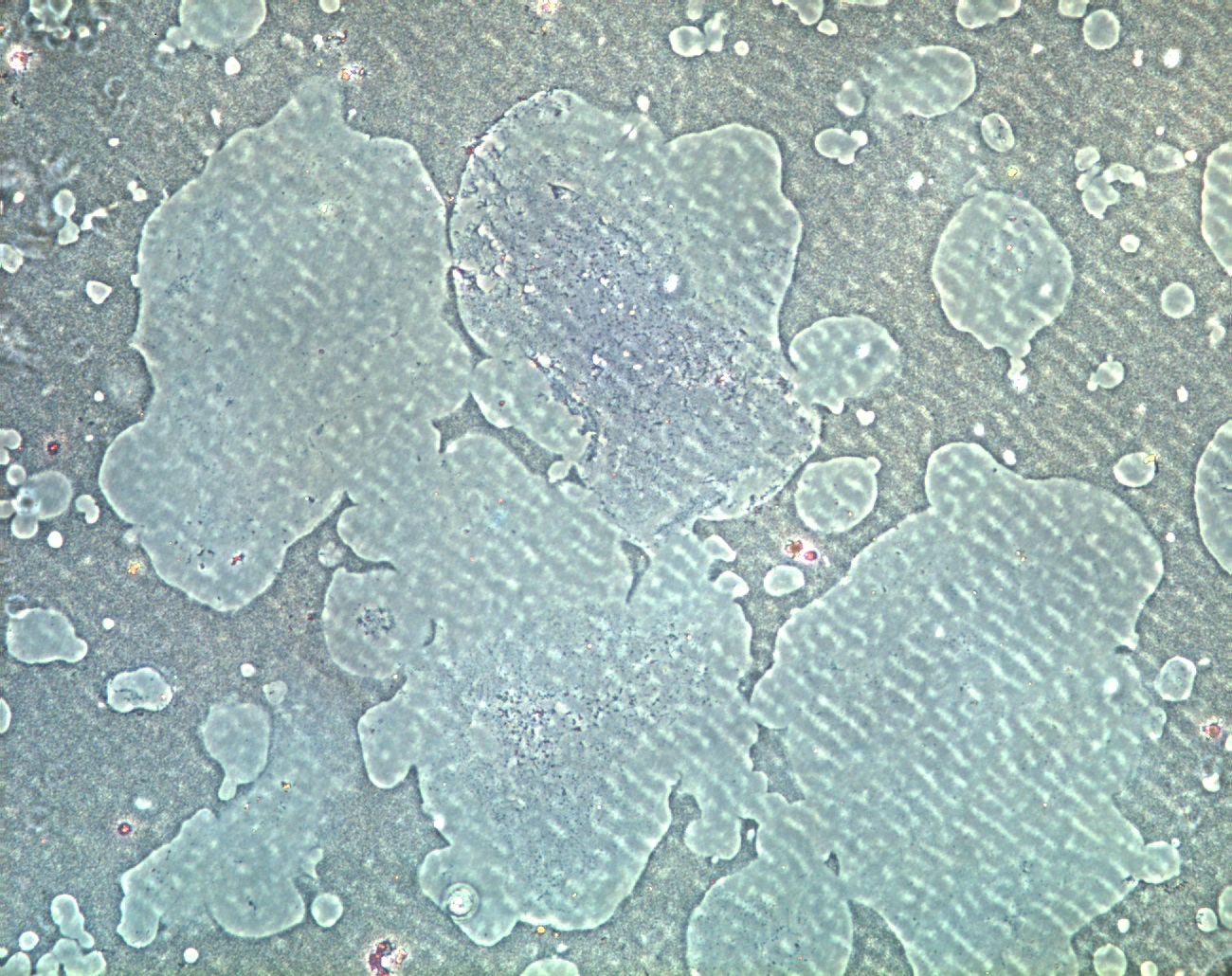

Supplement: Supplementary file 7 [file DataSheet2.ZIP › Fig.2-Source data/F/RANKL.jpg]

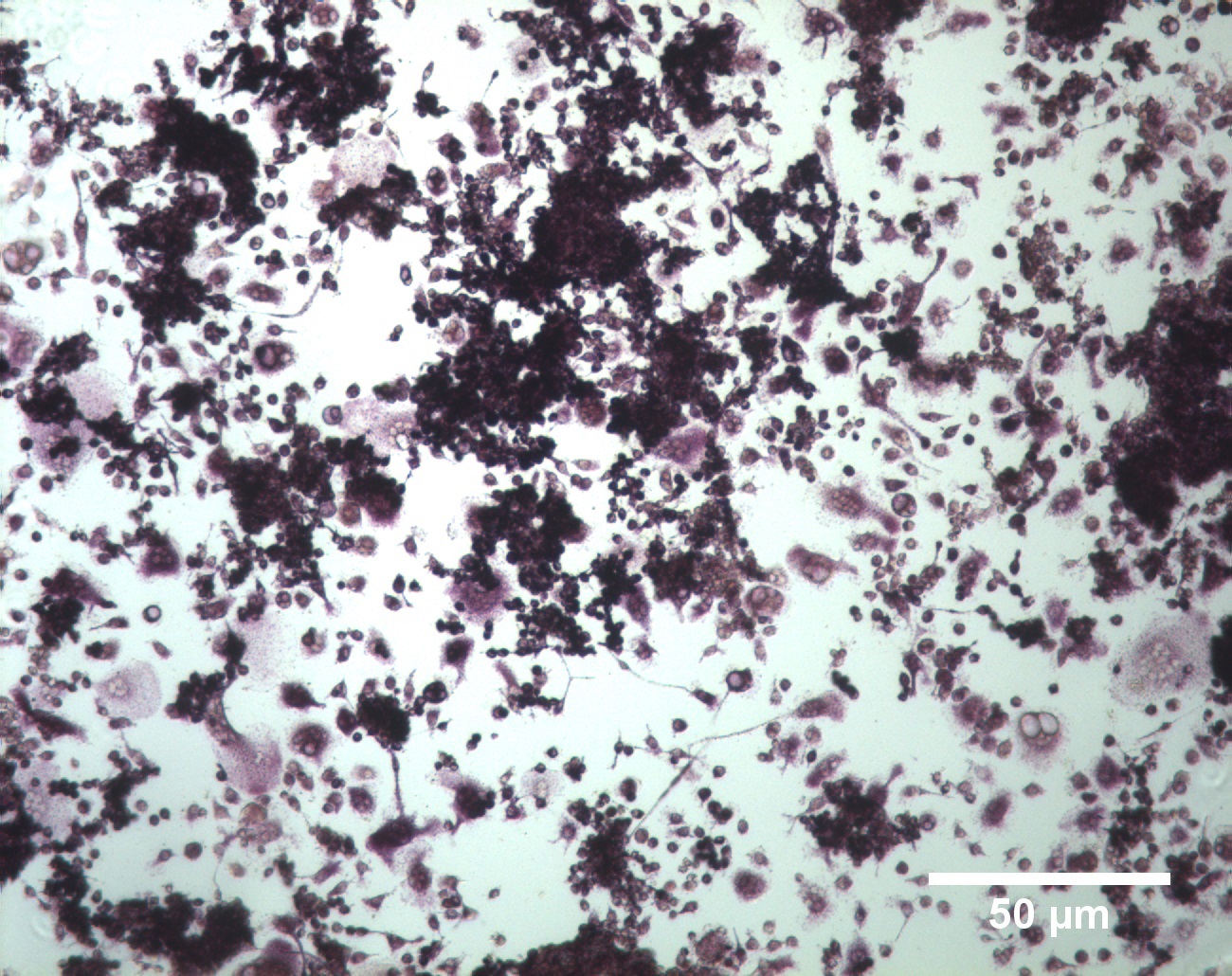

Supplement: Supplementary file 7 [file DataSheet2.ZIP › Fig.2-Source data/F/TRAP-RANKL+5-O-Methyl Quercetin (10 a╠M).jpg]

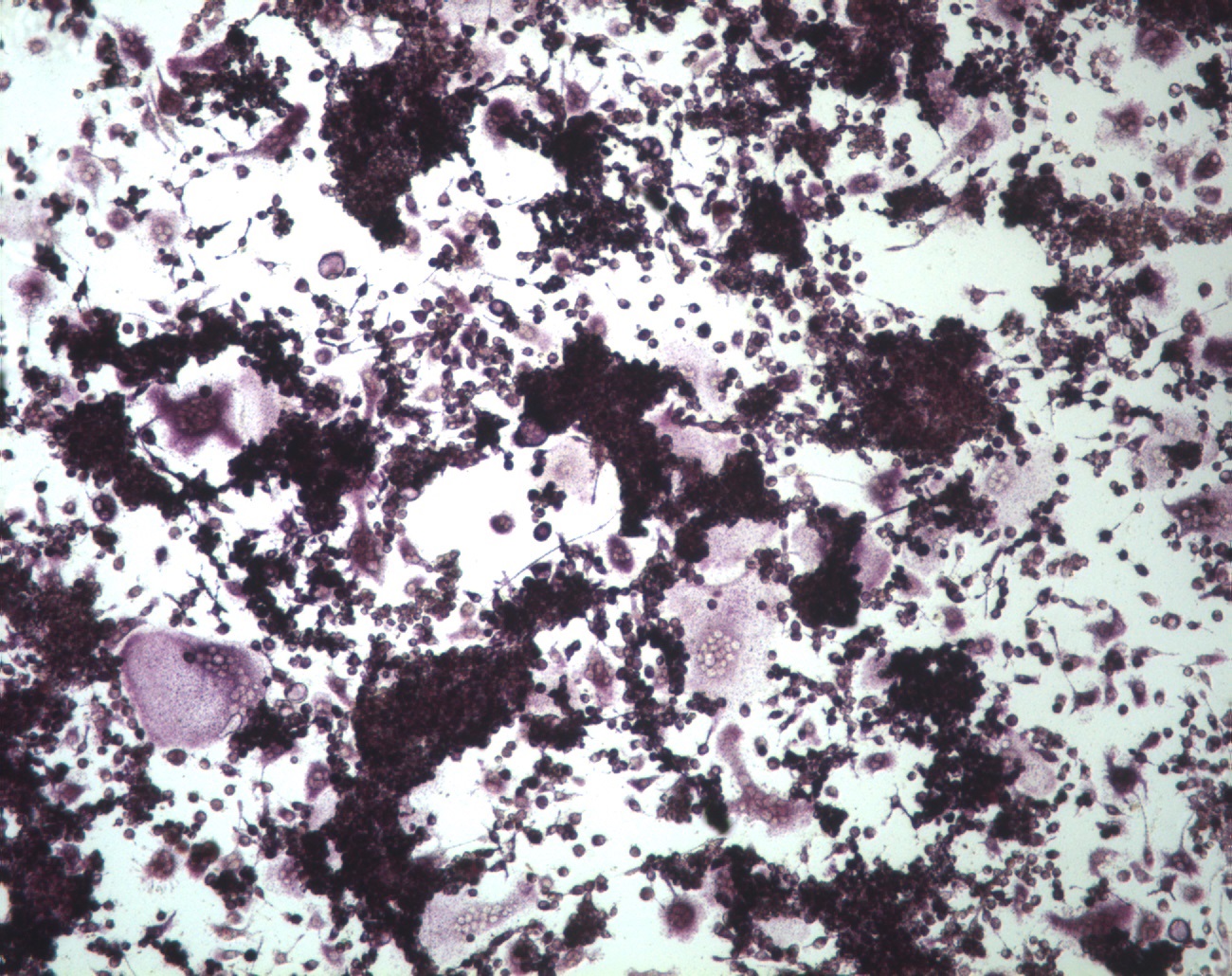

Supplement: Supplementary file 7 [file DataSheet2.ZIP › Fig.2-Source data/F/TRAP-RANKL+5-O-Methyl Quercetin (5 a╠M).jpg]

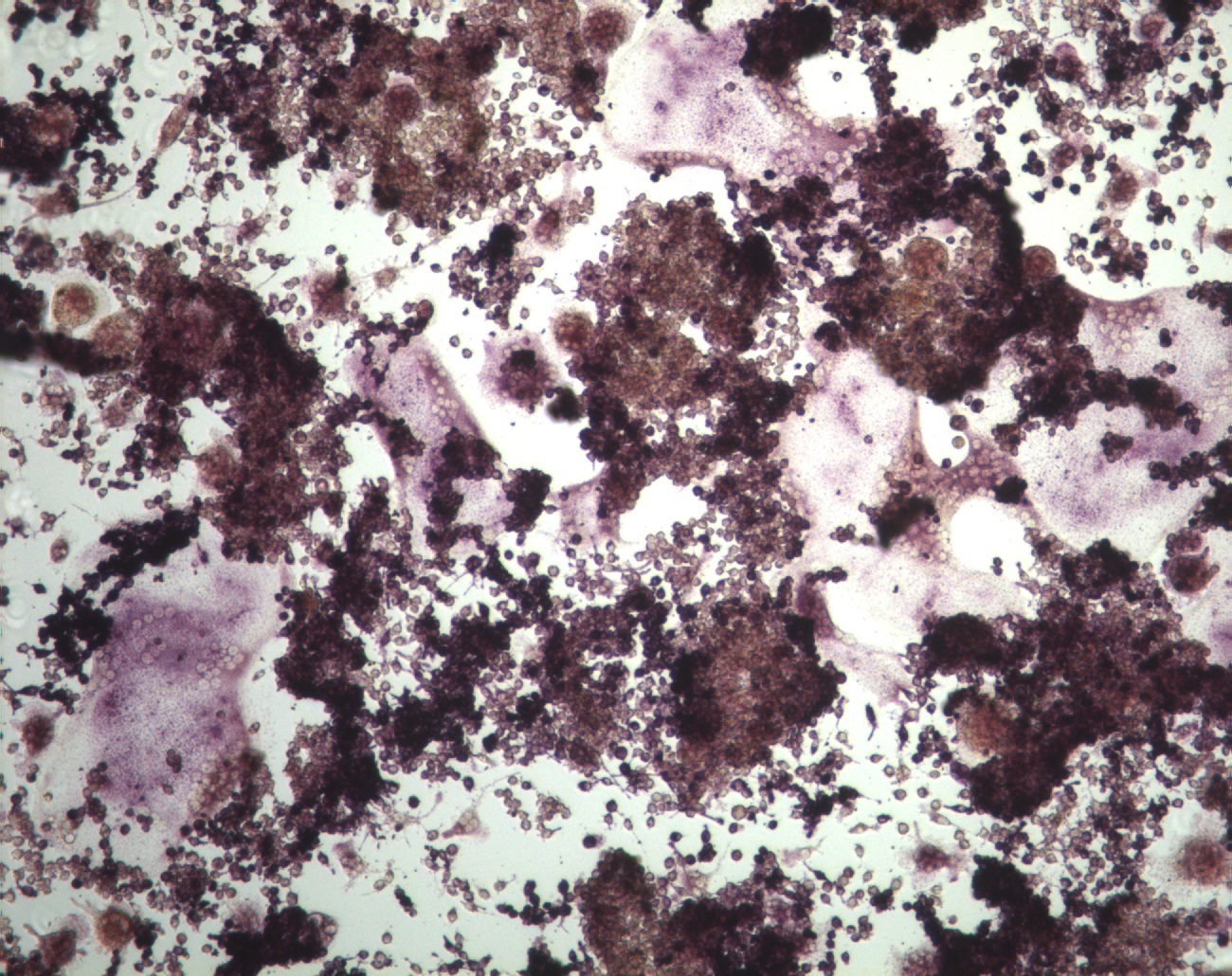

Supplement: Supplementary file 7 [file DataSheet2.ZIP › Fig.2-Source data/F/TRAP-RANKL+ML171 (2 uM).jpg]

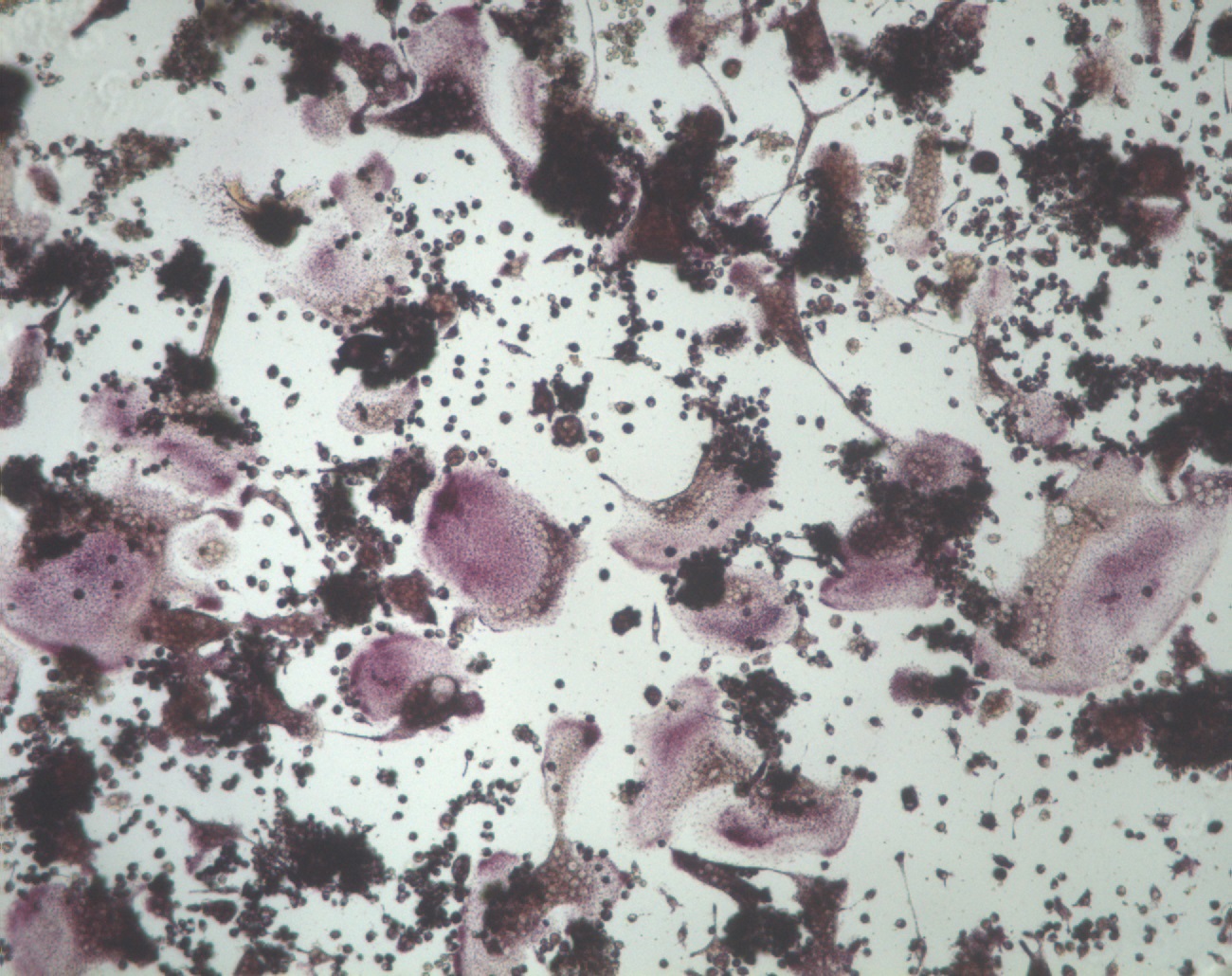

Supplement: Supplementary file 7 [file DataSheet2.ZIP › Fig.2-Source data/F/TRAP-RANKL+ML171 (5 uM).jpg]

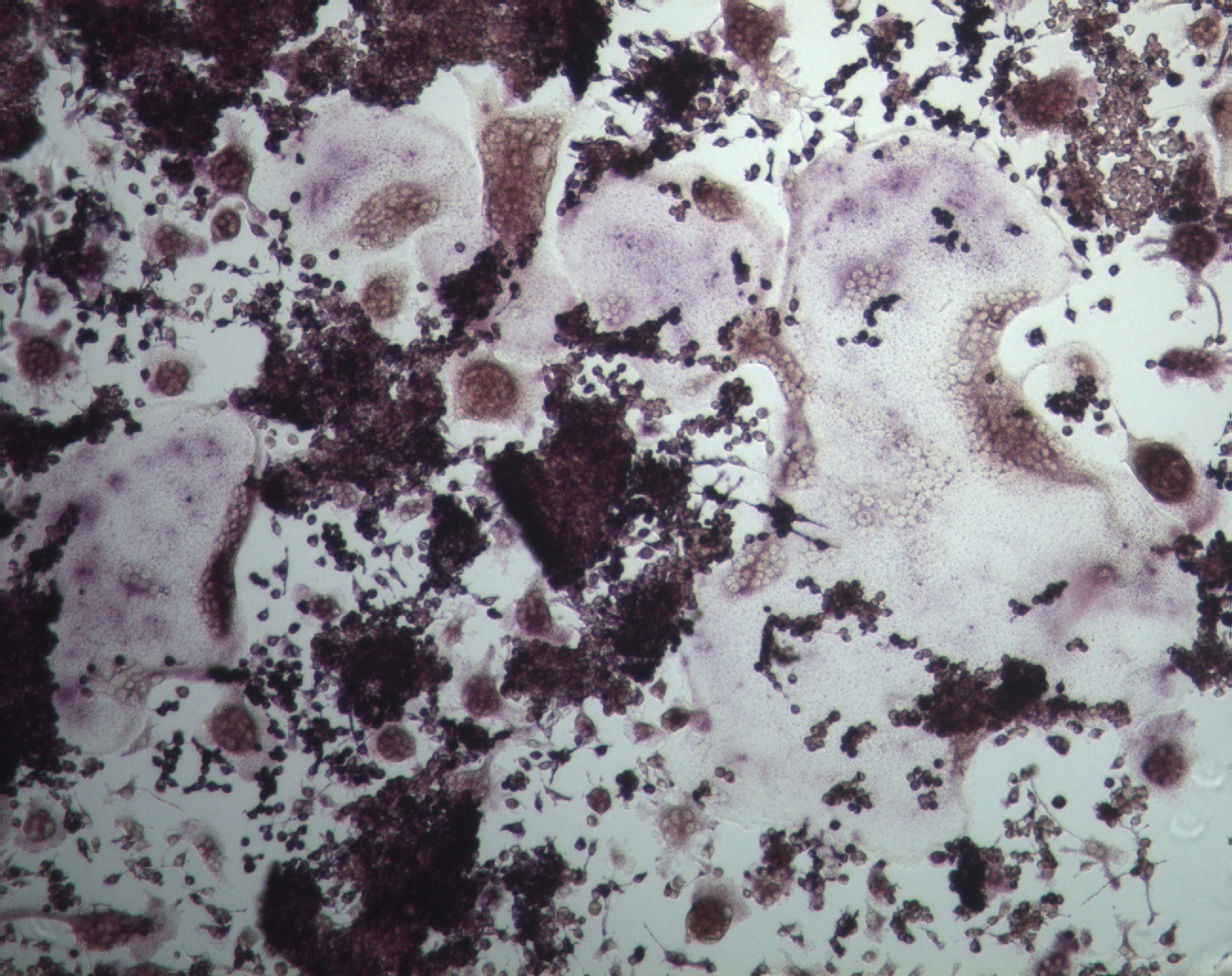

Supplement: Supplementary file 7 [file DataSheet2.ZIP › Fig.2-Source data/F/TRAP-RANKL.jpg]

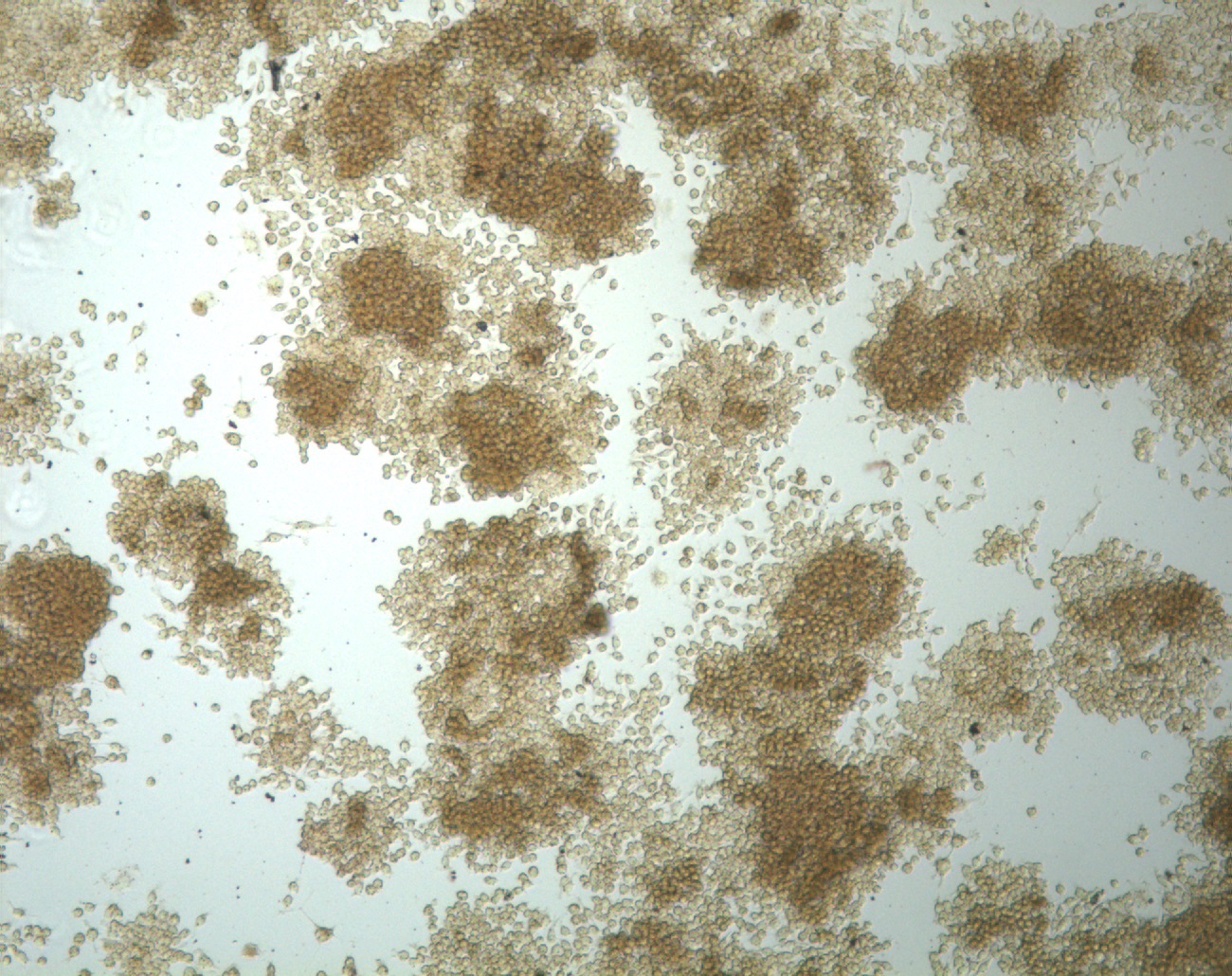

Supplement: Supplementary file 7 [file DataSheet2.ZIP › Fig.2-Source data/F/TRAP-cnotrol.jpg]

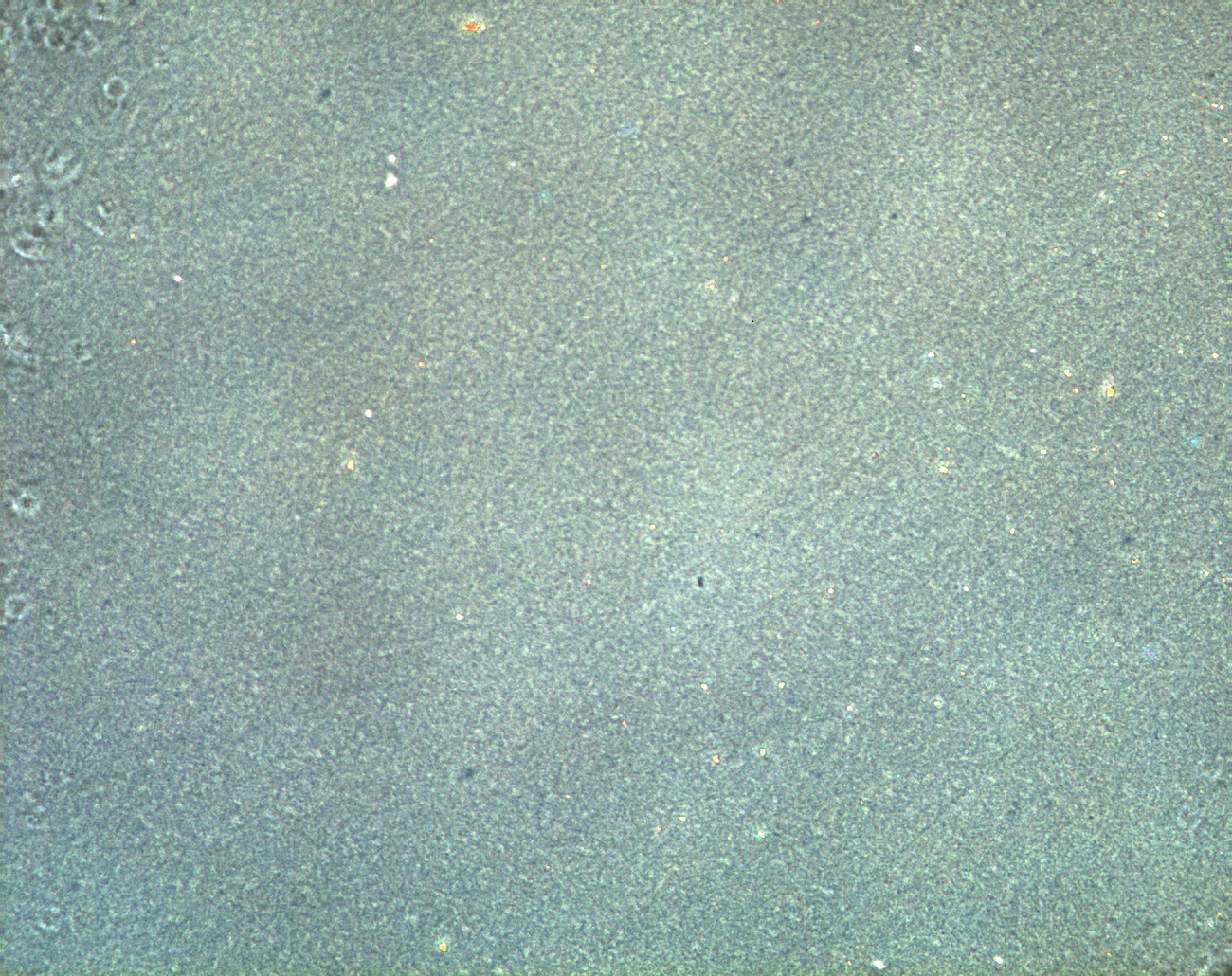

Supplement: Supplementary file 7 [file DataSheet2.ZIP › Fig.2-Source data/F/cnotrol.jpg]

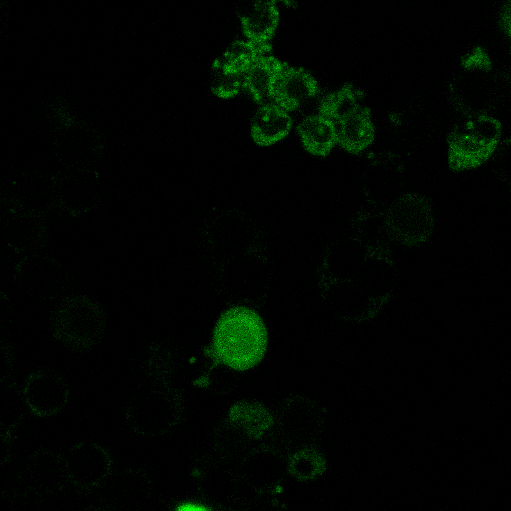

Supplement: Supplementary file 8 [file DataSheet5.ZIP › Fig.5-Source data/A/RANKL 1d-DCF Staining.tif]

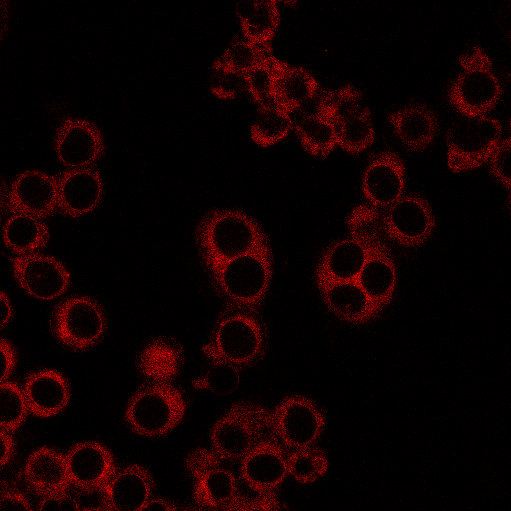

Supplement: Supplementary file 8 [file DataSheet5.ZIP › Fig.5-Source data/A/RANKL 1d-ER-Tracker.tif]

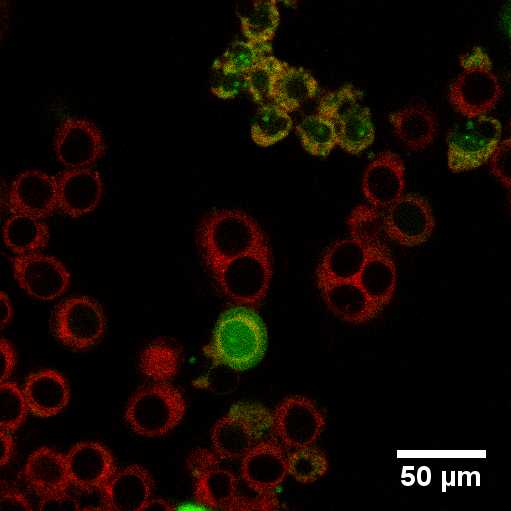

Supplement: Supplementary file 8 [file DataSheet5.ZIP › Fig.5-Source data/A/RANKL 1d-merged.tif]

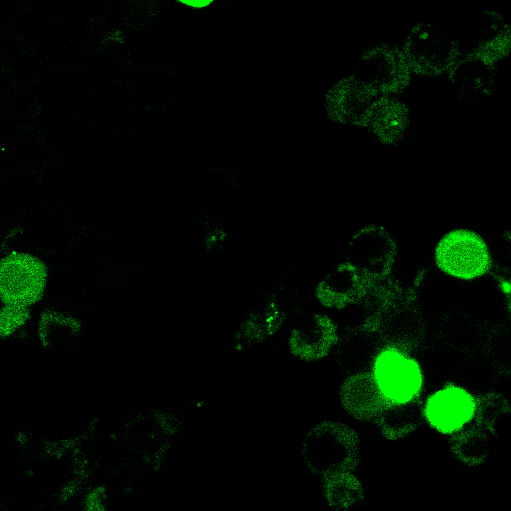

Supplement: Supplementary file 8 [file DataSheet5.ZIP › Fig.5-Source data/A/RANKL 2d-DCF Staining.tif]

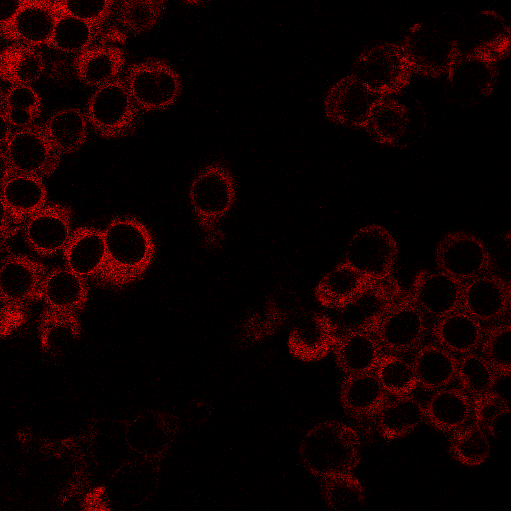

Supplement: Supplementary file 8 [file DataSheet5.ZIP › Fig.5-Source data/A/RANKL 2d-ER-Tracker.tif]

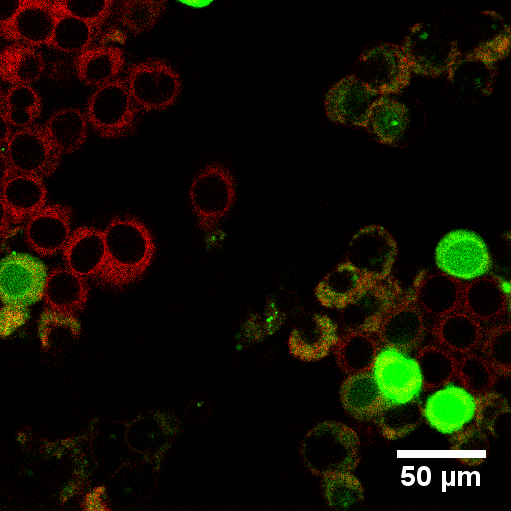

Supplement: Supplementary file 8 [file DataSheet5.ZIP › Fig.5-Source data/A/RANKL 2d-merged.tif]

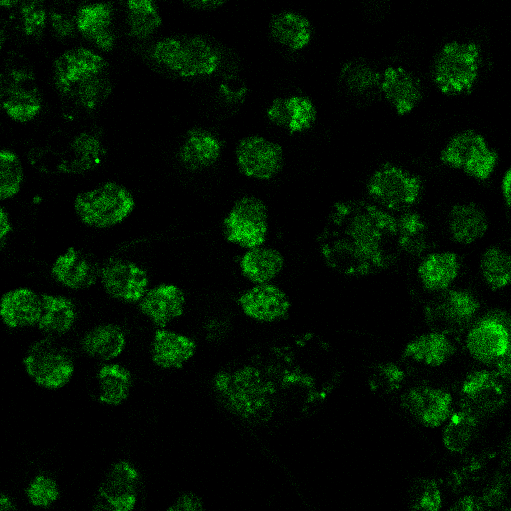

Supplement: Supplementary file 8 [file DataSheet5.ZIP › Fig.5-Source data/A/RANKL 3d-DCF Staining.tif]

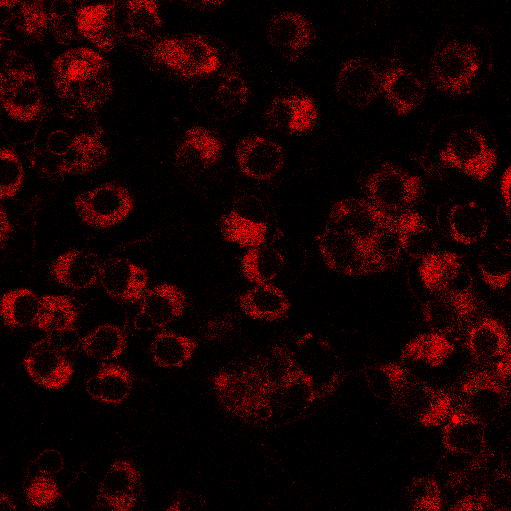

Supplement: Supplementary file 8 [file DataSheet5.ZIP › Fig.5-Source data/A/RANKL 3d-ER-Tracker.tif]

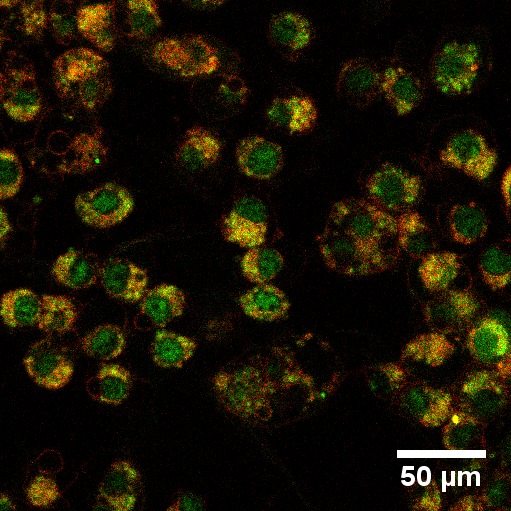

Supplement: Supplementary file 8 [file DataSheet5.ZIP › Fig.5-Source data/A/RANKL 3d-merged.tif]

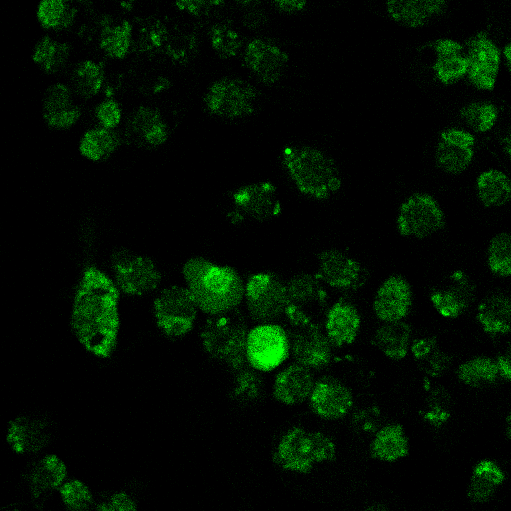

Supplement: Supplementary file 8 [file DataSheet5.ZIP › Fig.5-Source data/A/RANKL+sh-NC 3d-DCF Staining.tif]

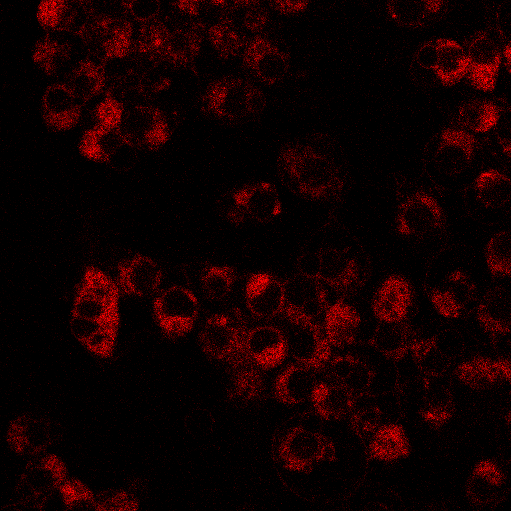

Supplement: Supplementary file 8 [file DataSheet5.ZIP › Fig.5-Source data/A/RANKL+sh-NC 3d-ER-Tracker.tif]

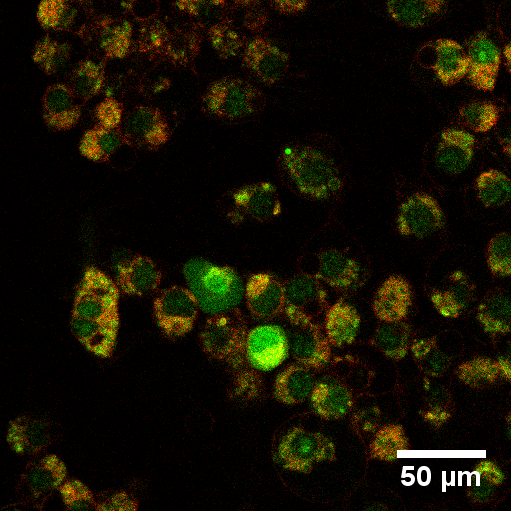

Supplement: Supplementary file 8 [file DataSheet5.ZIP › Fig.5-Source data/A/RANKL+sh-NC 3d-merged.tif]

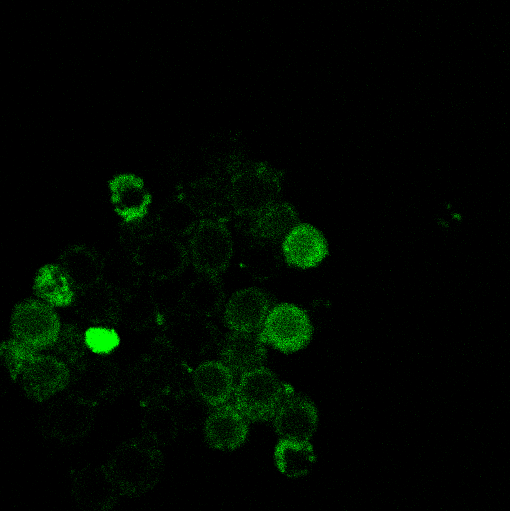

Supplement: Supplementary file 8 [file DataSheet5.ZIP › Fig.5-Source data/A/RANKL+sh-Nox4 3d-DCF Staining.tif]

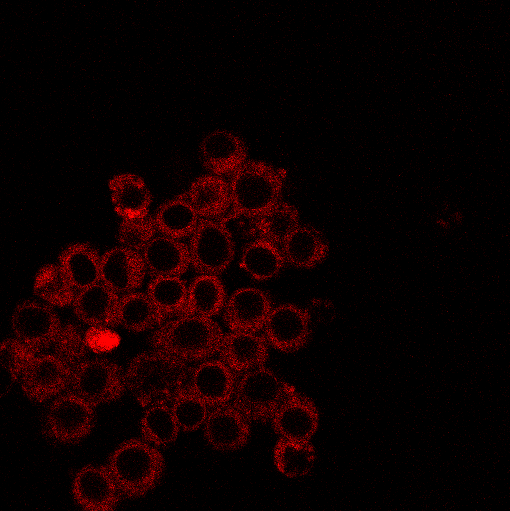

Supplement: Supplementary file 8 [file DataSheet5.ZIP › Fig.5-Source data/A/RANKL+sh-Nox4 3d-ER-Tracker.tif]

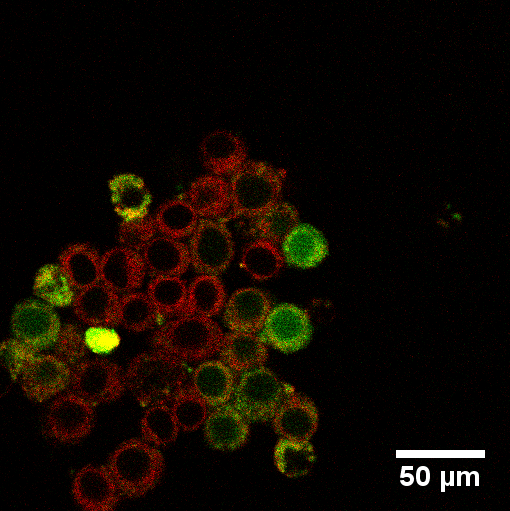

Supplement: Supplementary file 8 [file DataSheet5.ZIP › Fig.5-Source data/A/RANKL+sh-Nox4 3d-merged.tif]

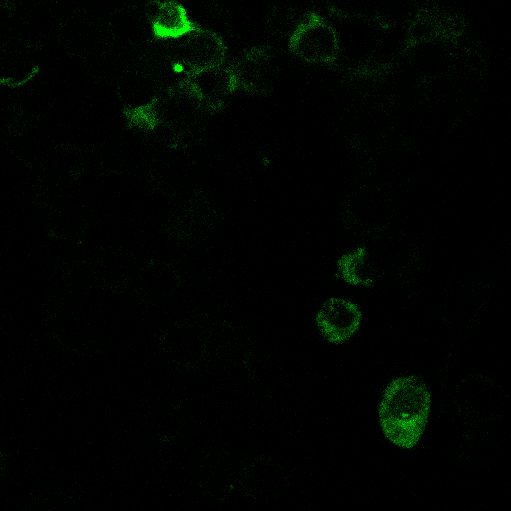

Supplement: Supplementary file 8 [file DataSheet5.ZIP › Fig.5-Source data/A/control 1d-DCF Staining.tif]

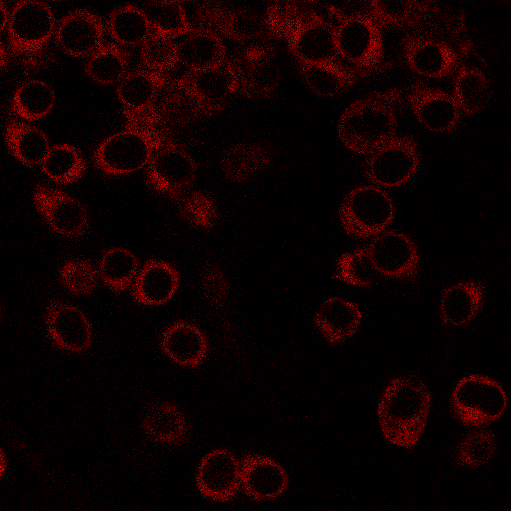

Supplement: Supplementary file 8 [file DataSheet5.ZIP › Fig.5-Source data/A/control 1d-ER-Tracker.tif]

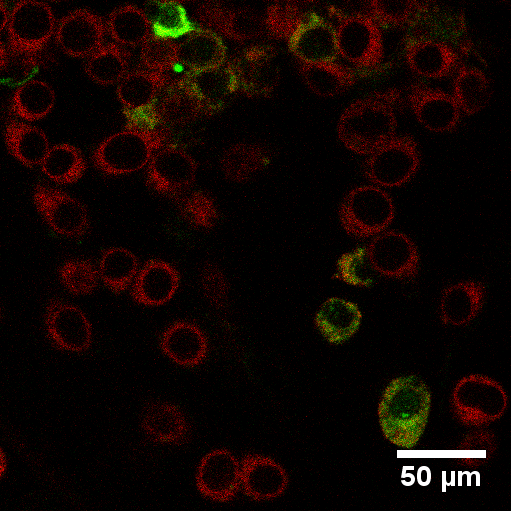

Supplement: Supplementary file 8 [file DataSheet5.ZIP › Fig.5-Source data/A/control 1d-merged.tif]

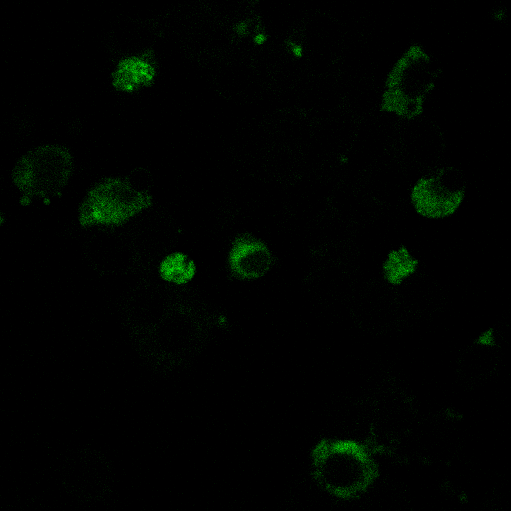

Supplement: Supplementary file 8 [file DataSheet5.ZIP › Fig.5-Source data/A/control 2d-DCF Staining.tif]

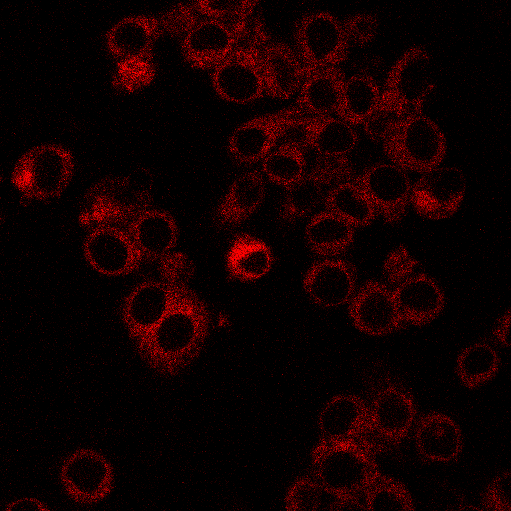

Supplement: Supplementary file 8 [file DataSheet5.ZIP › Fig.5-Source data/A/control 2d-ER-Tracker.tif]

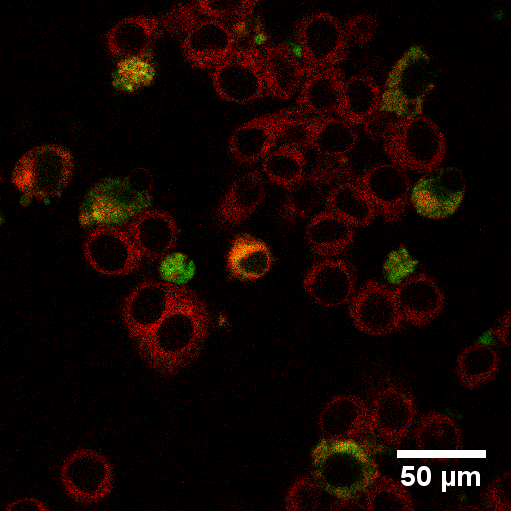

Supplement: Supplementary file 8 [file DataSheet5.ZIP › Fig.5-Source data/A/control 2d-merged.tif]

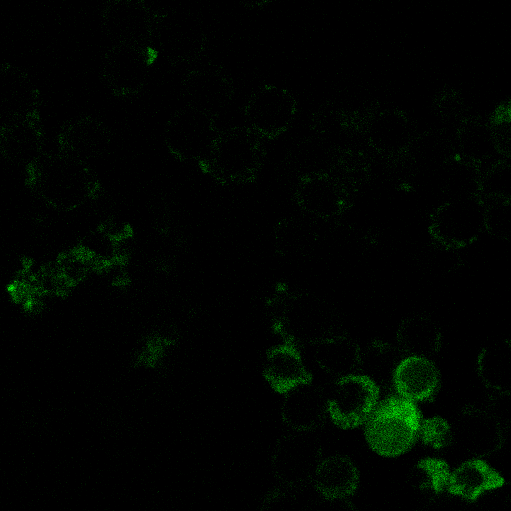

Supplement: Supplementary file 8 [file DataSheet5.ZIP › Fig.5-Source data/A/control 3d-DCF Staining.tif]

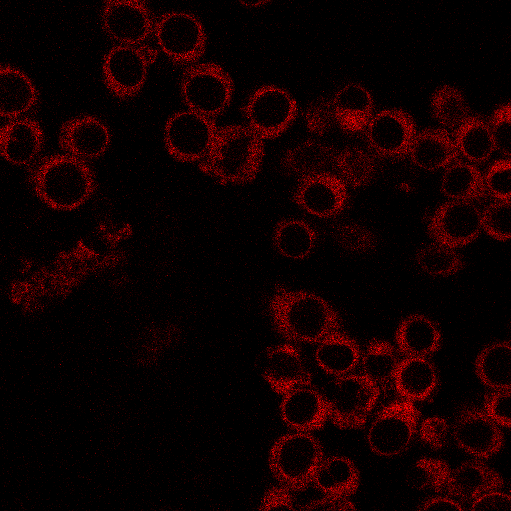

Supplement: Supplementary file 8 [file DataSheet5.ZIP › Fig.5-Source data/A/control 3d-ER-Tracker.tif]

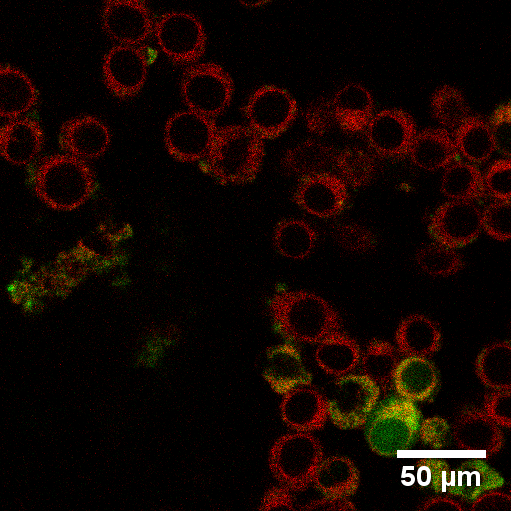

Supplement: Supplementary file 8 [file DataSheet5.ZIP › Fig.5-Source data/A/control 3d-merged.tif]

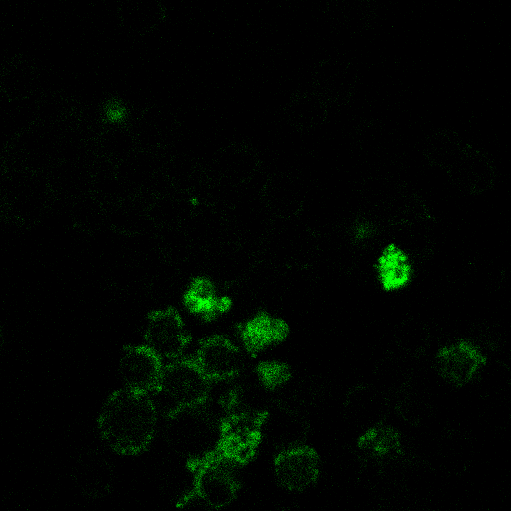

Supplement: Supplementary file 8 [file DataSheet5.ZIP › Fig.5-Source data/B/RANKL+NAC (10 mM)-DCF Staining.tif]

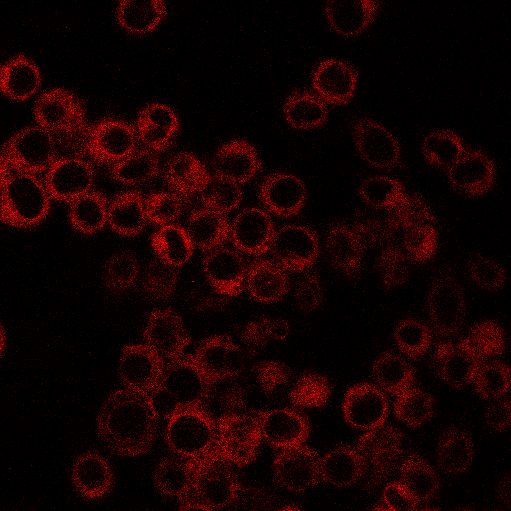

Supplement: Supplementary file 8 [file DataSheet5.ZIP › Fig.5-Source data/B/RANKL+NAC (10 mM)-ER-Tracker.tif]

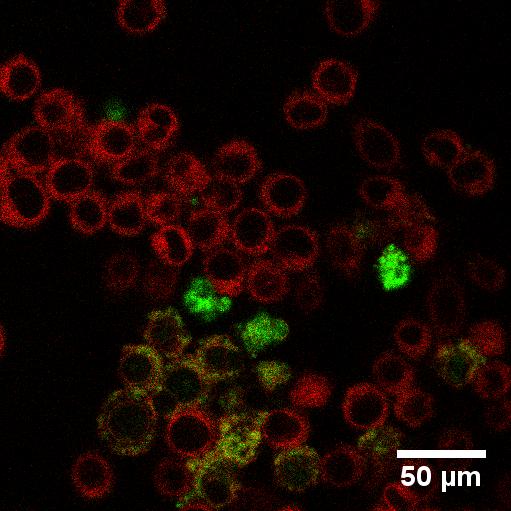

Supplement: Supplementary file 8 [file DataSheet5.ZIP › Fig.5-Source data/B/RANKL+NAC (10 mM)-merged.tif]

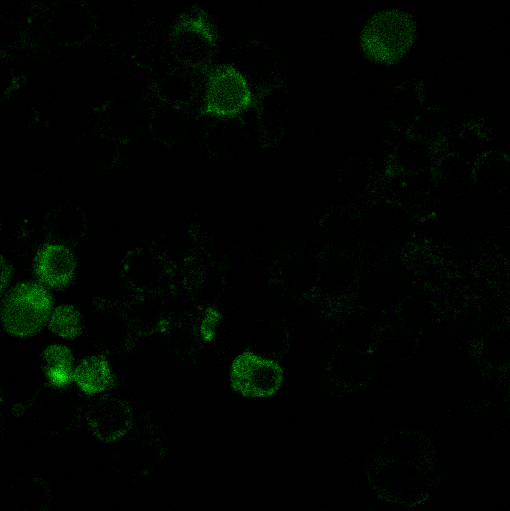

Supplement: Supplementary file 8 [file DataSheet5.ZIP › Fig.5-Source data/B/RANKL+NAC (20 mM)-DCF Staining.tif]

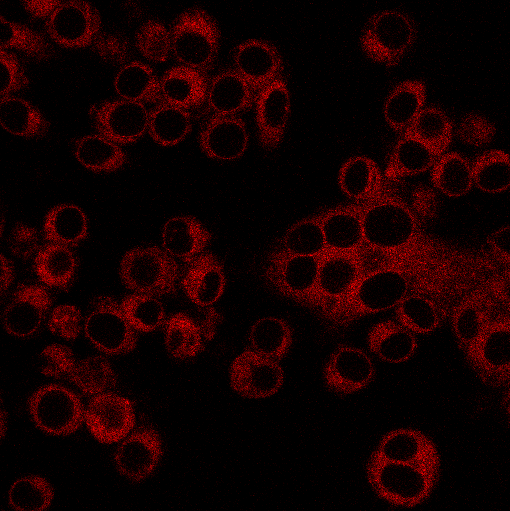

Supplement: Supplementary file 8 [file DataSheet5.ZIP › Fig.5-Source data/B/RANKL+NAC (20 mM)-ER-Tracker.tif]

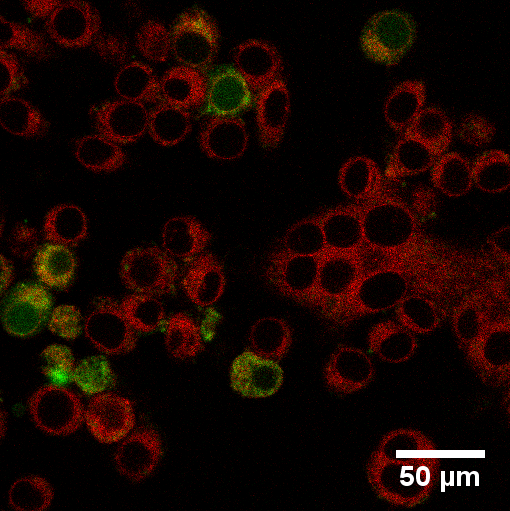

Supplement: Supplementary file 8 [file DataSheet5.ZIP › Fig.5-Source data/B/RANKL+NAC (20 mM)-merged.tif]

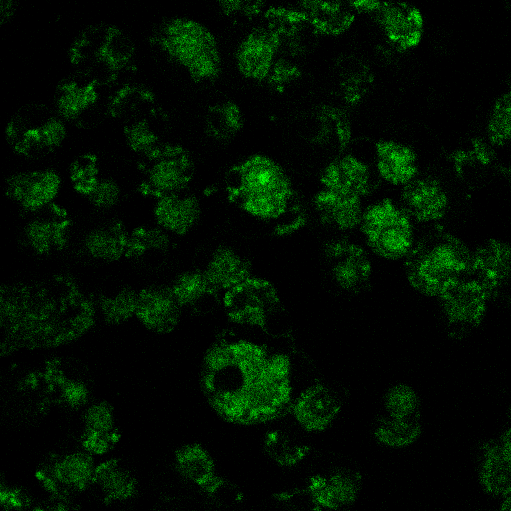

Supplement: Supplementary file 8 [file DataSheet5.ZIP › Fig.5-Source data/B/RANKL-DCF Staining.tif]

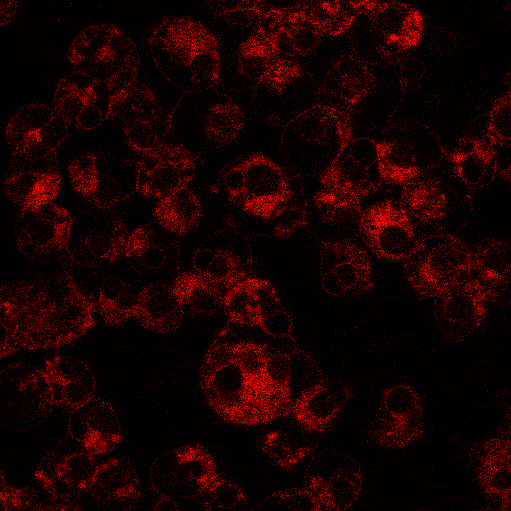

Supplement: Supplementary file 8 [file DataSheet5.ZIP › Fig.5-Source data/B/RANKL-ER-Tracker.tif]

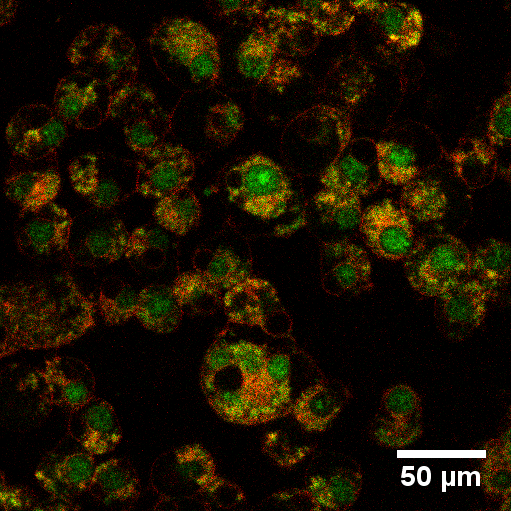

Supplement: Supplementary file 8 [file DataSheet5.ZIP › Fig.5-Source data/B/RANKL-merged.tif]

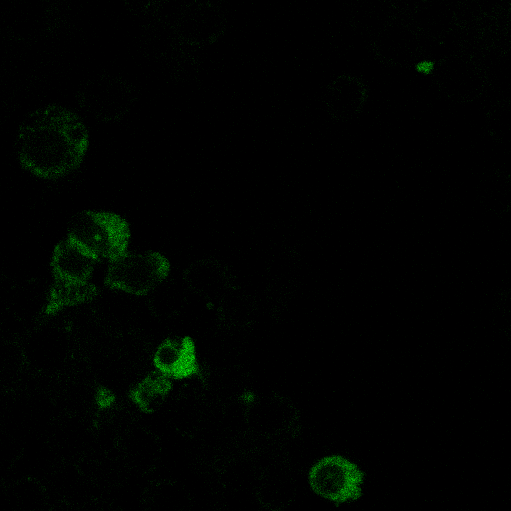

Supplement: Supplementary file 8 [file DataSheet5.ZIP › Fig.5-Source data/B/control-DCF Staining.tif]

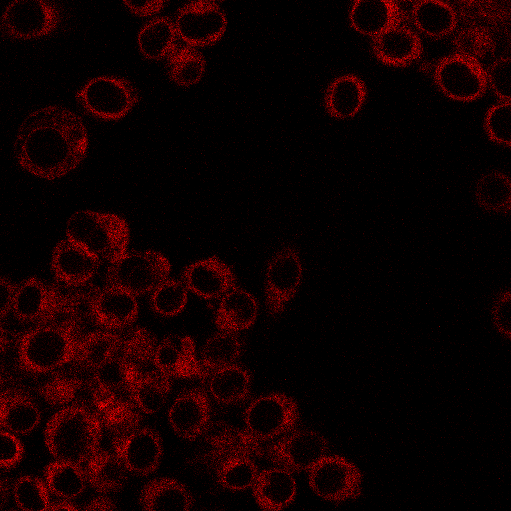

Supplement: Supplementary file 8 [file DataSheet5.ZIP › Fig.5-Source data/B/control-ER-Tracker.tif]

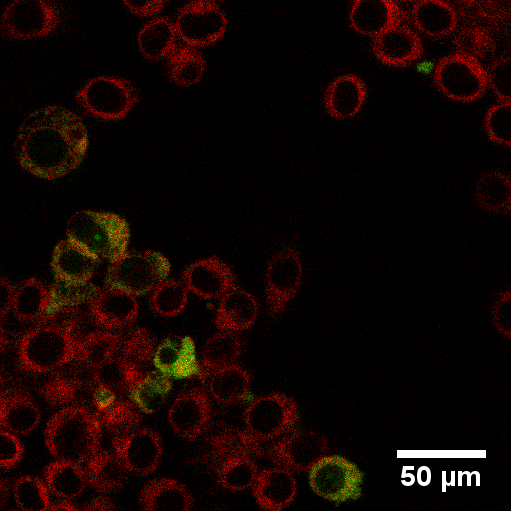

Supplement: Supplementary file 8 [file DataSheet5.ZIP › Fig.5-Source data/B/control-merged.tif]

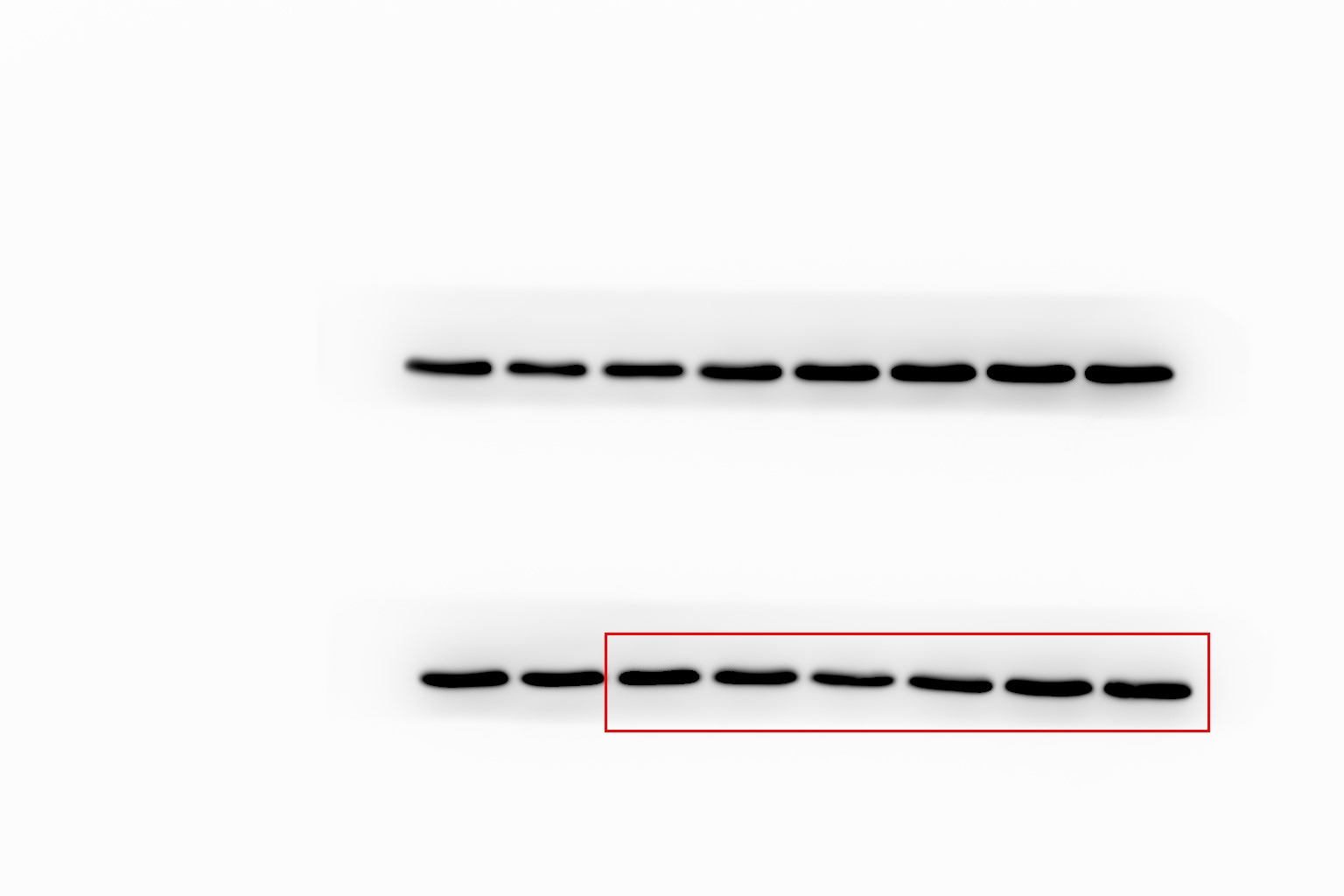

Supplement: Supplementary file 8 [file DataSheet5.ZIP › Fig.5-Source data/D/GAPDH.jpg]

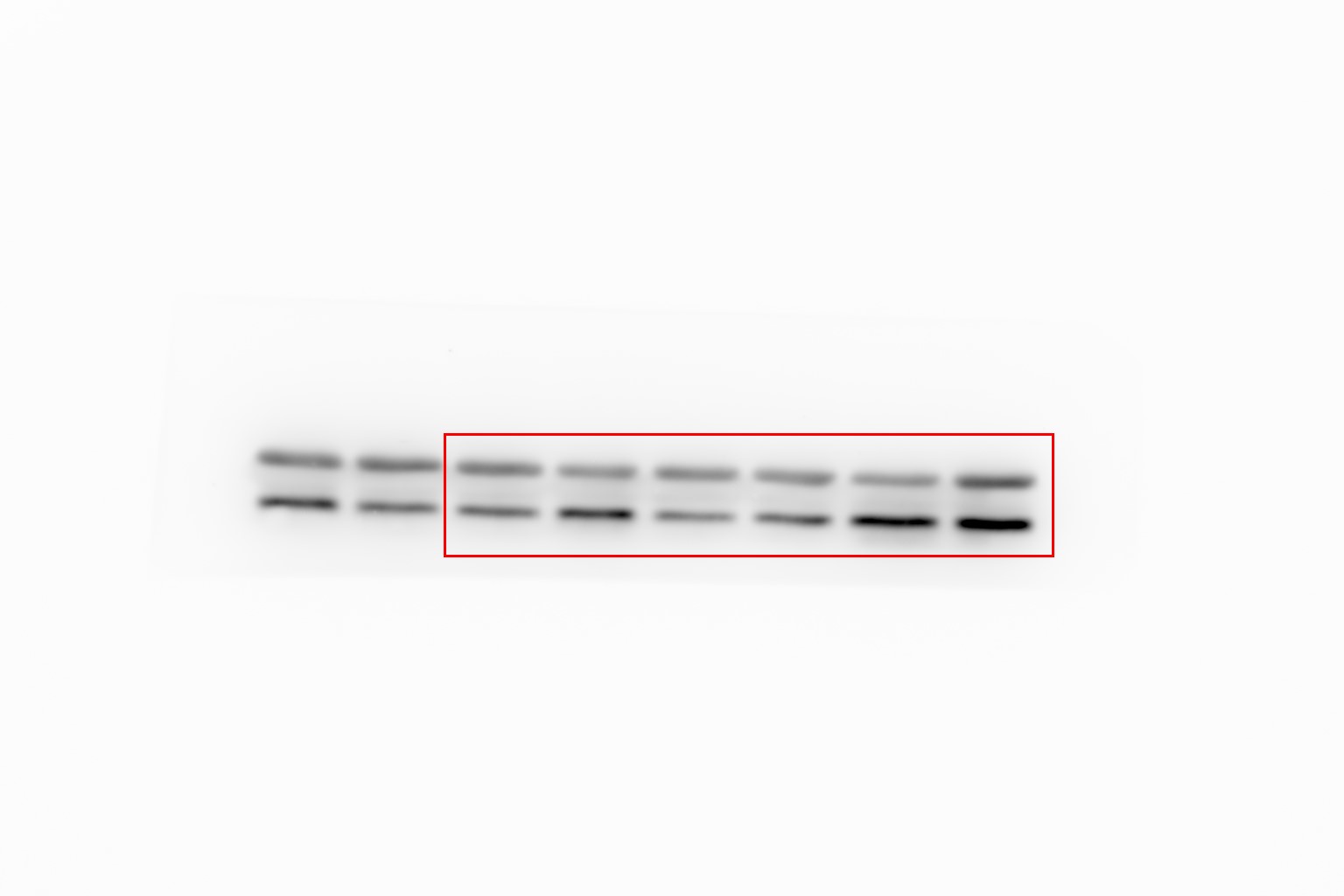

Supplement: Supplementary file 8 [file DataSheet5.ZIP › Fig.5-Source data/D/LC3.jpg]

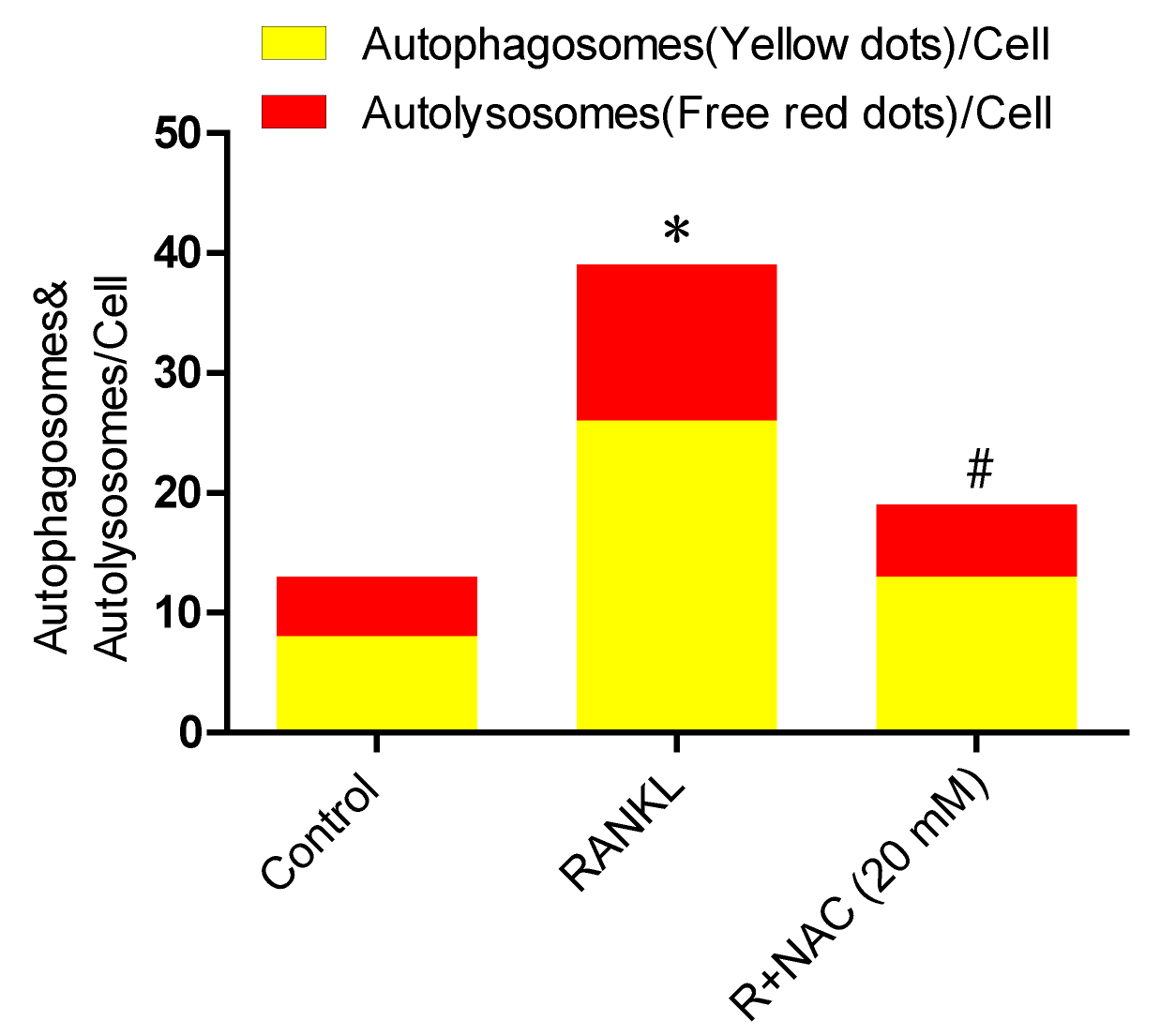

Supplement: Supplementary file 8 [file DataSheet5.ZIP › Fig.5-Source data/E/Fig.5E.tif]

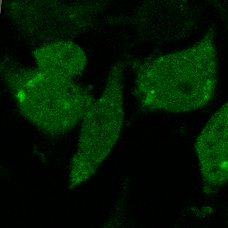

Supplement: Supplementary file 8 [file DataSheet5.ZIP › Fig.5-Source data/E/RANKL+NAC-GFP.tif]

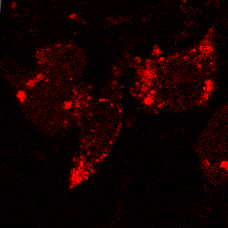

Supplement: Supplementary file 8 [file DataSheet5.ZIP › Fig.5-Source data/E/RANKL+NAC-mRFP.tif]

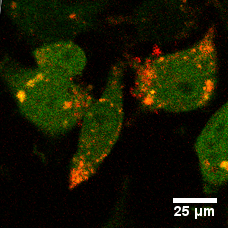

Supplement: Supplementary file 8 [file DataSheet5.ZIP › Fig.5-Source data/E/RANKL+NAC-mereged.tif]

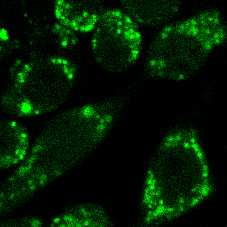

Supplement: Supplementary file 8 [file DataSheet5.ZIP › Fig.5-Source data/E/RANKL-GFP.tif]

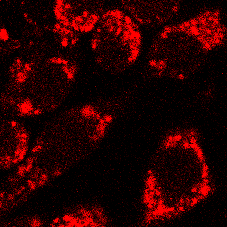

Supplement: Supplementary file 8 [file DataSheet5.ZIP › Fig.5-Source data/E/RANKL-mRFP.tif]

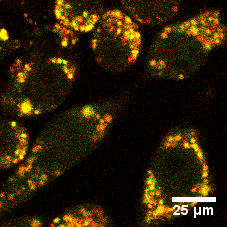

Supplement: Supplementary file 8 [file DataSheet5.ZIP › Fig.5-Source data/E/RANKL-merged.tif]

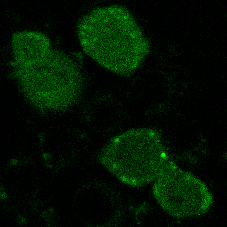

Supplement: Supplementary file 8 [file DataSheet5.ZIP › Fig.5-Source data/E/control-GFP.tif]

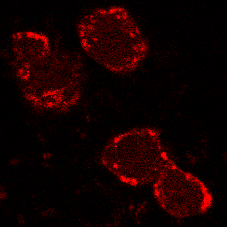

Supplement: Supplementary file 8 [file DataSheet5.ZIP › Fig.5-Source data/E/control-mRFP.tif]

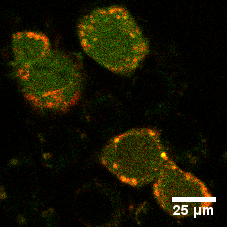

Supplement: Supplementary file 8 [file DataSheet5.ZIP › Fig.5-Source data/E/control-merged.tif]

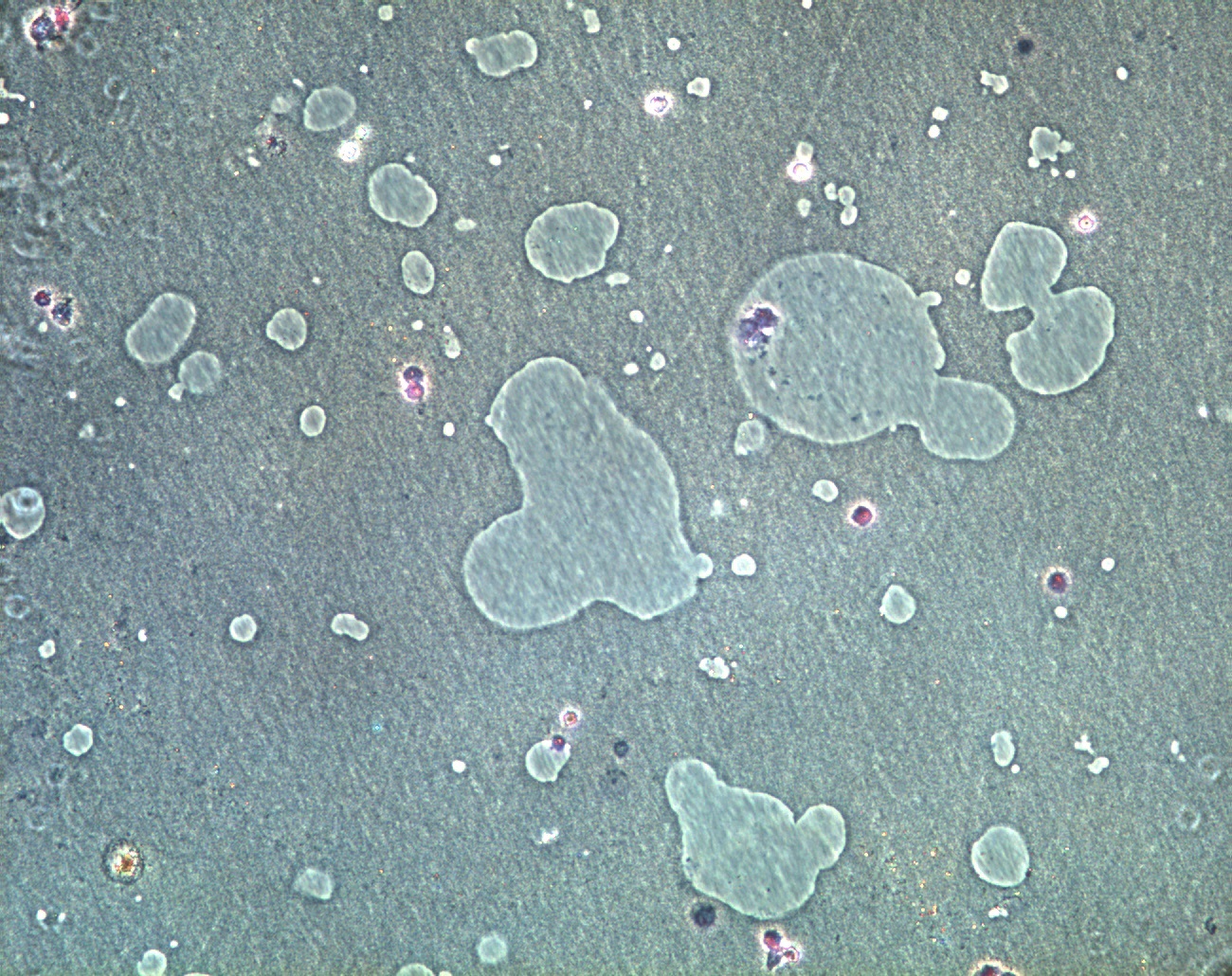

Supplement: Supplementary file 8 [file DataSheet5.ZIP › Fig.5-Source data/G/RANKL+NAC (10 mM).jpg]

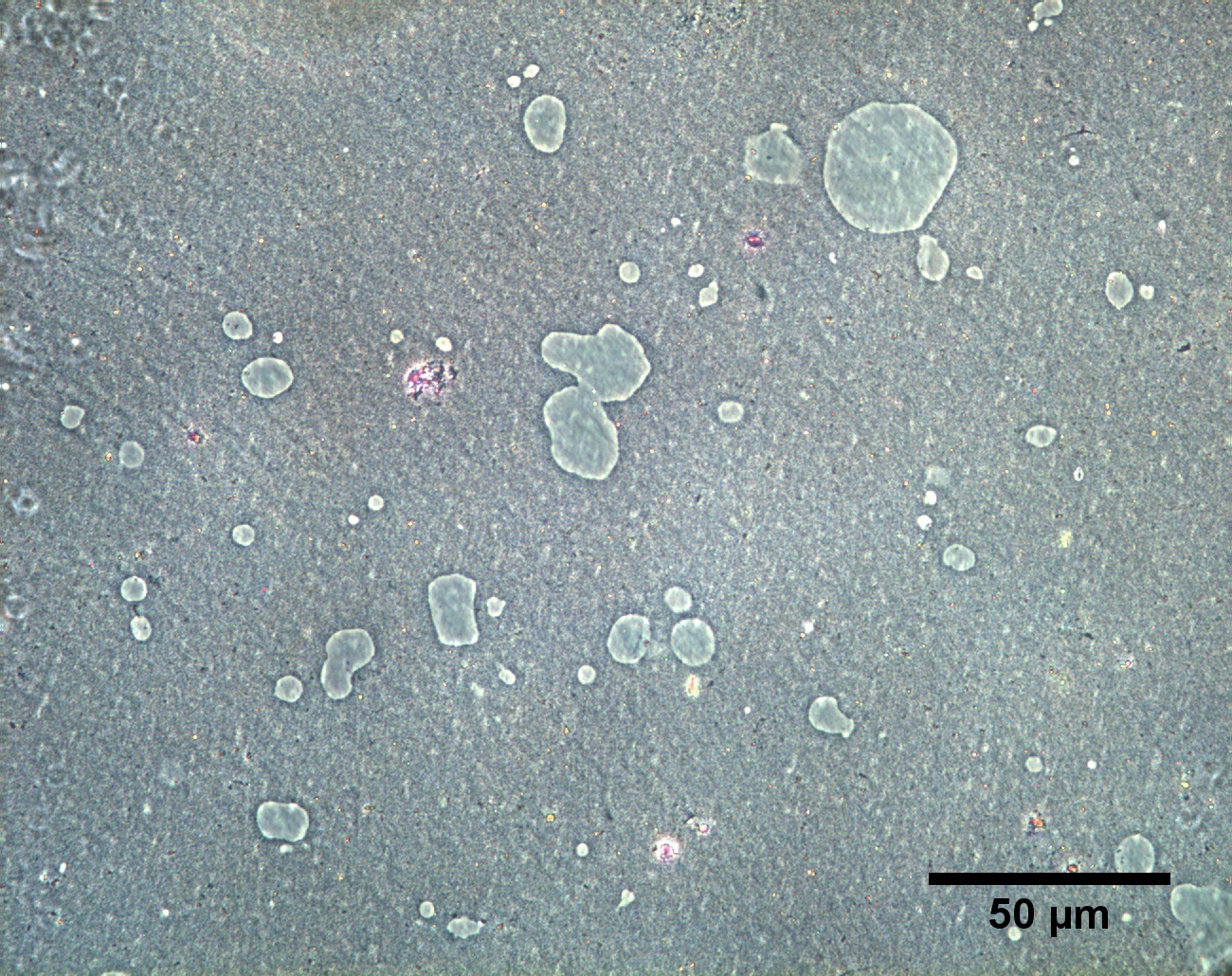

Supplement: Supplementary file 8 [file DataSheet5.ZIP › Fig.5-Source data/G/RANKL+NAC (20 mM).jpg]

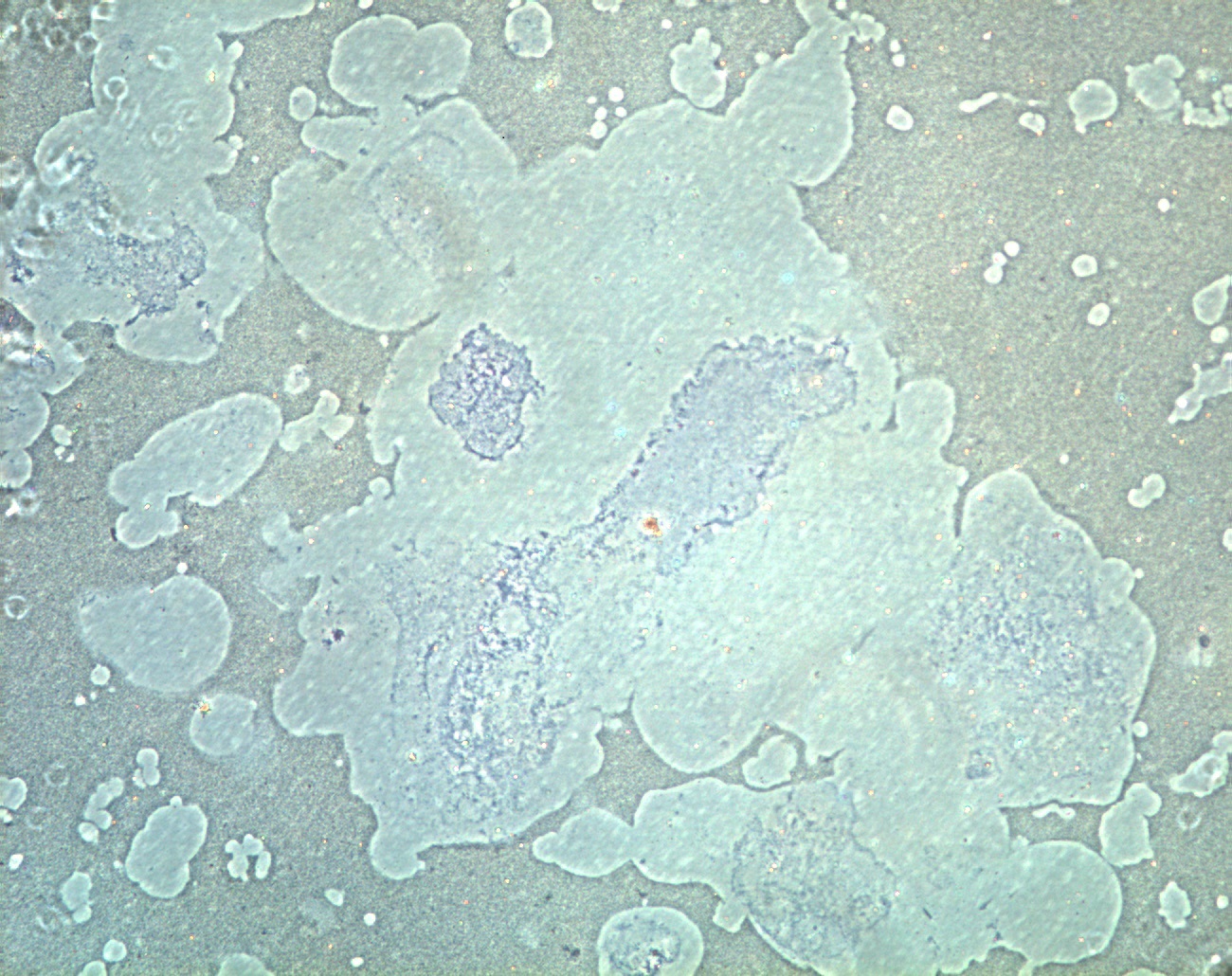

Supplement: Supplementary file 8 [file DataSheet5.ZIP › Fig.5-Source data/G/RANKL.jpg]

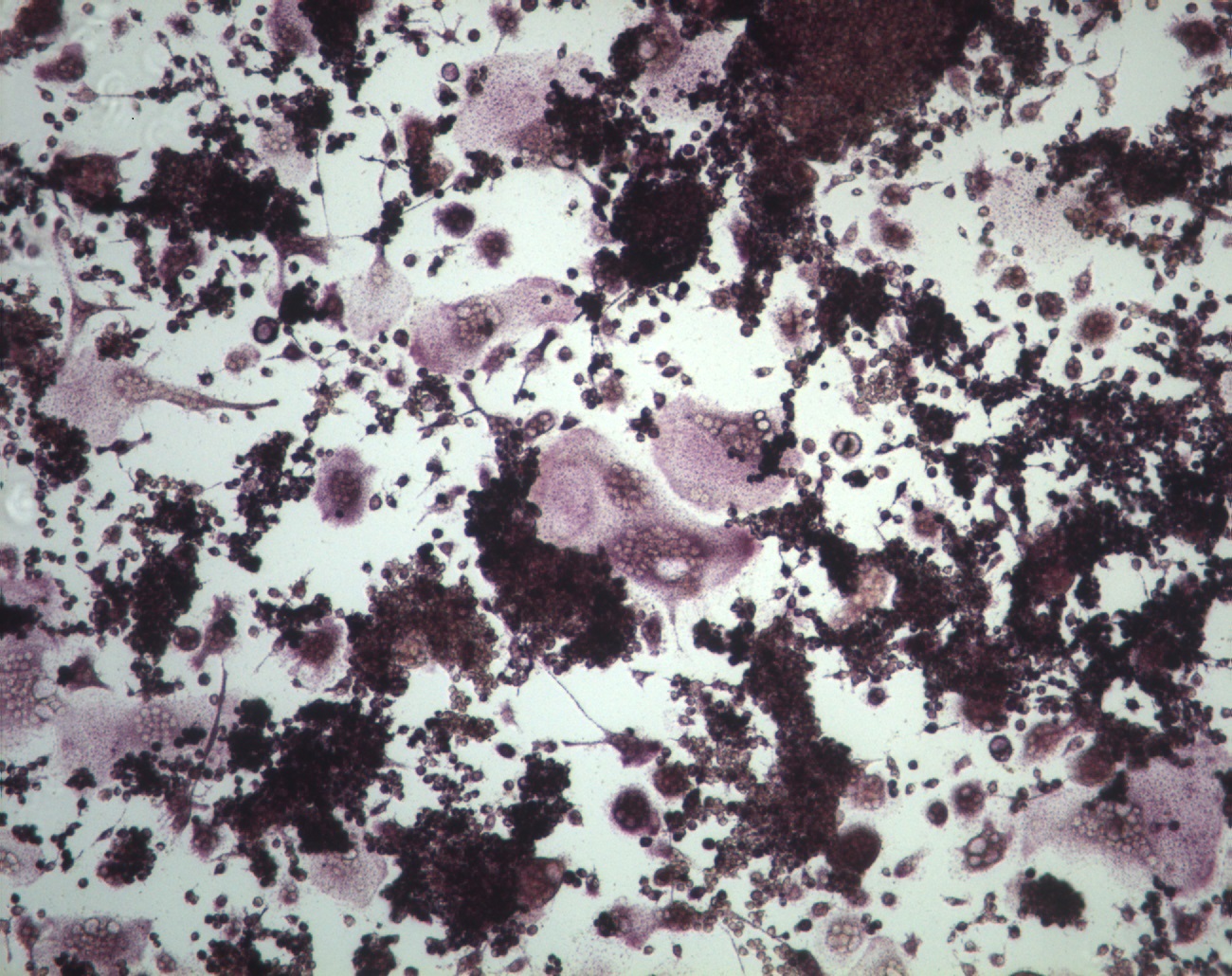

Supplement: Supplementary file 8 [file DataSheet5.ZIP › Fig.5-Source data/G/TRAP-RANKL+NAC (10 mM).jpg]

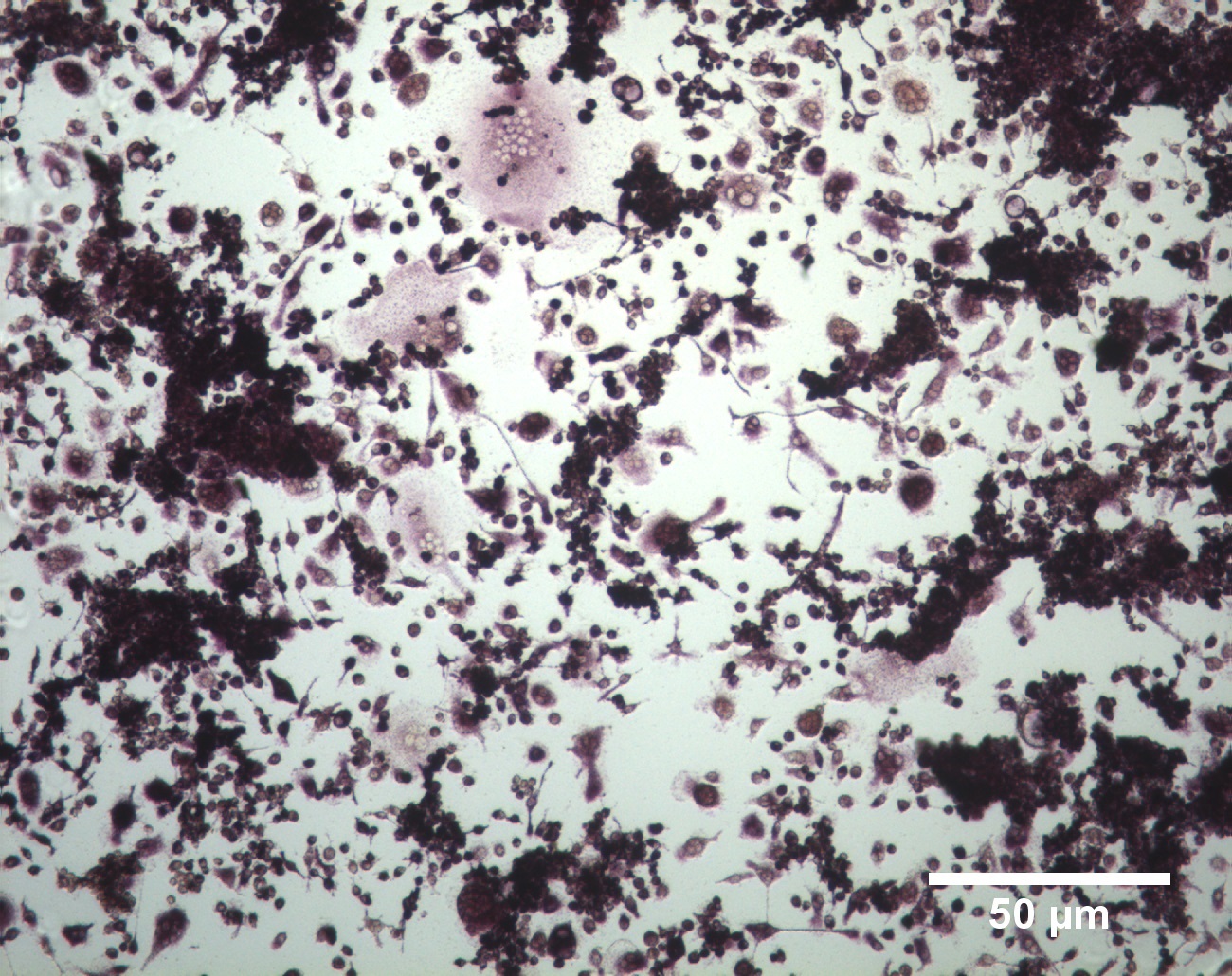

Supplement: Supplementary file 8 [file DataSheet5.ZIP › Fig.5-Source data/G/TRAP-RANKL+NAC (20 mM).jpg]

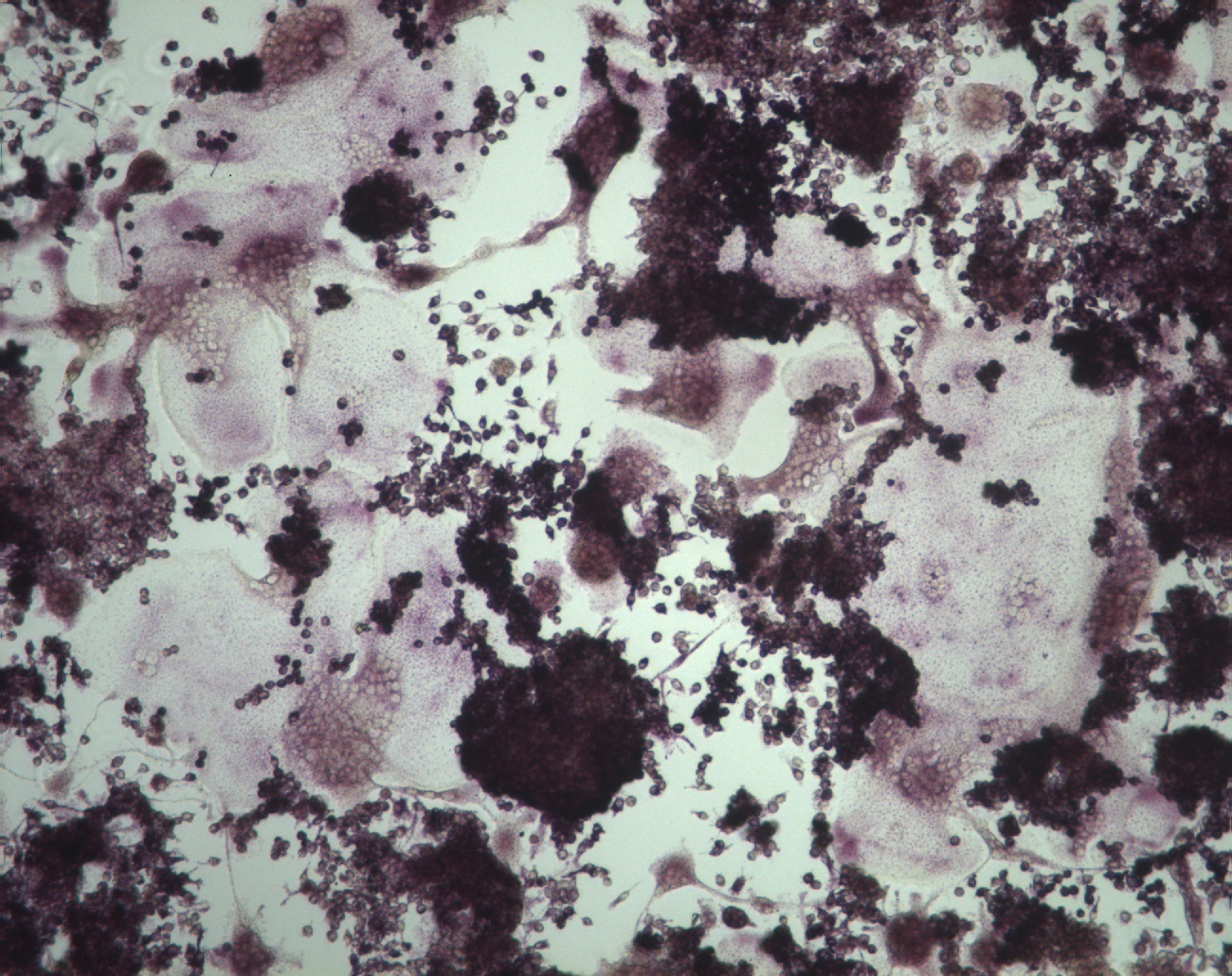

Supplement: Supplementary file 8 [file DataSheet5.ZIP › Fig.5-Source data/G/TRAP-RANKL.jpg]

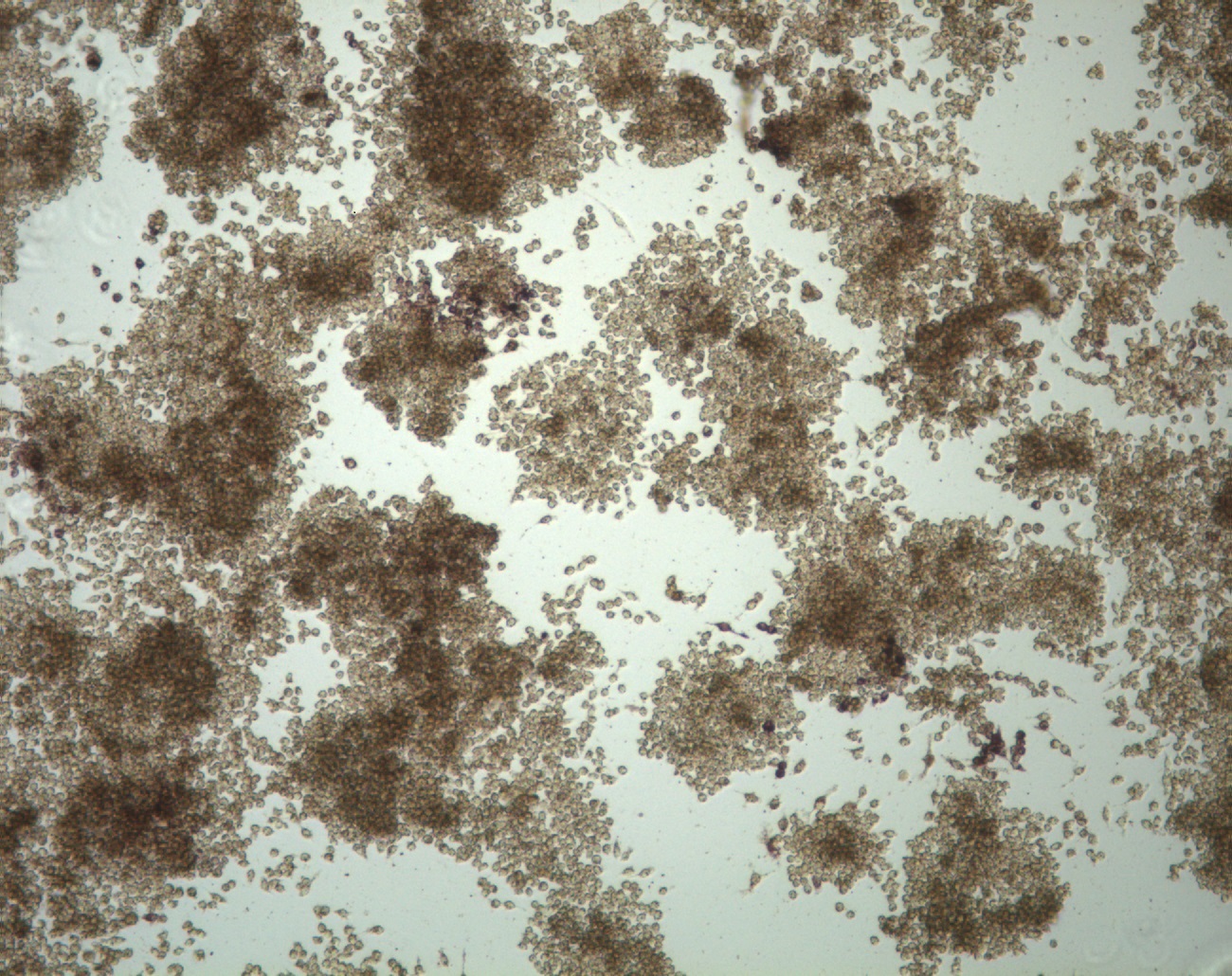

Supplement: Supplementary file 8 [file DataSheet5.ZIP › Fig.5-Source data/G/TRAP-control.jpg]

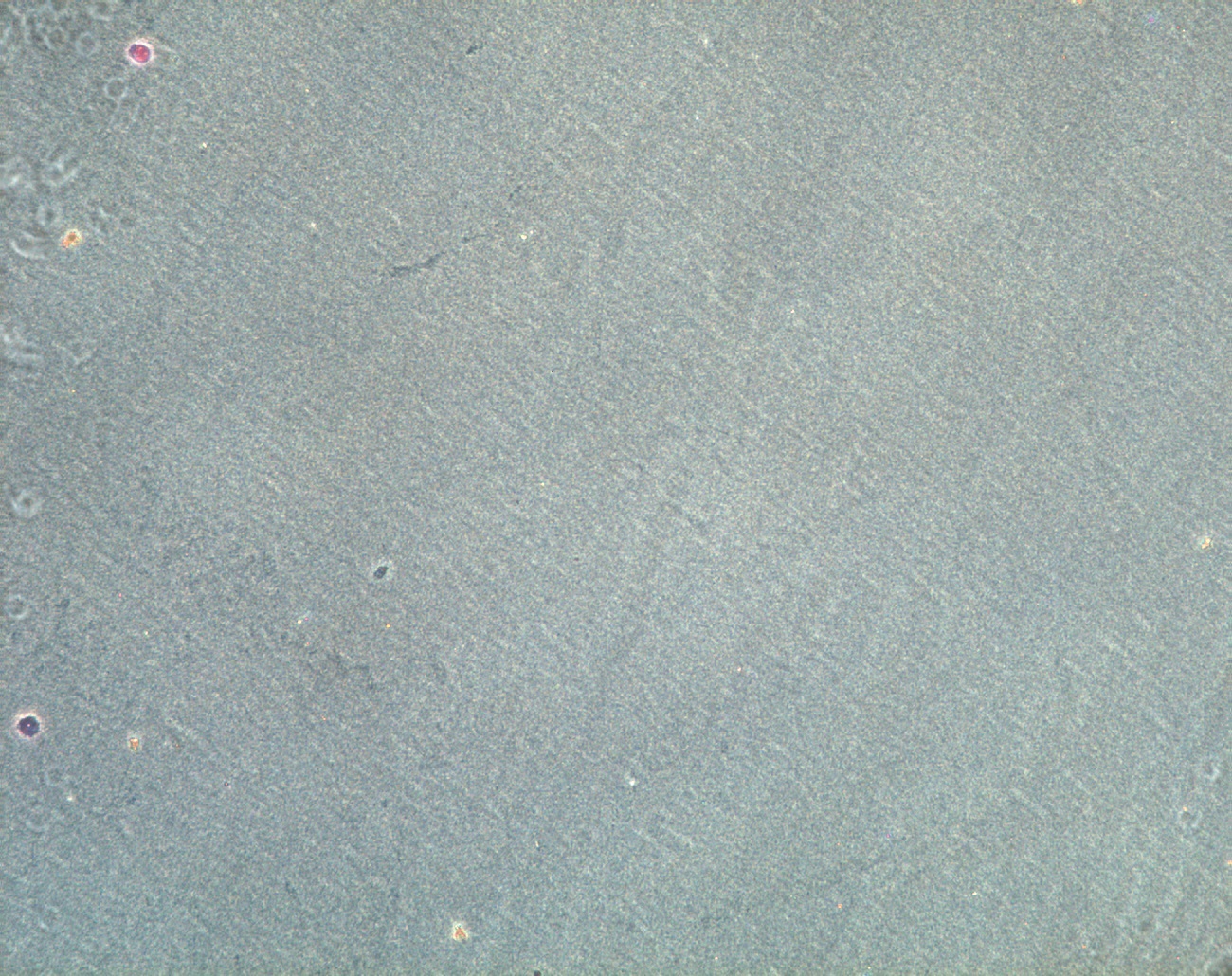

Supplement: Supplementary file 8 [file DataSheet5.ZIP › Fig.5-Source data/G/control.jpg]
