# Supplementary figures and images for: Nox4 Promotes RANKL-Induced Autophagy and Osteoclastogenesis via Activating ROS/PERK/eIF-2α/ATF4 Pathway (part 3 of 3)
Source: Front Pharmacol. 2021 Sep 28;12:751845. doi: 10.3389/fphar.2021.751845 (PMC8505706; doi:10.3389/fphar.2021.751845)

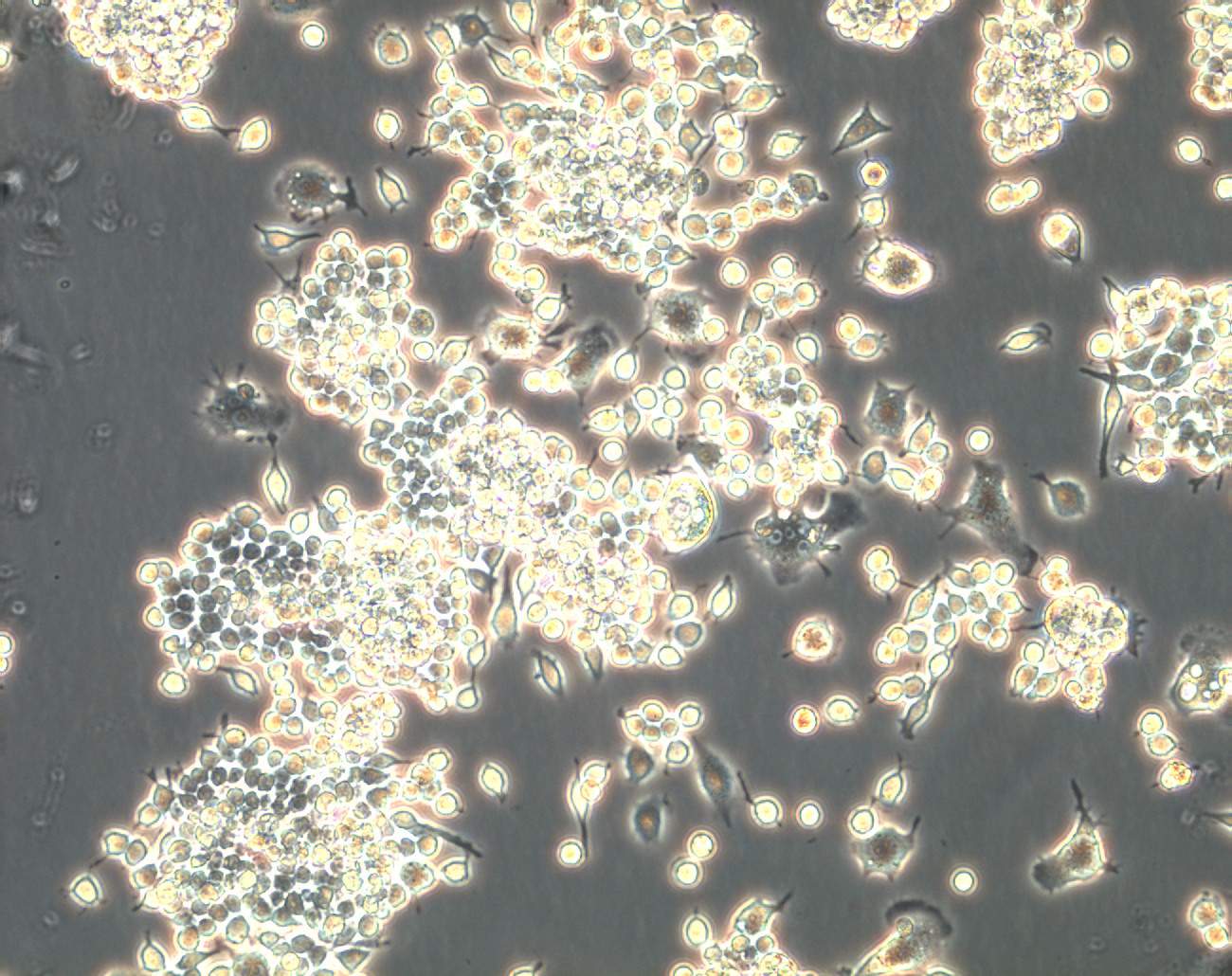

Supplement: Supplementary file 9 [file DataSheet7.ZIP › Supplementary Fig.1-Source data/A/RANKL.tif]

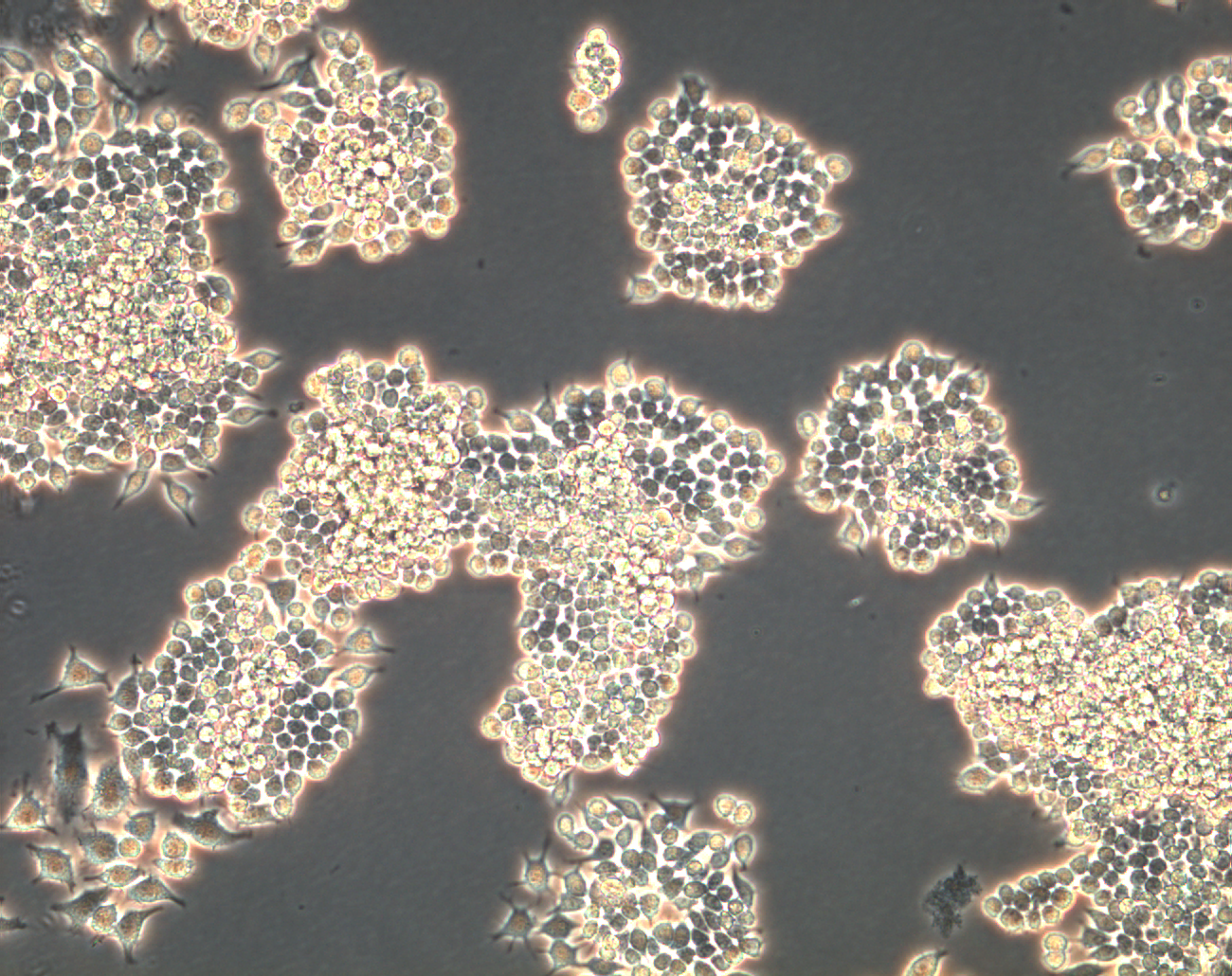

Supplement: Supplementary file 9 [file DataSheet7.ZIP › Supplementary Fig.1-Source data/A/control.tif]

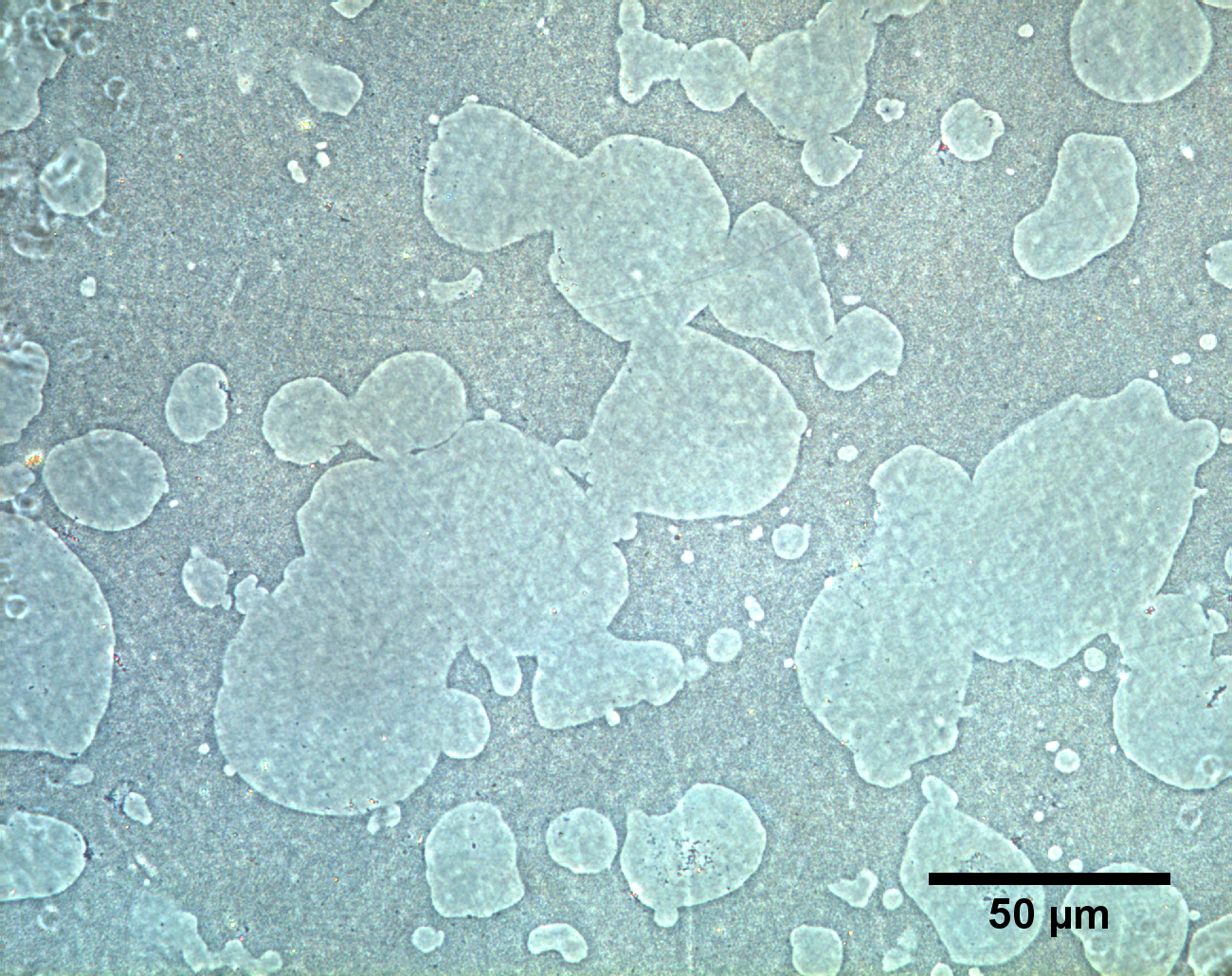

Supplement: Supplementary file 9 [file DataSheet7.ZIP › Supplementary Fig.1-Source data/C/RANKL.tif]

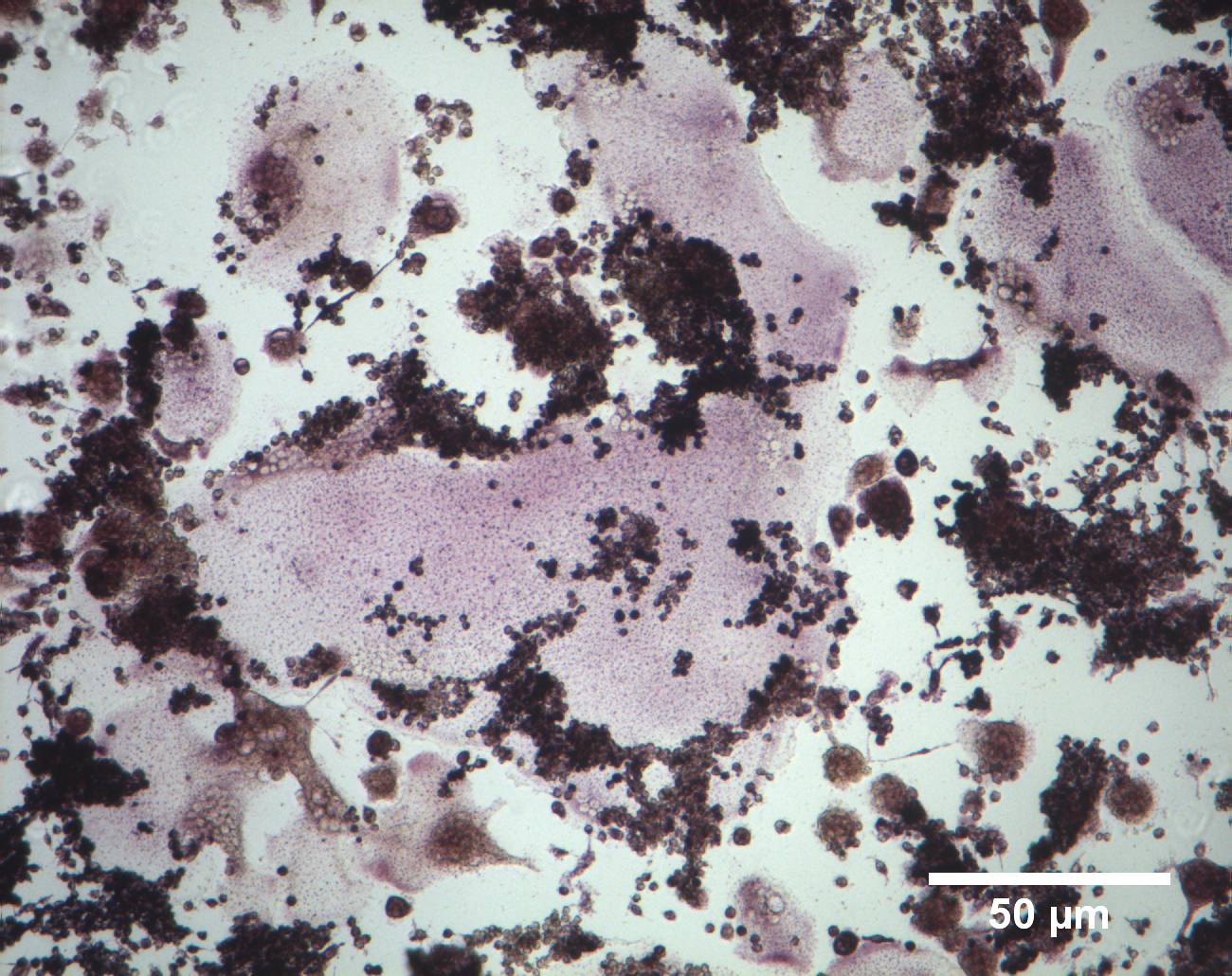

Supplement: Supplementary file 9 [file DataSheet7.ZIP › Supplementary Fig.1-Source data/C/TRAP-RANKL.tif]

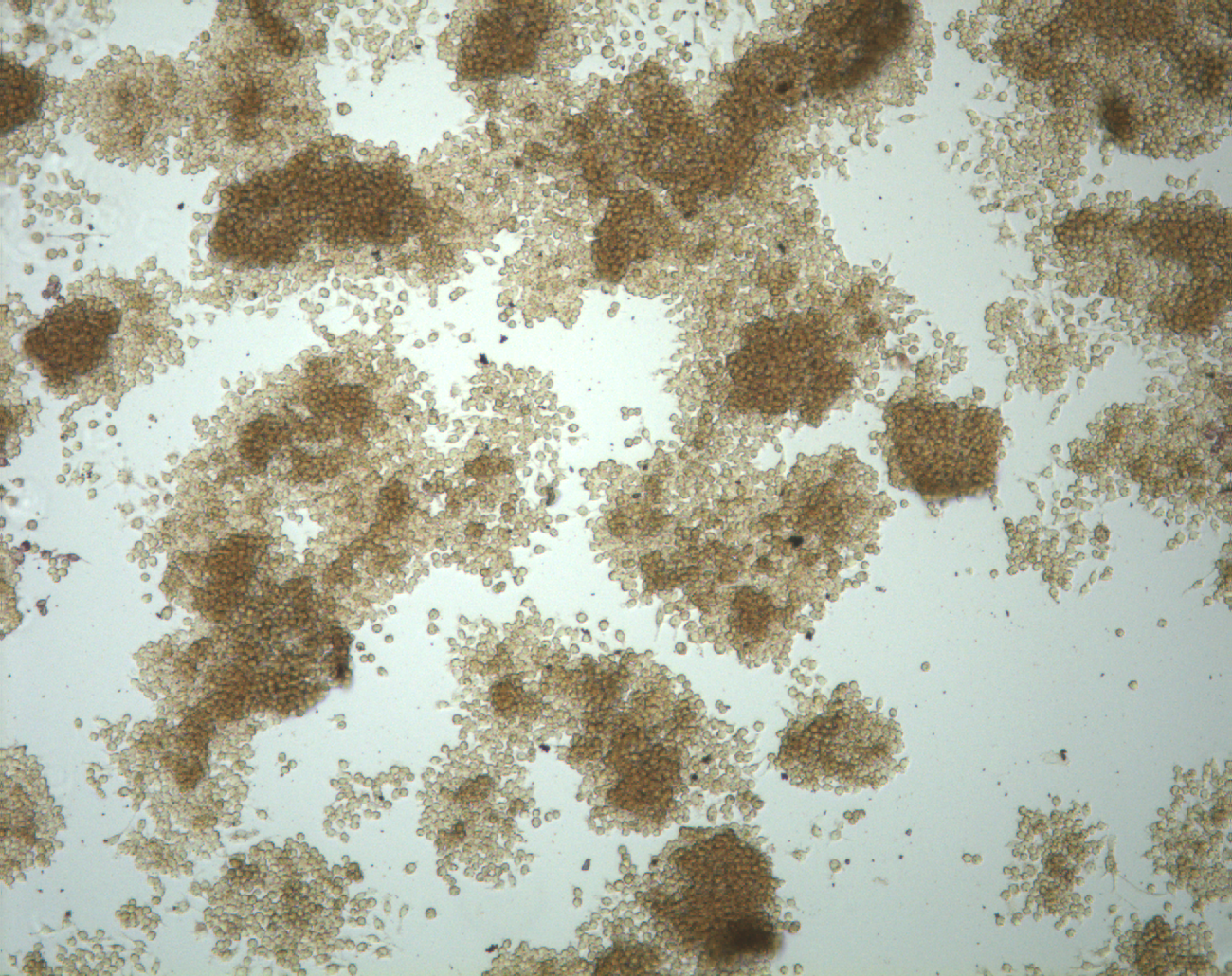

Supplement: Supplementary file 9 [file DataSheet7.ZIP › Supplementary Fig.1-Source data/C/TRAP-control.tif]

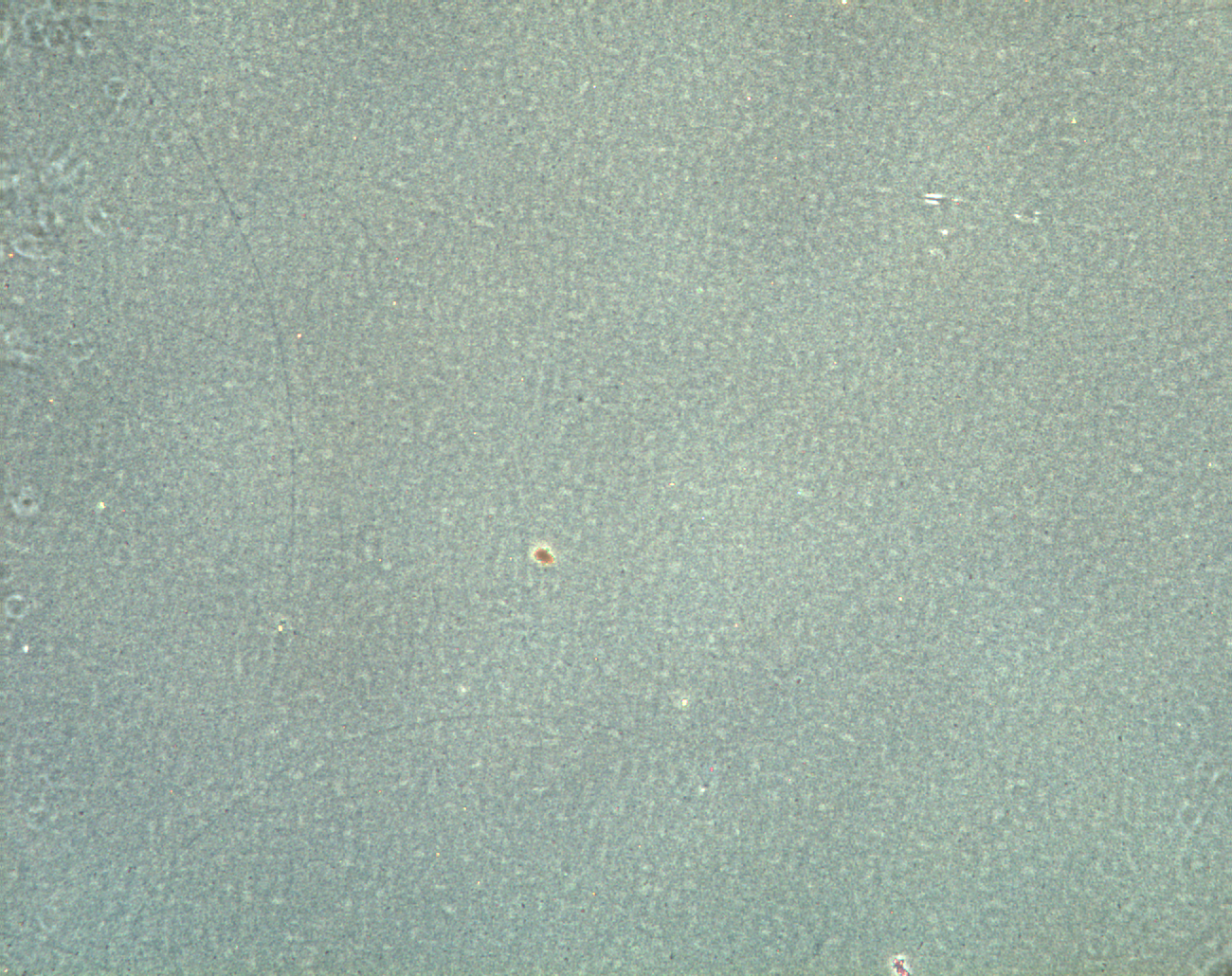

Supplement: Supplementary file 9 [file DataSheet7.ZIP › Supplementary Fig.1-Source data/C/control.tif]
